# Utilizing MIKC-type MADS-box protein SOC1 for yield potential enhancement in maize

**DOI:** 10.1007/s00299-021-02722-4

**Published:** 2021-06-06

**Authors:** Guo-qing Song, Xue Han, John T. Ryner, Addie Thompson, Kan Wang

**Affiliations:** 1 Department of Horticulture, Plant Biotechnology Resource and Outreach Center, Michigan State University, East Lansing, MI 48824 USA; 2 Department of Plant Soil and Microbial Sciences, Michigan State University, East Lansing, MI 48824 USA; 3 Department of Agronomy, Crop Bioengineering Center, Iowa State University, Ames, IA 50011-1051 USA

**Keywords:** Dwarf plant, Flowering, *SUPPRESSOR OF OVEREXPRESSION OF CONSTANS 1*, *Zea mays*

## Abstract

**Key message:**

Overexpression of *Zea mays SOC* gene promotes flowering, reduces plant height, and leads to no reduction in grain production per plant, suggesting enhanced yield potential, at least, through increasing planting density.

**Abstract:**

MIKC-type MADS-box gene *SUPPRESSOR OF OVEREXPRESSION OF CONSTANS 1* (*SOC1*) is an integrator conserved in the plant flowering pathway. In this study, the maize *SOC1* (*ZmSOC1*) gene was cloned and overexpressed in transgenic maize Hi-II genotype. The T_0_ plants were backcrossed with nontransgenic inbred B73 to produce first generation backcross (BC_1_) seeds. Phenotyping of both transgenic and null segregant (NT) BC_1_ plants was conducted in three independent experiments. The BC_1_ transgenic plants showed new attributes such as increased vegetative growth, accelerated flowering time, reduced overall plant height, and increased grain weight. Second generation backcross (BC_2_) plants were evaluated in the field using two planting densities. Compared to BC_2_ NT plants, BC_2_ transgenic plants, were 12–18% shorter, flowered 5 days earlier, and showed no reduction in grain production per plant and an increase in fat, starch, and simple sugars in the grain. Transcriptome comparison in young leaves of 56-day-old BC_1_ plants revealed that the overexpressed *ZmSOC1* resulted in 107 differentially expressed genes. The upregulated transcription factor DNA BINDING WITH ONE FINGER 5.4 (DOF5.4) was among the genes responsible for the reduced plant height. Modulating expression of *SOC1* opens a new and effective approach to promote flowering and reduce plant height, which may have potential to enhance crop yield and improve grain quality.

**Supplementary Information:**

The online version contains supplementary material available at 10.1007/s00299-021-02722-4.

## Introduction

Increasing crop production is key to feeding the future (Ash et al. 2010). Breeding efforts in both public and private sectors in agriculture have been made to increase yield through genetic manipulation of various traits such as biotic or abiotic stress resistance (Nelson et al. 2007; Tester and Langridge 2010). Of the proposed flowering pathway gene networks (Fornara et al. 2010; Hill and Li 2016), MADS-box genes play significant roles in the formation of floral meristem and floral organs (e.g., male and female gametophyte), the control of floral transition and flowering time, and the development of seed and fruit (Becker and Theissen 2003; Causier et al. 2002; Garcia-Marotoet al. 2003; Gramzow and Theissen 2010, 2013; Heijmans et al. 2012; Masiero et al. 2011; Ng and Yanofsky 2001; Parenicova et al. 2003; Theissen et al. 2000). In addition, MADS-box genes function in root growth, lateral root formation, and morphogenesis of other organs (Smaczniak et al. 2012; Tapia-Lopezet al. 2008; Teo et al. 2019; Yu et al. 2014; Zhang and Forde 1998). Manipulation of these MADS-box genes provides an alternative approach to modulating plant reproductive growth, with the potential to influence crop yield (Castelan-Munoz et al. 2019; Hill and Li 2016; Trevaskis 2018).

MADS-domain proteins differ in the domain following the C-terminus of the MADS-box (Gramzow and Theissen 2010; Heijmans et al. 2012; Masiero et al. 2011). Type-I MADS-box genes usually have a single exon and are further divided into three subfamilies, Mα, Mβ, and Mγ. Type-I MADS-domain proteins function in female gametophyte, embryo, and endosperm development, respectively (Masiero et al. 2011; Parenicova et al. 2003). Type-II MADS proteins are plant-specific MIKC proteins that have conserved MADS (M-), intervening (I-), keratin-like (K-), and C-terminal (C-) domains (Theissen et al. 1996). The MIKC proteins consist of MIKC* and MIKC^c^ (classical MIKC) subgroups and are key regulators in plant reproductive processes (Adamczyk and Fernandez 2009; Dreni and Kater 2014; Dreni and Zhang 2016; Gramzow and Theissen 2015; Liu et al. 2013; Smaczniak et al. 2012; Verelst et al. 2007). For example, six MIKC*-type genes [*AGAMOUS-LIKE 30* (*AGL30*), *AGL65*, *AGL66*, *AGL67*, *AGL94*, and *AGL104*] playing a significant role in regulating pollen development have been identified in *Arabidopsis thaliana* (Kwantes et al. 2012; Liu et al. 2013; Verelst et al. 2007). The MIKC^c^ genes are divided into 13 major gene clades that play specific roles in the ABC model of floral development and in timing plant flowering (Amasino 2010; Heijmans et al. 2012; Lee and Lee 2010; Smaczniak et al. 2012; Wellmer and Riechmann 2010). Of the MIKC^c^ gene clades, *SUPPRESSOR of OVEREXPRESSION OF CONSTANS 1* (*SOC1*) and *SOC1*-like genes in the TM3/SOC1 clade are major flowering pathway integrators that harmonize flowering signals from multiple pathways (Alter et al. 2016; Lee and Lee 2010; Pin et al. 2010; Wellmer and Riechmann 2010). Modulating expression of *SOC1* can change the overall process of floral induction and flowering control, thus affecting crop yield.

Due to their significance in plant development (Gramzow and Theissen 2010; Schilling et al. 2018), several MADS-box genes have been patented for their regulatory roles in enhancing agronomic traits (Bae et al. 2011; Cacharron et al. 2000; Danilevskaya and Bruce 2008; Giovannoni et al. 2013; Podila et al. 2005; Takatsuji and Kapoor 2002), of which a maize *ZMM28* MADS-box gene (patent application # WO2008148872A1) has been patented for yield increase through its overexpression (Danilevskaya and Bruce 2008). The *ZMM28* gene is a homolog of the *Arabidopsis FRUITFUL* (*FUL*)/ *AGAMOUS*-like 8 (*AGL8*) gene, and it regulates maize flowering (Munsteret al. 2002). Increasing the expression of the *ZMM28* gene enhanced grain yield in the field (Wu et al. 2019). In 2019, USDA APHIS released a petition from DU PONT Pioneer Hi-Bred for determination of nonregulated status for enhanced grain yield potential and glufosinate-ammonium resistant DP202216 maize after safety evaluation of the maize ZMM28 protein (Anderson et al. 2019a, b; Catron 2019).

The SOC1 protein is a MIKC protein. In *Arabidopsis*, the *SOC1* gene is a positive regulator of the downstream MADS-box genes such as *APETALA1* (*AP1*) and *FUL*/*AGL8* (Lee and Lee 2010). Maize (*Zea mays*) SOC1 gene (*ZmSOC1* or *ZmMADS1*) is a flowering activator (Alter et al. 2016). The effect of overexpression of the *ZmSOC1* on maize grain production has not been reported. In this report, we describe the potential of using the *ZmSOC1* to enhance maize grain yield. We present phenotypic data of the first (BC_1_) and second (BC_2_) generations of backcross (BC) plants. These data provide evidence that the constitutive expression of *ZmSOC1* can result in enhanced yield potential through (1) hastening plant growth, (2) promoting flowering, (3) reducing overall plant height, (4) shortening overall time (about 2 weeks) needed from sowing to harvest, and (5) increasing or having no reduction in grain weight per plant. We also provide RNA-seq data derived from young leaves of six transgenic and three nontransgenic BC_1_ lines to reveal the overall impact of *ZmSOC1* overexpression on expression of other associated genes.

### Materials and methods

#### Constructs and plant transformation

Seeds of maize inbred line B104 were germinated to grow to 2-week old plants to harvest leaves. Isolation of total RNA was conducted using the RNeasy Mini Kit (Qiagen, Valencia, CA, USA). The RNA sample was treated by DNase. Reverse transcription of RNA to cDNA was performed using SuperScript II reverse transcriptase (Invitrogen, Carlsbad, CA, USA).

Sequence of the maize SOC1 gene (*ZmSOC1* or *ZmMADS1*) has been published in GenBank (accession number: NM_001111682.1) (Alter et al. 2016). The *ZmSOC1* sequence is also available in GenBank (accession number: HQ858775.1). Two pairs of polymerase chain reaction (PCR) primers included in Table S1 were designed to amplify the *ZmSOC1* from the cDNA of the leaf tissues of maize inbred line B104. The first pair of primers, MK_F and MK_R, were used to amplify the exact coding region of *ZmSOC1*. Using the second pair of primers, MK_F1_*Kpn* I and MK_R1_*Xba* I, unique restriction enzymes *Kpn* I and *Xba* I were added to the 5′- and 3′-end of the *ZmSOC1*, respectively. The PCR products were double digested using *Kpn* I and *Xba* I. Meanwhile, the pKANNIBAL plasmid was digested using *Kpn* I and *Xba* I to remove the PDK intron. The digested PCR products and the pKANNIBAL without the intron were ligated to insert the *ZmSOC1* gene between the CaMV 35S promoter and the *Ocs* terminator (Fig. S1). The ligated pKANNIBAL-*ZmSOC1* plasmid was digested using *Not* I to release the CaMV 35S-*ZmSOC1-Ocs* expression cassette. The released cassette was blunted using Klenow enzyme. Binary vector pTF101.1 (Paz et al. 2004) was double digested by *Hin*d III and *Eco*R I, and the sticky ends of the digested pTF101.1 plasmid were blunted using Klenow enzyme. The blunt end pTF101.1 fragment and the blunt end CaMV 35S-*ZmSOC1-Ocs* expression cassette were ligated to generate pTF101.1-*ZmSOC1* for overexpression of the *ZmSOC1* (herein *ZmSOC1-*OX) (Fig. S1). The pTF101.1-*ZmSOC1* contains the *bialaphos resistance* (*bar*) gene under the CaMV 35S promoter for selection of transformed plant cells using glufosinate (GS) herbicide. The *ZmSOC1* in the resulting pTF101.1-*ZmSOC1* were sequenced. Sanger sequencing data confirmed that a 696-bp *ZmSOC1* derived from the cDNA of the maize inbred line B104 was successfully inserted into the binary vector pTF101.1-*ZmSOC1.* In the 696-bp *ZmSOC1* sequence, 694 bp are identical to the published 696-bp reference deposited in the GenBank (accession numbers HQ858775.1 and NM_001111682.1). The protein sequence of the cloned *ZmSOC1* is identical to that derived either from the HQ858775.1 or from a part of the NM_001111682.1. The pTF101.1-*ZmSOC1* verified through sequencing was used for maize transformation (Fig. S1).

The pTF101.1-*ZmSOC1* was transformed into *Agrobacterium tumefaciens* strain EHA101. The construct was introduced into maize using *Agrobacterium*-mediated immature embryo infection method of Hi-II (A188 × B73) genotype (Frame et al. 2015). The T_0_ transgenic Hi-II plants were backcrossed with nontransgenic inbred line B73 to produce first generation of backcross (BC_1_) seeds, which have about 75% of the B73 genetic background.

### Phenotyping of the BC_1_ plants grown in pots

In each experiment, ten BC_1_ lines, each having more than one hundred seeds, were used for phenotyping in this study. BC_1_ seeds were germinated in water-soaked Suremix Perlite planting medium (Michigan Grower Products Inc., Galesburg, MI) in 4-inch plastic pots (8.9 cm width × 12.7 cm height) in a greenhouse in the springs of 2018 and 2019. Three experiments were conducted, including two in 2018 and one in 2019. Ten seeds per selected line for each experiment were sowed on May 17 and June 11 in 2018 and 16 seeds per line were sowed on May 11 in 2019. In each experiment, poor germinating lines with less than two plants were excluded for further phenotypic data collection in this study. Individual BC_1_ plants were transplanted to a 4-gallon pot (top diameter 30 cm, bottom diameter 24 cm, depth 27 cm) and the plants were grown in a secured courtyard under natural environmental conditions at Michigan State University, East Lansing, Michigan (latitude 42.701847, longitude −84.482170). All of the plants were irrigated every other day and fertilized once a week using fertilizer (N:P:K = 20:20:20). Young leaves of 30 to 40-day old plants, 0.5 g per plant, were collected separately for DNA isolation, frozen in liquid nitrogen, and stored in a freezer at −80 °C.

Phenotypic data collection included plant height, seed germination date, date of tassel appearance, date of silk appearance, the total number of stem nodes and leaves, the number of cobs, dry weight of aerial parts without ears, dry weight of ear(s) excluding husk(s), and dry weight of grain. Plant heights measured during plant growth refer to stalk heights from the soil surface to the node of the highest leaf. The final heights of the maize plants refer to stalk heights from the soil surface to the top of tassels at harvest time. All of the plants for each experiment were harvested at the same time after they reached full physiological maturity in late October. The aerial parts of each plant were collected in a paper bag and dried at 25 °C for over 2 months in the lab prior to weighing the dry weights of total aerial parts, cob(s), and grain. For the third experiment in 2019, the date that an ear reached mature color (Fig. S2) was recorded for each plant. To reduce bias during data collection, identification of transgenic and nontransgenic plants using PCR was mostly performed about 1 month before plant harvesting. In total, plants from nine transgenic lines in three experiments were used for phenotypic data collection.

### Field test of BC_2_ plants

BC_1_ seeds of transgenic lines c7 and c9 were sown in a greenhouse with temperatures above 21 °C and a 16-h photoperiod. For further seed production, B73 was used as the pollinator. Ten seeds for each of the B73, c7, and c9 were sown at one time for four times at 5-day intervals to make sure the flowering time of B73 was able to meet that of the c7 and c9. The presence/absence of the transgene in the seedlings was determined using PCR. Flowering time for all plants was recorded. All tassels of BC_1_ plants were removed. Pollen from the B73 plants was used to pollinate the transgenic plants. The ears of transgenic plants were covered using pollinating bags. BC_2_ seeds from each individual plant were harvested separately.

BC_2_ seeds from three c7 lines and three c9 lines were grown in the field. For each line, 30 plants were randomly grown in each of the six plots, including three plots at a high planting density of 40,000 plants/acre and another three at a low planting density of 32,000 plants/acre. Two extra rows of B73 plants for each plot were used as protective borders. A drip irrigation system was installed in the field for plant irrigation as needed. Phenotypic data collection, including flowering time, plant height, leaf number, ear dry weight, and grain dry weight, were conducted using the same procedures described above for the BC_1_ plants. In addition, grain quality from 50 plants were measured using a Grain Analyser (Infratec^™^ 1241, FOSS Analytical AB, Denmark).

### Detection of the transgenic plants

DNA was isolated from about 200 mg of leaf tissue for each sample using the cetyltrimethylammonium bromide (CTAB) method (Doyle and Doyle 1987). Maize *Actin* gene primers ZmAct_F and ZmAct_R were used as a control to verify the template DNA. Two pairs of primers, bar-F and bar-R for the *bar* gene, 35S-F (3′ portion of the *CaMV 35S* promoter) and MK_R for the *ZmSOC1* gene (Table S1), were used to detect the presence of transgenes in each sample. PCR reaction conditions for all primer pairs started with an initial denaturation for two min at 94 °C, 30 cycles of 45 s at 94 °C, 60 s at 58 °C and 90 s at 72 °C, and a final extension for 10 min at 72 °C. All amplified PCR products were separated on 1% agarose gel containing ethidium bromide and visualized and photographed under UV light (Fig. S3).

### RNA sequencing and transcriptome analysis

For RNA isolation, leaves from 56-day-old plants at a vegetative growth stage were harvested (Fig. S2), frozen immediately in liquid nitrogen, and stored at −80 °C in a freezer. Two transgenic lines, c7 and c9, were used. A total of nine samples from nine plants were used, including three transgenic and three nontransgenic null segregants from the c7 transgenic event and 3 transgenic plants from the c9 transgenic event. Total RNA of each sample was isolated from about 500 mg of young leaf tissue using a separate CTAB method (Zamboni et al. 2008) and purified using RNeasy Mini Kit (Qiagen, Valencia, CA, USA). On-Column DNase digestion with the RNase-free DNase Set was used to remove DNA in the RNA samples (Qiagen). RNA quality was determined using the High Sensitivity RNA ScreenTape system (Agilent technologies, Santa Clara, CA). All of the RNA samples used for RNA sequencing had a quality score greater than 5.0, and cDNA of all these samples were synthesized for reverse-transcription of 3–5 μg RNA per sample using SuperScript II reverse transcriptase (Invitrogen, Carlsbad, CA, USA). Regular RT-PCR was used to verify the expression of the transgenes before RNA sequencing. The reaction conditions for RT-PCR were 94 °C for 2 min, 35 cycles of 45 s at 94 °C, 60 s at 62 °C and 60 s at 72 °C, with a final 10 min extension at 72 °C. RT-PCR products were separated and visualized on 1.0% agarose gel containing ethidium bromide.

The RNA samples were sequenced (150 bp-paired end reads) using the Illumina HiSeq4000. All sequencing was performed at the Research Technology Support Facility at Michigan State University (East Lansing, Michigan, USA). The FastQC program (www.bioinformatics.babraham.ac.uk/projects/fastqc/) was used to assess the quality of sequencing reads for the per base quality scores. About 32–42 million reads (MR) for each of the nine biological samples with average scores ranging from 37 to 40 were obtained for transcriptome analysis. The paired-end reads (~ 100 MR in total) combined from one sample of the NT line and one sample for each of the two TR lines were used for transcriptome assembly to develop a maize transcriptome reference ZmTrinity using Trinity/2.8.5 (Haas et al. 2013). This transcriptome reference was anticipated to cover all potential isoforms in the sequence reads. The RNA-seq reads of three biological replicates for each of the c7 and c9 transgenic lines and one c7 line were analyzed. The paired reads were aligned to the transcriptome reference ZmTrinity, and the abundance for each of a single read was estimated using the Trinity command “align_and_estimate_abundance.pl”. The Trinity command “run_DE_analysis.pl-method edgeR” was used to conduct differential expression analysis (Haas et al. 2013). Differentially expressed transcripts (DETs) with a false discovery rate (FDR) value below 0.05 (*p *value < 0.001) were used for further analysis of different pathway genes of maize.

Pathway genes of nine phytohormones in *Arabidopsis*, including auxin, cytokinin, ABA, ethylene, gibberellin, brassinosteroid, jasmonic acid, salicylic acid, and strigolactones, were retrieved from RIKEN Plant Hormone Research Network (http://hormones.psc.riken.jp/). Similarly, pathway genes of sugar in *Arabidopsis* were identified. These *Arabidopsis* hormones, MADS-box, and sugar genes (Table S2) were used as queries to blast against the transcriptome reference ZmTrinity and the isoforms showing *e* values less than −20 were identified and used for transcriptome comparisons. Flowering pathway genes in *Arabidopsis* and cereals (Walworth et al. 2016) were used to analyze flowering-related DETs identified in this study. Cytoscape 3.8.2 was used to construct gene networks of overrepresented gene ontology (GO) terms for the selected DETs under BiNGO’s default parameters with selected ontology file ‘GOSlim_Plants’ or ‘GO_Full’ and selected organism ‘*A. thaliana*’ (Maere et al. 2005; Shannon et al. 2003). Most of the analyses were performed using the resources at the High Performance Computing Center at Michigan State University.

Quantitative RT-PCR (RT-qPCR) using SYBR Green system (LifeTechnologies, Carlsbad, CA) were conducted to check the selected transcripts. The primers were designed according to the RNA-seq data, and ZmActin1 was used to normalize the RT-qPCR results (Table S1). RT-qPCR was performed on a Roche LightCycler^®^ 480 Instrument II. The reaction conditions for RT-qPCR were 95 °C for 5 min, 45 cycles of 30 s at 95 °C, 45 s at 62 °C and 30 s at 72 °C. Transcript levels within samples were normalized to Actin. Foldchanges were calculated using 2^−∆∆Ct^, where ∆∆Ct = (Ct_TARGET_–Ct_NOM_)_transgenic_ – (Ct_TARGET_–Ct_NOM_)_nontransgenic_. Three biological samples and three technical replicates were used for the analysis of each transgenic and nontransgenic line.

### Statistical analysis

Statistical analysis of the phenotypic data was conducted using ANOVA and TukeyHSD in RStudio (Version 1.0.136).

## Results

### The overexpressed ZmSOC1 causes phenotypic changes

Inbred maize genotypes remain recalcitrant for genetic transformation (Yadava et al. 2016). In this study, we performed transgene analysis in a transformable Hi-II genotype, which has a hybrid genetic background of A188 and B73 (Frame et al. 2015). To produce seeds, T_0_ transgenic plants were pollinated with nontransgenic inbred B73 to obtain BC_1_ seeds. A total of 16 independent *ZmSOC1* overexpression events were brought to maturity. BC_1_ and BC_2_ seeds should have  ~ 75 and 87.5% of B73 genetic background, respectively. Because of the complex hybrid background, each of the BC_1_ and BC_2_ plants of Hi-II was not genetically identical. To evaluate the effect of the *ZmSOC1* transgene in diverse genetic backgrounds, nontransgenic, null segregant plants from nine independent BC_1_ lines were identified and used as negative controls in the BC_1_ experiments. These null segregants (NT) shared the similar genetic background as well as the tissue culture effects with the transgenic counterparts (TR) because they were derived from the same ears. For the field test of the BC_2_ plants, six lines were derived from two BC_1_ lines (c7 and c9), for which RNA-seq was conducted.

Three BC_1_ experiments under three environmental conditions were conducted in two summers to evaluate the impact of *ZmSOC1-*OX on the following physiological or agronomic traits: (1) seed germination time, (2) time of tassel appearance, (3) time of silk appearance, (4) days between tassel and silk appearance, (5) leaf number, (6) plant height, (7) dry weight (plant aerial material excluding ears), (8) dry weight (ear), and (9) dry weight (grain). These selected physiological or agronomic traits are important criteria to evaluate yield potential. Of the nine traits analyzed (Table 1, Table S3), germination time of the TR seeds (5.3 ± 1.7 days) was similar to that of the NR seeds (5.1 ± 9.5 days); this was the only unchanged trait observed consistently in all three experiments. Two lines, c7 and c9, which had higher numbers of both TR and NT plants (Table S3), were selected for a detailed phenotypic and transcriptomic analysis. For the field test of the 1080 BC_2_ plants, the results confirmed the accelerated flowering and reduced plant stature in the TR plants. In addition, the BC_2_ TR plants showed unreduced grain production per plant and increased contents of starch, fat, and simple sugars in the grain. These phenotypic changes, as well as the *ZmSOC1-*OX-induced differentially expressed genes (DEGs), provide encouraging data that implicate the potential of utilizing the *ZmSOC1-*OX gene to increase maize yield.

**Table 1:** Analysis of variance of phenotypic changes of nine independent BC_1_ lines

Traits	2018 Exp. #1			2018 Exp. #2			2019 Exp. #3^a^				All 3 Exp		
	TR	NR	Signif	TR	NR	Signif	TR	NR	Signif		TR	NR	Signif
Germination time (days)	4.4 ± 0.9	4.3 ± 0.7		4.2 ± 0.8	4.2 ± 0.5		6.7 ± 1.5	6.4 ± 1.7			5.3 ± 1.7	5.1 ± 9.5	
Time of tassel appearance	56.8 ± 2.9	58.9 ± 1.5	*	55.8 ± 2.5	54.4 ± 2.6		68.3 ± 5.6	73.9 ± 5.9	***		61.3 ± 7.2	62.8 ± 11.4	
Time of silk appearance (days)	61.1 ± 4.7	67.4 ± 1.6	***	61.9 ± 3.9	61.1 ± 4.4		75.4 ± 7.8	83.4 ± 5.9	***		67.3 ± 9.2	70.9 ± 11.4	*
Time of tassel to silk appearances (days)	4.3 ± 2.6	8.6 ± 2.2	***	6.1 ± 2.6	6.6 ± 2.8		7.2 ± 3.7	10 ± 5	*		6.0 ± 3.3	8.2 ± 4.0	***
Plant height	133.3 ± 19.5	162.3 ± 8.8	***	162.3 ± 14.4	187.4 ± 8.7	***	117.0 ± 10.0	125.5 ± 11.2	**		134.9 ± 23.8	159.0 ± 30.2	***
Leaf number	10.2 ± 1.0	11.2 ± 0.8	*	12.2 ± 1.1	11 ± 1.6	**	NA	NA			11.2 ± 1.5	11.1 ± 1.4	
Dry weight of plant aerial (excluding ear)	102.1 ± 45.1	162.3 ± 27.1	***	NA	NA		68.2 ± 32.9	83.5 ± 16.8			82.2 ± 41.6	106.4 ± 41.4	**
Ear dry weight (g)	255.7 ± 43.2	225.4 ± 95.6		234.1 ± 46.3	188.7 ± 50.7	**	75.8 ± 32.8	67.4 ± 47.1			174.3 ± 93.2	146.9 ± 87.4	
Grain dry weight (g)	208.9 ± 40.3	182.3 ± 82.3		211.0 ± 41.7	166.8 ± 47.2	**	60.5 ± 31.0	53.0 ± 42.9			147.5 ± 82.7	124.6 ± 77.9	
	*n* = 23	*n* = 9		*n* = 23	*n* = 25		*n* = 33	*n* = 22			**n = 79**	**n = 56**	

*n* number of plants

^a^Low temperatures during the flowering time

Signif. codes: ***0.001, ** 0.01, *0.05, 0.1

### Overexpression of ZmSOC1 promotes flowering

**Fig. 1:**
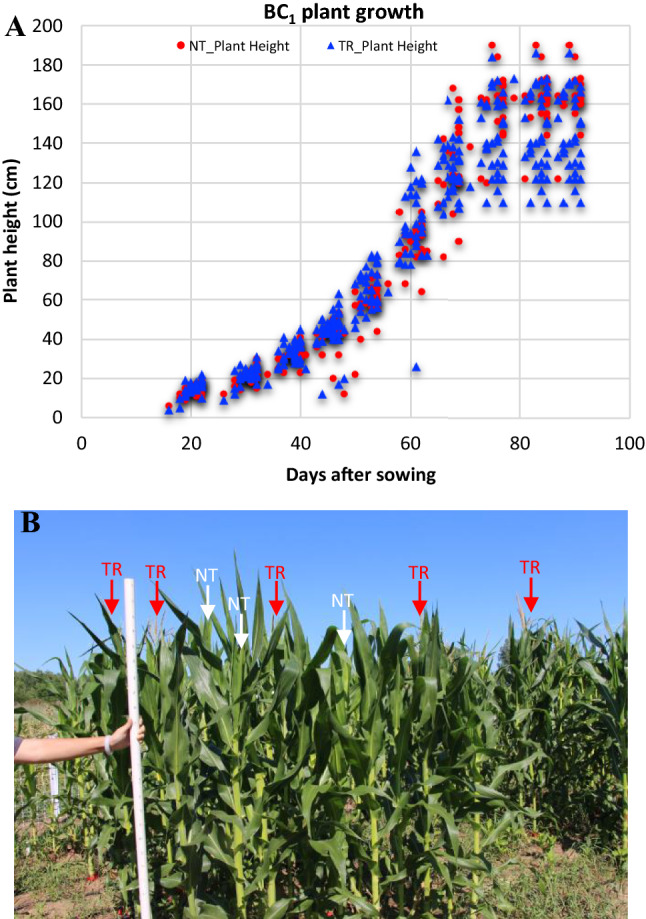
Growth of transgenic *ZmSOC1*-OX (TR) and null segregant (NT) maize plants of BC_1_ and BC_2_ lines. **A** Data from nine BC_1_ lines in experiment #3. **B** 60-day-old BC_2_ plants planted in a low-density field plot. Transgenic and nontransgenic plants were randomly grown in each plot

*ZmSOC1*-OX resulted in early flowering in both BC_1_ and BC_2_ plants. Upon close examination of BC_1_ TR and NT plants, we observed that the TR plants were taller than the NT plants before flowering in the first two experiments, but ended up shorter than the NT plants when they reached maturity (Table 1). For the BC_1_ lines tested in experiment #1 and #2 in 2018, the seeds were sown in mid-May and mid-June, respectively. To verify the observation, a third experiment was conducted in the summer of 2019. Seeds for experiment #3 were sown in mid-May, similar to experiment #1. We measured the heights of all plants from nine independent BC_1_ lines every 10 days from the 20^th^ to the 90^th^ day after sowing. The comparisons again showed that TR plants were taller than NT plants during the vegetative growth period (between 20 and 70 days after sowing) (Fig. 1A). After around day 70, NT plants grew taller, and mature TR plants were shorter than NT plants in the end (Table 1).

**Fig. 2:**
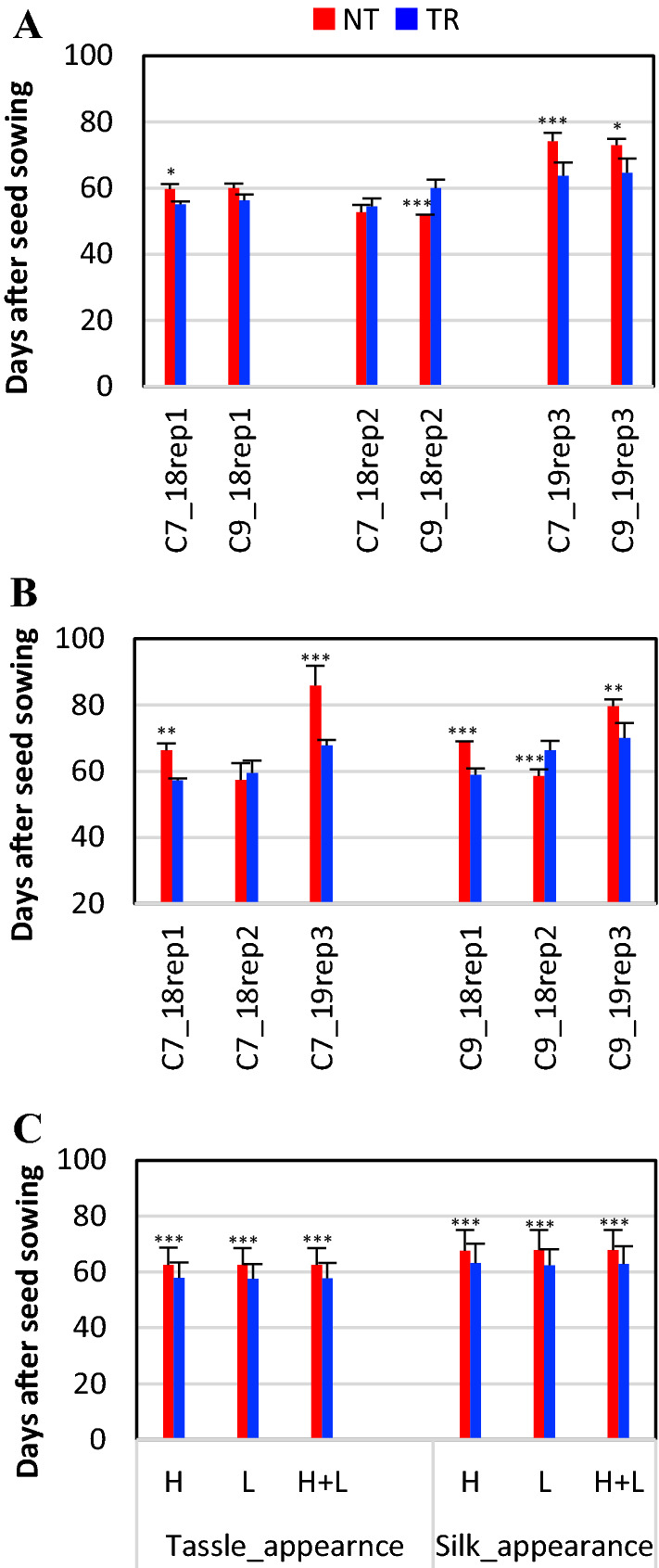
Flowering time of transgenic *ZmSOC1*-OX (TR) and null segregant (NT) maize plants of BC_1_ and BC_2_ lines. The BC_1_ plants were from two independent BC_1_ lines (c7 and c9). Rep1, rep2, and rep3 represent experiment #1, #2 and #3. **A** Tassel appearance time of BC_1_ lines. **B** Silk formation time of BC_1_ lines. Star(s) on each bar represent the comparison result between the TR and the NT plants of the same BC_1_ line in the same experiment, n for c7_18rep1 NT = 3, n for c7_18rep1 TR = 3, n for c7_18rep2 NT = 4, n for c7_18rep2 TR = 4, n for c7_17rep3 NT = 8, n for c7_19rep3 TR = 6, n for c9_18rep1 NT = 2, n for c9_18rep1 TR = 5, n for c9_18rep2 NT = 5, n for c9_18rep2 TR = 3, n for c9_19rep3 NT = 3, n for c9_19rep3 TR = 8. **C** Tassel and silk appearance time of BC_2_ lines. BC_2_ seeds from three c7 lines and three c9 lines were grown in a field. For each line, 30 plants were randomly grown in each of the six plots, including three plots for a high planting density of 40,000 plants/acre (*H*) and another three for a low planting density of 32,000 plants/acre (*L*). Star(s) on each bar represent the comparison result between the TR and the NT plants of the same planting density. Bars indicate standard deviation. Significance codes: *** < 0.001, ** < 0.01, *< 0.05

For the BC_1_ plants, slightly accelerated flowering, indicated by the time of tassel and silk appearance, was observed in TR plants in two of the three experiments. Tassels appeared 1 day earlier for TR (61.3 ± 7.2 days) than NT (62.8 ± 11.4 days) with no significant difference (Fig. 2A). Silks appeared three days earlier for TR (67.3 ± 9.2 days) than NT (70.9 ± 11.4 days) with a significance value of *p* = 0.05 (Fig. 2B). In addition, the time from the appearance of tassels to the appearance of silks was about two days shorter for the TR plants (6.0 ± 3.3 days) than that of the NT plants (8.2 ± 4.0 days) in all three experiments, which is highly significant (*p* = 0.001). The reduced nicking time between male and female flowers can facilitate an effective pollination that affects grain production. We also made backcrosses between B73 and TR plants to produce BC_2_ seeds for c7 and c9. All of the BC_1_ TR plants flowered 5–10 days earlier than the BC_1_ NR and B73 plants in the greenhouse. For the field test of the BC_2_ plants, dynamic changes of plant height at early plant growth stages were not measured. Regardless of the planting densities, early flowering (~ 5 days for the appearance of both tassels and silks) was observed for the TR plants (Figs. 1B, 2C). Overall, overexpression of *ZmSOC1* was able to promote flowering. The early flowering of *ZmSOC1*-OX plants grown under natural conditions either in pots for the BC_1_ or in the field for the BC_2_ TR plants is consistent with the results observed in the greenhouse-grown *ZmSOC1*-OX plants (Alter et al. 2016).

### Overexpression of ZmSOC1 reduces plant height

**Fig. 3:**
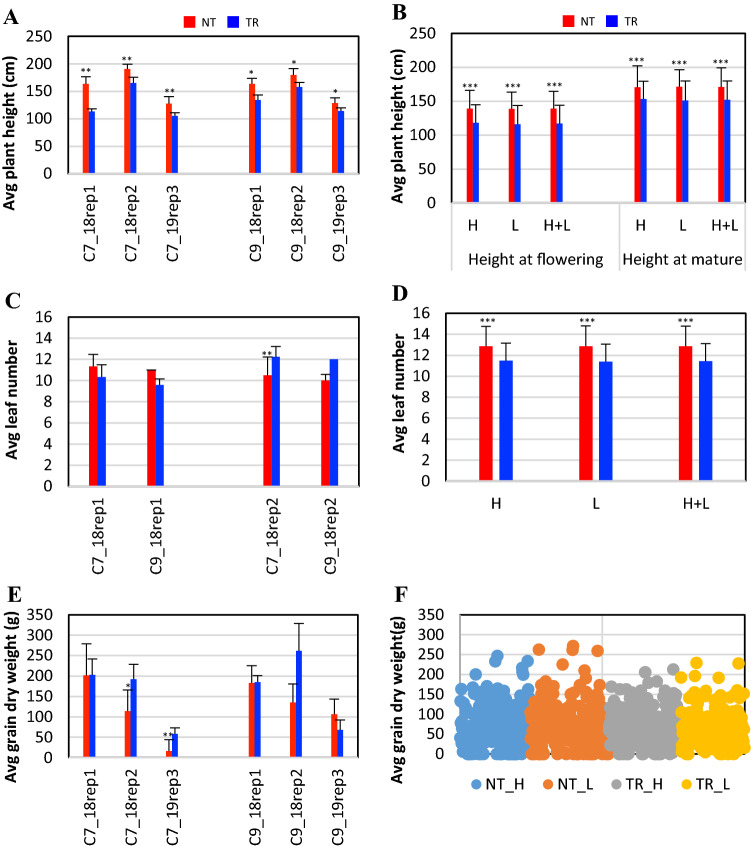
Plant height and leaf number of transgenic *ZmSOC1*-OX (TR) and, nontransgenic, null segregant (NT) maize plants of BC_1_ and BC_2_ lines. The BC_1_ plants were from two independent BC_1_ lines (c7 and c9). Rep1, rep2, and rep3 represent experiment #1, #2 and #3. BC_2_ seeds from three c7 lines and three c9 lines were grown in field. For each BC_2_ line, 30 plants were randomly grown in each of the six plots, including three plots for a high planting density of 40,000 plants/acre (*H*) and another three for a low planting density of 32,000 plants/acre (*L*). **A** Average plant height of mature BC_1_ plants. **B** Average leaf number of mature BC_1_ plants. **C** Average plant height of mature BC_2_ plants. **D** Average leaf number of mature BC_2_ plants. **E** Average grain dry weight per BC_1_ plant; average grain dry weight per BC_2_ plant. Star(s) on each bar represent the comparison result between the TR and the NT plants of the same BC_1_ line in the same experiment, n for c7_18rep1 NT = 3, n for c7_18rep1 TR = 3, n for c7_18rep2 NT = 4, n for c7_18rep2 TR = 4, n for c7_17rep3 NT = 8, n for c7_19rep3 TR = 6, n for c9_18rep1 NT = 2, n for c9_18rep1 TR = 5, n for c9_18rep2 NT = 5, n for c9_18rep2 TR = 3, n for c9_19rep3 NT = 3, n for c9_19rep3 TR = 8. For the BC_2_ plants, star(s) on each bar represent the comparison result between the TR and the NT plants of the same planting density. Bars indicate standard deviation. Significance codes: *** < 0.001, ** < 0.01, * < 0.05

Plant height of TR plants was significantly reduced (Fig. 3A, 3B, Table 1, Table S3). For mature BC_1_ plants, the average height from soil to tassels was 134.9 ± 23.8 cm for TR plants but 159 ± 30.2 for NT (*p* = 0.001) (Fig. 3A). For BC_2_ plants, the height of TR plants was shorter than NT plants at the time of both tassel appearance (TR 117.4 ± 26.9 cm vs. NT 140.0 ± 25.7 cm) and maturity (TR 152.3 ± 27.5 cm vs. NT 171.2 ± 28.1 cm) (Fig. 3B).

For leaf number in the BC_1_ TR plants, a significant decrease and a significant increase were observed in experiment #1 and experiment #2, respectively (Table 1). For the c7 and c9 lines, TR plants had 1 and 1.4 fewer leaves than NT plants, respectively, in experiment #1 (seed sown in mid-May 2018; Fig. 2B). In contrast, TR plants showed 1.8 and 2 more leaves than the NT plants, respectively, in experiment #2 (seed sown in mid-June 2018). The inconsistency is likely due to the planting times that caused variations in the plant growth conditions under natural environmental conditions. Consequently, the averages of the leaf numbers for BC_1_ plants in two experiments were similar for both TR (11.2 ± 1.5 leaves) and NT (11.1 ± 1.4 leaves) plants (Fig. 3C). For the BC_2_ plants, the leaf number of TR plants (11.4 ± 1.7 leaves) was less than the NT plants (12.9 ± 1.9 leaves) (Fig. 3D). Overall, it was reasonable to conclude that overexpression of the *ZmSOC1* reduces leaf number because of the larger population of the BC_2_ plants. The results of both reduced plant height and leaf number in the BC_2_ TR plants grown under the natural conditions are consistent with those reported in the greenhouse-grown BC_1_ plants (Alter et al. 2016).

### Overexpression of ZmSOC1 increases grain dry weight per plant

Yield potential of the *ZmSOC1*-OX plants was not reported (Alter et al. 2016). For the BC_1_ plants, the increased dry weight for both ear and grain was recorded in TR plants in all three experiments, with significant difference in one experiment (Table 1, Table S3). Average dry weight of ear was over 27 g heavier for TR plants (174.3 ± 93.2 g) when compared to NT plants (146.9 ± 87.4 g). A nearly 23 g increase in average grain weight in TR plants (147.5 ± 82.7 g) versus NT plants (124.6 ± 77.9 g) was observed. For the c7 line, the grain dry weight of the TR plants showed a slight increase in experiment #1 and #3 and a significant increase in experiment #2 (Figs. 2F, 3, Fig. S2C). For the c9 line, grain dry weight of the TR plants showed no significant difference in all three experiments (Fig. 3E).

On the other hand, plants with shorter stature are desirable for high-density planting in the field to increase yield per acre. This laid the foundation for our field test of the BC_2_ plants using two planting densities. For each of the TR and NT BC_2_ plants, the two planting densities did not result in any significant effect on flowering time, plant height, leaf number, average of grain dry weight per plant, or grain quality (Figs. 2, 3). For all of the BC_2_ plants cross two planting densities, the average of grain dry weight per plant for TR plants (67.9 ± 45.5 g) was almost the same as that for NT plants (67.8 ± 53.4 g) (Fig. 3F). Notably, while the randomly mixed TR and NT plants grown in each plot were helpful to minimize the errors in this study, the NT plants had advantages over the TR plants in competing for light during the growing season because they were taller after flowering. The unreduced grain weight in the TR plants in the mixed NT/TR field was encouraging. This suggests that a higher yield potential could be achieved for the TR plants when a uniform population were tested in the field.

### Overexpression of ZmSOC1 affects grain quality

Whether or not an overexpressed MADS-box gene is able to change grain quality is not known. We tested the grain quality from 35 BC_2_ (12 NT and 23 TR) and 15 wild type B73 plants. TR grain showed a significant increase in starch (73.1 vs. 67.7% in the NT, *p* < 0.001), fat (4.5 vs. 3.9%, *p* < 0.01) and simple sugars content relative to NT grain (2.4 vs. 1.8%, *p* < 0.05) (Table S6). No significant changes were observed in protein, lignin, or other components.

### Overexpression of ZmSOC1 functions at the transcript level

To reveal the potential impact of *ZmSOC1-*OX on transgenic plants at transcript levels, we analyzed the transcriptomes in leaves from three TR plants for each of the c7 and c9 line and three NT plants for the c7 line, in which the c7 NT plants were genetically similar to c9 NT plants. Transcriptome analysis of BC_1_ TR and NT plants was conducted on two major purposes, including to verify the expression of the transgenes and to reveal the genes that responded to the expression of *ZmSOC1*. Three comparisons were made to reveal DETs induced by *ZmSOC1-*OX. The comparison of c7 TR and NT resulted in 473 DETs, of which 322 were annotated to 249 unique genes; c9 TR and NT comparison resulted in 2576 DETs, of which 1692 were annotated to 1136 unique genes; and c9 TR and TR comparison resulted in 1,127 DETs, of which 676 were annotated to 485 unique genes (Table S4). The difference of the total number of DETs in the comparisons of the c9 TR and c7 TR likely reflects the genetic background variations and potential transgene insertion elicited changes in the two lines. The shared DETs in the two comparisons, including c9_TR versus NT and c7_TR versus NT, could be the transcripts responding to the overexpression of *ZmSOC1*.

**Fig. 4:**
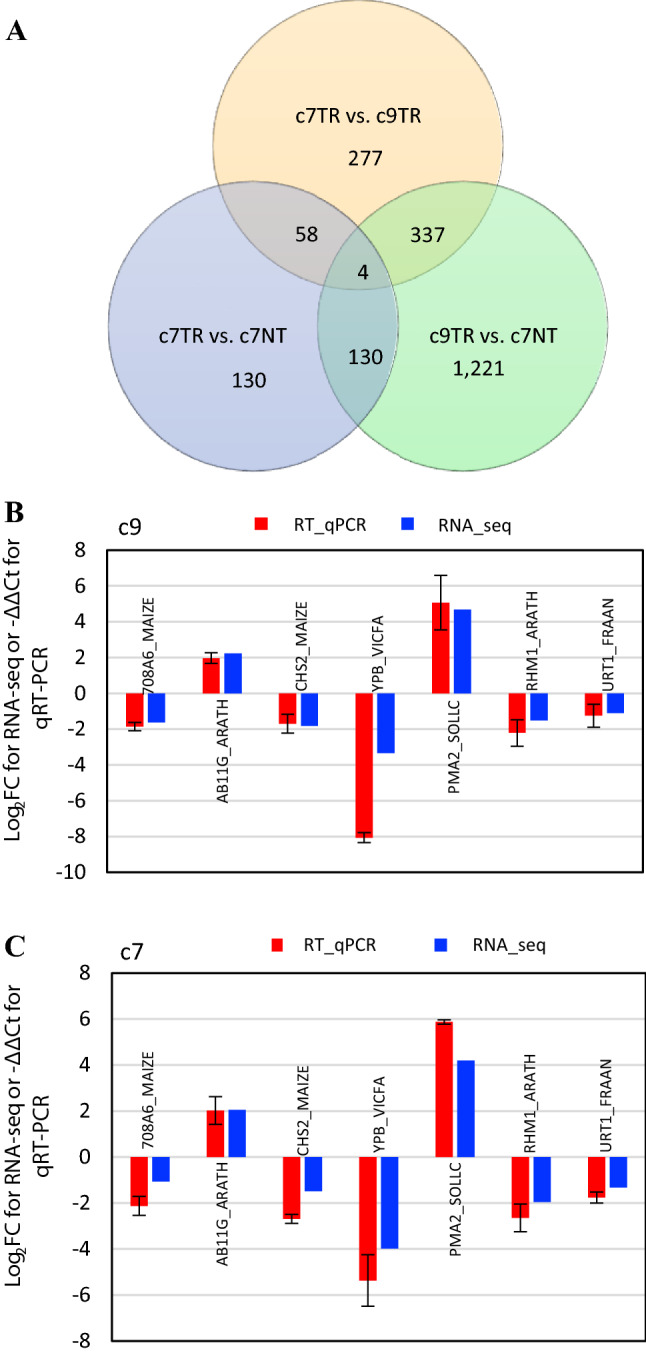
**A** Venn diagram illustrating overlap of the three transcriptomic comparisons of the annotated, differentially expressed transcripts (DETs) among BC_1_ null segregant c7NT and two transgenic *ZmSOC1*_OX lines of c7TR and c9TR. **B**, **C** Comparison of the RT-qPCR analysis result and the RNA-seq data of the selected DETs (Table 2, Table S5). −∆∆Ct is an average of three biological and three technical replicates for each DET. ZmActin1 (SAC1_ARATH) was used to normalize the RT-qPCR results. Bars indicate standard deviation

The Venn diagram in Fig. 4A illustrates the overlap of the DETs from the three transcriptome comparisons. There are 130, 277 and 1,221 DETs that are unique for c7_TR versus NT, c7_TR versus c9_TR and c9_TR versus NT, respectively. Fifty-eight DETs were found from both c7_TR versus NT and c7_TR versus c9_TR, 337 DETs were shared for both c7_TR versus c9_TR and c9_TR versus NT, and 130 DETs were shared for both c9_TR versus NT and c7_TR versus NT. Four DETs appeared to be present in all three transcriptome comparisons (Fig. 4A). These four DETs are NSE4A_ARATH (non-structural maintenance of chromosomes element 4), CP26B_ARATH (peptidyl-prolyl cis–trans isomerase), CHS2_MAIZE (chalcone synthase C2), and RA213_ARATH (ethylene-responsive transcription factor).

Of the annotated DETs, 134 DETs from 107 genes were shared in the two comparisons of c7_TR versus NT and c9_TR versus NT. Among them, 133 out of the 134 DETs were consistent in either up-regulation or down-regulation in the two comparisons (Table S5), including 96,861-fold and 127,973-fold increases of the overexpressed *ZmSOC1* (MAD50_ORYSJ) in c7 and c9 TR, respectively (Table 2). The high abundance of *ZmSOC1* transcript accumulation verified strong expression of *ZmSOC1* under the 35S promoter in both the c7 and c9 lines. The 134 shared DETs in the Venn diagram revealed the major genes affected by *ZmSOC1-*OX at the level of transcription (Table S5). From the 134 DETs, we picked primers to test seven selected DETs using RT-qPCR; the consistency of the RT-qPCR and RNA-seq results suggested that the RNA-seq data were reliable in this study (Fig. 4B, C).

**Table 2:** Differentially expressed transcripts (DETs) of flowering pathway and hormone genes in maize leaves during the fast-growing stage of the plants before flowering [Log_2_FC: Log_2_(Fold change) = Log_2_(TR/NT)

Maize_transcript _id	Annotation	Annotation_*e*_value	C7 DE_logFC	C9 DE_logFC	% identity	Blast*-e *value	Gene_ID	Gene_name	Pathway
DN20371_c2_g4_i2	DOF54_ARATH	8.89E-30	7.16	6.71	74.1	1.42E-24	AT2G34140.1	CYCLING DOF FACTOR 4 (CDF4)	Flowering
DN22669_c0_g2_i4	GIGAN_ORYSJ	0	9.05	8.86	63.9	0.00E + 00	AT1G22770.1	GIGANTEA (GI) (ZmGI)	Flowering
DN18903_c0_g1_i3	TRPA_MAIZE	2.69E-133	− 3.06	− 4.52	60.8	1.33E-113	AT4G02610.1		Auxin
DN19326_c1_g1_i1	C78A6_ARATH	1.43E-164	1.81	1.36	27.9	9.20E-48	AT4G31500.1	RNT1, RED1, SUR2, ATR4, CYP83B1	Auxin
DN19326_c1_g1_i2	C78A6_ARATH	9.29E-174	1.94	1.43	30.9	4.45E-55	AT4G31500.1	RNT1, RED1, SUR2, ATR4, CYP83B1	Auxin
DN18903_c0_g1_i1	TRPA_MAIZE	2.38E-103	− 3.38	− 5.05	51.1	2.41E-88	AT4G02610.1		Auxin
DN12409_c0_g2_i1	**708A6_MAIZE**	2.63E-144	− 2.13	− 1.86	29.5	2.69E-39	AT2G36800.1	UGT73C5, DOGT1	Brassinosteroid
DN9072_c0_g1_i3	**URT1_FRAAN**	5.49E-72	− 1.75	− 1.25	28.2	3.70E-32	AT2G36800.1	UGT73C5, DOGT1	Brassinosteroid
DN20315_c0_g6_i1	ACCO1_ORYSJ	2.70E-97	1.68	2.09	71.4	8.09E-78	AT1G62380.1	ATGA2OX8, GA2OX8	Ethylene
DN15903_c0_g1_i16	ACCO1_ORYSJ	1.03E-161	2.93	2.71	60.3	3.19E-125	AT1G62380.1	ATACO2, ACO2	Ethylene
DN22035_c0_g2_i1	AAMT3_MAIZE	0	− 2.98	− 3.93	25.7	1.54E-35	AT5G56300.1	GAMT2	Gibberellin
DN22319_c0_g1_i20	#N/A	#N/A	8.12	8.28	68.6	8.75E-44	AT3G24715.1	HCR1	
DN15759_c1_g2_i4	P2C27_ORYSJ	1.26E-151	8.45	9.01	36.1	2.60E-35	AT2G30020.1	AP2C1	
DN16626_c0_g3_i2	MAD50_ORYSJ	5.23E-116	− 2.03	− 1.41	62	3.97E-88	LOC_Os10g39130.1	OsMADS56 (ZmSOC1)	Flowering
DN16626_c0_g2_i8	MAD56_ORYSI	8.66E-105	− 1.42	− 1.36	69.4	8.03E-105	LOC_Os10g39130.1	OsMADS56 (ZmSOC1)	Flowering
DN21779_c0_g3_i1	HD3A_ORYSJ	2.60E-99	4.06	3.83	95.5	1.48E-103	LOC_Os01g11940.1	osFTL1 FT-Like1 (ZmFT)	Flowering
DN15967_c0_g7_i1	MAD15_ORYSJ	6.76E-32	4.54	4.68	98.2	1.71E-30	LOC_Os07g01820.1	OsMADS15 (ZmAP1)	Flowering
DN17448_c1_g1_i3	MAD15_ORYSJ	9.03E-120	5.92	5.58	73.2	9.67E-112	LOC_Os07g01820.1	OsMADS15 (ZmAP1)	Flowering
DN17448_c1_g1_i6	MAD15_ORYSJ	1.28E-114	6.98	5.84	73	7.65E-108	LOC_Os07g01820.1	OsMADS15 (ZmAP1)	Flowering
DN16626_c0_g3_i8^a^	MAD50_ORYSJ	1.12E-116	16.55	16.97	62	1.11E-88	LOC_Os10g39130.1	OsMADS56 (ZmSOC1)	Flowering
DN20803_c2_g1_i7	HSP7R_ARATH	0	− 1.91	− 1.56	30.4	2.03E-53	AT1G56410.1	HSP70T-1, ERD2	Sucrose

The bold DETs were verified by RT-qPCR. None of the DETs included in the table were the DETs in the comparison of the c7 TR and c9 TR *N/A* not annotated to known gene(s) or no *e *value

^a^Overexpressed ZmSOC1

**Fig. 5:**
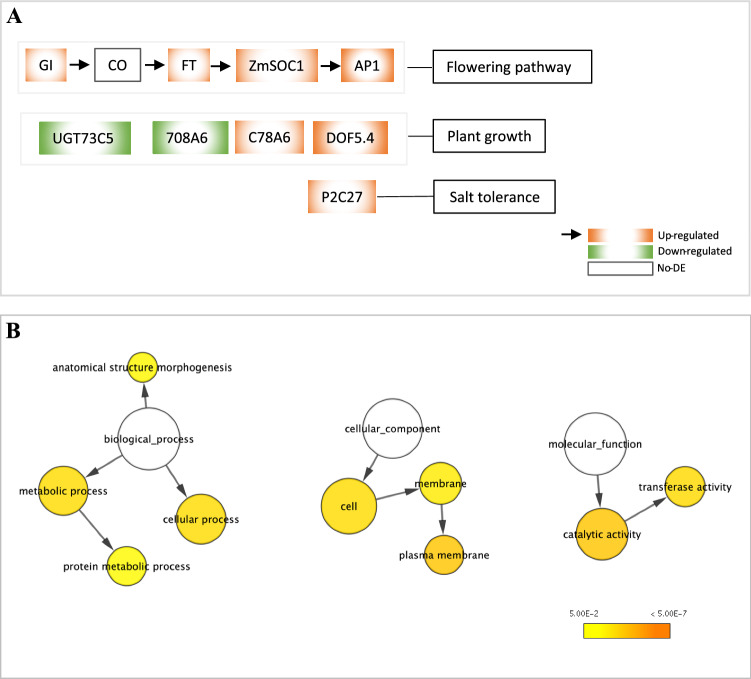
Major differentially expressed transcripts (DETs) and their potential functions (**A**) and gene networks of the shared DETs in leaf tissues of transgenic ZmSOC1_OX plants (**B**). The ontology file of GOSlim_Plants in BiNGO was used to identify overrepresented GO terms (*p* < 0.05). Bubble size and color indicate the frequency of the GO term and the *p *value, respectively

Based on the phenotypic changes observed in BC_1_ TR (such as earlier flowering and reduced plant stature, Table 1), we further analyzed the expression of flowering pathway genes and hormone-related genes. Twenty-one DETs of 14 genes were shared in the two comparisons of c7_TR versus NT and c9_TR versus NT (Table 2). In the flowering pathways, expression of two endogenous *ZmSOC1* genes was downregulated, and expression of four genes annotated as MAD15_ORYSJ, HD3A_ORYSJ, GIGAN_ORYSJ, and DOF54_ARATH was upregulated. MAD15_ORYSJ is an ortholog of *Arabidopsis AP1*, and HD3A_ORYSJ is an ortholog of *Arabidopsis FLOWERING LOCUS T* (*FT*). Both of them are positive regulators in plant flowering that interact directly with *SOC1* (Fornara et al. 2010). *GIGANTEA* (*GI*) is an upstream regulator of *CONSTANS* (*CO*) and *FT* in the circadian clock–controlled flowering pathway of *Arabidopsis*, and promotes the expression of the downstream flowering-time genes (Mizoguchi et al. 2005). GIGAN_ORYSJ, an ortholog of *GI*, showed increased expression of 530- and 465-fold for the c7 TR and c9 TR, respectively (Table 2). Overall, the highly upregulated GIGAN_ORYSJ, MAD15_ORYSJ (23–126 fold), and HD3A_ORYSJ (14–16 fold) and markedly reduced flowering time in both the c7 TR and c9 TR are likely the results of the overexpressed *ZmSOC1* in these plants (Fig. 5A).

The detected DETs in the pathways of auxin, brassinosteroid, ethylene, and gibberellin contributed to many observed phenotypes (e.g., reduced plant stature and the increased growth of the c7 and c9 TR plants during their vegetative growth) and nonvisible biochemical and physiological changes. DNA binding with one finger 5.4 (DOF5.4) is a transcription factor that negatively regulates cell cycle and cell expansion; enhanced expression of DOF5.4/OBP4 leads to plant dwarfing in *Arabidopsis* (Xu et al. 2016). In the c7 and c9 TR plants, DOF54_ARATH expression was upregulated to 143- and 104-fold, respectively (Table 2), supporting that the increase in expression of DOF54_ARATH may play a significant role in the reduced plant height of the c7 and c9 TR plants (Fig. 5A). Brassinosteroids (BRs) can promote plant growth and BR-deficient mutant plants often exhibit dwarfing (Fujioka and Yokota 2003; Grove et al. 1979; Kim et al. 2000; Mussiget al. 2003; Tanabe et al. 2005). In the BR pathway of *Arabidopsis*, UDP-GLYCOSYLTRANSFERASE 73C5 (*UGT73C5*) catalyzes inactivation of BRs and can lead to dwarfed BR-deficient plants (Poppenberger et al. 2005), which is often undesirable for crop yield. The DETs of both 708A6_MAIZE and URT1_FRAAN had high similarities (e < −20) to *UGT73C5*, and both transcripts showed decreased expression of 23–42% and had the potential for an increase in BRs that often benefits plant development (Table 2). It is possible that the decreased expression of 708A6_MAIZE and URT1_FRAAN was responsible for the increased growth of c7 and c9 TR plants during their vegetative growth. Of the auxin-related DETs, expression of TRPA_MAIZE with its function unknown was downregulated to 3–12%; expression of C78A6_ARATH was upregulated to 2.6–3.8 fold in the c7 and c9 TR plants (Table 2). Notably, the increased expression of C78A6_ARATH may result in increased size of leaves, flowers and seeds, but defects in reproductive development (Fang et al. 2012). Both the c7 and c9 TR plants did not exhibit defects in reproductive development, but leaf and seed sizes were not investigated in this study. PROBABLE PROTEIN POSPHATASE 2C 27 (P2C27_ORYSJ) triggers the expression of stress-responsive genes in *Arabidopsis* (Liu et al. 2012); the increased expression of maize *P2C27* could enhance salt tolerance in transgenic plants. Two ethylene-related, one gibberellin-related, and one sucrose-related DETs were also detected, although none of them seemed to have a close correlation with the phenotypic changes observed (Table 2). Overall, these major DETs driven by *ZmSOC1-*OX were the most likely candidates responsible for the phenotypic changes.

In addition to the DETs of flowering pathway and hormone-related genes, the other DETs also likely played a role in the overall phenotypic changes of the TR plants (Table 2, Fig. 5A, Table S5). To reveal the overall impact of the DETs detected in the c7 TR plants, GOSlim_Plants in Cytoscape 3.8.2 was used to visualize the overrepresented Gene Ontology (GO) terms. As shown in Fig. 5, a total of nine GO terms were overrepresented or enriched (*p* < 0.05). Four overrepresented terms in the biological process are “metabolic process”, “cellular process”, “protein metabolic process”, and “anatomical structure morphogenesis”, supporting that multiple biological processes were affected by the *ZmSOC1-*OX. Three overrepresented terms in the cellular component include “cell”, membrane”, and “plasma membrane”, suggesting that the phenotypic changes may be related to these cellular components. In the molecular function, two overrepresented GO terms are “transferase activity” and “catalytic activity” (Fig. 5B). More details of the overrepresented GO terms and their networks can be visualized using GO_full (Fig. S4). At a gene network level, the overrepresented GO terms reveal the overall impact of *ZmSOC1* overexpression on plant growth, flowering, yield potential, or grain quality.

## Discussion

Genetically modified (GM) crops for yield boosting are desirable to increase productivity without increasing land use, although they are not yet available on the market (ISAAA’s GM Approval Database. http://www.isaaa.org/gmapprovaldatabase/). In our efforts to produce GM crops for boosting yield, we chose the *SOC1* MADS-box gene as a target due to its significant role as a major integrator in the plant flowering pathway (Fornara et al. 2010; Lee and Lee 2010; Song and Chen 2018; Song et al. 2013). In this study, we cloned the *ZmSOC1* gene to explore the potential of utilizing this gene to increase maize grain production. The *ZmSOC1* was transformed into maize Hi-II. BC_1_ plants of nine transgenic lines were grown in three experiments to evaluate phenotypic changes of nine traits between transgenic and nontransgenic plants in each BC_1_ line population. We also conducted transcriptome analysis of six transgenic and three nontransgenic plants to reveal the DETs driven by *ZmSOC1* overexpression. We demonstrated that manipulation of the expression of *ZmSOC1* is a powerful approach to increase maize yield.

*SOC1* is a key pathway integrator and flower activator (Lee et al. 2000; Lee and Lee 2010; Moonet al. 2003). In monocots, functional *SOC1* orthologues have been identified and demonstrated to be flowering activators (Alter et al. 2016; Lee et al. 2004; Ryuet al. 2009). Functional analysis of maize *ZmMADS1* through both overexpression and RNA interference-mediated down-regulation has confirmed that maize *ZmMADS1* is a functional *SOC1* orthologue of maize (*ZmSOC1*) (Alter et al. 2016). In the overexpression experiment, transgenic plants (offspring of two lines) overexpressing the *ZmSOC1* using the maize ubiquitin promoter showed early flowering, decreased leaf number, and reduced plant height compared to nontransgenic maize plants under greenhouse conditions (Alter et al. 2016). Our data, based on the field evaluation of the BC_2_ plants from six lines, also found overexpression of *ZmSOC1* driven by 35S promoter was able to promote flowering and reduce plant height and leaf number. In addition, our work demonstrated that *ZmSOC1*-OX approach can be adopted to enhance maize yield potential.

Orthologues of *SOC1* in many plant species have been studied to reveal flowering mechanisms (Lee and Lee 2010). However, efforts using *SOC1* orthologues to increase crop yield have not been documented. Regardless of the results from potted plants in this study, the increased grain production per TR plant suggests that *ZmSOC1*-OX, at least in some transgenic lines, has potential to increase maize grain yield. Additionally, the reduced plant height suggests that TR plants can be planted at a higher density, potentially increasing maize grain yield per acre. More experiments are ongoing to investigate BC_2_ plants growing with different planting densities under field conditions.

MADS-box transcription factors (TFs) function at almost every aspect of plant reproductive development through a complex protein–protein interaction network (Hugouvieux and Zubieta 2018). In *Arabidopsis*, *CONSTANS* (*CO*) activates *SOC1* through *FT* to promote flowering (Yoo et al. 2005). In this study, comparative transcriptome analysis revealed that *ZmSOC1*-OX enhanced expression of maize *FLOWERING LOCUS T* (*ZmFT*) and maize *GIGANTEA* (*ZmGI*) genes. The result is similar to that observed in transgenic rice where rice *SOC1* was overexpressed (Lee et al. 2004). In maize, the orthologue of *FUL* (*ZMM28*) is the MADS-box gene considered to be a duplication of the maize *AP1* gene; it has been successfully used for increasing grain yield through overexpression (Wu et al. 2019). In this study, the overexpressed *ZmSOC1* enhanced expression of the downstream *AP1* MADS-box gene annotated as *ZmAP1*, but not the *FUL* gene (*ZMM28*), suggesting that *ZMM28* is not a direct target at transcript level by overexpressed *ZmSOC1* at the developmental stage tested. On the other hand, both *ZmSOC1*-OX and *ZMM28* could be essential at protein levels (Abraham-Juarez et al. 2020). In *Arabidopsis*, the functions of *AP1* and *FUL* are partially overlapping. The sequence difference in *ZmAP1* and *ZMM28* may be responsible for their divergence in responding to the overexpressed *ZmSOC1* (Kater et al. 2006; McCarthy et al. 2015).

The increased grain yield in the selected maize *ZMM28*-overexpression line DP202216 was attributed to increased early plant vigor and total leaf area (Wu et al. 2019). Similarly, the overexpressed *ZmSOC1* also increased plant vigor in this study. Phenotypic variations in the nine BC_1_ transgenic lines tested have suggested that there is high potential to identify ideal transgenic lines for high grain yield. Backcrosses of the selected *ZmSOC1-*OX line to B73 are ongoing to produce new inbred B73 lines containing homozygous *ZmSOC1-*OX for field trials and commercialization of the *ZmSOC1-*OX technology. In addition, further studies are still needed to determine transgene insertion position(s), copy number, and the correlation between the *ZmSOC1* expression levels and the phenotypic changes in all 16 transgenic lines we produced. Taken together, modulating expression of MADS-box genes (e.g., the *ZmSOC1* in this report) opens a new approach to enhance crop yield or change grain quality.

## Conclusions

This is the first investigation of using a *SOC1* gene to increase the potential for high grain yield. Overexpression of *ZmSOC1* accelerated flowering, reduced plant stature, and increased/unreduced grain dry weight of the BC_1_ and BC_2_ TR plants grown under natural conditions. In addition, the grain of BC2 TR plants showed an increase in the content of starch, simple sugars, and fat. Transcriptome analysis revealed potential genes responding to *ZmSOC1*-OX. Overall, the results facilitate a better understanding of *SOC1-*regulated growth and flowering in maize. More importantly, modulating expression of *SOC1* opens a new and effectual approach to promote flowering and reduce plant height, which may have the potential to enhance crop yield and improve grain quality.

### Author contributions statement

GS conceived and supervised the study; GS, XH, and JR conducted the experiments; KW supervised the production of transgenic maize at Iowa State University Plant Transformation Facility; AT and GS designed the field trials; GS analyzed data; and GS and KW wrote the manuscript. All authors read and approved the final manuscript.

## Supplementary Information

Below is the link to the electronic supplementary material.

Supplementary file1 (PDF 7317 KB)

Supplementary file2 (PPTX 14277 KB)
