# Supplementary material for: Utilizing MIKC-type MADS-box protein SOC1 for yield potential enhancement in maize
Source: Plant Cell Rep. 2021 Jun 6;40(9):1679–93. doi: 10.1007/s00299-021-02722-4 (PMC8376726; doi:10.1007/s00299-021-02722-4)
Supplement: Supplementary file 1 — Supplementary file1 (PDF 7317 KB) [file 299_2021_2722_MOESM1_ESM.pdf]

Table S1 PCR primers

Gene	Primer name	Primer sequence (5' to 3')	Transcript ID
**PCR or RT-PCR**			
ZmSOC1	MK_F	ATGGTGCGGGGCAAGACGCAG	
	MK_R	GCCTGACCTGACCGCCACTGC	
ZmSOC1	MK_F1	GGGGTACCATGGTGCGGGGCAAGACGCAG	
	MK_R1	GCTCTAGAGCCTGACCTGACCGCCACTGC	
	35S_F	TGA CGC ACA ATC CCA CTA TC	
bar	BAR_F	ATG AGC CCA GAA CGA CGC C	
	BAR_R	TCA GAT CTC GGT GAC GG	
ZmAct	ZmAct_F	AATGCTGGGGAAGACAGCTC	
	ZmAct_R	ACCAGGCTGCATAACTGCAT	
**RT-qPCR**			
708A6_MAIZE	708A6_F	CGA CAA GGT GAA GGA GGT TAT G	DN12409_c0_g2_i1
	708A6_R	CCC TTA AGC TTG GCG ATG AA	
AB11G_ARATH	AB11G_F	TCC GAT CTC AGA GCT ACC C	DN21605_c0_g1_i4
	AB11G_R	AAG GAC TGC GTC AGG AAA C	
CHS2_MAIZE	CHS2_F	GGA GAA CCC GAG CAT GTG	DN18418_c0_g1_i17
	CHS2_R	GCG TGA TCC GCG ACT TT	
CYPB_VICFA	CYPB_F	CCG ATC TCG CTA GTT CTC AAA	DN16412_c0_g5_i1
	CYPB_R	TTA GCA CAC CAG GGC TAA C	
PMA2_SOLLC	PMA2_F	GTC GTC TCT TCA TCT CAG CAC	DN22665_c1_g1_i5
	PMA2_R	ATT GTA ACG CGA GCT TGT AGA	
RHM1_ARATH	RHM1_F	CTA CAA CAT CGG CAC CAA GA	DN36404_c0_g1_i1
	RHM1_R	GAA GTA CCT CTG GTC GTT GAA G	
SAC1_ARATH	zmActin1_F	TCA AGC AGA AGA CGG CAT AC	DN18124_c2_g1_i7
	zmActin1_R	GAG AGG AAA GGC GGA GAA AG	
URT1_FRAAN	URT1_F	CCG CTC CAC ATC GTC ATT T	DN9072_c0_g1_i3
	URT1_R	TGG GTG GAG ACG AAA GAC A	

Table S2 MADS-box genes in *A. thaliana*

Gene ID*	Annotation
>AT1G01530.1	AGL28 | AGAMOUS-like 28 | chr1:192640-193662 REVERSE LENGTH=247
>AT1G17310.1	MADS-box transcription factor family protein | chr1:5928014-5928667 REVERSE LENGTH=217
>AT1G18750.1	AGL65 | AGAMOUS-like 65 | chr1:6467266-6469640 FORWARD LENGTH=389
>AT1G22130.1	AGL104 | AGAMOUS-like 104 | chr1:7812387-7814259 REVERSE LENGTH=335
>AT1G22590.2	AGL87 | AGAMOUS-like 87 | chr1:7983511-7984002 FORWARD LENGTH=163
>AT1G24260.2	SEP3, AGL9 | K-box region and MADS-box transcription factor family protein | chr1:8593790-8595862
>AT1G26310.1	CAL, CAL1, AGL10 | K-box region and MADS-box transcription factor family protein | chr1:9100330-9
>AT1G28450.1	AGL58 | AGAMOUS-like 58 | chr1:10003966-10004523 FORWARD LENGTH=185
>AT1G28460.1	AGL59 | AGAMOUS-like 59 | chr1:10006230-10006778 FORWARD LENGTH=182
>AT1G29962.1	AGL64 | AGAMOUS-like 64 | chr1:10496730-10497287 FORWARD LENGTH=185
>AT1G31140.2	GOA | GORDITA | chr1:11118031-11119673 FORWARD LENGTH=215
>AT1G31630.1	AGL86 | AGAMOUS-like 86 | chr1:11318528-11319547 REVERSE LENGTH=339
>AT1G33070.1	MADS-box family protein | chr1:11982889-11985299 FORWARD LENGTH=165
>AT1G46408.1	AGL97 | AGAMOUS-like 97 | chr1:17232135-17232935 REVERSE LENGTH=266
>AT1G47760.1	AGL102 | AGAMOUS-like 102 | chr1:17572451-17573159 FORWARD LENGTH=184
>AT1G48150.1	MADS-box transcription factor family protein | chr1:17785397-17786368 FORWARD LENGTH=323
>AT1G50780.1	2Fe-2S ferredoxin-like superfamily protein | chr1:18821267-18823077 FORWARD LENGTH=323
>AT1G54760.1	AGL85 | AGAMOUS-like 85 | chr1:20433912-20434397 FORWARD LENGTH=161
>AT1G59810.1	AGL50 | AGAMOUS-like 50 | chr1:22008604-22009455 FORWARD LENGTH=283
>AT1G59920.1	MADS-box family protein | chr1:22059424-22059822 REVERSE LENGTH=132
>AT1G60040.1	AGL49 | AGAMOUS-like 49 | chr1:22119075-22119929 REVERSE LENGTH=284
>AT1G60920.1	AGL55 | AGAMOUS-like 55 | chr1:22429692-22430267 REVERSE LENGTH=191
>AT1G61040.1	VIP5 | plus-3 domain-containing protein | chr1:22483817-22485748 FORWARD LENGTH=643
>AT1G65330.1	PHE1, AGL37 | MADS-box transcription factor family protein | chr1:24266481-24267320 REVERSE LENGTH
>AT1G65360.1	AGL23 | AGAMOUS-like 23 | chr1:24281337-24282151 FORWARD LENGTH=226
>AT1G69120.1	AP1, AGL7 | K-box region and MADS-box transcription factor family protein | chr1:25982576-2598610
>AT1G69180.1	CRC | Plant-specific transcription factor YABBY family protein | chr1:26007734-26008940 REVERSE LE
>AT1G69540.1	AGL94 | AGAMOUS-like 94 | chr1:26145306-26147159 REVERSE LENGTH=344
>AT1G71692.1	AGL12, XAL1 | AGAMOUS-like 12 | chr1:26952903-26954939 REVERSE LENGTH=211
>AT1G72350.1	MADS-box transcription factor family protein | chr1:27239273-27239947 REVERSE LENGTH=224
>AT1G77080.4	MAF1, FLM, AGL27 | K-box region and MADS-box transcription factor family protein | chr1:28955679-
>AT1G77950.1	AGL67 | AGAMOUS-like 67 | chr1:29307029-29309667 FORWARD LENGTH=252
>AT1G77980.1	AGL66 | AGAMOUS-like 66 | chr1:29315212-29317067 REVERSE LENGTH=332
>AT2G03060.2	AGL30 | AGAMOUS-like 30 | chr2:901614-903569 FORWARD LENGTH=386
>AT2G14210.1	ANR1, AGL44 | AGAMOUS-like 44 | chr2:6018841-6023585 FORWARD LENGTH=234
>AT2G15660.1	AGL95 | AGAMOUS-like 95 | chr2:6824786-6825538 REVERSE LENGTH=250
>AT2G22630.1	AGL17 | AGAMOUS-like 17 | chr2:9618372-9621641 FORWARD LENGTH=227
>AT2G24840.1	AGL61, DIA | AGAMOUS-like 61 | chr2:10581082-10581876 FORWARD LENGTH=264
>AT2G26320.1	AGL33 | AGAMOUS-like 33 | chr2:11205389-11206287 REVERSE LENGTH=109
>AT2G28700.1	AGL46 | AGAMOUS-like 46 | chr2:12317384-12318724 REVERSE LENGTH=329

Gene ID*	Annotation
>AT2G34440.1	AGL29 | AGAMOUS-like 29 | chr2:14526950-14527468 FORWARD LENGTH=172
>AT2G40210.1	AGL48 | AGAMOUS-like 48 | chr2:16793213-16794328 REVERSE LENGTH=371
>AT2G42830.2	SHP2, AGL5 | K-box region and MADS-box transcription factor family protein | chr2:17820602-178238
>AT2G45650.1	AGL6 | AGAMOUS-like 6 | chr2:18804453-18806291 FORWARD LENGTH=252
>AT3G02310.1	SEP2, AGL4 | K-box region and MADS-box transcription factor family protein | chr3:464554-466687 R
>AT3G04100.1	AGL57 | AGAMOUS-like 57 | chr3:1075299-1075922 FORWARD LENGTH=207
>AT3G05860.1	MADS-box transcription factor family protein | chr3:1751406-1752355 REVERSE LENGTH=260
>AT3G12145.1	FLR1, FLOR1 | Leucine-rich repeat (LRR) family protein | chr3:3874764-3876075 REVERSE LENGTH=325
>AT3G12510.1	MADS-box family protein | chr3:3967127-3967648 FORWARD LENGTH=173
>AT3G18650.1	AGL103 | AGAMOUS-like 103 | chr3:6417344-6418504 REVERSE LENGTH=386
>AT3G30260.1	AGL79 | AGAMOUS-like 79 | chr3:11909119-11912880 FORWARD LENGTH=249
>AT3G54340.1	AP3, ATAP3 | K-box region and MADS-box transcription factor family protein | chr3:20119428-201210
>AT3G57230.1	AGL16 | AGAMOUS-like 16 | chr3:21177710-21180671 FORWARD LENGTH=240
>AT3G57390.1	AGL18 | AGAMOUS-like 18 | chr3:21233910-21235735 FORWARD LENGTH=256
>AT3G61120.1	AGL13 | AGAMOUS-like 13 | chr3:22618414-22620466 REVERSE LENGTH=244
>AT3G66656.1	AGL91 | AGAMOUS-like 91 | chr3:2091262-2091798 REVERSE LENGTH=178
>AT4G02235.1	AGL51 | AGAMOUS-like 51 | chr4:980955-981711 FORWARD LENGTH=196
>AT4G09960.3	STK, AGL11 | K-box region and MADS-box transcription factor family protein | chr4:6236713-6240494
>AT4G11250.1	AGL52 | AGAMOUS-like 52 | chr4:6849578-6850567 FORWARD LENGTH=329
>AT4G11880.1	AGL14 | AGAMOUS-like 14 | chr4:7143512-7147108 FORWARD LENGTH=221
>AT4G18960.1	AG | K-box region and MADS-box transcription factor family protein | chr4:10383917-10388272 FORWA
>AT4G22950.1	AGL19, GL19 | AGAMOUS-like 19 | chr4:12023946-12027421 REVERSE LENGTH=219
>AT4G24540.1	AGL24 | AGAMOUS-like 24 | chr4:12671160-12673645 REVERSE LENGTH=220
>AT4G36590.1	MADS-box transcription factor family protein | chr4:17261146-17262189 REVERSE LENGTH=248
>AT4G37940.1	AGL21 | AGAMOUS-like 21 | chr4:17835695-17838621 REVERSE LENGTH=228
>AT5G04640.1	AGL99 | AGAMOUS-like 99 | chr5:1332825-1333793 FORWARD LENGTH=322
>AT5G06500.1	AGL96 | AGAMOUS-like 96 | chr5:1982444-1983172 FORWARD LENGTH=242
>AT5G10140.1	FLC, FLF, AGL25 | K-box region and MADS-box transcription factor family protein | chr5:3173724-31
>AT5G13790.1	AGL15 | AGAMOUS-like 15 | chr5:4449128-4450802 REVERSE LENGTH=268
>AT5G15800.2	SEP1, AGL2 | K-box region and MADS-box transcription factor family protein | chr5:5151594-5153767
>AT5G18000.1	VDD | VERDANDI | chr5:5960071-5961473 REVERSE LENGTH=307
>AT5G20240.1	PI | K-box region and MADS-box transcription factor family protein | chr5:6829203-6831208 FORWARD
>AT5G23150.1	HUA2 | Tudor/PWWP/MBT domain-containing protein | chr5:7786173-7792080 FORWARD LENGTH=1392
>AT5G23260.2	TT16, ABS, AGL32 | K-box region and MADS-box transcription factor family protein | chr5:7836442-7
>AT5G26580.1	AGL34 | AGAMOUS-like-34 | chr5:9393065-9394102 REVERSE LENGTH=345
>AT5G26630.1	MADS-box transcription factor family protein | chr5:9350815-9351471 FORWARD LENGTH=218
>AT5G26650.1	AGL36 | AGAMOUS-like 36 | chr5:9343785-9344885 FORWARD LENGTH=366
>AT5G26950.1	AGL93 | AGAMOUS-like 93 | chr5:9483251-9484120 REVERSE LENGTH=289
>AT5G27050.1	AGL101 | AGAMOUS-like 101 | chr5:9520276-9520638 FORWARD LENGTH=120
>AT5G27070.1	AGL53 | AGAMOUS-like 53 | chr5:9527741-9528604 FORWARD LENGTH=287
>AT5G27090.1	AGL54 | AGAMOUS-like 54 | chr5:9531845-9532408 FORWARD LENGTH=187

Gene ID*	Annotation
>AT5G27130.1	AGL39 | AGAMOUS-like 39 | chr5:9546633-9547553 FORWARD LENGTH=306
>AT5G27580.1	AGL89 | AGAMOUS-like 89 | chr5:9736651-9737322 FORWARD LENGTH=223
>AT5G27810.1	MADS-box transcription factor family protein | chr5:9855827-9856186 FORWARD LENGTH=119
>AT5G27944.1	MADS-box transcription factor family protein | chr5:9975918-9976592 REVERSE LENGTH=224
>AT5G27960.1	AGL90 | AGAMOUS-like 90 | chr5:9991685-9992770 REVERSE LENGTH=320
>AT5G35120.1	MADS-box family protein | chr5:13386852-13387175 REVERSE LENGTH=107
>AT5G37415.1	AGL105 | AGAMOUS-like 105 | chr5:14839047-14840301 REVERSE LENGTH=316
>AT5G38620.1	MADS-box transcription factor family protein | chr5:15463858-15464907 REVERSE LENGTH=349
>AT5G38740.1	AGL77 | AGAMOUS-like 77 | chr5:15513025-15514305 REVERSE LENGTH=426
>AT5G39750.1	EMB3008, AGL81 | AGAMOUS-like 81 | chr5:15906875-15907942 FORWARD LENGTH=355
>AT5G39810.1	AGL98 | AGAMOUS-like 98 | chr5:15937278-15938344 REVERSE LENGTH=329
>AT5G40070.1	MADS-box family protein | chr5:16039179-16039747 FORWARD LENGTH=157
>AT5G40120.1	AGL76 | AGAMOUS-like 76 | chr5:16051879-16053036 FORWARD LENGTH=385
>AT5G40220.1	AGL43 | AGAMOUS-like 43 | chr5:16078390-16079364 REVERSE LENGTH=324
>AT5G41200.1	AGL75 | AGAMOUS-like 75 | chr5:16490544-16491536 FORWARD LENGTH=330
>AT5G41410.1	BEL1 | POX (plant homeobox) family protein | chr5:16580424-16583770 FORWARD LENGTH=611
>AT5G46320.1	MADS-box family protein | chr5:18788246-18788888 REVERSE LENGTH=118
>AT5G48670.1	FEM111, AGL80 | AGAMOUS-like 80 | chr5:19738825-19739790 REVERSE LENGTH=321
>AT5G49420.1	MADS-box transcription factor family protein | chr5:20035166-20036170 REVERSE LENGTH=334
>AT5G49490.1	AGL83 | AGAMOUS-like 83 | chr5:20075328-20076185 FORWARD LENGTH=285
>AT5G51860.1	K-box region and MADS-box transcription factor family protein | chr5:21081844-21084126 REVERSE
>AT5G51870.3	AGL71 | AGAMOUS-like 71 | chr5:21085635-21087923 REVERSE LENGTH=219
>AT5G55690.1	MADS-box transcription factor family protein | chr5:22548790-22549623 REVERSE LENGTH=277
>AT5G58890.1	AGL82 | AGAMOUS-like 82 | chr5:23780832-23781716 FORWARD LENGTH=294
>AT5G60440.1	AGL62 | AGAMOUS-like 62 | chr5:24306329-24307520 FORWARD LENGTH=299
>AT5G60910.1	AGL8, FUL | AGAMOUS-like 8 | chr5:24502736-24506013 REVERSE LENGTH=242
>AT5G62165.1	AGL42 | AGAMOUS-like 42 | chr5:24965075-24968437 FORWARD LENGTH=210
>AT5G65050.3	AGL31, MAF2 | AGAMOUS-like 31 | chr5:25982415-25986114 FORWARD LENGTH=196
>AT5G65060.1	MAF3, FCL3, AGL70 | K-box region and MADS-box transcription factor family protein | chr5:25987527
>AT5G65070.3	MAF4 | K-box region and MADS-box transcription factor family protein | chr5:25992310-25995930 FOR
>AT5G65080.1	MAF5, AGL68 | K-box region and MADS-box transcription factor family protein | chr5:25997650-26002
>AT5G65330.1	AGL78 | AGAMOUS-like 78 | chr5:26110326-26111351 FORWARD LENGTH=341

*------------------

The following sequences were not found:

AT2G11990.1

AT2G12000.1

AT3G31902.1

AT3G32313.1

Table S3 Original data used for statistic analyses of phenotypic changes

Corn_ID	Corn _ID2	Year	NT or TR	Corn_ID3	Corn_ID4	germination time (days after sowing)	Tassel appearance time (days after sowing)	Ear_ DW (g)	Grain_ DW (g)	Height P (cm)	Time Silk (days)	Leaf No	Height Soil to Corn (cm)	Plant_ DW (g)	ear_time (days)
C1-7	C1	rep1	NT	C1NT	C1NTrep1	5	58	0	0	171	9	12	73	141	67
C2-10	C2	rep1	NT	C2NT	C2NTrep1	4	57	284	229	160	10	11	55	215	67
C2-9	C2	rep1	NT	C2NT	C2NTrep1	4	58	285	244	153	9	12	61	149	67
C5-3	C5	rep1	NT	C5NT	C5NTrep1	4	58	237	197	157	11	10	57	125	69
C7-1	C7	rep1	NT	C7NT	C7NTrep1	4	58	199	148	154	10	10	55	165	68
C7-3	C7	rep1	NT	C7NT	C7NTrep1	4	61	205	167	160	6	12	65	145	67
C7-9	C7	rep1	NT	C7NT	C7NTrep1	6	60	335	290	178	4	12	66	188	64
C9-3	C9	rep1	NT	C9NT	C9NTrep1	4	59	215	153	157	10	11	56	173	69
C9-6	C9	rep1	NT	C9NT	C9NTrep1	4	61	269	213	171	8	11	61	160	69
C16-10	C16	rep1	TR	C16TR	C16TRrep1	4	56	302	239	136	8	11	48	143	64
C16-2	C16	rep1	TR	C16TR	C16TRrep1	4	56	302	246	146	3	12	55	105	59
C2-5	C2	rep1	TR	C2TR	C2TRrep1	4	60	286	254	145	9	10	53	126	69
C2-7	C2	rep1	TR	C2TR	C2TRrep1	4	57	243	201	134	10	10	43	124	67
C3-2	C3	rep1	TR	C3TR	C3TRrep1	8	63	367	315	176	6	13	62	241	69
C3-3	C3	rep1	TR	C3TR	C3TRrep1	4	58	284	218	149	6	10	61	147	64
C5-1	C5	rep1	TR	C5TR	C5TRrep1	4	54	197	143	94	4	9	35	67	58
C5-10	C5	rep1	TR	C5TR	C5TRrep1	5	61	251	206	145	7	10	54	182	68
C5-2	C5	rep1	TR	C5TR	C5TRrep1	4	54	220	177	126	2	11	58	70	56
C5-4	C5	rep1	TR	C5TR	C5TRrep1	4	54	215	173	114	5	9	45	68	59
C5-5	C5	rep1	TR	C5TR	C5TRrep1	4	60	274	238	171	7	11	79	133	67
C5-6	C5	rep1	TR	C5TR	C5TRrep1	5	63	258	219	155	5	11	60	127	68
C5-7	C5	rep1	TR	C5TR	C5TRrep1	4	54	194	157	119	0	9	49	72	54
C6-2	C6	rep1	TR	C6TR	C6TRrep1	4	54	319	257	124	4	10	51	67	58
C6-5	C6	rep1	TR	C6TR	C6TRrep1	5	56	278	228	120	3	10	37	72	59
C7-10	C7	rep1	TR	C7TR	C7TRrep1	5	55	214	179	109	3	9	41	63	58
C7-6	C7	rep1	TR	C7TR	C7TRrep1	4	54	226	182	114	3	11	39	73	57
C7-7	C7	rep1	TR	C7TR	C7TRrep1	4	56	289	248	118	1	11	50	78	57
C9-1	C9	rep1	TR	C9TR	C9TRrep1	4	55	227	178	127	3	10	46	93	58
C9-2	C9	rep1	TR	C9TR	C9TRrep1	4	54	226	176	138	3	10	56	72	57
C9-5	C9	rep1	TR	C9TR	C9TRrep1	4	56	211	167	123	3	9	48	82	59
C9-7	C9	rep1	TR	C9TR	C9TRrep1	4	57	247	198	145	2	9	57	67	59
C9-9	C9	rep1	TR	C9TR	C9TRrep1	6	59	252	205	139	3	10	49	77	62
C1-17	C1	rep2	NT	C1NT	C1NTrep2	4	57	224	194	190	7	13	73	n/a	64
C11-16	C11	rep2	NT	C11NT	C11NTrep2	4	56	276	219	185	12	15	65	n/a	68
C11-20	C11	rep2	NT	C11NT	C11NTrep2	6	56	221	196	207	9	12	60	n/a	65
C16-14	C16	rep2	NT	C16NT	C16NTrep2	4	57	209	193	187	4	12	65	n/a	61
C2-11	C2	rep2	NT	C2NT	C2NTrep2	5	57	196	163	179	8	11	50	n/a	65
C2-14	C2	rep2	NT	C2NT	C2NTrep2	5	60	215	194	185	7	11	58	n/a	67
C2-18	C2	rep2	NT	C2NT	C2NTrep2	4	52	222	204	181	8	11	45	n/a	60
C3-13	C3	rep2	NT	C3NT	C3NTrep2	4	54	242	218	186	6	11	66	n/a	60
C3-14	C3	rep2	NT	C3NT	C3NTrep2	4	56	209	185	179	5	12	48	n/a	61
C3-16	C3	rep2	NT	C3NT	C3NTrep2	4	58	139	111	183	14	10	51	n/a	72
C3-17	C3	rep2	NT	C3NT	C3NTrep2	4	54	208	185	197	9	11	65	n/a	63
C5-14	C5	rep2	NT	C5NT	C5NTrep2	4	52	212	189	186	7	11	60	n/a	59
C5-16	C5	rep2	NT	C5NT	C5NTrep2	4	55	214	193	179	3	11	63	n/a	58
C5-17	C5	rep2	NT	C5NT	C5NTrep2	4	53	218	205	173	5	11	68	n/a	58
C6-18	C6	rep2	NT	C6NT	C6NTrep2	5	54	218	197	182	4	12	55	n/a	58
C7-12	C7	rep2	NT	C7NT	C7NTrep2	4	52	143	127	180	4	10	41	n/a	56
C7-14	C7	rep2	NT	C7NT	C7NTrep2	4	52	183	161	189	3	10	50	n/a	55
C7-19	C7	rep2	NT	C7NT	C7NTrep2	4	56	70	39	198	9	13	83	n/a	65
C7-20	C7	rep2	NT	C7NT	C7NTrep2	4	51	144	128	197	3	9	43	n/a	54
C8-19	C8	rep2	NT	C8NT	C8NTrep2	4	59	234	213	191	6	13	84	n/a	65

Corn_ID	Corn _ID2	Year	NT or TR	Corn_ID3	Corn_ID4	germination time (days after sowing)	Tassel appearance time (days after sowing)	Ear_ DW (g)	Grain_ DW (g)	Height P (cm)	Time Silk (days)	Leaf No	Height Soil to Corn (cm)	Plant_ DW (g)	ear_time (days)
C9-13	C9	rep2	NT	C9NT	C9NTrep2	4	52	62	54	187	4	7	30	n/a	56
C9-15	C9	rep2	NT	C9NT	C9NTrep2	4	52	199	175	180	5	11	53	n/a	57
C9-17	C9	rep2	NT	C9NT	C9NTrep2	4	52	144	138	198	8	9	53	n/a	60
C9-18	C9	rep2	NT	C9NT	C9NTrep2	5	52	167	153	205	8	10	34	n/a	60
C9-19	C9	rep2	NT	C9NT	C9NTrep2	4	52	149	135	180	8	10	40	n/a	60
C1-13	C1	rep2	TR	C1TR	C1TRrep2	5	61	179	162	157	3	15	73	n/a	64
C1-16	C1	rep2	TR	C1TR	C1TRrep2	4	56	290	258	165	5	13	66	n/a	61
C16-13	C16	rep2	TR	C16TR	C16TRrep2	3	56	224	202	126	6	11	48	n/a	62
C2-15	C2	rep2	TR	C2TR	C2TRrep2	4	58	233	208	157	5	13	55	n/a	63
C2-16	C2	rep2	TR	C2TR	C2TRrep2	4	56	269	244	178	8	13	55	n/a	64
C2-19	C2	rep2	TR	C2TR	C2TRrep2	4	55	244	221	151	7	11	58	n/a	62
C3-19	C3	rep2	TR	C3TR	C3TRrep2	4	52	188	171	184	4	11	62	n/a	56
C5-11	C5	rep2	TR	C5TR	C5TRrep2	4	56	286	261	157	4	12	68	n/a	60
C5-12	C5	rep2	TR	C5TR	C5TRrep2	4	55	242	219	171	4	13	79	n/a	59
C5-15	C5	rep2	TR	C5TR	C5TRrep2	5	53	165	151	175	3	11	48	n/a	56
C5-19	C5	rep2	TR	C5TR	C5TRrep2	4	55	233	219	150	5	12	79	n/a	60
C5-20	C5	rep2	TR	C5TR	C5TRrep2	5	59	235	215	143	4	12	75	n/a	63
C6-14	C6	rep2	TR	C6TR	C6TRrep2	3	56	208	186	140	14	13	53	n/a	70
C6-15	C6	rep2	TR	C6TR	C6TRrep2	4	52	229	207	176	7	10	49	n/a	59
C6-19	C6	rep2	TR	C6TR	C6TRrep2	4	54	322	296	178	7	13	74	n/a	61
C7-11	C7	rep2	TR	C7TR	C7TRrep2	4	55	237	213	165	5	13	77	n/a	60
C7-16	C7	rep2	TR	C7TR	C7TRrep2	4	56	249	222	155	6	13	78	n/a	62
C7-17	C7	rep2	TR	C7TR	C7TRrep2	4	51	156	141	180	3	11	50	n/a	54
C7-18	C7	rep2	TR	C7TR	C7TRrep2	4	56	225	193	160	6	12	76	n/a	62
C8-11	C8	rep2	TR	C8TR	C8TRrep2	7	59	288	257	164	7	13	84	n/a	66
C9-12	C9	rep2	TR	C9TR	C9TRrep2	5	57	147	130	170	11	12	59	n/a	68
C9-16	C9	rep2	TR	C9TR	C9TRrep2	4	55	234	214	174	8	11	43	n/a	63
C9-20	C9	rep2	TR	C9TR	C9TRrep2	4	60	302	262	158	8	12	54	n/a	68
C1-2	C1	rep3	NT	C1NT	C1NTrep3	8	80	11	1	110	6	n/a	n/a	89	86
C11-4	C11	rep3	NT	C11NT	C11NTrep3	9	67	87	72	115	5	n/a	n/a	144	72
C16-1	C16	rep3	NT	C16NT	C16NTrep3	5	74	121	97	123	4	n/a	n/a	187	78
C16-2	C16	rep3	NT	C16NT	C16NTrep3	5	71	101	84	140	4	n/a	n/a	162	75
C2-3	C2	rep3	NT	C2NT	C2NTrep3	5	75	99	78	126	11	n/a	n/a	200	86
C2-9	C2	rep3	NT	C2NT	C2NTrep3	11	81	72	53	130	10	n/a	n/a	183	91
C3-1	C3	rep3	NT	C3NT	C3NTrep3	8	71	53	41	127	15	n/a	n/a	156	86
c5-1	c5	rep3	NT	c5NT	c5NTrep3	6	71	126	105	118	6	n/a	n/a	221	77
c5-3	c5	rep3	NT	c5NT	c5NTrep3	6	76	101	85	140	7	n/a	n/a	174	83
C5-5	C5	rep3	NT	C5NT	C5NTrep3	6	73	53	35	113	16	n/a	n/a	141	89
C6-3	C6	rep3	NT	C6NT	C6NTrep3	9	74	80	66	109	12	n/a	n/a	186	86
C7-1	C7	rep3	NT	C7NT	C7NTrep3	5	72	10	1	125	19	n/a	n/a	106	91
C7-11	C7	rep3	NT	C7NT	C7NTrep3	6	74	0	0	122	8	n/a	n/a	91	82
C7-14	C7	rep3	NT	C7NT	C7NTrep3	8	74	11	0	119	22	n/a	n/a	95	96
C7-3	C7	rep3	NT	C7NT	C7NTrep3	5	72	52	35	119	14	n/a	n/a	156	86
C7-4	C7	rep3	NT	C7NT	C7NTrep3	5	78	20	6	150	10	n/a	n/a	96	88
C7-6	C7	rep3	NT	C7NT	C7NTrep3	5	78	8	1	121	n/a	n/a	n/a	110	78
C7-7	C7	rep3	NT	C7NT	C7NTrep3	6	72	90	80	146	8	n/a	n/a	161	80
C7-9	C7	rep3	NT	C7NT	C7NTrep3	5	74	16	6	121	12	n/a	n/a	94	86
C9-1	C9	rep3	NT	C9NT	C9NTrep3	5	75	78	64	119	7	n/a	n/a	143	82
C9-10	C9	rep3	NT	C9NT	C9NTrep3	7	73	140	123	129	6	n/a	n/a	202	79
C9-5	C9	rep3	NT	C9NT	C9NTrep3	5	71	153	132	138	7	n/a	n/a	223	78
C1-3	C1	rep3	TR	C1TR	C1TRrep3	6	72	64	53	130	6	n/a	n/a	133	78
C11-3	C11	rep3	TR	C11TR	C11TRrep3	9	72	117	96	130	8	n/a	n/a	205	80
C16-5	C16	rep3	TR	C16TR	C16TRrep3	8	67	113	97	120	5	n/a	n/a	179	72

Corn_ID	Corn _ID2	Year	NT or TR	Corn_ID3	Corn_ID4	germination time (days after sowing)	Tassel appearance time (days after sowing)	Ear_ DW (g)	Grain_ DW (g)	Height P (cm)	Time Silk (days)	Leaf No	Height Soil to Corn (cm)	Plant_ DW (g)	ear_time (days)
C16-6	C16	rep3	TR	C16TR	C16TRrep3	8	66	85	74	119	7	n/a	n/a	125	73
C2-1	C2	rep3	TR	C2TR	C2TRrep3	5	67	103	85	132	10	n/a	n/a	185	77
C2-2	C2	rep3	TR	C2TR	C2TRrep3	5	72	78	67	130	9	n/a	n/a	127	81
C2-6	C2	rep3	TR	C2TR	C2TRrep3	5	72	116	92	132	11	n/a	n/a	227	83
C2-8	C2	rep3	TR	C2TR	C2TRrep3	5	71	120	98	121	11	n/a	n/a	240	82
C3-4	C3	rep3	TR	C3TR	C3TRrep3	9	75	11	1	120	16	n/a	n/a	88	91
C3-5	C3	rep3	TR	C3TR	C3TRrep3	9	68	86	75	117	6	n/a	n/a	124	74
C5-11	C5	rep3	TR	C5TR	C5TRrep3	11	82	24	12	118	7	n/a	n/a	121	89
c5-2	c5	rep3	TR	c5TR	c5TRrep3	6	74	7	1	118	16	n/a	n/a	97	90
c5-4	c5	rep3	TR	c5TR	c5TRrep3	7	72	100	86	123	5	n/a	n/a	176	77
C5-7	C5	rep3	TR	C5TR	C5TRrep3	6	74	23	14	123	13	n/a	n/a	161	87
C5-8	C5	rep3	TR	C5TR	C5TRrep3	8	78	9	1	140	12	n/a	n/a	121	90
C5-9	C5	rep3	TR	C5TR	C5TRrep3	8	74	43	30	118	6	n/a	n/a	166	80
C6-1	C6	rep3	TR	C6TR	C6TRrep3	7	62	59	45	108	7	n/a	n/a	105	69
C6-2	C6	rep3	TR	C6TR	C6TRrep3	7	68	128	105	110	6	n/a	n/a	255	74
C6-4	C6	rep3	TR	C6TR	C6TRrep3	6	68	90	70	105	6	n/a	n/a	133	74
C7-10	C7	rep3	TR	C7TR	C7TRrep3	6	65	52	37	104	6	n/a	n/a	98	71
C7-12	C7	rep3	TR	C7TR	C7TRrep3	7	63	76	65	112	4	n/a	n/a	119	67
C7-13	C7	rep3	TR	C7TR	C7TRrep3	8	63	65	52	99	4	n/a	n/a	92	67
C7-2	C7	rep3	TR	C7TR	C7TRrep3	5	71	92	77	108	-4	n/a	n/a	147	67
C7-5	C7	rep3	TR	C7TR	C7TRrep3	5	60	61	50	98	8	n/a	n/a	87	68
C7-8	C7	rep3	TR	C7TR	C7TRrep3	5	60	80	69	110	7	n/a	n/a	95	67
C9-11	C9	rep3	TR	C9TR	C9TRrep3	7	74	88	73	113	6	n/a	n/a	154	80
C9-2	C9	rep3	TR	C9TR	C9TRrep3	7	62	83	11	118	5	n/a	n/a	132	67
C9-3	C9	rep3	TR	C9TR	C9TRrep3	5	65	88	77	119	5	n/a	n/a	124	70
C9-4	C9	rep3	TR	C9TR	C9TRrep3	6	62	93	79	117	5	n/a	n/a	194	67
C9-6	C9	rep3	TR	C9TR	C9TRrep3	8	65	104	89	101	5	n/a	n/a	154	70
C9-7	C9	rep3	TR	C9TR	C9TRrep3	6	66	83	71	116	6	n/a	n/a	135	72
C9-8	C9	rep3	TR	C9TR	C9TRrep3	6	60	90	79	118	6	n/a	n/a	145	66
C9-9	C9	rep3	TR	C9TR	C9TRrep3	6	63	72	64	113	6	n/a	n/a	111	69

Table S4. Summary of transcriptome comparison

	Total # DETs identified	# DETs annotated	# unique genes matched
**c7 TR vs c7 NT**	473	322	249
**c9 TR vs c7 NT**	2576	1692	1136
**c7 TR vs c9 TR**	1127	676	485

Table S5 Annotated differentially expressed transcripts identified in the comparisons of c7TR vs. c7NT, c9TR vs. c7NT, and c7TR vs. c9TR in maize leaves during the fast growing stage of the plants before flowering. Log_2FC: Log_2Fold Change. Log_2CPM: Log_2Count Per Million Reads. FDR: False Discovery Rate. Highlighted lane represents ZmSOC1 transgene transcript.

DET	Subject id	c9TR vs c7NT				c7TR vs c7NT				c7TR vs c9TR			
		Log_2	Log_2CPM	PValue	19FDR	Log_2	Log_2CPM	PValue	19FDR	Log_2	Log_2CPM	PValue	19FDR
TRINITY_DN21650_c1_g1_i11	NSE4A_ARATH	1.85	1.75	1.5E-03	4.1E-02	-8.13	-0.42	1.2E-06	3.6E-04	-10.12	1.40	6.2E-16	2.3E-13
TRINITY_DN11979_c0_g1_i1	CP26B_ARATH	-1.43	3.13	1.8E-04	7.5E-03	-11.29	2.60	8.8E-30	5.2E-26	-9.98	1.27	2.2E-16	8.6E-14
TRINITY_DN18418_c0_g1_i17	CHS2_MAIZE	-1.69	6.09	2.3E-06	1.6E-04	-2.68	5.83	6.8E-11	4.9E-08	-1.01	4.59	1.6E-04	1.2E-02
TRINITY_DN19487_c0_g1_i1	RA213_ARATH	1.97	3.38	2.3E-06	1.6E-04	3.01	4.16	6.8E-11	4.9E-08	1.01	4.63	5.8E-05	4.7E-03
TRINITY_DN19487_c0_g1_i3	RA213_ARATH	-10.33	1.64	3.7E-09	4.3E-07	-10.23	1.56	1.6E-08	7.1E-06	#N/A	#N/A	#N/A	#N/A
TRINITY_DN18665_c2_g1_i12	H4_SOYBN	-8.18	-0.40	1.8E-07	1.7E-05	-8.08	-0.47	1.5E-06	4.7E-04	#N/A	#N/A	#N/A	#N/A
TRINITY_DN16412_c0_g5_i1	CYPB_VICFA	-8.06	-0.53	8.9E-07	6.8E-05	-5.37	-0.56	1.6E-04	2.5E-02	#N/A	#N/A	#N/A	#N/A
TRINITY_DN12322_c0_g1_i1	CLH2_ARATH	-7.71	3.36	7.9E-06	4.9E-04	-7.62	3.27	4.8E-05	9.2E-03	#N/A	#N/A	#N/A	#N/A
TRINITY_DN14065_c0_g1_i3	E2FE_ARATH	-6.19	-0.55	1.2E-04	5.4E-03	-7.90	-0.63	7.4E-05	1.3E-02	#N/A	#N/A	#N/A	#N/A
TRINITY_DN22198_c1_g4_i2	DDPS2_ARATH	-6.17	5.42	7.1E-04	2.4E-02	-7.26	5.31	2.8E-04	4.1E-02	#N/A	#N/A	#N/A	#N/A
TRINITY_DN10883_c0_g1_i1	LECH_HORVU	-5.82	3.61	8.2E-06	5.0E-04	-5.00	3.54	3.7E-04	5.1E-02	#N/A	#N/A	#N/A	#N/A
TRINITY_DN7619_c0_g1_i1	SBT18_ARATH	-5.23	3.09	4.3E-04	1.6E-02	-6.67	2.97	6.4E-05	1.2E-02	#N/A	#N/A	#N/A	#N/A
TRINITY_DN18903_c0_g1_i1	TRPA_MAIZE	-5.05	3.43	2.1E-07	1.9E-05	-3.38	3.43	1.3E-04	2.1E-02	#N/A	#N/A	#N/A	#N/A
TRINITY_DN18903_c0_g1_i3	TRPA_MAIZE	-4.52	5.47	2.5E-09	3.0E-07	-3.06	5.48	7.5E-06	1.8E-03	#N/A	#N/A	#N/A	#N/A
TRINITY_DN22035_c0_g2_i1	AAMT3_MAIZE	-3.93	3.59	1.1E-10	1.7E-08	-2.98	3.58	2.1E-06	6.1E-04	#N/A	#N/A	#N/A	#N/A
TRINITY_DN18418_c0_g1_i14	CHS2_MAIZE	-2.65	5.64	5.5E-13	1.1E-10	-2.57	5.57	4.6E-09	2.4E-06	#N/A	#N/A	#N/A	#N/A
TRINITY_DN20524_c0_g6_i2	CB2D_SOLLC	-2.58	4.16	5.6E-16	1.5E-13	-2.99	4.02	2.0E-16	3.2E-13	#N/A	#N/A	#N/A	#N/A
TRINITY_DN16963_c0_g1_i3	DFRA_MAIZE	-2.49	3.87	1.7E-13	3.5E-11	-2.77	3.75	6.5E-08	2.6E-05	#N/A	#N/A	#N/A	#N/A
TRINITY_DN21662_c2_g3_i1	CB25_PETSP	-2.30	3.25	3.5E-10	4.8E-08	-1.88	3.25	1.2E-04	2.0E-02	#N/A	#N/A	#N/A	#N/A
TRINITY_DN18917_c0_g2_i5	CER3_ARATH	-2.25	4.35	9.4E-11	1.4E-08	-1.62	4.39	1.1E-04	1.8E-02	#N/A	#N/A	#N/A	#N/A
TRINITY_DN36404_c0_g1_i1	RHM1_ARATH	-2.20	7.35	5.3E-10	6.9E-08	-2.64	7.19	1.9E-10	1.3E-07	#N/A	#N/A	#N/A	#N/A
TRINITY_DN19812_c1_g1_i3	CB48_MAIZE	-2.14	6.91	1.2E-12	2.3E-10	-1.85	6.88	3.3E-05	6.8E-03	#N/A	#N/A	#N/A	#N/A
TRINITY_DN15374_c0_g4_i2	CPNB4_ARATH	-2.07	3.32	1.8E-11	2.9E-09	-1.77	3.30	2.3E-04	3.4E-02	#N/A	#N/A	#N/A	#N/A
TRINITY_DN17315_c2_g4_i1	POLX_TOBAC	-2.00	4.00	3.5E-10	4.8E-08	-2.14	3.89	4.6E-09	2.4E-06	#N/A	#N/A	#N/A	#N/A
TRINITY_DN15969_c0_g3_i4	DTX46_ARATH	-1.93	3.02	1.4E-07	1.3E-05	-1.86	2.95	7.0E-06	1.8E-03	#N/A	#N/A	#N/A	#N/A
TRINITY_DN14692_c0_g1_i3	YSL14_ORYSJ	-1.93	2.61	1.6E-05	9.4E-04	-2.54	2.42	2.6E-06	7.3E-04	#N/A	#N/A	#N/A	#N/A
TRINITY_DN15370_c0_g1_i4	PP111_ARATH	-1.87	3.79	1.4E-12	2.7E-10	-1.55	3.78	6.4E-05	1.2E-02	#N/A	#N/A	#N/A	#N/A
TRINITY_DN17073_c0_g8_i2	KCS6_ARATH	-1.87	1.91	2.1E-05	1.2E-03	-1.93	1.81	2.6E-04	3.7E-02	#N/A	#N/A	#N/A	#N/A
TRINITY_DN12409_c0_g2_i1	708A6_MAIZE	-1.86	5.92	4.1E-12	7.1E-10	-2.13	5.78	2.7E-10	1.8E-07	#N/A	#N/A	#N/A	#N/A
TRINITY_DN15773_c2_g1_i4	CB22_MAIZE	-1.85	4.79	2.6E-09	3.1E-07	-2.02	4.67	1.9E-08	8.7E-06	#N/A	#N/A	#N/A	#N/A
TRINITY_DN18200_c0_g1_i2	CB2D_SOLLC	-1.85	6.92	4.1E-09	4.8E-07	-1.82	6.84	1.1E-08	5.0E-06	#N/A	#N/A	#N/A	#N/A
TRINITY_DN18116_c0_g1_i18	RBP45_NICPL	-1.84	1.54	5.3E-04	1.9E-02	-5.04	1.15	3.1E-10	2.0E-07	#N/A	#N/A	#N/A	#N/A
TRINITY_DN18418_c0_g1_i8	CHS2_MAIZE	-1.76	4.67	1.6E-08	1.8E-06	-2.34	4.47	1.3E-09	7.5E-07	#N/A	#N/A	#N/A	#N/A
TRINITY_DN22375_c0_g4_i1	PER45_ARATH	-1.62	4.44	2.0E-03	5.2E-02	-2.19	4.23	1.6E-04	2.5E-02	#N/A	#N/A	#N/A	#N/A
TRINITY_DN21244_c3_g9_i1	CHS2_MAIZE	-1.59	2.60	1.2E-05	7.3E-04	-2.95	2.28	3.3E-05	6.7E-03	#N/A	#N/A	#N/A	#N/A
TRINITY_DN20803_c2_g1_i7	HSP7R_ARATH	-1.56	4.95	7.8E-04	2.6E-02	-1.91	4.78	1.3E-04	2.1E-02	#N/A	#N/A	#N/A	#N/A
TRINITY_DN16007_c1_g1_i18	UFAA1_MYCTU	-1.51	2.01	2.1E-04	8.7E-03	-1.95	1.82	8.9E-05	1.6E-02	#N/A	#N/A	#N/A	#N/A
TRINITY_DN16963_c0_g1_i4	DFRA_MAIZE	-1.47	2.19	1.8E-04	7.5E-03	-2.54	1.89	2.6E-06	7.3E-04	#N/A	#N/A	#N/A	#N/A
TRINITY_DN16626_c0_g3_i2	MAD50_ORYSJ	-1.41	2.29	1.4E-03	4.1E-02	-2.03	2.06	1.3E-04	2.1E-02	#N/A	#N/A	#N/A	#N/A
TRINITY_DN16626_c0_g2_i8	MAD56_ORYSI	-1.36	6.03	3.5E-06	2.3E-04	-1.42	5.92	1.8E-04	2.7E-02	#N/A	#N/A	#N/A	#N/A
TRINITY_DN14795_c0_g1_i3	LECH_HORVU	-1.30	3.92	1.0E-05	6.2E-04	-1.50	3.78	2.4E-05	5.2E-03	#N/A	#N/A	#N/A	#N/A
TRINITY_DN9072_c0_g1_i3	URT1_FRAAN	-1.25	6.81	3.2E-05	1.7E-03	-1.75	6.59	4.0E-07	1.4E-04	#N/A	#N/A	#N/A	#N/A
TRINITY_DN19707_c2_g1_i4	HPSE3_ARATH	-1.20	3.75	2.7E-04	1.1E-02	-1.42	3.61	3.8E-04	5.3E-02	#N/A	#N/A	#N/A	#N/A
TRINITY_DN20732_c0_g1_i21	NIP21_MAIZE	-1.14	3.41	7.4E-04	2.4E-02	-1.55	3.20	7.1E-05	1.3E-02	#N/A	#N/A	#N/A	#N/A
TRINITY_DN22772_c1_g1_i12	C3H22_ORYSJ	-0.98	3.34	3.1E-04	1.2E-02	-1.37	3.14	3.5E-04	5.0E-02	#N/A	#N/A	#N/A	#N/A
TRINITY_DN14795_c0_g1_i2	LECH_HORVU	-0.98	4.57	1.5E-04	6.8E-03	-1.33	4.37	3.7E-05	7.3E-03	#N/A	#N/A	#N/A	#N/A
TRINITY_DN18341_c0_g1_i1	RHM1_ARATH	-0.96	5.31	1.4E-05	8.0E-04	-1.47	5.07	4.0E-05	7.9E-03	#N/A	#N/A	#N/A	#N/A
TRINITY_DN16639_c1_g1_i1	RHM1_ARATH	-0.96	4.38	7.8E-05	3.7E-03	-1.32	4.18	3.0E-05	6.2E-03	#N/A	#N/A	#N/A	#N/A
TRINITY_DN15312_c1_g3_i4	BBX24_ARATH	0.98	4.46	1.3E-05	7.9E-04	1.91	5.05	1.0E-04	1.7E-02	#N/A	#N/A	#N/A	#N/A
TRINITY_DN15065_c0_g3_i3	AKR1_SOYBN	1.27	3.68	3.6E-06	2.4E-04	1.27	3.58	3.7E-04	5.1E-02	#N/A	#N/A	#N/A	#N/A
TRINITY_DN13123_c0_g1_i4	EXGA_EMENI	1.36	4.05	2.4E-07	2.1E-05	1.35	3.95	8.9E-05	1.6E-02	#N/A	#N/A	#N/A	#N/A
TRINITY_DN19326_c1_g1_i1	C78A6_ARATH	1.36	5.25	1.5E-07	1.4E-05	1.81	5.50	6.4E-05	1.2E-02	#N/A	#N/A	#N/A	#N/A
TRINITY_DN15375_c0_g3_i12	UBC25_ARATH	1.37	4.12	1.9E-05	1.1E-03	1.59	4.20	3.7E-05	7.4E-03	#N/A	#N/A	#N/A	#N/A
TRINITY_DN17659_c0_g1_i9	APT2_ARATH	1.38	4.23	8.3E-08	8.0E-06	1.58	4.28	1.0E-05	2.5E-03	#N/A	#N/A	#N/A	#N/A
TRINITY_DN19326_c1_g1_i2	C78A6_ARATH	1.43	6.61	1.7E-07	1.5E-05	1.94	6.91	5.4E-05	1.0E-02	#N/A	#N/A	#N/A	#N/A
TRINITY_DN15255_c0_g1_i14	MLO1_ARATH	1.52	3.67	1.2E-03	3.7E-02	2.07	4.01	1.7E-05	3.7E-03	#N/A	#N/A	#N/A	#N/A
TRINITY_DN16232_c1_g2_i3	ODBA2_ARATH	1.78	3.10	1.4E-05	8.3E-04	1.95	3.15	1.4E-05	3.3E-03	#N/A	#N/A	#N/A	#N/A
TRINITY_DN15960_c0_g1_i12	ZIP7_ORYSJ	1.83	3.12	3.7E-08	3.7E-06	2.09	3.24	1.2E-04	1.9E-02	#N/A	#N/A	#N/A	#N/A

DET	Subject id	c9TR vs c7NT				c7TR vs c7NT				c7TR vs c9TR			
		Log_2	Log_2CPM	PValue	19FDR	Log_2	Log_2CPM	PValue	19FDR	Log_2	Log_2CPM	PValue	19FDR
TRINITY_DN21605_c0_g1_i4	AB11G_ARATH	1.97	2.65	2.7E-07	2.3E-05	2.03	2.62	1.4E-05	3.2E-03	#N/A	#N/A	#N/A	#N/A
TRINITY_DN18844_c1_g7_i1	YSL13_ORYSJ	2.02	2.47	5.4E-08	5.4E-06	1.91	2.30	4.8E-05	9.2E-03	#N/A	#N/A	#N/A	#N/A
TRINITY_DN21121_c1_g1_i6	SC11C_PONAB	2.06	2.19	4.4E-07	3.6E-05	2.09	2.13	1.3E-05	2.9E-03	#N/A	#N/A	#N/A	#N/A
TRINITY_DN20315_c0_g6_i1	ACCO1_ORYSJ	2.09	5.29	3.0E-17	8.9E-15	1.68	4.89	2.6E-06	7.5E-04	#N/A	#N/A	#N/A	#N/A
TRINITY_DN22761_c0_g1_i3	VCR_ARATH	2.09	3.14	2.4E-11	3.8E-09	2.20	3.14	1.5E-08	7.0E-06	#N/A	#N/A	#N/A	#N/A
TRINITY_DN15687_c0_g6_i1	CYPH_CATRO	2.09	0.55	1.2E-04	5.4E-03	2.33	0.65	1.0E-04	1.7E-02	#N/A	#N/A	#N/A	#N/A
TRINITY_DN22030_c3_g1_i4	RFC3_ARATH	2.16	2.73	1.6E-08	1.8E-06	1.83	2.38	4.2E-05	8.2E-03	#N/A	#N/A	#N/A	#N/A
TRINITY_DN19529_c0_g2_i14	XB32_ORYSJ	2.22	1.79	1.3E-05	7.5E-04	2.62	2.03	1.4E-07	5.5E-05	#N/A	#N/A	#N/A	#N/A
TRINITY_DN18112_c1_g1_i7	NUD18_ARATH	2.32	3.49	1.4E-14	3.2E-12	2.40	3.47	2.3E-08	1.0E-05	#N/A	#N/A	#N/A	#N/A
TRINITY_DN15255_c0_g1_i10	MLO1_ARATH	2.32	0.83	6.1E-04	2.1E-02	2.68	1.04	1.8E-04	2.8E-02	#N/A	#N/A	#N/A	#N/A
TRINITY_DN20821_c0_g2_i5	PYRG2_XENLA	2.70	1.34	2.7E-07	2.3E-05	2.93	1.44	8.7E-07	2.8E-04	#N/A	#N/A	#N/A	#N/A
TRINITY_DN15903_c0_g1_i16	ACCO1_ORYSJ	2.71	0.71	3.4E-06	2.2E-04	2.93	0.81	7.1E-06	1.8E-03	#N/A	#N/A	#N/A	#N/A
TRINITY_DN21933_c0_g2_i2	CRK7_ARATH	3.05	0.25	4.1E-05	2.1E-03	3.32	0.40	1.9E-04	2.8E-02	#N/A	#N/A	#N/A	#N/A
TRINITY_DN17090_c0_g2_i12	CATB_PONAB	3.22	0.00	9.3E-05	4.3E-03	3.51	0.17	2.9E-04	4.2E-02	#N/A	#N/A	#N/A	#N/A
TRINITY_DN17001_c0_g1_i3	G11A_ORYSI	3.51	1.27	3.4E-07	2.9E-05	3.53	1.20	4.2E-07	1.5E-04	#N/A	#N/A	#N/A	#N/A
TRINITY_DN15534_c0_g1_i10	AB17C_DANRE	3.59	1.21	6.5E-05	3.2E-03	3.83	1.34	4.6E-06	1.2E-03	#N/A	#N/A	#N/A	#N/A
TRINITY_DN21779_c0_g3_i1	HD3A_ORYSJ	3.83	2.06	7.6E-08	7.4E-06	4.06	2.18	1.4E-09	8.1E-07	#N/A	#N/A	#N/A	#N/A
TRINITY_DN17646_c0_g2_i6	COL9_ARATH	4.19	1.68	3.1E-11	4.9E-09	3.86	1.28	2.1E-06	6.2E-04	#N/A	#N/A	#N/A	#N/A
TRINITY_DN19529_c0_g2_i11	XB32_ORYSJ	4.55	-0.68	5.8E-04	2.0E-02	5.11	-0.27	8.1E-05	1.4E-02	#N/A	#N/A	#N/A	#N/A
TRINITY_DN21561_c0_g1_i9	SFH6_ARATH	4.67	0.78	1.8E-07	1.6E-05	4.46	0.50	6.4E-06	1.6E-03	#N/A	#N/A	#N/A	#N/A
TRINITY_DN15967_c0_g7_i1	MAD15_ORYSJ	4.68	0.78	1.5E-07	1.4E-05	4.54	0.55	9.6E-05	1.6E-02	#N/A	#N/A	#N/A	#N/A
TRINITY_DN15919_c0_g2_i1	TRL31_ORYSJ	5.00	0.84	3.4E-11	5.3E-09	6.21	1.91	3.1E-15	4.2E-12	#N/A	#N/A	#N/A	#N/A
TRINITY_DN22665_c1_g1_i5	PMA2_SOLLC	5.06	2.05	1.2E-09	1.5E-07	5.87	2.75	7.9E-06	1.9E-03	#N/A	#N/A	#N/A	#N/A
TRINITY_DN16357_c0_g1_i7	AN13B_HUMAN	5.09	0.62	1.9E-03	5.1E-02	5.01	0.47	8.3E-05	1.5E-02	#N/A	#N/A	#N/A	#N/A
TRINITY_DN21886_c0_g1_i5	BET11_ARATH	5.12	0.44	1.8E-06	1.3E-04	5.03	0.27	3.6E-05	7.3E-03	#N/A	#N/A	#N/A	#N/A
TRINITY_DN19742_c4_g3_i1	POLX_TOBAC	5.35	0.06	2.9E-10	4.0E-08	5.48	0.10	6.2E-05	1.1E-02	#N/A	#N/A	#N/A	#N/A
TRINITY_DN17810_c0_g4_i2	RBX1_SALSA	5.39	0.97	5.7E-15	1.4E-12	5.64	1.12	2.2E-13	2.3E-10	#N/A	#N/A	#N/A	#N/A
TRINITY_DN17448_c1_g1_i3	MAD15_ORYSJ	5.58	3.19	3.1E-27	2.1E-24	5.92	3.41	1.7E-14	2.3E-11	#N/A	#N/A	#N/A	#N/A
TRINITY_DN21121_c1_g1_i23	SC11C_PONAB	5.78	0.55	1.4E-06	1.0E-04	6.43	1.06	6.3E-10	3.9E-07	#N/A	#N/A	#N/A	#N/A
TRINITY_DN17448_c1_g1_i6	MAD15_ORYSJ	5.84	1.54	3.3E-08	3.4E-06	6.98	2.54	1.8E-05	4.0E-03	#N/A	#N/A	#N/A	#N/A
TRINITY_DN21543_c0_g1_i16	Y005_SYNY3	6.12	2.41	4.2E-05	2.2E-03	6.34	2.54	2.1E-05	4.7E-03	#N/A	#N/A	#N/A	#N/A
TRINITY_DN15122_c1_g3_i13	RRFC_ORYSJ	6.48	-0.14	9.3E-09	1.0E-06	5.94	-0.69	1.1E-05	2.5E-03	#N/A	#N/A	#N/A	#N/A
TRINITY_DN17073_c0_g8_i1	KCS6_ARATH	6.51	-0.11	3.1E-09	3.7E-07	7.02	0.29	1.0E-07	4.1E-05	#N/A	#N/A	#N/A	#N/A
TRINITY_DN20371_c2_g4_i2	DOF54_ARATH	6.71	0.10	6.2E-10	8.1E-08	7.16	0.47	2.0E-10	1.3E-07	#N/A	#N/A	#N/A	#N/A
TRINITY_DN20463_c0_g1_i18	Y2168_ARATH	7.74	-0.77	1.4E-06	1.1E-04	7.93	-0.61	6.7E-06	1.7E-03	#N/A	#N/A	#N/A	#N/A
TRINITY_DN18018_c0_g1_i16	QKIL2_ARATH	7.93	-0.61	5.1E-07	4.1E-05	8.32	-0.25	8.3E-08	3.2E-05	#N/A	#N/A	#N/A	#N/A
TRINITY_DN20613_c0_g4_i6	MFS12_MOUSE	7.99	-0.55	2.6E-07	2.3E-05	7.90	-0.63	4.8E-06	1.3E-03	#N/A	#N/A	#N/A	#N/A
TRINITY_DN15122_c1_g3_i3	RRFC_ORYSJ	8.04	-0.51	4.0E-07	3.3E-05	8.14	-0.41	3.5E-07	1.3E-04	#N/A	#N/A	#N/A	#N/A
TRINITY_DN21213_c0_g1_i1	IBB1_COILA	8.09	-0.46	2.8E-07	2.4E-05	8.69	0.08	2.0E-05	4.4E-03	#N/A	#N/A	#N/A	#N/A
TRINITY_DN17289_c0_g7_i2	CML20_ORYSJ	8.10	-0.46	1.6E-07	1.4E-05	8.12	-0.44	2.9E-05	6.0E-03	#N/A	#N/A	#N/A	#N/A
TRINITY_DN22362_c2_g1_i4	MOC32_MAIZE	8.10	-0.45	3.9E-08	3.9E-06	8.50	-0.09	3.2E-07	1.1E-04	#N/A	#N/A	#N/A	#N/A
TRINITY_DN19672_c0_g3_i5	2A5G_ARATH	8.11	1.39	1.3E-20	5.1E-18	8.29	1.51	9.3E-14	1.1E-10	#N/A	#N/A	#N/A	#N/A
TRINITY_DN19374_c1_g5_i3	ARMC7_MOUSE	8.13	-0.43	1.5E-08	1.6E-06	8.23	-0.33	6.7E-07	2.2E-04	#N/A	#N/A	#N/A	#N/A
TRINITY_DN15521_c1_g2_i10	MBOA1_ARATH	8.15	-0.41	4.5E-04	1.6E-02	9.13	0.50	4.2E-11	3.3E-08	#N/A	#N/A	#N/A	#N/A
TRINITY_DN21040_c0_g1_i4	POLB_MAIZE	8.24	-0.33	3.6E-08	3.7E-06	8.30	-0.27	6.3E-07	2.1E-04	#N/A	#N/A	#N/A	#N/A
TRINITY_DN21418_c0_g3_i4	GLN11_ORYSJ	8.57	3.54	6.8E-53	1.8E-49	8.81	3.72	1.1E-23	4.1E-20	#N/A	#N/A	#N/A	#N/A
TRINITY_DN15371_c0_g1_i16	TRE_ORYSJ	8.73	0.12	1.6E-12	3.0E-10	8.19	-0.37	1.7E-07	6.5E-05	#N/A	#N/A	#N/A	#N/A
TRINITY_DN22669_c0_g2_i4	GIGAN_ORYSJ	8.86	0.24	5.0E-14	1.1E-11	9.05	0.42	1.0E-10	7.4E-08	#N/A	#N/A	#N/A	#N/A
TRINITY_DN20732_c0_g1_i13	NIP21_MAIZE	8.87	0.25	6.9E-11	1.0E-08	9.98	1.31	2.2E-13	2.3E-10	#N/A	#N/A	#N/A	#N/A
TRINITY_DN22027_c0_g1_i7	Y5158_ARATH	8.99	0.36	2.9E-14	6.4E-12	8.92	0.31	6.2E-11	4.6E-08	#N/A	#N/A	#N/A	#N/A
TRINITY_DN15759_c1_g2_i4	P2C27_ORYSJ	9.01	0.38	3.5E-13	6.9E-11	8.45	-0.13	9.6E-08	3.7E-05	#N/A	#N/A	#N/A	#N/A
TRINITY_DN18330_c0_g4_i6	PXG4_ARATH	9.10	0.47	3.8E-13	7.6E-11	8.79	0.18	4.1E-08	1.7E-05	#N/A	#N/A	#N/A	#N/A
TRINITY_DN20287_c0_g5_i2	R35A3_ARATH	9.17	0.53	2.0E-09	2.4E-07	9.42	0.78	1.1E-11	1.0E-08	#N/A	#N/A	#N/A	#N/A
TRINITY_DN16443_c0_g1_i6	ACA1_ORYSJ	9.34	0.70	6.3E-09	7.2E-07	9.22	0.59	2.7E-11	2.2E-08	#N/A	#N/A	#N/A	#N/A
TRINITY_DN15973_c0_g3_i2	FUT1_ARATH	9.55	0.89	1.4E-17	4.1E-15	9.64	0.99	7.2E-13	7.1E-10	#N/A	#N/A	#N/A	#N/A
TRINITY_DN15655_c2_g2_i9	BP73_ORYSJ	9.70	1.03	1.1E-18	3.5E-16	9.31	0.68	3.6E-11	2.8E-08	#N/A	#N/A	#N/A	#N/A
TRINITY_DN21551_c0_g2_i14	YZR3_ARATH	9.80	1.14	2.2E-15	5.5E-13	9.71	1.06	2.1E-13	2.3E-10	#N/A	#N/A	#N/A	#N/A
TRINITY_DN19418_c1_g1_i15	PSAB_SORBI	9.85	1.18	1.2E-12	2.3E-10	10.76	2.08	4.9E-23	1.7E-19	#N/A	#N/A	#N/A	#N/A
TRINITY_DN16357_c0_g1_i10	AN13B_HUMAN	9.88	1.21	2.0E-18	6.5E-16	9.64	0.99	1.0E-14	1.4E-11	#N/A	#N/A	#N/A	#N/A
TRINITY_DN15878_c0_g1_i16	Y5564_ARATH	9.90	1.23	1.3E-10	1.9E-08	7.97	-0.57	8.9E-06	2.1E-03	#N/A	#N/A	#N/A	#N/A
TRINITY_DN22441_c0_g3_i4	KC1D_ARATH	10.02	1.34	8.9E-22	3.7E-19	9.63	0.98	2.3E-11	2.0E-08	#N/A	#N/A	#N/A	#N/A

DET	Subject id	c9TR vs c7NT				c7TR vs c7NT				c7TR vs c9TR			
		Log_2	Log_2CPM	PValue	19FDR	Log_2	Log_2CPM	PValue	19FDR	Log_2	Log_2CPM	PValue	19FDR
TRINITY_DN16027_c1_g3_i8	LPPE2_ARATH	10.02	1.35	2.6E-19	8.9E-17	9.18	0.55	2.1E-13	2.3E-10	#N/A	#N/A	#N/A	#N/A
TRINITY_DN16683_c0_g4_i1	COMT1_AMMMJ	10.10	1.42	3.8E-21	1.5E-18	10.51	1.83	3.0E-19	7.2E-16	#N/A	#N/A	#N/A	#N/A
TRINITY_DN19997_c0_g2_i2	PPR68_ARATH	10.10	1.43	2.9E-22	1.3E-19	10.51	1.84	1.3E-17	2.7E-14	#N/A	#N/A	#N/A	#N/A
TRINITY_DN16683_c0_g4_i4	COMT1_CATRO	10.13	1.45	5.5E-23	2.6E-20	10.01	1.35	1.5E-15	2.2E-12	#N/A	#N/A	#N/A	#N/A
TRINITY_DN19997_c0_g2_i1	PPR68_ARATH	10.14	1.46	3.9E-23	1.9E-20	10.73	2.05	1.3E-15	2.0E-12	#N/A	#N/A	#N/A	#N/A
TRINITY_DN15934_c3_g3_i11	IAA10_ORYSJ	10.56	1.87	4.6E-27	3.0E-24	10.69	2.01	5.3E-20	1.3E-16	#N/A	#N/A	#N/A	#N/A
TRINITY_DN18330_c0_g4_i1	PXG4_ARATH	10.64	1.95	1.3E-26	7.8E-24	10.42	1.75	2.1E-14	2.8E-11	#N/A	#N/A	#N/A	#N/A
TRINITY_DN20463_c0_g1_i2	Y2168_ARATH	10.74	2.05	1.2E-27	8.2E-25	10.57	1.89	3.1E-18	7.2E-15	#N/A	#N/A	#N/A	#N/A
TRINITY_DN14613_c0_g5_i5	CX171_ARATH	10.98	2.28	4.7E-28	3.3E-25	10.40	1.73	2.0E-16	3.2E-13	#N/A	#N/A	#N/A	#N/A
TRINITY_DN22803_c2_g4_i2	OMT1_ORYSJ	11.36	2.66	3.7E-39	5.0E-36	11.25	2.56	4.3E-29	2.3E-25	#N/A	#N/A	#N/A	#N/A
TRINITY_DN19390_c2_g1_i1	UCRIA_WHEAT	11.67	5.71	6.6E-109	1.11E-104	11.32	5.22	2.5E-04	3.6E-02	#N/A	#N/A	#N/A	#N/A
TRINITY_DN15948_c1_g1_i5	PMA1_NICPL	11.94	3.23	1.5E-03	4.2E-02	13.06	4.36	4.6E-07	1.6E-04	#N/A	#N/A	#N/A	#N/A
TRINITY_DN21207_c4_g2_i5	ARF_DUGJA	12.27	3.55	9.9E-46	1.8E-42	12.89	4.19	1.1E-36	1.0E-32	#N/A	#N/A	#N/A	#N/A
TRINITY_DN29579_c0_g1_i1	PAT_STRHY	12.49	5.73	1.6E-99	2.1E-95	12.51	5.67	8.7E-14	1.0E-10	#N/A	#N/A	#N/A	#N/A
TRINITY_DN16626_c0_g3_i8	MAD50_ORYSJ	16.97	8.24	7.1E-182	4.8E-177	16.55	7.84	3.6E-94	2.3E-89	#N/A	#N/A	#N/A	#N/A
TRINITY_DN14712_c0_g2_i12	ATG8C_ORYSJ	#N/A	#N/A	#N/A	#N/A	-11.35	2.66	1.1E-05	2.6E-03	-12.95	4.20	9.3E-54	5.6E-50
TRINITY_DN18269_c0_g1_i11	CYSKP_SOLTU	#N/A	#N/A	#N/A	#N/A	-12.91	4.21	2.1E-45	3.5E-41	-12.50	3.75	3.5E-36	6.4E-33
TRINITY_DN21329_c1_g1_i16	EXEC2_ORYSJ	#N/A	#N/A	#N/A	#N/A	-12.99	4.29	5.5E-44	7.2E-40	-12.39	3.64	2.7E-34	4.2E-31
TRINITY_DN19867_c3_g1_i4	THIC1_ARATH	#N/A	#N/A	#N/A	#N/A	-12.21	3.51	8.5E-35	6.2E-31	-12.02	3.28	1.1E-29	1.2E-26
TRINITY_DN16340_c3_g1_i1	RS51_ARATH	#N/A	#N/A	#N/A	#N/A	-11.69	2.99	6.5E-34	4.2E-30	-11.18	2.44	4.9E-21	2.8E-18
TRINITY_DN21312_c1_g1_i1	EBP_ORYSJ	#N/A	#N/A	#N/A	#N/A	-11.11	2.42	4.4E-25	1.7E-21	-10.88	2.15	4.3E-20	2.4E-17
TRINITY_DN16340_c3_g1_i10	RS52_ARATH	#N/A	#N/A	#N/A	#N/A	-11.30	2.61	2.5E-27	1.1E-23	-10.73	2.00	1.4E-17	5.9E-15
TRINITY_DN15483_c0_g2_i4	ODO1_DICDI	#N/A	#N/A	#N/A	#N/A	-9.90	1.24	2.7E-15	3.9E-12	-10.14	1.43	2.5E-21	1.5E-18
TRINITY_DN20703_c1_g1_i2	PRR37_ORYSI	#N/A	#N/A	#N/A	#N/A	-8.97	0.35	3.0E-08	1.3E-05	-10.13	1.42	3.0E-13	8.0E-11
TRINITY_DN18629_c0_g1_i16	CCX4_ARATH	#N/A	#N/A	#N/A	#N/A	-10.53	1.85	1.6E-21	4.6E-18	-10.05	1.34	1.6E-14	4.9E-12
TRINITY_DN21426_c2_g1_i17	CCD4_ARATH	#N/A	#N/A	#N/A	#N/A	-9.45	0.81	5.9E-07	2.0E-04	-9.86	1.16	2.8E-12	6.7E-10
TRINITY_DN20151_c0_g1_i16	RH3_ORYSJ	#N/A	#N/A	#N/A	#N/A	-10.13	1.46	4.2E-17	7.6E-14	-9.56	0.87	9.8E-16	3.5E-13
TRINITY_DN16969_c1_g1_i1	DXR_ORYSJ	#N/A	#N/A	#N/A	#N/A	-9.86	1.20	3.8E-17	7.1E-14	-9.51	0.82	1.4E-10	2.7E-08
TRINITY_DN21312_c1_g2_i3	TL1Y_ARATH	#N/A	#N/A	#N/A	#N/A	-9.41	4.54	3.7E-36	3.0E-32	-9.09	4.26	4.7E-38	1.1E-34
TRINITY_DN16462_c0_g3_i1	CYT12_ORYSJ	#N/A	#N/A	#N/A	#N/A	-8.58	-0.02	1.4E-09	8.0E-07	-9.07	0.40	3.5E-14	1.1E-11
TRINITY_DN20256_c1_g2_i5	HT1_ARATH	#N/A	#N/A	#N/A	#N/A	-7.88	-0.65	6.0E-06	1.5E-03	-8.60	-0.03	4.5E-07	5.3E-05
TRINITY_DN20417_c1_g1_i6	ASD_ARATH	#N/A	#N/A	#N/A	#N/A	-9.98	1.32	4.7E-09	2.5E-06	-8.39	-0.23	6.6E-07	7.4E-05
TRINITY_DN19163_c1_g1_i5	RFA3_ORYSJ	#N/A	#N/A	#N/A	#N/A	-8.24	-0.33	2.6E-06	7.3E-04	-8.37	-0.25	8.9E-09	1.4E-06
TRINITY_DN22411_c0_g3_i20	C3H28_ORYSJ	#N/A	#N/A	#N/A	#N/A	-8.54	-0.06	6.1E-09	3.1E-06	-8.37	-0.25	1.1E-05	1.0E-03
TRINITY_DN15749_c0_g1_i3	TTL_ARATH	#N/A	#N/A	#N/A	#N/A	-8.16	-0.41	2.7E-05	5.7E-03	-8.27	-0.34	2.7E-08	3.9E-06
TRINITY_DN14764_c0_g1_i3	PERK8_ARATH	#N/A	#N/A	#N/A	#N/A	-7.84	-0.68	5.3E-06	1.4E-03	-8.24	-0.37	5.3E-06	5.3E-04
TRINITY_DN20955_c0_g1_i12	PSBE_WHEAT	#N/A	#N/A	#N/A	#N/A	-8.24	-0.32	2.8E-06	7.8E-04	-7.97	-0.61	5.2E-07	6.0E-05
TRINITY_DN17007_c0_g1_i2	SAMH1_DICDI	#N/A	#N/A	#N/A	#N/A	-8.19	-0.38	6.4E-07	2.1E-04	-7.68	-0.87	4.1E-06	4.1E-04
TRINITY_DN21312_c1_g2_i13	EBP_ORYSJ	#N/A	#N/A	#N/A	#N/A	-6.97	3.28	3.4E-05	6.8E-03	-7.07	3.43	5.4E-05	4.5E-03
TRINITY_DN20755_c0_g1_i6	TM1L2_MOUSE	#N/A	#N/A	#N/A	#N/A	-6.03	0.04	5.2E-06	1.4E-03	-6.72	0.76	5.2E-13	1.4E-10
TRINITY_DN22204_c1_g1_i5	DXR_ORYSJ	#N/A	#N/A	#N/A	#N/A	-6.29	0.84	2.4E-12	2.3E-09	-6.19	0.80	3.2E-10	6.0E-08
TRINITY_DN18298_c0_g1_i1	MY1R1_SOLTU	#N/A	#N/A	#N/A	#N/A	-5.89	0.39	1.2E-08	5.4E-06	-5.12	-0.29	8.9E-05	6.9E-03
TRINITY_DN17007_c0_g1_i10	SAMH1_CHICK	#N/A	#N/A	#N/A	#N/A	-5.35	1.00	4.8E-09	2.5E-06	-5.05	0.77	4.6E-07	5.4E-05
TRINITY_DN18257_c0_g3_i11	TYPA_BACSU	#N/A	#N/A	#N/A	#N/A	-3.68	1.14	5.0E-06	1.3E-03	-3.69	1.21	4.7E-06	4.7E-04
TRINITY_DN21312_c1_g2_i14	EBP_ORYSJ	#N/A	#N/A	#N/A	#N/A	-2.21	2.53	1.7E-04	2.7E-02	-1.67	2.16	2.9E-05	2.6E-03
TRINITY_DN18264_c1_g4_i1	POLX_TOBAC	#N/A	#N/A	#N/A	#N/A	1.77	4.74	2.1E-06	6.2E-04	1.22	4.94	1.8E-04	1.3E-02
TRINITY_DN15960_c0_g1_i10	ZIP7_ORYSJ	#N/A	#N/A	#N/A	#N/A	3.37	4.32	8.5E-09	4.2E-06	1.83	4.60	4.1E-04	2.7E-02
TRINITY_DN21312_c1_g2_i6	EBP_ORYSJ	#N/A	#N/A	#N/A	#N/A	2.55	2.41	4.5E-07	1.6E-04	2.07	2.54	1.1E-05	1.1E-03
TRINITY_DN19542_c0_g1_i2	YCF23_PYRYE	#N/A	#N/A	#N/A	#N/A	5.41	4.59	1.3E-04	2.1E-02	5.83	4.64	7.6E-05	6.0E-03
TRINITY_DN15852_c0_g3_i7	HMGCL_ARATH	#N/A	#N/A	#N/A	#N/A	8.76	0.15	5.3E-11	4.1E-08	6.25	0.21	1.6E-10	3.0E-08
TRINITY_DN16751_c1_g1_i2	ORG2_ARATH	#N/A	#N/A	#N/A	#N/A	9.34	2.59	4.2E-10	2.6E-07	7.11	2.65	9.0E-08	1.2E-05
TRINITY_DN16751_c1_g1_i3	ORG2_ARATH	#N/A	#N/A	#N/A	#N/A	11.60	2.91	3.5E-13	3.6E-10	7.74	2.97	5.9E-11	1.2E-08
TRINITY_DN20151_c0_g1_i10	RH3_ORYSJ	#N/A	#N/A	#N/A	#N/A	6.84	4.52	1.1E-04	1.8E-02	8.29	4.57	3.0E-04	2.0E-02
TRINITY_DN19434_c0_g2_i1	MTN1_ARATH	#N/A	#N/A	#N/A	#N/A	8.33	-0.25	5.3E-07	1.8E-04	8.43	-0.21	8.7E-08	1.2E-05
TRINITY_DN15649_c0_g3_i7	DCUP1_ORYSJ	#N/A	#N/A	#N/A	#N/A	5.23	-0.19	3.0E-04	4.3E-02	8.47	-0.18	3.6E-07	4.4E-05
TRINITY_DN18075_c4_g1_i4	RK18_ORYSJ	#N/A	#N/A	#N/A	#N/A	8.41	-0.17	2.5E-04	3.6E-02	8.52	-0.14	1.5E-04	1.1E-02
TRINITY_DN15749_c0_g1_i12	TTL_ARATH	#N/A	#N/A	#N/A	#N/A	8.51	-0.08	5.8E-08	2.3E-05	8.61	-0.04	5.3E-09	8.6E-07
TRINITY_DN14555_c0_g1_i3	PGP2_ARATH	#N/A	#N/A	#N/A	#N/A	8.59	-0.01	3.8E-06	1.0E-03	8.69	0.03	1.2E-06	1.3E-04
TRINITY_DN18189_c1_g2_i2	ELP6_ARATH	#N/A	#N/A	#N/A	#N/A	8.70	0.10	8.1E-10	4.9E-07	8.81	0.14	2.4E-11	5.2E-09
TRINITY_DN19730_c0_g1_i4	C7016_ORYSJ	#N/A	#N/A	#N/A	#N/A	9.13	0.50	7.4E-05	1.3E-02	9.23	0.54	4.8E-05	4.1E-03
TRINITY_DN16260_c4_g3_i3	Y4141_ARATH	#N/A	#N/A	#N/A	#N/A	9.24	0.60	7.6E-05	1.3E-02	9.35	0.65	5.3E-05	4.4E-03

DET	Subject id	c9TR vs c7NT				c7TR vs c7NT				c7TR vs c9TR			
		Log_2	Log_2CPM	PValue	19FDR	Log_2	Log_2CPM	PValue	19FDR	Log_2	Log_2CPM	PValue	19FDR
TRINITY_DN17985_c0_g1_i14	RN170_XENLA	#N/A	#N/A	#N/A	#N/A	7.35	3.40	5.3E-05	1.0E-02	9.67	3.44	2.6E-06	2.7E-04
TRINITY_DN15795_c0_g1_i2	KU80_ORYSJ	#N/A	#N/A	#N/A	#N/A	9.57	0.92	1.8E-06	5.4E-04	9.67	0.96	9.4E-07	1.0E-04
TRINITY_DN18461_c1_g2_i5	PMT2_ARATH	#N/A	#N/A	#N/A	#N/A	9.64	0.99	4.2E-09	2.3E-06	9.74	1.04	1.0E-09	1.8E-07
TRINITY_DN20106_c0_g1_i6	C3H25_ORYSJ	#N/A	#N/A	#N/A	#N/A	9.97	1.31	8.3E-07	2.7E-04	10.07	1.35	4.3E-07	5.1E-05
TRINITY_DN15111_c0_g2_i1	Y5224_ARATH	#N/A	#N/A	#N/A	#N/A	10.08	1.42	3.9E-05	7.7E-03	10.19	1.46	2.8E-05	2.5E-03
TRINITY_DN14888_c0_g2_i9	AMT32_ORYSJ	#N/A	#N/A	#N/A	#N/A	10.20	1.53	1.1E-05	2.6E-03	10.30	1.58	7.5E-06	7.2E-04
TRINITY_DN18350_c1_g2_i3	B2_DAUCA	#N/A	#N/A	#N/A	#N/A	10.78	2.10	4.7E-05	9.0E-03	10.89	2.15	3.7E-05	3.2E-03
TRINITY_DN16462_c0_g3_i4	CYT12_ORYSJ	#N/A	#N/A	#N/A	#N/A	10.99	2.30	2.1E-25	8.5E-22	11.09	2.36	1.0E-31	1.3E-28
TRINITY_DN20066_c0_g3_i1	AKRCA_ARATH	#N/A	#N/A	#N/A	#N/A	11.06	2.37	2.7E-05	5.7E-03	11.16	2.42	2.1E-05	1.9E-03
TRINITY_DN14888_c0_g2_i4	AMT32_ORYSJ	#N/A	#N/A	#N/A	#N/A	11.81	3.11	8.2E-06	2.0E-03	11.91	3.16	6.7E-06	6.5E-04
TRINITY_DN15711_c0_g2_i9	FER3_MAIZE	#N/A	#N/A	#N/A	#N/A	12.35	3.65	1.5E-08	6.9E-06	12.45	3.70	9.4E-09	1.5E-06
TRINITY_DN16330_c1_g2_i4	AKR2A_ARATH	#N/A	#N/A	#N/A	#N/A	12.37	3.67	8.3E-07	2.7E-04	12.47	3.72	6.6E-07	7.4E-05
TRINITY_DN22035_c0_g2_i9	AAMT3_MAIZE	#N/A	#N/A	#N/A	#N/A	-10.83	2.15	4.8E-07	1.6E-04	#N/A	#N/A	#N/A	#N/A
TRINITY_DN17767_c0_g1_i5	BLH8_ARATH	#N/A	#N/A	#N/A	#N/A	-10.64	1.96	2.1E-17	3.9E-14	#N/A	#N/A	#N/A	#N/A
TRINITY_DN18936_c0_g1_i1	Y5614_ARATH	#N/A	#N/A	#N/A	#N/A	-10.56	1.88	7.6E-17	1.3E-13	#N/A	#N/A	#N/A	#N/A
TRINITY_DN19672_c0_g3_i9	2A5G_ARATH	#N/A	#N/A	#N/A	#N/A	-10.36	1.69	3.8E-18	8.6E-15	#N/A	#N/A	#N/A	#N/A
TRINITY_DN20296_c0_g1_i12	PP399_ARATH	#N/A	#N/A	#N/A	#N/A	-10.30	1.62	8.3E-08	3.2E-05	#N/A	#N/A	#N/A	#N/A
TRINITY_DN19418_c1_g1_i6	PSAB_SORBI	#N/A	#N/A	#N/A	#N/A	-10.03	1.36	2.3E-10	1.5E-07	#N/A	#N/A	#N/A	#N/A
TRINITY_DN21095_c2_g2_i13	Y3913_ARATH	#N/A	#N/A	#N/A	#N/A	-9.52	0.86	2.4E-06	7.0E-04	#N/A	#N/A	#N/A	#N/A
TRINITY_DN15383_c0_g1_i9	NIPL2_ARATH	#N/A	#N/A	#N/A	#N/A	-9.45	0.81	1.6E-06	4.9E-04	#N/A	#N/A	#N/A	#N/A
TRINITY_DN22700_c4_g3_i4	HFA2E_ORYSJ	#N/A	#N/A	#N/A	#N/A	-9.41	0.78	1.8E-04	2.8E-02	#N/A	#N/A	#N/A	#N/A
TRINITY_DN22044_c2_g1_i1	RCD1_ARATH	#N/A	#N/A	#N/A	#N/A	-9.18	0.55	1.7E-10	1.2E-07	#N/A	#N/A	#N/A	#N/A
TRINITY_DN19697_c0_g1_i13	SSY22_ORYSJ	#N/A	#N/A	#N/A	#N/A	-8.98	0.35	6.6E-07	2.2E-04	#N/A	#N/A	#N/A	#N/A
TRINITY_DN21163_c3_g5_i7	XYNA_THESA	#N/A	#N/A	#N/A	#N/A	-8.97	0.35	5.6E-08	2.3E-05	#N/A	#N/A	#N/A	#N/A
TRINITY_DN20711_c0_g3_i25	VP52A_ARATH	#N/A	#N/A	#N/A	#N/A	-8.94	0.32	6.2E-11	4.6E-08	#N/A	#N/A	#N/A	#N/A
TRINITY_DN14828_c0_g1_i16	4CL3_ORYSJ	#N/A	#N/A	#N/A	#N/A	-8.85	0.24	1.5E-06	4.6E-04	#N/A	#N/A	#N/A	#N/A
TRINITY_DN21370_c0_g2_i3	KPRS4_ORYSJ	#N/A	#N/A	#N/A	#N/A	-8.60	0.00	5.6E-08	2.3E-05	#N/A	#N/A	#N/A	#N/A
TRINITY_DN18085_c0_g1_i12	DIV_ANTMA	#N/A	#N/A	#N/A	#N/A	-8.52	-0.07	7.7E-09	3.8E-06	#N/A	#N/A	#N/A	#N/A
TRINITY_DN18189_c1_g2_i5	ELP6_ARATH	#N/A	#N/A	#N/A	#N/A	-8.43	-0.15	2.1E-06	6.1E-04	#N/A	#N/A	#N/A	#N/A
TRINITY_DN17686_c3_g1_i4	SVKA_DICDI	#N/A	#N/A	#N/A	#N/A	-8.26	-0.31	4.5E-07	1.6E-04	#N/A	#N/A	#N/A	#N/A
TRINITY_DN20614_c1_g1_i9	CALS5_ARATH	#N/A	#N/A	#N/A	#N/A	-8.18	-0.38	2.4E-05	5.2E-03	#N/A	#N/A	#N/A	#N/A
TRINITY_DN15254_c1_g4_i5	PEX1_ARATH	#N/A	#N/A	#N/A	#N/A	-8.03	-0.52	7.5E-07	2.4E-04	#N/A	#N/A	#N/A	#N/A
TRINITY_DN21021_c0_g2_i2	CALSC_ARATH	#N/A	#N/A	#N/A	#N/A	-7.98	-0.56	1.9E-05	4.3E-03	#N/A	#N/A	#N/A	#N/A
TRINITY_DN21410_c0_g1_i14	NACK1_TOBAC	#N/A	#N/A	#N/A	#N/A	-7.93	-0.60	7.2E-06	1.8E-03	#N/A	#N/A	#N/A	#N/A
TRINITY_DN14680_c1_g2_i9	RTE1_ARATH	#N/A	#N/A	#N/A	#N/A	-7.91	-0.62	3.5E-06	9.6E-04	#N/A	#N/A	#N/A	#N/A
TRINITY_DN19399_c0_g1_i5	PP434_ARATH	#N/A	#N/A	#N/A	#N/A	-7.87	-0.66	5.6E-06	1.4E-03	#N/A	#N/A	#N/A	#N/A
TRINITY_DN17007_c0_g1_i11	SAMH1_DANRE	#N/A	#N/A	#N/A	#N/A	-7.81	-0.72	8.6E-06	2.1E-03	#N/A	#N/A	#N/A	#N/A
TRINITY_DN20441_c2_g3_i10	B120_ARATH	#N/A	#N/A	#N/A	#N/A	-7.79	-0.72	6.2E-05	1.1E-02	#N/A	#N/A	#N/A	#N/A
TRINITY_DN19465_c1_g9_i5	NAC48_ORYSJ	#N/A	#N/A	#N/A	#N/A	-7.52	0.76	3.0E-06	8.4E-04	#N/A	#N/A	#N/A	#N/A
TRINITY_DN12980_c0_g1_i5	MIEL1_ARATH	#N/A	#N/A	#N/A	#N/A	-7.46	-1.01	2.1E-04	3.1E-02	#N/A	#N/A	#N/A	#N/A
TRINITY_DN22770_c4_g2_i5	GRV2_ARATH	#N/A	#N/A	#N/A	#N/A	-7.27	3.95	1.2E-28	6.2E-25	#N/A	#N/A	#N/A	#N/A
TRINITY_DN18669_c0_g1_i21	D14_ORYSJ	#N/A	#N/A	#N/A	#N/A	-6.49	-0.18	3.5E-04	4.9E-02	#N/A	#N/A	#N/A	#N/A
TRINITY_DN19682_c0_g1_i6	PFPB_RICCO	#N/A	#N/A	#N/A	#N/A	-5.89	1.15	1.2E-04	2.0E-02	#N/A	#N/A	#N/A	#N/A
TRINITY_DN21410_c0_g1_i18	NACK1_TOBAC	#N/A	#N/A	#N/A	#N/A	-5.86	-0.05	2.6E-08	1.1E-05	#N/A	#N/A	#N/A	#N/A
TRINITY_DN21561_c0_g1_i10	SFH6_ARATH	#N/A	#N/A	#N/A	#N/A	-5.40	-0.45	3.8E-05	7.6E-03	#N/A	#N/A	#N/A	#N/A
TRINITY_DN21150_c0_g1_i5	PXM16_ARATH	#N/A	#N/A	#N/A	#N/A	-5.18	1.62	3.1E-05	6.4E-03	#N/A	#N/A	#N/A	#N/A
TRINITY_DN20423_c0_g1_i15	FBT1_ARATH	#N/A	#N/A	#N/A	#N/A	-4.88	-0.22	1.6E-04	2.6E-02	#N/A	#N/A	#N/A	#N/A
TRINITY_DN15934_c3_g3_i5	IAA10_ORYSJ	#N/A	#N/A	#N/A	#N/A	-4.46	-0.46	1.6E-04	2.6E-02	#N/A	#N/A	#N/A	#N/A
TRINITY_DN22027_c0_g1_i1	Y5158_ARATH	#N/A	#N/A	#N/A	#N/A	-3.81	0.38	6.0E-05	1.1E-02	#N/A	#N/A	#N/A	#N/A
TRINITY_DN18685_c0_g1_i1	AAP7_ARATH	#N/A	#N/A	#N/A	#N/A	-3.32	1.16	3.0E-05	6.2E-03	#N/A	#N/A	#N/A	#N/A
TRINITY_DN17151_c1_g3_i3	Y5830_ARATH	#N/A	#N/A	#N/A	#N/A	-2.61	2.18	5.0E-05	9.6E-03	#N/A	#N/A	#N/A	#N/A
TRINITY_DN22204_c1_g1_i16	DXR_ORYSJ	#N/A	#N/A	#N/A	#N/A	-2.30	0.86	5.3E-05	1.0E-02	#N/A	#N/A	#N/A	#N/A
TRINITY_DN22276_c2_g8_i2	HFC2A_ORYSJ	#N/A	#N/A	#N/A	#N/A	-2.24	1.86	9.0E-05	1.6E-02	#N/A	#N/A	#N/A	#N/A
TRINITY_DN15534_c0_g1_i5	AB17C_DANRE	#N/A	#N/A	#N/A	#N/A	-1.97	2.61	2.6E-04	3.8E-02	#N/A	#N/A	#N/A	#N/A
TRINITY_DN21406_c0_g2_i8	NSN1_ARATH	#N/A	#N/A	#N/A	#N/A	-1.85	1.85	2.3E-04	3.4E-02	#N/A	#N/A	#N/A	#N/A
TRINITY_DN17478_c0_g2_i1	WAK3_ARATH	#N/A	#N/A	#N/A	#N/A	-1.78	3.11	2.1E-04	3.1E-02	#N/A	#N/A	#N/A	#N/A
TRINITY_DN20463_c0_g1_i19	Y2168_ARATH	#N/A	#N/A	#N/A	#N/A	-1.74	2.28	2.0E-04	3.0E-02	#N/A	#N/A	#N/A	#N/A
TRINITY_DN14613_c0_g5_i3	CX171_ARATH	#N/A	#N/A	#N/A	#N/A	-1.71	2.43	4.2E-05	8.3E-03	#N/A	#N/A	#N/A	#N/A
TRINITY_DN16626_c0_g3_i3	MAD50_ORYSJ	#N/A	#N/A	#N/A	#N/A	-1.61	3.25	1.8E-05	4.0E-03	#N/A	#N/A	#N/A	#N/A
TRINITY_DN16232_c1_g2_i12	ODBA2_ARATH	#N/A	#N/A	#N/A	#N/A	1.13	6.44	2.0E-04	3.0E-02	#N/A	#N/A	#N/A	#N/A
TRINITY_DN17448_c1_g2_i6	MAD14_ORYSI	#N/A	#N/A	#N/A	#N/A	1.38	5.16	2.2E-05	4.8E-03	#N/A	#N/A	#N/A	#N/A

DET	Subject id	c9TR vs c7NT				c7TR vs c7NT				c7TR vs c9TR			
		Log_2	Log_2CPM	PValue	19FDR	Log_2	Log_2CPM	PValue	19FDR	Log_2	Log_2CPM	PValue	19FDR
TRINITY_DN18519_c0_g1_i13	TIP41_MAIZE	#N/A	#N/A	#N/A	#N/A	1.38	5.32	4.5E-05	8.7E-03	#N/A	#N/A	#N/A	#N/A
TRINITY_DN19867_c3_g1_i1	THIC1_ARATH	#N/A	#N/A	#N/A	#N/A	1.51	4.47	1.2E-06	3.6E-04	#N/A	#N/A	#N/A	#N/A
TRINITY_DN21451_c1_g2_i1	KSG3_ARATH	#N/A	#N/A	#N/A	#N/A	1.57	3.75	2.9E-05	6.0E-03	#N/A	#N/A	#N/A	#N/A
TRINITY_DN18629_c0_g1_i1	CCX4_ARATH	#N/A	#N/A	#N/A	#N/A	1.63	2.95	1.8E-05	4.0E-03	#N/A	#N/A	#N/A	#N/A
TRINITY_DN21312_c1_g2_i9	EBP_ORYSJ	#N/A	#N/A	#N/A	#N/A	1.68	4.58	6.4E-05	1.2E-02	#N/A	#N/A	#N/A	#N/A
TRINITY_DN14229_c0_g1_i4	PHP5_ORYSJ	#N/A	#N/A	#N/A	#N/A	1.95	1.89	1.7E-04	2.6E-02	#N/A	#N/A	#N/A	#N/A
TRINITY_DN17977_c0_g2_i8	Y5458_ARATH	#N/A	#N/A	#N/A	#N/A	1.95	2.61	3.3E-05	6.8E-03	#N/A	#N/A	#N/A	#N/A
TRINITY_DN15554_c2_g1_i22	CSLH2_ORYSI	#N/A	#N/A	#N/A	#N/A	1.98	4.15	1.8E-04	2.8E-02	#N/A	#N/A	#N/A	#N/A
TRINITY_DN18519_c0_g1_i14	TIP41_MAIZE	#N/A	#N/A	#N/A	#N/A	2.08	4.82	2.9E-04	4.1E-02	#N/A	#N/A	#N/A	#N/A
TRINITY_DN16471_c0_g1_i3	FBW2_ARATH	#N/A	#N/A	#N/A	#N/A	2.45	2.58	2.2E-04	3.2E-02	#N/A	#N/A	#N/A	#N/A
TRINITY_DN19739_c0_g1_i3	ZMYM1_HUMAN	#N/A	#N/A	#N/A	#N/A	2.47	3.68	2.9E-05	6.0E-03	#N/A	#N/A	#N/A	#N/A
TRINITY_DN15869_c0_g7_i5	NAC68_ORYSJ	#N/A	#N/A	#N/A	#N/A	2.80	4.63	3.4E-04	4.8E-02	#N/A	#N/A	#N/A	#N/A
TRINITY_DN18316_c0_g3_i9	NADO1_ORYSJ	#N/A	#N/A	#N/A	#N/A	2.85	0.00	9.8E-05	1.7E-02	#N/A	#N/A	#N/A	#N/A
TRINITY_DN19739_c0_g1_i10	ZMYM1_HUMAN	#N/A	#N/A	#N/A	#N/A	2.92	4.49	2.4E-05	5.1E-03	#N/A	#N/A	#N/A	#N/A
TRINITY_DN12195_c0_g1_i3	FLS_PETHY	#N/A	#N/A	#N/A	#N/A	2.93	-0.30	3.4E-04	4.8E-02	#N/A	#N/A	#N/A	#N/A
TRINITY_DN20586_c0_g4_i5	NIR_MAIZE	#N/A	#N/A	#N/A	#N/A	3.09	0.06	2.0E-04	3.0E-02	#N/A	#N/A	#N/A	#N/A
TRINITY_DN22338_c0_g1_i15	HAK2_ORYSJ	#N/A	#N/A	#N/A	#N/A	3.38	1.75	4.0E-04	5.5E-02	#N/A	#N/A	#N/A	#N/A
TRINITY_DN19739_c0_g1_i9	ZMYM1_HUMAN	#N/A	#N/A	#N/A	#N/A	3.42	2.37	6.4E-06	1.6E-03	#N/A	#N/A	#N/A	#N/A
TRINITY_DN14367_c0_g1_i2	HKT8_ORYSI	#N/A	#N/A	#N/A	#N/A	3.86	0.63	2.8E-05	5.9E-03	#N/A	#N/A	#N/A	#N/A
TRINITY_DN20250_c1_g1_i14	RGLG1_ARATH	#N/A	#N/A	#N/A	#N/A	3.99	1.20	1.8E-04	2.7E-02	#N/A	#N/A	#N/A	#N/A
TRINITY_DN17304_c0_g3_i1	RADL3_ARATH	#N/A	#N/A	#N/A	#N/A	4.32	2.94	5.5E-05	1.0E-02	#N/A	#N/A	#N/A	#N/A
TRINITY_DN16628_c0_g2_i9	PGLR_VITVI	#N/A	#N/A	#N/A	#N/A	4.79	1.63	1.7E-04	2.6E-02	#N/A	#N/A	#N/A	#N/A
TRINITY_DN22770_c4_g2_i7	GRV2_ARATH	#N/A	#N/A	#N/A	#N/A	5.12	4.29	2.2E-07	8.1E-05	#N/A	#N/A	#N/A	#N/A
TRINITY_DN20085_c0_g1_i12	AKT1_ORYSJ	#N/A	#N/A	#N/A	#N/A	5.44	-0.46	1.4E-04	2.2E-02	#N/A	#N/A	#N/A	#N/A
TRINITY_DN21341_c0_g1_i9	TBCD_ARATH	#N/A	#N/A	#N/A	#N/A	6.43	0.45	5.2E-05	9.9E-03	#N/A	#N/A	#N/A	#N/A
TRINITY_DN22204_c1_g1_i7	DXR_ORYSJ	#N/A	#N/A	#N/A	#N/A	6.60	-0.06	3.7E-08	1.5E-05	#N/A	#N/A	#N/A	#N/A
TRINITY_DN21365_c0_g1_i9	YLS3_ARATH	#N/A	#N/A	#N/A	#N/A	7.29	2.94	3.7E-04	5.1E-02	#N/A	#N/A	#N/A	#N/A
TRINITY_DN15334_c1_g3_i9	DCNL2_MOUSE	#N/A	#N/A	#N/A	#N/A	7.33	-1.12	2.5E-04	3.6E-02	#N/A	#N/A	#N/A	#N/A
TRINITY_DN18596_c1_g1_i3	Y1491_ARATH	#N/A	#N/A	#N/A	#N/A	7.70	-0.80	9.8E-05	1.7E-02	#N/A	#N/A	#N/A	#N/A
TRINITY_DN21751_c2_g3_i3	PAO2_ARATH	#N/A	#N/A	#N/A	#N/A	7.95	-0.58	2.6E-05	5.6E-03	#N/A	#N/A	#N/A	#N/A
TRINITY_DN18596_c1_g1_i6	Y1491_ARATH	#N/A	#N/A	#N/A	#N/A	7.95	-0.58	3.8E-06	1.0E-03	#N/A	#N/A	#N/A	#N/A
TRINITY_DN22280_c1_g2_i8	DNJH_CUCSA	#N/A	#N/A	#N/A	#N/A	8.20	-0.37	3.4E-04	4.8E-02	#N/A	#N/A	#N/A	#N/A
TRINITY_DN18740_c0_g1_i5	OBGM_ORYSJ	#N/A	#N/A	#N/A	#N/A	8.22	-0.34	5.1E-07	1.7E-04	#N/A	#N/A	#N/A	#N/A
TRINITY_DN20800_c0_g3_i4	RS26_ORYSJ	#N/A	#N/A	#N/A	#N/A	8.24	-0.33	4.6E-05	8.9E-03	#N/A	#N/A	#N/A	#N/A
TRINITY_DN19912_c0_g3_i6	MD33A_ARATH	#N/A	#N/A	#N/A	#N/A	8.27	-0.30	3.1E-06	8.5E-04	#N/A	#N/A	#N/A	#N/A
TRINITY_DN22062_c0_g1_i3	MIRO1_ARATH	#N/A	#N/A	#N/A	#N/A	8.28	-0.30	3.6E-04	5.1E-02	#N/A	#N/A	#N/A	#N/A
TRINITY_DN22313_c0_g1_i11	RNP1_ARATH	#N/A	#N/A	#N/A	#N/A	8.31	-0.27	5.5E-05	1.0E-02	#N/A	#N/A	#N/A	#N/A
TRINITY_DN15465_c2_g3_i9	LRK41_ARATH	#N/A	#N/A	#N/A	#N/A	8.33	-0.25	2.7E-04	3.9E-02	#N/A	#N/A	#N/A	#N/A
TRINITY_DN16511_c0_g3_i5	SCRL1_ORYSJ	#N/A	#N/A	#N/A	#N/A	8.39	2.33	1.0E-05	2.4E-03	#N/A	#N/A	#N/A	#N/A
TRINITY_DN20461_c0_g1_i8	SR140_MOUSE	#N/A	#N/A	#N/A	#N/A	8.40	2.37	2.6E-09	1.5E-06	#N/A	#N/A	#N/A	#N/A
TRINITY_DN19617_c2_g1_i15	GSTX2_MAIZE	#N/A	#N/A	#N/A	#N/A	8.46	-0.12	2.4E-08	1.1E-05	#N/A	#N/A	#N/A	#N/A
TRINITY_DN17391_c2_g1_i1	SSDH_ORYSJ	#N/A	#N/A	#N/A	#N/A	8.50	-0.09	2.4E-08	1.1E-05	#N/A	#N/A	#N/A	#N/A
TRINITY_DN18989_c0_g1_i14	ABAH2_ORYSI	#N/A	#N/A	#N/A	#N/A	8.54	-0.05	4.4E-07	1.6E-04	#N/A	#N/A	#N/A	#N/A
TRINITY_DN20513_c2_g2_i19	SIPL4_ORYSJ	#N/A	#N/A	#N/A	#N/A	8.55	-0.04	4.1E-06	1.1E-03	#N/A	#N/A	#N/A	#N/A
TRINITY_DN22027_c0_g1_i4	Y5158_ARATH	#N/A	#N/A	#N/A	#N/A	8.63	0.03	5.3E-10	3.3E-07	#N/A	#N/A	#N/A	#N/A
TRINITY_DN17984_c0_g3_i2	NAC67_ORYSJ	#N/A	#N/A	#N/A	#N/A	8.63	0.03	1.4E-04	2.2E-02	#N/A	#N/A	#N/A	#N/A
TRINITY_DN16271_c6_g1_i9	FUCO2_ARATH	#N/A	#N/A	#N/A	#N/A	8.63	0.03	2.4E-05	5.1E-03	#N/A	#N/A	#N/A	#N/A
TRINITY_DN19135_c2_g2_i13	UGE4_ORYSJ	#N/A	#N/A	#N/A	#N/A	8.67	0.07	2.8E-07	1.0E-04	#N/A	#N/A	#N/A	#N/A
TRINITY_DN22867_c5_g1_i8	POLX_TOBAC	#N/A	#N/A	#N/A	#N/A	8.69	0.09	2.8E-10	1.8E-07	#N/A	#N/A	#N/A	#N/A
TRINITY_DN16782_c0_g1_i2	Y4141_ARATH	#N/A	#N/A	#N/A	#N/A	8.69	0.09	6.9E-06	1.7E-03	#N/A	#N/A	#N/A	#N/A
TRINITY_DN21431_c0_g1_i14	Y1534_ARATH	#N/A	#N/A	#N/A	#N/A	8.79	0.18	1.8E-10	1.2E-07	#N/A	#N/A	#N/A	#N/A
TRINITY_DN17258_c0_g4_i3	GLR35_ARATH	#N/A	#N/A	#N/A	#N/A	8.85	0.24	4.3E-09	2.3E-06	#N/A	#N/A	#N/A	#N/A
TRINITY_DN22477_c2_g1_i13	FBX14_ARATH	#N/A	#N/A	#N/A	#N/A	8.87	0.25	1.1E-11	1.0E-08	#N/A	#N/A	#N/A	#N/A
TRINITY_DN17879_c1_g3_i1	NH2L1_XENLA	#N/A	#N/A	#N/A	#N/A	9.03	0.40	5.0E-05	9.5E-03	#N/A	#N/A	#N/A	#N/A
TRINITY_DN15255_c0_g1_i16	MLO1_ARATH	#N/A	#N/A	#N/A	#N/A	9.08	0.46	6.5E-09	3.2E-06	#N/A	#N/A	#N/A	#N/A
TRINITY_DN15234_c0_g3_i1	C7A14_ARATH	#N/A	#N/A	#N/A	#N/A	9.12	2.28	1.7E-04	2.7E-02	#N/A	#N/A	#N/A	#N/A
TRINITY_DN13575_c0_g1_i3	Y2910_ARATH	#N/A	#N/A	#N/A	#N/A	9.14	0.51	2.5E-04	3.7E-02	#N/A	#N/A	#N/A	#N/A
TRINITY_DN16486_c0_g1_i20	SCP51_ARATH	#N/A	#N/A	#N/A	#N/A	9.20	0.56	5.0E-13	5.0E-10	#N/A	#N/A	#N/A	#N/A
TRINITY_DN22370_c1_g1_i15	HOS1_ARATH	#N/A	#N/A	#N/A	#N/A	9.20	0.57	1.5E-10	1.0E-07	#N/A	#N/A	#N/A	#N/A
TRINITY_DN16534_c1_g1_i5	ZCD_CROSA	#N/A	#N/A	#N/A	#N/A	9.25	0.61	5.9E-06	1.5E-03	#N/A	#N/A	#N/A	#N/A
TRINITY_DN18834_c0_g1_i12	RHIE_DICD3	#N/A	#N/A	#N/A	#N/A	9.29	0.65	1.7E-10	1.2E-07	#N/A	#N/A	#N/A	#N/A

DET	Subject id	c9TR vs c7NT				c7TR vs c7NT				c7TR vs c9TR			
		Log_2	Log_2CPM	PValue	19FDR	Log_2	Log_2CPM	PValue	19FDR	Log_2	Log_2CPM	PValue	19FDR
TRINITY_DN15973_c0_g3_i1	FUT1_ARATH	#N/A	#N/A	#N/A	#N/A	9.34	0.70	2.7E-11	2.2E-08	#N/A	#N/A	#N/A	#N/A
TRINITY_DN16782_c0_g1_i7	Y4141_ARATH	#N/A	#N/A	#N/A	#N/A	9.52	0.87	9.6E-05	1.6E-02	#N/A	#N/A	#N/A	#N/A
TRINITY_DN15234_c0_g3_i9	C7A14_ARATH	#N/A	#N/A	#N/A	#N/A	9.55	3.45	3.5E-04	4.9E-02	#N/A	#N/A	#N/A	#N/A
TRINITY_DN18225_c1_g2_i3	RAE1C_ARATH	#N/A	#N/A	#N/A	#N/A	9.56	0.91	9.4E-05	1.6E-02	#N/A	#N/A	#N/A	#N/A
TRINITY_DN18350_c1_g2_i2	B2_DAUCA	#N/A	#N/A	#N/A	#N/A	9.57	0.92	3.5E-06	9.7E-04	#N/A	#N/A	#N/A	#N/A
TRINITY_DN22075_c0_g1_i11	LORF2_HUMAN	#N/A	#N/A	#N/A	#N/A	9.58	0.93	2.5E-13	2.6E-10	#N/A	#N/A	#N/A	#N/A
TRINITY_DN20106_c0_g1_i7	C3H25_ORYSJ	#N/A	#N/A	#N/A	#N/A	9.72	1.06	2.2E-08	9.8E-06	#N/A	#N/A	#N/A	#N/A
TRINITY_DN21575_c1_g2_i2	SPX4_ORYSJ	#N/A	#N/A	#N/A	#N/A	9.79	1.13	2.8E-05	6.0E-03	#N/A	#N/A	#N/A	#N/A
TRINITY_DN22062_c0_g1_i16	MIRO1_ARATH	#N/A	#N/A	#N/A	#N/A	9.83	1.17	8.9E-07	2.8E-04	#N/A	#N/A	#N/A	#N/A
TRINITY_DN19342_c2_g1_i18	AGO11_ORYSJ	#N/A	#N/A	#N/A	#N/A	9.83	1.17	3.6E-14	4.5E-11	#N/A	#N/A	#N/A	#N/A
TRINITY_DN19405_c1_g2_i1	K1468_XENTR	#N/A	#N/A	#N/A	#N/A	9.88	1.22	5.8E-17	1.0E-13	#N/A	#N/A	#N/A	#N/A
TRINITY_DN16503_c2_g1_i8	CINV1_ORYSJ	#N/A	#N/A	#N/A	#N/A	10.19	1.52	6.2E-11	4.6E-08	#N/A	#N/A	#N/A	#N/A
TRINITY_DN19418_c1_g1_i14	PSAA_SORBI	#N/A	#N/A	#N/A	#N/A	10.24	1.57	6.5E-09	3.2E-06	#N/A	#N/A	#N/A	#N/A
TRINITY_DN21992_c0_g6_i4	ALFC3_ARATH	#N/A	#N/A	#N/A	#N/A	10.79	2.11	1.4E-17	2.8E-14	#N/A	#N/A	#N/A	#N/A
TRINITY_DN22114_c0_g1_i18	TRH22_ORYSJ	#N/A	#N/A	#N/A	#N/A	11.03	2.35	1.0E-05	2.4E-03	#N/A	#N/A	#N/A	#N/A
TRINITY_DN22700_c4_g3_i13	HFA2E_ORYSJ	#N/A	#N/A	#N/A	#N/A	11.06	2.37	3.6E-06	9.8E-04	#N/A	#N/A	#N/A	#N/A
TRINITY_DN22700_c4_g3_i8	HFA2E_ORYSJ	#N/A	#N/A	#N/A	#N/A	11.29	2.60	2.9E-09	1.6E-06	#N/A	#N/A	#N/A	#N/A
TRINITY_DN20664_c0_g1_i4	ASPR1_ORYSJ	#N/A	#N/A	#N/A	#N/A	11.46	2.77	1.5E-05	3.3E-03	#N/A	#N/A	#N/A	#N/A
TRINITY_DN19979_c0_g1_i10	UBC2_WHEAT	#N/A	#N/A	#N/A	#N/A	11.83	3.13	8.8E-09	4.2E-06	#N/A	#N/A	#N/A	#N/A
TRINITY_DN16782_c0_g1_i4	Y4141_ARATH	#N/A	#N/A	#N/A	#N/A	12.57	3.87	2.1E-08	9.6E-06	#N/A	#N/A	#N/A	#N/A
TRINITY_DN15786_c1_g2_i3	U2AFA_ORYSJ	13.54	4.82	4.3E-38	5.4E-35	#N/A	#N/A	#N/A	#N/A	-13.56	4.81	1.0E-36	1.9E-33
TRINITY_DN16678_c0_g1_i11	RL7A_ORYSJ	13.36	4.64	1.6E-69	9.8E-66	#N/A	#N/A	#N/A	#N/A	-13.39	4.63	8.0E-64	1.1E-59
TRINITY_DN17780_c0_g5_i3	CATA2_MAIZE	13.17	4.45	1.9E-56	5.7E-53	#N/A	#N/A	#N/A	#N/A	-13.20	4.45	1.8E-53	1.0E-49
TRINITY_DN21779_c1_g1_i12	SUT1_ORYSJ	13.14	4.42	1.0E-29	7.9E-27	#N/A	#N/A	#N/A	#N/A	-13.17	4.42	9.4E-29	9.2E-26
TRINITY_DN17134_c1_g3_i2	GSHRP_TOBAC	12.77	4.05	3.4E-58	1.2E-54	#N/A	#N/A	#N/A	#N/A	-12.80	4.05	5.8E-54	3.9E-50
TRINITY_DN15117_c1_g1_i2	IBMP_CAMVS	12.60	3.88	9.1E-68	4.7E-64	#N/A	#N/A	#N/A	#N/A	-12.62	3.88	2.3E-61	2.2E-57
TRINITY_DN19270_c1_g2_i9	GSTUH_ARATH	12.22	3.51	4.5E-58	1.5E-54	#N/A	#N/A	#N/A	#N/A	-12.25	3.50	1.0E-52	5.2E-49
TRINITY_DN16098_c3_g2_i5	SDAF2_ARATH	12.18	3.47	1.1E-57	3.6E-54	#N/A	#N/A	#N/A	#N/A	-12.21	3.46	2.1E-52	1.0E-48
TRINITY_DN19405_c1_g3_i2	RGA3_SOLBU	12.17	3.46	7.6E-32	7.3E-29	#N/A	#N/A	#N/A	#N/A	-12.20	3.45	1.6E-30	1.8E-27
TRINITY_DN15786_c1_g2_i6	U2AFA_ORYSJ	12.12	3.40	1.2E-41	1.9E-38	#N/A	#N/A	#N/A	#N/A	-12.14	3.40	1.3E-40	3.3E-37
TRINITY_DN18257_c0_g1_i1	IBR3_ARATH	11.96	3.25	9.2E-53	2.4E-49	#N/A	#N/A	#N/A	#N/A	-11.99	3.25	2.1E-48	7.1E-45
TRINITY_DN16778_c0_g1_i4	RP8L3_ARATH	11.94	3.23	2.8E-53	7.9E-50	#N/A	#N/A	#N/A	#N/A	-11.97	3.22	8.9E-49	3.3E-45
TRINITY_DN20658_c0_g2_i15	YTHD2_BOVIN	11.75	3.04	6.8E-39	9.0E-36	#N/A	#N/A	#N/A	#N/A	-11.78	3.04	4.8E-38	1.1E-34
TRINITY_DN21575_c1_g1_i11	DRP2B_ARATH	11.67	2.97	4.4E-35	5.0E-32	#N/A	#N/A	#N/A	#N/A	-11.70	2.96	1.2E-34	1.9E-31
TRINITY_DN16378_c1_g2_i5	TIRC_ORYSJ	11.60	2.90	1.0E-37	1.2E-34	#N/A	#N/A	#N/A	#N/A	-11.63	2.89	7.8E-37	1.5E-33
TRINITY_DN18582_c0_g1_i3	IOJAP_MAIZE	11.55	2.85	9.7E-27	6.1E-24	#N/A	#N/A	#N/A	#N/A	-11.58	2.84	7.6E-26	6.2E-23
TRINITY_DN14792_c0_g1_i3	TIP11_MAIZE	11.51	2.80	4.3E-20	1.6E-17	#N/A	#N/A	#N/A	#N/A	-11.54	2.79	1.3E-19	7.0E-17
TRINITY_DN17857_c0_g1_i9	TCPZA_ARATH	11.29	2.59	6.2E-39	8.4E-36	#N/A	#N/A	#N/A	#N/A	-11.32	2.59	1.5E-37	3.0E-34
TRINITY_DN19787_c0_g2_i20	NAATA_HORVU	11.24	2.53	1.6E-20	6.1E-18	#N/A	#N/A	#N/A	#N/A	-11.26	2.53	5.6E-20	3.0E-17
TRINITY_DN19974_c0_g1_i4	VAR3_ARATH	11.22	2.52	2.8E-19	9.6E-17	#N/A	#N/A	#N/A	#N/A	-11.25	2.51	8.2E-19	3.9E-16
TRINITY_DN16778_c0_g1_i2	RP8L3_ARATH	11.22	2.52	3.5E-32	3.4E-29	#N/A	#N/A	#N/A	#N/A	-11.25	2.51	1.3E-31	1.7E-28
TRINITY_DN18152_c1_g1_i8	ATK1_ARATH	11.08	2.38	1.6E-31	1.5E-28	#N/A	#N/A	#N/A	#N/A	-11.11	2.37	8.4E-31	9.9E-28
TRINITY_DN17852_c1_g1_i4	HIS1_ORYSJ	6.08	2.33	9.9E-05	4.6E-03	#N/A	#N/A	#N/A	#N/A	-11.04	2.30	3.8E-20	2.1E-17
TRINITY_DN20297_c1_g1_i3	P2C33_ORYSJ	10.99	2.29	7.4E-34	8.1E-31	#N/A	#N/A	#N/A	#N/A	-11.02	2.29	1.2E-32	1.7E-29
TRINITY_DN18787_c0_g1_i3	PRNL1_ARATH	10.96	2.27	3.9E-30	3.2E-27	#N/A	#N/A	#N/A	#N/A	-10.99	2.26	3.1E-28	2.8E-25
TRINITY_DN19354_c0_g1_i1	TBC5A_DICDI	10.95	2.25	6.0E-29	4.6E-26	#N/A	#N/A	#N/A	#N/A	-10.97	2.24	3.1E-27	2.6E-24
TRINITY_DN20634_c0_g1_i10	PP312_ARATH	10.94	2.24	3.6E-30	3.1E-27	#N/A	#N/A	#N/A	#N/A	-10.96	2.23	1.9E-29	2.0E-26
TRINITY_DN21479_c0_g4_i20	ZDHC2_ARATH	10.83	2.13	1.2E-30	1.1E-27	#N/A	#N/A	#N/A	#N/A	-10.86	2.13	1.3E-29	1.4E-26
TRINITY_DN15511_c0_g1_i5	PP279_ARATH	10.81	2.12	6.3E-29	4.7E-26	#N/A	#N/A	#N/A	#N/A	-10.84	2.11	5.1E-27	4.3E-24
TRINITY_DN18152_c1_g1_i6	ATK1_ARATH	10.80	2.10	3.2E-16	8.5E-14	#N/A	#N/A	#N/A	#N/A	-10.83	2.10	1.0E-15	3.6E-13
TRINITY_DN17252_c2_g3_i7	PFD2_ARATH	10.64	1.95	1.1E-28	7.8E-26	#N/A	#N/A	#N/A	#N/A	-10.67	1.94	6.1E-28	5.4E-25
TRINITY_DN15869_c0_g3_i3	NAC68_ORYSJ	8.59	1.86	1.5E-23	7.3E-21	#N/A	#N/A	#N/A	#N/A	-10.58	1.85	1.6E-23	1.1E-20
TRINITY_DN19331_c0_g1_i4	CYP23_ARATH	10.53	1.84	9.7E-26	5.5E-23	#N/A	#N/A	#N/A	#N/A	-10.55	1.83	2.6E-24	1.9E-21
TRINITY_DN18152_c1_g1_i22	ATK1_ARATH	10.50	1.82	6.0E-04	2.1E-02	#N/A	#N/A	#N/A	#N/A	-10.53	1.81	6.0E-04	3.8E-02
TRINITY_DN20225_c0_g1_i2	MAG2L_ARATH	10.48	1.79	9.7E-26	5.5E-23	#N/A	#N/A	#N/A	#N/A	-10.51	1.78	2.5E-24	1.8E-21
TRINITY_DN15283_c1_g1_i8	RSLE2_ORYSJ	10.48	1.79	8.3E-26	4.8E-23	#N/A	#N/A	#N/A	#N/A	-10.50	1.78	2.5E-24	1.8E-21
TRINITY_DN19405_c1_g3_i1	TAO1_ARATH	10.43	1.75	1.8E-22	8.1E-20	#N/A	#N/A	#N/A	#N/A	-10.46	1.74	1.7E-21	1.0E-18
TRINITY_DN21841_c0_g2_i16	SIP1_ORYSJ	10.41	1.72	5.1E-26	3.0E-23	#N/A	#N/A	#N/A	#N/A	-10.44	1.72	1.5E-24	1.1E-21
TRINITY_DN15470_c0_g1_i1	PAP6_ARATH	9.48	4.40	1.1E-51	2.6E-48	#N/A	#N/A	#N/A	#N/A	-10.42	4.40	2.4E-50	9.8E-47
TRINITY_DN18924_c0_g1_i3	C86B1_ARATH	10.35	1.67	2.7E-25	1.4E-22	#N/A	#N/A	#N/A	#N/A	-10.38	1.66	6.1E-24	4.4E-21
TRINITY_DN19733_c0_g3_i5	BAM1_ARATH	10.29	1.61	6.5E-15	1.5E-12	#N/A	#N/A	#N/A	#N/A	-10.32	1.60	1.4E-14	4.4E-12

DET	Subject id	c9TR vs c7NT				c7TR vs c7NT				c7TR vs c9TR			
		Log_2	Log_2CPM	PValue	19FDR	Log_2	Log_2CPM	PValue	19FDR	Log_2	Log_2CPM	PValue	19FDR
TRINITY_DN17131_c0_g1_i1	PUX8_ARATH	10.27	1.59	8.6E-05	4.1E-03	#N/A	#N/A	#N/A	#N/A	-10.30	1.58	8.7E-05	6.8E-03
TRINITY_DN21726_c0_g1_i2	CRTSO_DAUCA	10.23	1.55	3.3E-22	1.4E-19	#N/A	#N/A	#N/A	#N/A	-10.26	1.55	3.0E-21	1.8E-18
TRINITY_DN22272_c0_g3_i1	DNAJ6_ARATH	8.09	1.38	5.3E-11	8.1E-09	#N/A	#N/A	#N/A	#N/A	-10.08	1.36	1.0E-11	2.3E-09
TRINITY_DN18418_c0_g1_i15	CHS1_MAIZE	10.00	1.33	6.6E-22	2.8E-19	#N/A	#N/A	#N/A	#N/A	-10.03	1.32	6.3E-21	3.5E-18
TRINITY_DN21770_c0_g5_i4	MTNA_MAIZE	9.92	1.25	9.4E-11	1.4E-08	#N/A	#N/A	#N/A	#N/A	-9.95	1.25	1.3E-10	2.5E-08
TRINITY_DN19574_c0_g2_i13	SBT35_ARATH	9.86	1.19	1.8E-19	6.3E-17	#N/A	#N/A	#N/A	#N/A	-9.89	1.18	8.9E-19	4.2E-16
TRINITY_DN21922_c0_g4_i1	BLH6_ARATH	9.85	1.19	5.3E-16	1.4E-13	#N/A	#N/A	#N/A	#N/A	-9.88	1.18	1.3E-15	4.6E-13
TRINITY_DN21922_c0_g4_i8	BLH6_ARATH	9.84	1.17	2.4E-18	7.8E-16	#N/A	#N/A	#N/A	#N/A	-9.87	1.16	9.0E-18	3.8E-15
TRINITY_DN20280_c2_g1_i6	YQXC_BACSU	9.80	1.13	9.1E-19	3.0E-16	#N/A	#N/A	#N/A	#N/A	-9.83	1.12	4.0E-18	1.8E-15
TRINITY_DN19574_c0_g2_i5	SBT37_ARATH	9.76	1.10	6.1E-09	7.0E-07	#N/A	#N/A	#N/A	#N/A	-9.79	1.09	7.0E-09	1.1E-06
TRINITY_DN19666_c0_g1_i1	PP301_ARATH	9.74	1.07	6.8E-18	2.1E-15	#N/A	#N/A	#N/A	#N/A	-9.77	1.07	2.3E-17	9.3E-15
TRINITY_DN15158_c1_g5_i1	WAK2_ARATH	9.74	1.07	3.3E-18	1.0E-15	#N/A	#N/A	#N/A	#N/A	-9.77	1.07	1.1E-17	4.8E-15
TRINITY_DN15061_c3_g3_i9	FB302_ARATH	9.73	1.07	2.5E-19	8.8E-17	#N/A	#N/A	#N/A	#N/A	-9.76	1.06	1.2E-18	5.7E-16
TRINITY_DN19270_c1_g2_i4	GSTUH_ARATH	9.67	1.00	6.9E-17	2.0E-14	#N/A	#N/A	#N/A	#N/A	-9.70	1.00	2.1E-16	8.0E-14
TRINITY_DN20177_c1_g3_i15	AMPL2_ORYSJ	9.63	0.97	1.7E-04	7.4E-03	#N/A	#N/A	#N/A	#N/A	-9.66	0.96	1.7E-04	1.2E-02
TRINITY_DN16474_c0_g1_i17	NTPCR_HUMAN	9.50	0.85	2.8E-11	4.5E-09	#N/A	#N/A	#N/A	#N/A	-9.53	0.84	3.7E-11	7.9E-09
TRINITY_DN18240_c2_g3_i3	RCL1_ARATH	9.48	0.83	1.2E-14	2.7E-12	#N/A	#N/A	#N/A	#N/A	-9.51	0.82	2.3E-14	7.1E-12
TRINITY_DN17823_c1_g1_i10	PP384_ARATH	9.47	0.81	2.3E-17	6.7E-15	#N/A	#N/A	#N/A	#N/A	-9.50	0.81	6.4E-17	2.6E-14
TRINITY_DN15037_c0_g1_i7	ELOC_RAT	6.06	0.78	2.0E-09	2.4E-07	#N/A	#N/A	#N/A	#N/A	-9.44	0.75	1.7E-15	5.7E-13
TRINITY_DN18204_c0_g2_i9	GAM1_ORYSI	9.23	0.59	1.8E-08	1.9E-06	#N/A	#N/A	#N/A	#N/A	-9.26	0.58	1.9E-08	2.8E-06
TRINITY_DN17134_c1_g3_i3	GSHRP_TOBAC	9.14	0.51	4.7E-14	1.0E-11	#N/A	#N/A	#N/A	#N/A	-9.17	0.50	7.6E-14	2.2E-11
TRINITY_DN19974_c0_g1_i2	VAR3_ARATH	9.14	0.51	1.9E-11	3.1E-09	#N/A	#N/A	#N/A	#N/A	-9.17	0.50	2.2E-11	4.9E-09
TRINITY_DN15817_c0_g2_i14	MEL1_ORYSJ	9.13	0.49	4.0E-13	7.8E-11	#N/A	#N/A	#N/A	#N/A	-9.15	0.48	5.7E-13	1.5E-10
TRINITY_DN18924_c0_g1_i5	C86B1_ARATH	9.12	0.49	5.3E-13	1.0E-10	#N/A	#N/A	#N/A	#N/A	-9.15	0.48	7.3E-13	1.9E-10
TRINITY_DN22739_c2_g3_i1	CIPK2_ORYSJ	9.09	0.46	2.5E-15	6.3E-13	#N/A	#N/A	#N/A	#N/A	-9.12	0.45	4.8E-15	1.6E-12
TRINITY_DN22739_c2_g3_i2	CIPK2_ORYSJ	9.09	0.45	4.4E-14	9.7E-12	#N/A	#N/A	#N/A	#N/A	-9.12	0.44	7.2E-14	2.1E-11
TRINITY_DN15665_c0_g1_i1	RH56_ARATH	9.01	0.38	9.4E-10	1.2E-07	#N/A	#N/A	#N/A	#N/A	-9.03	0.37	1.1E-09	1.9E-07
TRINITY_DN18257_c0_g1_i6	IBR3_ARATH	9.00	0.37	5.9E-15	1.4E-12	#N/A	#N/A	#N/A	#N/A	-9.03	0.36	1.3E-14	4.0E-12
TRINITY_DN21726_c0_g2_i10	CRTSO_ARATH	5.22	0.41	6.3E-06	4.0E-04	#N/A	#N/A	#N/A	#N/A	-9.03	0.36	7.4E-14	2.1E-11
TRINITY_DN16778_c0_g1_i5	RP8L3_ARATH	8.94	0.31	7.8E-14	1.6E-11	#N/A	#N/A	#N/A	#N/A	-8.96	0.30	1.6E-13	4.4E-11
TRINITY_DN18001_c0_g2_i4	HVA22_HORVU	5.67	0.32	6.6E-12	1.1E-09	#N/A	#N/A	#N/A	#N/A	-8.95	0.29	5.3E-14	1.6E-11
TRINITY_DN20708_c0_g1_i5	CYB6_MAIZE	8.90	0.28	1.0E-06	7.8E-05	#N/A	#N/A	#N/A	#N/A	-8.94	0.27	1.1E-06	1.2E-04
TRINITY_DN13129_c0_g1_i2	NTPR_ENTHA	8.90	0.28	1.1E-13	2.4E-11	#N/A	#N/A	#N/A	#N/A	-8.93	0.27	2.4E-13	6.5E-11
TRINITY_DN20806_c0_g1_i10	BGAL5_ORYSJ	5.25	0.29	2.6E-07	2.3E-05	#N/A	#N/A	#N/A	#N/A	-8.91	0.25	3.4E-13	9.1E-11
TRINITY_DN9774_c0_g1_i1	TF29_SCHPO	8.88	0.26	7.4E-14	1.6E-11	#N/A	#N/A	#N/A	#N/A	-8.91	0.24	1.6E-13	4.5E-11
TRINITY_DN15655_c2_g2_i7	BP73_ORYSJ	8.86	0.25	3.8E-10	5.1E-08	#N/A	#N/A	#N/A	#N/A	-8.89	0.24	5.3E-10	9.6E-08
TRINITY_DN20766_c1_g1_i1	RSSA_VITVI	8.78	0.17	7.5E-12	1.3E-09	#N/A	#N/A	#N/A	#N/A	-8.81	0.16	1.3E-11	3.0E-09
TRINITY_DN17780_c0_g5_i6	CATA2_MAIZE	8.77	0.15	1.1E-12	2.0E-10	#N/A	#N/A	#N/A	#N/A	-8.80	0.14	2.4E-12	5.9E-10
TRINITY_DN21241_c1_g1_i7	THO4B_ARATH	8.76	0.14	2.7E-11	4.3E-09	#N/A	#N/A	#N/A	#N/A	-8.79	0.13	4.7E-11	9.8E-09
TRINITY_DN17704_c1_g1_i2	NAC94_ARATH	8.74	0.13	1.2E-12	2.2E-10	#N/A	#N/A	#N/A	#N/A	-8.77	0.12	2.7E-12	6.4E-10
TRINITY_DN19319_c0_g3_i4	MANA_CANEN	8.64	0.04	2.6E-11	4.1E-09	#N/A	#N/A	#N/A	#N/A	-8.67	0.03	5.1E-11	1.1E-08
TRINITY_DN17392_c0_g2_i5	AGUA_ARATH	8.59	-0.01	5.9E-07	4.7E-05	#N/A	#N/A	#N/A	#N/A	-8.62	-0.02	6.7E-07	7.5E-05
TRINITY_DN19331_c0_g1_i11	CYP23_ARATH	8.58	-0.02	3.5E-11	5.4E-09	#N/A	#N/A	#N/A	#N/A	-8.61	-0.03	8.0E-11	1.6E-08
TRINITY_DN19809_c0_g2_i3	VIL1_ARATH	8.56	-0.04	2.3E-08	2.4E-06	#N/A	#N/A	#N/A	#N/A	-8.59	-0.05	3.0E-08	4.3E-06
TRINITY_DN15885_c0_g3_i2	PP135_ARATH	8.51	-0.08	5.4E-09	6.2E-07	#N/A	#N/A	#N/A	#N/A	-8.54	-0.10	8.1E-09	1.3E-06
TRINITY_DN18713_c0_g1_i7	SNM1_ARATH	8.50	-0.09	3.6E-08	3.7E-06	#N/A	#N/A	#N/A	#N/A	-8.53	-0.10	4.8E-08	6.7E-06
TRINITY_DN19574_c0_g2_i17	SBT37_ARATH	8.50	-0.09	6.3E-05	3.1E-03	#N/A	#N/A	#N/A	#N/A	-8.53	-0.10	6.4E-05	5.2E-03
TRINITY_DN20882_c0_g2_i9	FRS5_ARATH	8.47	-0.12	2.0E-06	1.4E-04	#N/A	#N/A	#N/A	#N/A	-8.50	-0.13	2.3E-06	2.4E-04
TRINITY_DN19808_c1_g2_i4	GDL83_ARATH	8.39	-0.19	1.4E-07	1.3E-05	#N/A	#N/A	#N/A	#N/A	-8.42	-0.21	1.8E-07	2.3E-05
TRINITY_DN11476_c0_g1_i1	SERK1_ARATH	8.39	-0.20	7.3E-10	9.3E-08	#N/A	#N/A	#N/A	#N/A	-8.41	-0.21	1.4E-09	2.4E-07
TRINITY_DN15737_c0_g2_i2	TAN1_MAIZE	6.36	-0.25	3.6E-07	3.0E-05	#N/A	#N/A	#N/A	#N/A	-8.34	-0.28	8.3E-08	1.1E-05
TRINITY_DN15817_c0_g2_i2	MEL1_ORYSJ	8.28	-0.29	1.6E-08	1.7E-06	#N/A	#N/A	#N/A	#N/A	-8.31	-0.30	2.3E-08	3.4E-06
TRINITY_DN16621_c0_g1_i1	LRP1B_ARATH	8.21	-0.35	3.0E-05	1.6E-03	#N/A	#N/A	#N/A	#N/A	-8.24	-0.36	3.0E-05	2.7E-03
TRINITY_DN16849_c0_g3_i6	14334_ORYSJ	11.44	2.74	9.2E-42	1.4E-38	#N/A	#N/A	#N/A	#N/A	-8.21	2.74	1.1E-31	1.4E-28
TRINITY_DN22272_c0_g3_i9	DNAJ6_ARATH	8.87	4.31	3.3E-26	2.0E-23	#N/A	#N/A	#N/A	#N/A	-8.18	4.31	5.3E-43	1.5E-39
TRINITY_DN14891_c0_g2_i3	DTX48_ARATH	8.13	-0.43	3.1E-07	2.7E-05	#N/A	#N/A	#N/A	#N/A	-8.16	-0.44	4.1E-07	4.8E-05
TRINITY_DN19125_c0_g1_i10	TRA1_MAIZE	4.87	-0.40	5.4E-05	2.7E-03	#N/A	#N/A	#N/A	#N/A	-8.15	-0.45	6.0E-08	8.3E-06
TRINITY_DN14792_c0_g1_i6	TIP11_MAIZE	7.96	-0.58	2.7E-07	2.3E-05	#N/A	#N/A	#N/A	#N/A	-7.98	-0.60	3.6E-07	4.3E-05
TRINITY_DN16408_c0_g1_i8	EXLA1_ORYSJ	7.95	-0.59	6.6E-07	5.2E-05	#N/A	#N/A	#N/A	#N/A	-7.98	-0.60	7.7E-07	8.5E-05
TRINITY_DN20539_c0_g1_i12	TET2_ARATH	7.88	-0.65	2.7E-07	2.3E-05	#N/A	#N/A	#N/A	#N/A	-7.91	-0.66	3.5E-07	4.3E-05
TRINITY_DN19433_c0_g1_i1	TCEB2_TULGE	7.75	-0.76	4.7E-07	3.8E-05	#N/A	#N/A	#N/A	#N/A	-7.78	-0.78	6.1E-07	6.9E-05

DET	Subject id	c9TR vs c7NT				c7TR vs c7NT				c7TR vs c9TR			
		Log_2	Log_2CPM	PValue	19FDR	Log_2	Log_2CPM	PValue	19FDR	Log_2	Log_2CPM	PValue	19FDR
TRINITY_DN19958_c5_g1_i4	CTPA2_ARATH	7.60	-0.89	1.6E-04	6.9E-03	#N/A	#N/A	#N/A	#N/A	-7.63	-0.91	1.6E-04	1.2E-02
TRINITY_DN18611_c0_g3_i3	CKS1_ORYSJ	9.50	0.84	1.8E-15	4.4E-13	#N/A	#N/A	#N/A	#N/A	-7.60	0.84	3.8E-14	1.1E-11
TRINITY_DN18001_c0_g2_i1	HVA22_HORVU	10.88	2.18	6.0E-30	4.9E-27	#N/A	#N/A	#N/A	#N/A	-7.26	2.18	2.4E-23	1.7E-20
TRINITY_DN18713_c0_g1_i5	SNM1_ARATH	10.31	1.63	1.4E-09	1.7E-07	#N/A	#N/A	#N/A	#N/A	-7.04	1.63	2.3E-06	2.4E-04
TRINITY_DN20732_c0_g1_i18	NIP22_MAIZE	6.07	4.85	1.2E-35	1.4E-32	#N/A	#N/A	#N/A	#N/A	-6.83	4.84	4.4E-40	1.1E-36
TRINITY_DN18001_c0_g2_i3	HVA22_HORVU	13.21	4.49	4.1E-83	4.0E-79	#N/A	#N/A	#N/A	#N/A	-6.69	4.50	3.1E-58	2.6E-54
TRINITY_DN18001_c0_g2_i2	HVA22_HORVU	6.53	2.73	2.8E-31	2.6E-28	#N/A	#N/A	#N/A	#N/A	-6.68	2.73	8.7E-22	5.3E-19
TRINITY_DN19374_c1_g3_i9	CER7_ARATH	1.94	2.89	4.0E-04	1.5E-02	#N/A	#N/A	#N/A	#N/A	-6.20	2.58	1.3E-04	9.4E-03
TRINITY_DN16378_c1_g2_i7	TIRC_ORYSJ	8.17	4.44	1.2E-07	1.2E-05	#N/A	#N/A	#N/A	#N/A	-5.98	4.45	1.9E-05	1.7E-03
TRINITY_DN21801_c0_g1_i15	ACOC_ORYSJ	4.61	-0.32	4.4E-04	1.6E-02	#N/A	#N/A	#N/A	#N/A	-5.54	-0.35	5.6E-06	5.5E-04
TRINITY_DN14522_c0_g1_i6	HA22E_ARATH	11.91	3.20	9.8E-51	2.3E-47	#N/A	#N/A	#N/A	#N/A	-5.41	3.22	9.2E-05	7.1E-03
TRINITY_DN15312_c1_g2_i1	TLP_ORYSJ	8.87	0.25	1.4E-12	2.6E-10	#N/A	#N/A	#N/A	#N/A	-5.30	0.26	3.6E-09	6.0E-07
TRINITY_DN16849_c0_g3_i2	14332_ORYSJ	7.94	-0.59	4.8E-06	3.1E-04	#N/A	#N/A	#N/A	#N/A	-5.29	-0.58	1.7E-04	1.2E-02
TRINITY_DN15999_c0_g2_i2	AHL10_ARATH	4.67	-0.59	5.8E-05	2.9E-03	#N/A	#N/A	#N/A	#N/A	-5.16	-0.61	1.3E-04	9.7E-03
TRINITY_DN16873_c0_g2_i10	U83A1_ARATH	2.23	0.88	5.6E-04	1.9E-02	#N/A	#N/A	#N/A	#N/A	-5.07	0.65	3.4E-07	4.1E-05
TRINITY_DN19787_c0_g2_i21	NAATA_HORVU	9.01	0.38	1.7E-14	3.8E-12	#N/A	#N/A	#N/A	#N/A	-5.06	0.41	3.3E-05	2.8E-03
TRINITY_DN20756_c3_g6_i4	AKR2_ORYSJ	5.20	1.54	9.3E-12	1.6E-09	#N/A	#N/A	#N/A	#N/A	-4.87	1.55	3.0E-07	3.7E-05
TRINITY_DN21779_c1_g1_i35	SUT1_ORYSJ	4.58	3.35	6.8E-25	3.5E-22	#N/A	#N/A	#N/A	#N/A	-4.47	3.35	9.2E-16	3.4E-13
TRINITY_DN22610_c2_g1_i22	U2A2B_ORYSJ	4.56	-0.30	1.0E-05	6.2E-04	#N/A	#N/A	#N/A	#N/A	-4.30	-0.30	5.8E-06	5.7E-04
TRINITY_DN16837_c0_g1_i16	RH56_ORYSJ	4.57	2.39	1.9E-10	2.7E-08	#N/A	#N/A	#N/A	#N/A	-4.28	2.40	3.8E-09	6.2E-07
TRINITY_DN15528_c0_g1_i6	PR38B_HUMAN	5.90	1.49	1.0E-13	2.2E-11	#N/A	#N/A	#N/A	#N/A	-4.17	1.53	6.9E-05	5.5E-03
TRINITY_DN18737_c0_g1_i3	C70B2_ARATH	4.43	2.00	1.4E-05	8.3E-04	#N/A	#N/A	#N/A	#N/A	-3.98	2.02	4.1E-06	4.2E-04
TRINITY_DN22272_c0_g3_i2	DNAJ6_ARATH	4.48	2.16	1.2E-05	7.4E-04	#N/A	#N/A	#N/A	#N/A	-3.76	2.19	1.3E-07	1.7E-05
TRINITY_DN12439_c0_g1_i2	U83A1_ARATH	3.46	-0.17	1.0E-03	3.2E-02	#N/A	#N/A	#N/A	#N/A	-3.76	-0.20	2.4E-05	2.1E-03
TRINITY_DN21479_c0_g4_i17	ZDHC2_ARATH	4.94	1.17	3.5E-10	4.8E-08	#N/A	#N/A	#N/A	#N/A	-3.52	1.24	4.3E-09	7.0E-07
TRINITY_DN21479_c0_g4_i1	ZDHC2_ARATH	6.27	0.46	1.8E-10	2.6E-08	#N/A	#N/A	#N/A	#N/A	-3.44	0.55	4.6E-07	5.4E-05
TRINITY_DN16922_c0_g2_i5	TPPC2_PIG	3.66	0.74	1.6E-07	1.5E-05	#N/A	#N/A	#N/A	#N/A	-3.42	0.75	2.0E-06	2.1E-04
TRINITY_DN18504_c1_g4_i8	CCC11_ORYSJ	3.10	0.66	7.0E-06	4.3E-04	#N/A	#N/A	#N/A	#N/A	-3.39	0.62	6.7E-07	7.5E-05
TRINITY_DN19974_c0_g1_i5	VAR3_ARATH	5.37	0.89	6.4E-06	4.0E-04	#N/A	#N/A	#N/A	#N/A	-3.35	0.98	2.9E-07	3.6E-05
TRINITY_DN21694_c1_g1_i2	CIPK9_ORYSJ	2.82	3.48	2.6E-15	6.5E-13	#N/A	#N/A	#N/A	#N/A	-3.18	3.43	1.9E-19	9.8E-17
TRINITY_DN21680_c0_g2_i2	NHX2_ARATH	3.08	3.81	1.1E-15	2.9E-13	#N/A	#N/A	#N/A	#N/A	-3.14	3.80	1.4E-18	6.5E-16
TRINITY_DN15531_c0_g2_i3	Y4139_ARATH	3.03	3.67	2.5E-16	6.8E-14	#N/A	#N/A	#N/A	#N/A	-3.00	3.66	2.6E-19	1.3E-16
TRINITY_DN22272_c0_g3_i11	DNAJ6_ARATH	3.28	1.39	7.6E-05	3.6E-03	#N/A	#N/A	#N/A	#N/A	-2.99	1.41	2.1E-04	1.5E-02
TRINITY_DN22491_c1_g2_i9	Y1669_ARATH	2.62	5.44	6.3E-04	2.1E-02	#N/A	#N/A	#N/A	#N/A	-2.95	5.39	1.8E-04	1.3E-02
TRINITY_DN17596_c2_g6_i3	FMDA_SCHPO	3.25	2.71	1.6E-10	2.3E-08	#N/A	#N/A	#N/A	#N/A	-2.95	2.74	1.0E-08	1.5E-06
TRINITY_DN19835_c0_g1_i7	APC4_ARATH	3.15	1.37	5.5E-07	4.4E-05	#N/A	#N/A	#N/A	#N/A	-2.85	1.39	1.3E-04	9.4E-03
TRINITY_DN20700_c0_g3_i4	RD23D_ARATH	2.05	1.88	5.2E-04	1.8E-02	#N/A	#N/A	#N/A	#N/A	-2.71	1.77	1.5E-07	1.9E-05
TRINITY_DN17984_c0_g1_i1	NAC67_ORYSJ	2.78	2.10	3.3E-04	1.3E-02	#N/A	#N/A	#N/A	#N/A	-2.68	2.11	5.4E-04	3.4E-02
TRINITY_DN22184_c0_g2_i7	HNRPQ_RAT	2.99	0.16	3.9E-05	2.0E-03	#N/A	#N/A	#N/A	#N/A	-2.65	0.18	7.4E-05	5.9E-03
TRINITY_DN16316_c0_g2_i5	HBPL1_ARATH	2.67	1.06	7.5E-06	4.7E-04	#N/A	#N/A	#N/A	#N/A	-2.64	1.06	3.3E-07	4.1E-05
TRINITY_DN18737_c0_g1_i2	C70B2_ARATH	2.60	3.24	1.8E-07	1.6E-05	#N/A	#N/A	#N/A	#N/A	-2.62	3.23	9.0E-13	2.2E-10
TRINITY_DN20533_c0_g1_i6	AB22G_ARATH	2.80	3.01	6.0E-12	1.0E-09	#N/A	#N/A	#N/A	#N/A	-2.62	3.03	1.2E-04	8.9E-03
TRINITY_DN20477_c0_g1_i7	POLX_TOBAC	2.24	0.83	1.1E-04	5.2E-03	#N/A	#N/A	#N/A	#N/A	-2.59	0.77	1.3E-05	1.2E-03
TRINITY_DN17398_c1_g1_i8	ZW10_ARATH	3.11	2.36	1.3E-10	1.9E-08	#N/A	#N/A	#N/A	#N/A	-2.42	2.44	3.2E-05	2.8E-03
TRINITY_DN21726_c0_g2_i2	CRTSO_ARATH	2.84	2.39	5.0E-12	8.6E-10	#N/A	#N/A	#N/A	#N/A	-2.35	2.45	1.6E-06	1.7E-04
TRINITY_DN19331_c0_g1_i5	CYP23_ARATH	2.33	0.17	5.3E-04	1.9E-02	#N/A	#N/A	#N/A	#N/A	-2.35	0.15	2.8E-04	1.9E-02
TRINITY_DN15268_c0_g1_i6	AKRC9_ARATH	2.16	1.40	8.5E-07	6.5E-05	#N/A	#N/A	#N/A	#N/A	-2.24	1.38	2.3E-06	2.4E-04
TRINITY_DN20133_c0_g2_i11	CSPLH_MAIZE	2.89	2.99	1.0E-03	3.2E-02	#N/A	#N/A	#N/A	#N/A	-2.19	3.08	1.2E-05	1.1E-03
TRINITY_DN15268_c0_g1_i4	AKRC9_ARATH	1.59	4.16	3.5E-05	1.8E-03	#N/A	#N/A	#N/A	#N/A	-2.15	4.03	3.4E-08	4.8E-06
TRINITY_DN22344_c2_g3_i10	AGT23_ARATH	1.80	2.48	4.6E-04	1.7E-02	#N/A	#N/A	#N/A	#N/A	-2.13	2.41	6.5E-04	4.0E-02
TRINITY_DN22763_c2_g1_i1	C81E1_GLYEC	2.77	1.52	9.0E-06	5.5E-04	#N/A	#N/A	#N/A	#N/A	-2.04	1.63	1.9E-04	1.4E-02
TRINITY_DN15203_c1_g3_i10	DJB13_HUMAN	2.63	1.39	5.8E-05	2.9E-03	#N/A	#N/A	#N/A	#N/A	-1.92	1.50	2.0E-05	1.8E-03
TRINITY_DN19942_c4_g2_i9	M2K2_ARATH	1.14	3.77	1.1E-03	3.3E-02	#N/A	#N/A	#N/A	#N/A	-1.76	3.60	6.3E-05	5.1E-03
TRINITY_DN22823_c3_g4_i1	C72A1_CATRO	1.52	5.19	2.0E-06	1.4E-04	#N/A	#N/A	#N/A	#N/A	-1.75	5.13	1.3E-06	1.4E-04
TRINITY_DN21900_c0_g2_i4	RIBA1_ORYSJ	1.46	2.75	1.5E-05	8.7E-04	#N/A	#N/A	#N/A	#N/A	-1.73	2.68	2.2E-04	1.5E-02
TRINITY_DN13033_c0_g1_i1	SAP17_ORYSJ	1.64	4.05	2.6E-04	1.1E-02	#N/A	#N/A	#N/A	#N/A	-1.70	4.03	3.7E-05	3.2E-03
TRINITY_DN22429_c0_g2_i1	PUR4_ARATH	1.53	3.87	1.1E-05	6.5E-04	#N/A	#N/A	#N/A	#N/A	-1.60	3.84	1.4E-05	1.3E-03
TRINITY_DN20963_c1_g2_i2	PKL_ARATH	1.60	3.90	1.4E-05	8.2E-04	#N/A	#N/A	#N/A	#N/A	-1.60	3.90	9.3E-04	5.5E-02
TRINITY_DN15268_c0_g1_i1	AKRC9_ARATH	1.45	4.38	1.9E-04	8.1E-03	#N/A	#N/A	#N/A	#N/A	-1.59	4.34	4.4E-04	2.9E-02
TRINITY_DN19319_c0_g3_i3	MANA_CANEN	1.68	1.73	1.9E-04	8.1E-03	#N/A	#N/A	#N/A	#N/A	-1.52	1.77	5.7E-04	3.6E-02
TRINITY_DN16869_c1_g3_i10	ACR4_ARATH	1.20	3.10	1.3E-03	3.7E-02	#N/A	#N/A	#N/A	#N/A	-1.52	3.01	2.5E-06	2.6E-04

DET	Subject id	c9TR vs c7NT				c7TR vs c7NT				c7TR vs c9TR			
		Log_2	Log_2CPM	PValue	19FDR	Log_2	Log_2CPM	PValue	19FDR	Log_2	Log_2CPM	PValue	19FDR
TRINITY_DN20400_c0_g1_i9	LMLN_DROPS	0.97	4.52	1.1E-03	3.2E-02	#N/A	#N/A	#N/A	#N/A	-1.35	4.40	4.1E-05	3.4E-03
TRINITY_DN20536_c0_g1_i13	SOL1_ARATH	1.43	2.55	2.3E-04	9.4E-03	#N/A	#N/A	#N/A	#N/A	-1.34	2.57	4.8E-04	3.1E-02
TRINITY_DN18316_c0_g1_i15	ACT2_ORYSJ	1.09	3.31	8.2E-04	2.6E-02	#N/A	#N/A	#N/A	#N/A	-1.32	3.24	2.7E-05	2.4E-03
TRINITY_DN20666_c0_g1_i13	NSUN2_CHICK	1.68	3.19	1.0E-06	7.8E-05	#N/A	#N/A	#N/A	#N/A	-1.30	3.28	4.4E-05	3.7E-03
TRINITY_DN15411_c0_g1_i7	CYSKP_SOLTU	1.28	5.44	6.8E-09	7.7E-07	#N/A	#N/A	#N/A	#N/A	-1.27	5.44	1.5E-08	2.3E-06
TRINITY_DN17643_c0_g1_i6	SUOX_ARATH	1.19	6.20	4.3E-07	3.6E-05	#N/A	#N/A	#N/A	#N/A	-1.23	6.18	7.1E-06	6.9E-04
TRINITY_DN18726_c0_g1_i14	UBP1B_ARATH	0.97	5.00	8.3E-05	3.9E-03	#N/A	#N/A	#N/A	#N/A	-1.19	4.92	2.2E-04	1.5E-02
TRINITY_DN20069_c0_g1_i10	VPS39_MOUSE	1.68	4.26	1.6E-07	1.5E-05	#N/A	#N/A	#N/A	#N/A	-1.18	4.39	2.5E-04	1.7E-02
TRINITY_DN16579_c0_g1_i1	P24B2_ARATH	1.13	4.98	4.0E-07	3.4E-05	#N/A	#N/A	#N/A	#N/A	-1.18	4.96	2.1E-06	2.2E-04
TRINITY_DN18541_c0_g1_i28	AB2C_ARATH	1.27	4.47	9.1E-06	5.6E-04	#N/A	#N/A	#N/A	#N/A	-1.17	4.50	1.6E-04	1.2E-02
TRINITY_DN18692_c1_g3_i3	AGT23_ARATH	0.82	4.63	2.2E-04	9.1E-03	#N/A	#N/A	#N/A	#N/A	-1.11	4.53	1.5E-04	1.1E-02
TRINITY_DN19475_c0_g1_i9	GLK2_ORYSJ	1.26	5.20	2.4E-08	2.5E-06	#N/A	#N/A	#N/A	#N/A	-1.09	5.25	1.2E-05	1.1E-03
TRINITY_DN18132_c1_g1_i9	LTPG1_ARATH	1.60	3.48	1.6E-05	9.0E-04	#N/A	#N/A	#N/A	#N/A	-1.09	3.62	4.2E-04	2.8E-02
TRINITY_DN18654_c3_g6_i4	SUT34_ARATH	1.14	3.78	1.1E-04	5.1E-03	#N/A	#N/A	#N/A	#N/A	-0.95	3.84	8.2E-04	5.0E-02
TRINITY_DN22392_c0_g1_i12	NLP2_ORYSJ	1.20	6.58	1.3E-04	5.8E-03	#N/A	#N/A	#N/A	#N/A	-0.90	6.67	6.4E-04	4.0E-02
TRINITY_DN17113_c1_g1_i4	GSTF1_MAIZE	0.86	5.73	5.6E-05	2.8E-03	#N/A	#N/A	#N/A	#N/A	-0.90	5.71	1.1E-04	8.5E-03
TRINITY_DN15869_c0_g7_i1	NAC68_ORYSJ	0.73	6.05	1.9E-03	5.1E-02	#N/A	#N/A	#N/A	#N/A	-0.87	5.99	7.6E-04	4.6E-02
TRINITY_DN17113_c1_g1_i6	GSTF1_MAIZE	0.95	4.20	1.1E-04	4.9E-03	#N/A	#N/A	#N/A	#N/A	-0.86	4.23	7.1E-04	4.3E-02
TRINITY_DN18653_c0_g4_i4	APX2_ORYSJ	-0.74	5.80	6.4E-04	2.2E-02	#N/A	#N/A	#N/A	#N/A	0.81	5.84	2.2E-04	1.5E-02
TRINITY_DN19095_c0_g1_i15	DJ1B_ARATH	-0.80	5.00	2.1E-03	5.4E-02	#N/A	#N/A	#N/A	#N/A	0.84	5.02	8.0E-04	4.9E-02
TRINITY_DN18653_c0_g4_i12	APX2_ORYSJ	-0.75	8.14	7.0E-04	2.3E-02	#N/A	#N/A	#N/A	#N/A	0.86	8.21	7.5E-04	4.6E-02
TRINITY_DN15690_c1_g1_i5	RBS_MAIZE	-1.81	10.40	1.0E-09	1.3E-07	#N/A	#N/A	#N/A	#N/A	1.01	9.81	7.4E-04	4.5E-02
TRINITY_DN19676_c0_g2_i1	2A5N_ARATH	-1.05	4.41	2.5E-05	1.4E-03	#N/A	#N/A	#N/A	#N/A	1.01	4.38	5.1E-04	3.3E-02
TRINITY_DN22357_c1_g1_i5	CUT1A_ARATH	-1.12	8.88	4.7E-04	1.7E-02	#N/A	#N/A	#N/A	#N/A	1.03	8.81	1.1E-04	8.4E-03
TRINITY_DN20873_c1_g2_i6	CA4_ARATH	-1.16	8.66	7.3E-05	3.5E-03	#N/A	#N/A	#N/A	#N/A	1.03	8.58	1.9E-05	1.7E-03
TRINITY_DN18687_c0_g1_i1	UAP2_SCHPO	-1.05	3.69	1.2E-04	5.4E-03	#N/A	#N/A	#N/A	#N/A	1.05	3.68	1.3E-04	9.6E-03
TRINITY_DN16105_c0_g1_i3	PEPP_ALLCE	-0.98	4.56	8.6E-05	4.1E-03	#N/A	#N/A	#N/A	#N/A	1.07	4.61	2.0E-04	1.4E-02
TRINITY_DN14712_c0_g3_i3	CHMP1_ORYSJ	-0.89	5.00	1.6E-03	4.5E-02	#N/A	#N/A	#N/A	#N/A	1.09	5.13	2.7E-04	1.8E-02
TRINITY_DN18413_c0_g1_i2	Y3544_ARATH	-0.97	4.09	3.3E-05	1.8E-03	#N/A	#N/A	#N/A	#N/A	1.12	4.19	4.8E-05	4.0E-03
TRINITY_DN16532_c1_g1_i5	GPPL2_ARATH	-1.48	8.02	1.0E-04	4.6E-03	#N/A	#N/A	#N/A	#N/A	1.17	7.79	1.2E-06	1.3E-04
TRINITY_DN14765_c1_g1_i1	NIK1_ARATH	-1.10	3.56	3.6E-04	1.3E-02	#N/A	#N/A	#N/A	#N/A	1.18	3.61	3.2E-04	2.2E-02
TRINITY_DN19334_c2_g3_i4	HNRPQ_RAT	-1.32	3.53	4.6E-05	2.4E-03	#N/A	#N/A	#N/A	#N/A	1.20	3.44	3.6E-04	2.4E-02
TRINITY_DN16678_c0_g1_i9	RL7A_ORYSJ	-1.57	6.20	3.8E-06	2.5E-04	#N/A	#N/A	#N/A	#N/A	1.22	5.94	7.4E-04	4.5E-02
TRINITY_DN22028_c0_g1_i13	CNIF1_ARATH	-1.31	5.95	3.0E-04	1.2E-02	#N/A	#N/A	#N/A	#N/A	1.23	5.89	7.0E-06	6.8E-04
TRINITY_DN16842_c0_g1_i2	Y3800_AROAE	-1.25	3.90	2.5E-05	1.4E-03	#N/A	#N/A	#N/A	#N/A	1.27	3.92	4.1E-05	3.4E-03
TRINITY_DN19331_c0_g1_i15	CYP23_ARATH	-1.53	4.14	1.6E-10	2.3E-08	#N/A	#N/A	#N/A	#N/A	1.28	3.95	1.9E-06	2.0E-04
TRINITY_DN18257_c0_g1_i2	IBR3_ARATH	-1.34	4.52	5.8E-05	2.9E-03	#N/A	#N/A	#N/A	#N/A	1.29	4.49	1.8E-04	1.3E-02
TRINITY_DN18346_c0_g3_i1	TKPR1_ARATH	-1.50	2.61	2.6E-06	1.8E-04	#N/A	#N/A	#N/A	#N/A	1.30	2.47	7.0E-04	4.3E-02
TRINITY_DN19834_c0_g3_i2	RS141_MAIZE	-1.80	2.95	8.3E-07	6.4E-05	#N/A	#N/A	#N/A	#N/A	1.30	2.57	4.7E-04	3.0E-02
TRINITY_DN20375_c1_g2_i4	MOSA_MAIZE	-1.48	7.14	1.4E-04	6.1E-03	#N/A	#N/A	#N/A	#N/A	1.36	7.05	2.9E-07	3.6E-05
TRINITY_DN15625_c1_g1_i9	APX8_ORYSJ	-1.94	5.01	1.4E-07	1.3E-05	#N/A	#N/A	#N/A	#N/A	1.39	4.58	1.8E-05	1.6E-03
TRINITY_DN11515_c0_g1_i1	CAPP1_MAIZE	-1.17	4.69	2.5E-04	1.0E-02	#N/A	#N/A	#N/A	#N/A	1.39	4.84	7.7E-05	6.1E-03
TRINITY_DN18582_c0_g1_i6	IOJAP_MAIZE	-1.60	5.13	2.0E-05	1.1E-03	#N/A	#N/A	#N/A	#N/A	1.40	4.97	2.3E-04	1.6E-02
TRINITY_DN22028_c0_g1_i4	CNIF1_ARATH	-1.67	5.24	2.0E-05	1.1E-03	#N/A	#N/A	#N/A	#N/A	1.43	5.06	2.7E-05	2.4E-03
TRINITY_DN21479_c0_g4_i18	ZDHC2_ARATH	-1.76	3.08	4.1E-05	2.1E-03	#N/A	#N/A	#N/A	#N/A	1.44	2.84	1.6E-04	1.2E-02
TRINITY_DN16532_c1_g1_i6	GPPL2_ARATH	-1.64	2.82	9.9E-06	6.0E-04	#N/A	#N/A	#N/A	#N/A	1.47	2.68	2.6E-04	1.8E-02
TRINITY_DN19177_c0_g1_i6	THIC1_ARATH	-1.42	3.88	1.9E-07	1.7E-05	#N/A	#N/A	#N/A	#N/A	1.50	3.93	6.7E-06	6.6E-04
TRINITY_DN15625_c1_g1_i20	APX8_ORYSJ	-1.80	6.36	1.1E-08	1.2E-06	#N/A	#N/A	#N/A	#N/A	1.53	6.15	7.9E-10	1.4E-07
TRINITY_DN16431_c0_g1_i5	PGP1B_ARATH	-1.98	5.57	2.7E-12	4.9E-10	#N/A	#N/A	#N/A	#N/A	1.54	5.23	2.2E-08	3.2E-06
TRINITY_DN15238_c0_g1_i11	PGTB2_HUMAN	-1.59	2.80	5.7E-07	4.6E-05	#N/A	#N/A	#N/A	#N/A	1.56	2.77	4.1E-05	3.5E-03
TRINITY_DN18919_c0_g1_i8	DCE1_ARATH	-1.58	2.85	3.6E-05	1.9E-03	#N/A	#N/A	#N/A	#N/A	1.65	2.89	6.1E-05	5.0E-03
TRINITY_DN18294_c1_g1_i2	TIC21_ARATH	-2.01	5.81	1.3E-11	2.1E-09	#N/A	#N/A	#N/A	#N/A	1.65	5.52	3.0E-08	4.3E-06
TRINITY_DN18257_c0_g1_i4	IBR3_ARATH	-1.83	1.70	7.0E-05	3.4E-03	#N/A	#N/A	#N/A	#N/A	1.70	1.60	4.4E-04	2.9E-02
TRINITY_DN22122_c0_g1_i13	EF1G1_ORYSJ	-2.10	2.47	1.0E-05	6.3E-04	#N/A	#N/A	#N/A	#N/A	1.73	2.18	7.0E-05	5.6E-03
TRINITY_DN22018_c0_g1_i23	SPSA3_ORYSJ	-1.36	2.25	4.8E-04	1.7E-02	#N/A	#N/A	#N/A	#N/A	1.74	2.52	2.2E-05	1.9E-03
TRINITY_DN19354_c0_g1_i7	TBC5A_DICDI	-1.98	4.41	4.5E-06	2.9E-04	#N/A	#N/A	#N/A	#N/A	1.81	4.27	6.1E-05	5.0E-03
TRINITY_DN16810_c0_g1_i3	ALF1_PEA	-1.07	7.18	3.9E-04	1.4E-02	#N/A	#N/A	#N/A	#N/A	1.83	7.73	5.6E-11	1.2E-08
TRINITY_DN20781_c0_g2_i1	FK163_ARATH	-1.81	2.13	1.3E-04	5.9E-03	#N/A	#N/A	#N/A	#N/A	1.92	2.21	2.3E-04	1.6E-02
TRINITY_DN15447_c1_g1_i7	INVA_MAIZE	-1.97	3.61	2.1E-04	8.8E-03	#N/A	#N/A	#N/A	#N/A	2.00	3.62	2.7E-04	1.9E-02
TRINITY_DN21563_c0_g1_i7	RL23A_RAT	-2.70	1.63	1.3E-08	1.4E-06	#N/A	#N/A	#N/A	#N/A	2.11	1.14	2.5E-04	1.7E-02
TRINITY_DN20286_c0_g1_i8	CNR13_MAIZE	-2.21	2.48	2.3E-07	2.0E-05	#N/A	#N/A	#N/A	#N/A	2.11	2.39	1.1E-04	8.1E-03

DET	Subject id	c9TR vs c7NT				c7TR vs c7NT				c7TR vs c9TR			
		Log_2	Log_2CPM	PValue	19FDR	Log_2	Log_2CPM	PValue	19FDR	Log_2	Log_2CPM	PValue	19FDR
TRINITY_DN17643_c0_g1_i3	SUOX_ARATH	-1.94	3.08	2.8E-07	2.4E-05	#N/A	#N/A	#N/A	#N/A	2.14	3.23	1.4E-09	2.4E-07
TRINITY_DN19135_c2_g2_i11	FRI1_MAIZE	-1.62	5.38	4.1E-04	1.5E-02	#N/A	#N/A	#N/A	#N/A	2.20	5.83	1.8E-05	1.6E-03
TRINITY_DN16378_c1_g2_i2	TIRC_ORYSJ	-1.88	5.03	2.8E-15	6.8E-13	#N/A	#N/A	#N/A	#N/A	2.24	5.32	3.8E-19	1.9E-16
TRINITY_DN17187_c0_g3_i5	ACR6_ARATH	-1.93	0.01	9.4E-04	2.9E-02	#N/A	#N/A	#N/A	#N/A	2.38	0.35	3.3E-05	2.9E-03
TRINITY_DN18953_c0_g1_i10	DHAR3_ARATH	-2.59	1.50	5.7E-05	2.8E-03	#N/A	#N/A	#N/A	#N/A	2.48	1.40	1.3E-04	9.5E-03
TRINITY_DN18322_c0_g3_i1	SSL3_ARATH	-2.99	1.35	4.7E-10	6.2E-08	#N/A	#N/A	#N/A	#N/A	2.56	0.99	5.4E-07	6.2E-05
TRINITY_DN18504_c1_g4_i12	CCC11_ORYSJ	-2.50	0.92	1.1E-04	5.2E-03	#N/A	#N/A	#N/A	#N/A	2.77	1.13	3.7E-05	3.1E-03
TRINITY_DN20297_c1_g1_i1	P2C33_ORYSJ	-3.07	3.25	1.9E-19	6.8E-17	#N/A	#N/A	#N/A	#N/A	2.99	3.17	5.7E-18	2.5E-15
TRINITY_DN19195_c2_g2_i5	BAM1_ARATH	-3.07	0.06	9.5E-05	4.4E-03	#N/A	#N/A	#N/A	#N/A	3.05	0.04	1.7E-04	1.2E-02
TRINITY_DN22275_c0_g1_i11	LOX4_ORYSJ	-4.79	3.09	8.3E-08	8.0E-06	#N/A	#N/A	#N/A	#N/A	3.22	1.64	6.7E-05	5.4E-03
TRINITY_DN17902_c0_g1_i2	UBIQP_HORVU	-3.46	0.30	4.2E-06	2.8E-04	#N/A	#N/A	#N/A	#N/A	3.28	0.15	1.2E-04	9.1E-03
TRINITY_DN15411_c0_g1_i5	CYSKP_SOLTU	-3.39	2.47	8.8E-17	2.5E-14	#N/A	#N/A	#N/A	#N/A	3.41	2.49	7.4E-16	2.7E-13
TRINITY_DN21406_c0_g1_i1	SCL28_ARATH	-3.98	0.45	1.2E-09	1.5E-07	#N/A	#N/A	#N/A	#N/A	3.43	-0.04	2.3E-04	1.6E-02
TRINITY_DN15838_c0_g1_i9	ATG10_ARATH	-3.55	-0.19	1.2E-03	3.6E-02	#N/A	#N/A	#N/A	#N/A	3.51	-0.24	6.1E-04	3.8E-02
TRINITY_DN22404_c1_g2_i7	HEI10_ORYSJ	-3.71	-0.12	2.5E-06	1.7E-04	#N/A	#N/A	#N/A	#N/A	3.84	-0.01	2.3E-07	2.9E-05
TRINITY_DN17014_c1_g4_i3	ACA9_ARATH	-4.08	0.13	9.4E-05	4.4E-03	#N/A	#N/A	#N/A	#N/A	3.91	-0.03	8.7E-05	6.8E-03
TRINITY_DN22114_c0_g1_i21	TRH22_ORYSJ	-4.30	2.64	2.2E-13	4.5E-11	#N/A	#N/A	#N/A	#N/A	3.97	2.32	2.3E-04	1.6E-02
TRINITY_DN20852_c0_g1_i1	YIPL1_ARATH	-3.83	0.13	6.1E-07	4.8E-05	#N/A	#N/A	#N/A	#N/A	4.17	0.43	3.1E-07	3.7E-05
TRINITY_DN20279_c1_g1_i3	TRA1_MAIZE	-4.52	0.30	4.4E-11	6.8E-09	#N/A	#N/A	#N/A	#N/A	4.29	0.09	3.6E-09	5.9E-07
TRINITY_DN14522_c0_g1_i1	HA22E_ARATH	-3.71	1.81	9.2E-06	5.6E-04	#N/A	#N/A	#N/A	#N/A	4.57	2.62	1.0E-17	4.3E-15
TRINITY_DN18132_c1_g1_i1	LTPG1_ARATH	-4.43	1.22	1.5E-05	8.5E-04	#N/A	#N/A	#N/A	#N/A	4.78	1.54	3.0E-06	3.1E-04
TRINITY_DN17252_c2_g3_i2	PFD2_ARATH	-4.92	0.40	2.1E-04	8.7E-03	#N/A	#N/A	#N/A	#N/A	4.92	0.40	1.2E-04	9.3E-03
TRINITY_DN16936_c0_g1_i2	ODPB1_ORYSJ	-5.10	3.60	1.8E-29	1.4E-26	#N/A	#N/A	#N/A	#N/A	5.28	3.77	3.8E-35	6.5E-32
TRINITY_DN17643_c0_g1_i4	SUOX_ARATH	-5.47	4.65	2.2E-04	9.2E-03	#N/A	#N/A	#N/A	#N/A	5.53	4.70	2.5E-04	1.7E-02
TRINITY_DN21074_c0_g2_i16	SYP81_ARATH	-5.50	0.27	7.4E-08	7.2E-06	#N/A	#N/A	#N/A	#N/A	5.56	0.32	1.0E-08	1.6E-06
TRINITY_DN16255_c1_g2_i19	AFC2_ARATH	-6.32	0.29	3.4E-07	2.8E-05	#N/A	#N/A	#N/A	#N/A	5.61	-0.36	8.0E-05	6.3E-03
TRINITY_DN17970_c1_g3_i3	PPD3_ARATH	-5.34	0.44	3.7E-09	4.3E-07	#N/A	#N/A	#N/A	#N/A	5.66	0.74	6.3E-13	1.6E-10
TRINITY_DN21179_c0_g2_i15	CDT1A_ARATH	-5.90	0.69	1.2E-13	2.5E-11	#N/A	#N/A	#N/A	#N/A	5.81	0.60	8.9E-13	2.2E-10
TRINITY_DN17066_c1_g1_i3	RS24_ARATH	-7.04	3.02	9.5E-27	6.0E-24	#N/A	#N/A	#N/A	#N/A	5.94	1.95	7.6E-04	4.6E-02
TRINITY_DN20328_c0_g1_i7	HYES_PIG	-6.11	0.53	3.2E-12	5.7E-10	#N/A	#N/A	#N/A	#N/A	6.13	0.54	1.2E-12	2.9E-10
TRINITY_DN16738_c0_g1_i4	XRCC1_ARATH	-6.61	-0.19	1.1E-08	1.2E-06	#N/A	#N/A	#N/A	#N/A	6.14	-0.62	1.3E-06	1.4E-04
TRINITY_DN17050_c0_g2_i2	RK17_TOBAC	-7.19	3.83	9.1E-30	7.3E-27	#N/A	#N/A	#N/A	#N/A	6.34	2.99	8.2E-06	7.9E-04
TRINITY_DN16678_c0_g1_i16	RL7A_ORYSJ	-6.95	0.88	4.7E-12	8.0E-10	#N/A	#N/A	#N/A	#N/A	6.41	0.36	3.1E-08	4.5E-06
TRINITY_DN16858_c0_g1_i4	PP127_ARATH	-6.65	0.58	5.0E-13	9.7E-11	#N/A	#N/A	#N/A	#N/A	6.83	0.75	7.8E-13	2.0E-10
TRINITY_DN18806_c1_g1_i9	FDH1_ORYSJ	-6.57	0.49	1.7E-14	3.8E-12	#N/A	#N/A	#N/A	#N/A	7.41	1.29	1.7E-18	7.7E-16
TRINITY_DN17970_c1_g3_i4	PPD3_ARATH	-7.39	3.85	1.1E-13	2.2E-11	#N/A	#N/A	#N/A	#N/A	7.53	3.98	2.1E-18	9.5E-16
TRINITY_DN21180_c1_g1_i5	UNE12_ARATH	-8.40	-0.20	4.6E-09	5.4E-07	#N/A	#N/A	#N/A	#N/A	7.85	-0.74	6.5E-04	4.0E-02
TRINITY_DN16579_c0_g1_i3	P24B2_ARATH	-7.98	1.09	5.3E-14	1.1E-11	#N/A	#N/A	#N/A	#N/A	7.96	1.06	2.7E-15	9.1E-13
TRINITY_DN20279_c1_g1_i13	TRA1_MAIZE	-8.53	-0.08	1.1E-08	1.2E-06	#N/A	#N/A	#N/A	#N/A	7.99	-0.61	1.2E-07	1.6E-05
TRINITY_DN21101_c0_g1_i3	SYGM1_ARATH	-8.43	-0.18	2.4E-08	2.5E-06	#N/A	#N/A	#N/A	#N/A	8.10	-0.52	6.4E-08	8.8E-06
TRINITY_DN16873_c0_g2_i12	U83A1_ARATH	-7.30	0.47	1.1E-07	1.0E-05	#N/A	#N/A	#N/A	#N/A	8.21	1.32	3.0E-16	1.1E-13
TRINITY_DN16368_c0_g6_i1	YI31B_YEAST	-8.19	1.30	2.5E-18	8.0E-16	#N/A	#N/A	#N/A	#N/A	8.26	1.35	4.5E-20	2.4E-17
TRINITY_DN19762_c0_g1_i2	PTR53_ARATH	-8.00	-0.57	4.1E-06	2.7E-04	#N/A	#N/A	#N/A	#N/A	8.39	-0.25	4.8E-08	6.7E-06
TRINITY_DN18806_c1_g1_i8	FDH1_ORYSJ	-8.49	-0.13	3.1E-10	4.2E-08	#N/A	#N/A	#N/A	#N/A	8.41	-0.23	4.1E-09	6.8E-07
TRINITY_DN15560_c0_g1_i17	NPRT1_ARATH	-8.77	0.14	1.8E-10	2.6E-08	#N/A	#N/A	#N/A	#N/A	8.46	-0.18	2.1E-09	3.6E-07
TRINITY_DN18694_c0_g1_i8	CLCD_ARATH	-8.70	0.07	4.0E-11	6.3E-09	#N/A	#N/A	#N/A	#N/A	8.50	-0.14	4.0E-10	7.4E-08
TRINITY_DN19803_c0_g5_i4	Y5129_ARATH	-9.04	0.39	2.2E-12	4.0E-10	#N/A	#N/A	#N/A	#N/A	8.51	-0.14	1.3E-07	1.7E-05
TRINITY_DN21354_c0_g8_i2	B651B_ARATH	-8.74	0.11	2.3E-10	3.2E-08	#N/A	#N/A	#N/A	#N/A	8.61	-0.04	5.6E-09	8.9E-07
TRINITY_DN19428_c0_g1_i18	VCS_ARATH	-8.58	-0.04	1.2E-10	1.7E-08	#N/A	#N/A	#N/A	#N/A	8.68	0.02	1.9E-11	4.3E-09
TRINITY_DN17066_c1_g1_i8	RS24_ARATH	-10.45	1.75	2.1E-25	1.2E-22	#N/A	#N/A	#N/A	#N/A	8.73	0.06	3.7E-04	2.5E-02
TRINITY_DN19937_c3_g1_i2	THIC_ARATH	-8.84	0.20	4.1E-12	7.1E-10	#N/A	#N/A	#N/A	#N/A	8.73	0.07	6.2E-10	1.1E-07
TRINITY_DN21067_c0_g2_i2	MCM31_MAIZE	-9.28	0.62	1.8E-10	2.5E-08	#N/A	#N/A	#N/A	#N/A	8.79	0.12	8.3E-04	5.0E-02
TRINITY_DN21224_c0_g1_i4	M3K1_ARATH	-8.83	0.19	8.5E-12	1.4E-09	#N/A	#N/A	#N/A	#N/A	8.87	0.20	3.7E-13	9.8E-11
TRINITY_DN17831_c0_g1_i1	XPO4_MOUSE	-8.68	0.05	1.8E-08	1.9E-06	#N/A	#N/A	#N/A	#N/A	8.90	0.23	6.6E-09	1.1E-06
TRINITY_DN16609_c0_g1_i9	YC20L_ARATH	-9.84	1.16	3.1E-20	1.2E-17	#N/A	#N/A	#N/A	#N/A	8.90	0.23	9.8E-07	1.1E-04
TRINITY_DN22404_c1_g2_i1	HEI10_ORYSJ	-8.63	0.01	1.4E-11	2.2E-09	#N/A	#N/A	#N/A	#N/A	9.06	0.38	3.2E-13	8.5E-11
TRINITY_DN19177_c0_g1_i10	THIC1_ARATH	-8.77	0.14	3.8E-10	5.1E-08	#N/A	#N/A	#N/A	#N/A	9.15	0.46	4.5E-15	1.5E-12
TRINITY_DN17821_c0_g1_i1	YTHD2_MOUSE	-9.18	0.53	1.4E-05	8.4E-04	#N/A	#N/A	#N/A	#N/A	9.18	0.49	1.9E-14	5.8E-12
TRINITY_DN22761_c1_g1_i4	VCS_ARATH	-8.76	0.12	1.6E-11	2.6E-09	#N/A	#N/A	#N/A	#N/A	9.18	0.50	7.8E-13	2.0E-10
TRINITY_DN22275_c0_g1_i2	LOX4_ORYSJ	-10.82	2.12	1.4E-10	2.0E-08	#N/A	#N/A	#N/A	#N/A	9.30	0.60	7.2E-13	1.9E-10
TRINITY_DN19354_c0_g1_i5	TBC5A_DICDI	-10.01	1.33	4.1E-22	1.8E-19	#N/A	#N/A	#N/A	#N/A	9.44	0.74	3.3E-15	1.1E-12

DET	Subject id	c9TR vs c7NT				c7TR vs c7NT				c7TR vs c9TR			
		Log_2	Log_2CPM	PValue	19FDR	Log_2	Log_2CPM	PValue	19FDR	Log_2	Log_2CPM	PValue	19FDR
TRINITY_DN18726_c0_g1_i10	UBP1B_ARATH	-9.02	0.37	1.9E-08	2.0E-06	#N/A	#N/A	#N/A	#N/A	9.46	0.76	1.3E-13	3.6E-11
TRINITY_DN20279_c1_g1_i17	TRA1_MAIZE	-9.03	0.38	3.4E-12	5.9E-10	#N/A	#N/A	#N/A	#N/A	9.47	0.77	4.3E-11	9.0E-09
TRINITY_DN19937_c3_g1_i17	THIC_ARATH	-9.21	0.55	1.3E-15	3.3E-13	#N/A	#N/A	#N/A	#N/A	9.54	0.84	4.4E-10	8.2E-08
TRINITY_DN21551_c0_g2_i5	YZR3_ARATH	-9.18	0.52	1.4E-15	3.4E-13	#N/A	#N/A	#N/A	#N/A	9.59	0.88	6.7E-13	1.7E-10
TRINITY_DN17441_c0_g3_i4	YIPL1_ARATH	-8.92	0.28	9.6E-12	1.6E-09	#N/A	#N/A	#N/A	#N/A	9.66	0.95	6.2E-16	2.3E-13
TRINITY_DN21900_c0_g2_i15	RIBA1_ORYSJ	-9.62	0.95	1.0E-16	2.9E-14	#N/A	#N/A	#N/A	#N/A	9.73	1.02	1.7E-15	5.7E-13
TRINITY_DN18748_c0_g4_i1	RH27_ORYSJ	-10.68	1.98	2.6E-30	2.3E-27	#N/A	#N/A	#N/A	#N/A	9.76	1.05	2.4E-05	2.2E-03
TRINITY_DN18859_c0_g1_i12	BI1L_ARATH	-10.51	1.82	1.1E-27	7.5E-25	#N/A	#N/A	#N/A	#N/A	9.81	1.10	5.5E-06	5.5E-04
TRINITY_DN22058_c1_g1_i7	HSP7S_PEA	-10.11	7.24	2.3E-16	6.3E-14	#N/A	#N/A	#N/A	#N/A	9.83	6.96	4.0E-19	2.0E-16
TRINITY_DN15238_c0_g1_i3	PGTB2_HUMAN	-9.53	0.86	4.8E-15	1.2E-12	#N/A	#N/A	#N/A	#N/A	9.87	1.16	1.2E-16	4.7E-14
TRINITY_DN21900_c0_g2_i9	RIBA1_ORYSJ	-8.51	-0.10	2.0E-05	1.1E-03	#N/A	#N/A	#N/A	#N/A	9.90	1.19	8.1E-09	1.3E-06
TRINITY_DN15312_c1_g1_i1	ZEAM_MAIZE	-9.96	1.28	5.2E-14	1.1E-11	#N/A	#N/A	#N/A	#N/A	9.91	1.20	3.1E-14	9.3E-12
TRINITY_DN16679_c1_g1_i11	SAPK3_ORYSJ	-9.37	0.71	1.8E-15	4.4E-13	#N/A	#N/A	#N/A	#N/A	9.96	1.24	7.3E-16	2.7E-13
TRINITY_DN21900_c0_g2_i8	RIBA1_ORYSJ	-10.01	1.32	1.4E-14	3.2E-12	#N/A	#N/A	#N/A	#N/A	10.11	1.39	8.4E-20	4.5E-17
TRINITY_DN15520_c0_g2_i10	GDPD2_ARATH	-10.90	2.20	4.4E-19	1.5E-16	#N/A	#N/A	#N/A	#N/A	10.13	1.40	7.1E-18	3.1E-15
TRINITY_DN16546_c1_g3_i20	FBT7_ARATH	-10.10	1.42	1.0E-03	3.2E-02	#N/A	#N/A	#N/A	#N/A	10.19	1.47	2.5E-14	7.5E-12
TRINITY_DN19340_c1_g6_i6	APX6_ARATH	-10.08	1.39	9.3E-16	2.4E-13	#N/A	#N/A	#N/A	#N/A	10.20	1.48	3.9E-20	2.2E-17
TRINITY_DN18294_c1_g1_i4	TIC21_ARATH	-10.76	2.06	1.8E-25	9.9E-23	#N/A	#N/A	#N/A	#N/A	10.25	1.52	1.6E-15	5.6E-13
TRINITY_DN21551_c0_g2_i10	YZR3_ARATH	-9.20	0.54	2.8E-10	3.9E-08	#N/A	#N/A	#N/A	#N/A	10.27	1.55	1.8E-21	1.1E-18
TRINITY_DN15528_c0_g1_i7	PR38B_MOUSE	-10.28	1.59	9.1E-25	4.7E-22	#N/A	#N/A	#N/A	#N/A	10.28	1.56	1.1E-22	7.7E-20
TRINITY_DN21761_c3_g1_i6	CERK1_ORYSJ	-9.14	0.48	4.1E-14	9.1E-12	#N/A	#N/A	#N/A	#N/A	10.33	1.61	3.8E-22	2.4E-19
TRINITY_DN21535_c0_g1_i12	PP320_ARATH	-10.33	1.63	6.9E-21	2.7E-18	#N/A	#N/A	#N/A	#N/A	10.34	1.62	1.1E-15	3.7E-13
TRINITY_DN21922_c0_g4_i2	BLH6_ARATH	-10.43	1.74	2.3E-25	1.3E-22	#N/A	#N/A	#N/A	#N/A	10.52	1.79	8.1E-22	5.0E-19
TRINITY_DN16869_c1_g3_i13	ACR4_ARATH	-9.48	0.80	9.5E-10	1.2E-07	#N/A	#N/A	#N/A	#N/A	10.55	1.82	2.3E-22	1.5E-19
TRINITY_DN16828_c0_g2_i6	CCR1_ARATH	-10.68	1.98	3.5E-25	1.9E-22	#N/A	#N/A	#N/A	#N/A	10.59	1.85	5.7E-06	5.6E-04
TRINITY_DN15652_c0_g1_i18	Y2309_ARATH	-10.19	4.06	5.9E-47	1.2E-43	#N/A	#N/A	#N/A	#N/A	10.59	4.44	1.4E-67	3.1E-63
TRINITY_DN17011_c0_g1_i12	YTFP_BACSU	-10.49	1.79	2.8E-18	8.9E-16	#N/A	#N/A	#N/A	#N/A	10.65	1.92	5.4E-22	3.4E-19
TRINITY_DN16452_c0_g1_i9	H1_MAIZE	-11.02	2.31	2.3E-27	1.5E-24	#N/A	#N/A	#N/A	#N/A	10.68	1.94	1.6E-07	2.1E-05
TRINITY_DN21761_c3_g1_i5	CERK1_ORYSJ	-11.07	2.37	7.2E-33	7.3E-30	#N/A	#N/A	#N/A	#N/A	10.72	1.99	2.0E-25	1.6E-22
TRINITY_DN21179_c0_g2_i10	CDT1A_ARATH	-10.49	1.80	3.2E-20	1.2E-17	#N/A	#N/A	#N/A	#N/A	10.78	2.05	7.3E-26	6.0E-23
TRINITY_DN16858_c0_g1_i11	PP127_ARATH	-10.69	1.99	1.4E-22	6.5E-20	#N/A	#N/A	#N/A	#N/A	10.85	2.12	1.3E-23	9.6E-21
TRINITY_DN17790_c0_g2_i3	BX9_MAIZE	-11.80	3.09	4.5E-44	7.2E-41	#N/A	#N/A	#N/A	#N/A	10.88	2.14	1.8E-28	1.7E-25
TRINITY_DN15238_c0_g1_i8	PGTB2_HUMAN	-10.55	1.86	7.0E-28	4.9E-25	#N/A	#N/A	#N/A	#N/A	10.89	2.15	8.7E-31	1.0E-27
TRINITY_DN19354_c0_g1_i2	TBC5A_DICDI	-11.25	2.55	1.3E-38	1.6E-35	#N/A	#N/A	#N/A	#N/A	10.94	2.20	1.1E-29	1.2E-26
TRINITY_DN17830_c1_g2_i1	P2C55_ARATH	-11.61	2.90	2.0E-46	3.8E-43	#N/A	#N/A	#N/A	#N/A	10.96	2.22	2.7E-06	2.8E-04
TRINITY_DN22188_c1_g1_i1	IP5PF_ARATH	-11.20	2.50	5.5E-37	6.6E-34	#N/A	#N/A	#N/A	#N/A	11.09	2.36	9.2E-33	1.4E-29
TRINITY_DN21779_c1_g1_i33	SUT1_ORYSJ	-10.44	1.75	1.1E-25	6.3E-23	#N/A	#N/A	#N/A	#N/A	11.14	2.40	1.0E-34	1.7E-31
TRINITY_DN16579_c0_g1_i2	P24B2_ARATH	-11.58	2.87	3.2E-44	5.4E-41	#N/A	#N/A	#N/A	#N/A	11.32	2.58	2.7E-32	3.8E-29
TRINITY_DN20328_c0_g1_i8	HYES_RAT	-11.29	2.59	3.4E-41	4.8E-38	#N/A	#N/A	#N/A	#N/A	11.36	2.62	5.0E-29	5.1E-26
TRINITY_DN21224_c0_g1_i14	M3K1_ARATH	-10.65	1.95	1.2E-21	4.8E-19	#N/A	#N/A	#N/A	#N/A	11.43	2.69	5.9E-40	1.4E-36
TRINITY_DN17024_c1_g2_i2	YI31B_YEAST	-10.64	1.95	2.9E-06	1.9E-04	#N/A	#N/A	#N/A	#N/A	11.60	2.85	2.9E-08	4.2E-06
TRINITY_DN19979_c0_g1_i9	UBC2_WHEAT	-12.93	4.21	2.1E-79	1.8E-75	#N/A	#N/A	#N/A	#N/A	11.71	2.96	5.9E-08	8.2E-06
TRINITY_DN19937_c3_g1_i10	THIC_ARATH	-11.86	3.15	1.1E-45	2.1E-42	#N/A	#N/A	#N/A	#N/A	11.93	3.19	9.5E-32	1.3E-28
TRINITY_DN20533_c0_g1_i5	AB22G_ARATH	-12.23	3.52	1.0E-25	5.9E-23	#N/A	#N/A	#N/A	#N/A	11.94	3.19	1.6E-31	2.0E-28
TRINITY_DN19473_c2_g1_i4	ANTL1_ARATH	-11.65	2.94	4.3E-47	8.7E-44	#N/A	#N/A	#N/A	#N/A	12.02	3.27	2.0E-37	4.0E-34
TRINITY_DN20411_c0_g1_i3	PGKH_WHEAT	-12.40	3.68	5.5E-05	2.8E-03	#N/A	#N/A	#N/A	#N/A	12.06	3.31	8.1E-04	4.9E-02
TRINITY_DN19475_c0_g1_i1	GLK2_ORYSJ	-12.02	3.30	1.2E-56	3.6E-53	#N/A	#N/A	#N/A	#N/A	12.22	3.47	1.4E-48	4.9E-45
TRINITY_DN16449_c0_g5_i2	PPA27_ARATH	-12.34	3.63	2.0E-65	9.1E-62	#N/A	#N/A	#N/A	#N/A	12.32	3.57	1.7E-57	1.2E-53
TRINITY_DN18726_c0_g1_i5	UBP1B_ARATH	-12.44	3.73	2.3E-66	1.1E-62	#N/A	#N/A	#N/A	#N/A	12.55	3.80	3.6E-63	4.0E-59
TRINITY_DN15411_c0_g1_i12	CYSKP_SOLTU	-12.62	3.91	2.5E-62	1.0E-58	#N/A	#N/A	#N/A	#N/A	12.64	3.89	6.1E-65	1.0E-60
TRINITY_DN19827_c2_g2_i4	XRN4_ARATH	-13.00	4.28	2.1E-74	1.4E-70	#N/A	#N/A	#N/A	#N/A	12.77	4.02	6.1E-07	6.9E-05
TRINITY_DN15652_c0_g1_i12	Y2309_ARATH	-14.04	5.32	3.8E-116	1.3E-111	#N/A	#N/A	#N/A	#N/A	14.28	5.53	1.2E-84	7.7E-80
TRINITY_DN21464_c0_g2_i30	VPEA_ARATH	-13.36	4.64	1.3E-03	3.8E-02	#N/A	#N/A	#N/A	#N/A	#N/A	#N/A	#N/A	#N/A
TRINITY_DN16828_c0_g2_i3	TKPR1_ARATH	-12.76	4.05	3.7E-49	8.0E-46	#N/A	#N/A	#N/A	#N/A	#N/A	#N/A	#N/A	#N/A
TRINITY_DN21983_c1_g4_i9	GYRA_ORYSJ	-12.32	3.61	1.7E-30	1.5E-27	#N/A	#N/A	#N/A	#N/A	#N/A	#N/A	#N/A	#N/A
TRINITY_DN18612_c0_g1_i12	PNSB2_ARATH	-11.95	3.23	1.4E-03	4.0E-02	#N/A	#N/A	#N/A	#N/A	#N/A	#N/A	#N/A	#N/A
TRINITY_DN15028_c3_g3_i5	EF1G2_ORYSJ	-11.92	3.21	1.8E-03	4.8E-02	#N/A	#N/A	#N/A	#N/A	#N/A	#N/A	#N/A	#N/A
TRINITY_DN22778_c4_g1_i1	POLX_TOBAC	-11.86	3.15	8.2E-29	6.1E-26	#N/A	#N/A	#N/A	#N/A	#N/A	#N/A	#N/A	#N/A
TRINITY_DN19979_c0_g1_i5	UBC2_WHEAT	-11.34	2.64	3.8E-42	6.0E-39	#N/A	#N/A	#N/A	#N/A	#N/A	#N/A	#N/A	#N/A
TRINITY_DN16154_c0_g1_i16	GPAT1_ARATH	-11.07	2.37	9.0E-07	6.9E-05	#N/A	#N/A	#N/A	#N/A	#N/A	#N/A	#N/A	#N/A
TRINITY_DN22483_c1_g1_i6	EX84B_ARATH	-10.92	2.22	7.2E-27	4.6E-24	#N/A	#N/A	#N/A	#N/A	#N/A	#N/A	#N/A	#N/A

DET	Subject id	c9TR vs c7NT				c7TR vs c7NT				c7TR vs c9TR			
		Log_2	Log_2CPM	PValue	19FDR	Log_2	Log_2CPM	PValue	19FDR	Log_2	Log_2CPM	PValue	19FDR
TRINITY_DN21535_c0_g1_i2	PP320_ARATH	-10.61	1.91	2.2E-14	4.8E-12	#N/A	#N/A	#N/A	#N/A	#N/A	#N/A	#N/A	#N/A
TRINITY_DN18155_c1_g1_i6	CRPM2_ARATH	-10.59	1.89	1.7E-24	8.7E-22	#N/A	#N/A	#N/A	#N/A	#N/A	#N/A	#N/A	#N/A
TRINITY_DN22145_c3_g2_i4	DHRS7_HUMAN	-10.58	1.88	2.3E-20	8.7E-18	#N/A	#N/A	#N/A	#N/A	#N/A	#N/A	#N/A	#N/A
TRINITY_DN16351_c0_g6_i1	CADH_MAIZE	-10.56	1.87	1.2E-03	3.6E-02	#N/A	#N/A	#N/A	#N/A	#N/A	#N/A	#N/A	#N/A
TRINITY_DN22062_c0_g1_i15	MIRO1_ARATH	-10.56	1.87	9.0E-29	6.6E-26	#N/A	#N/A	#N/A	#N/A	#N/A	#N/A	#N/A	#N/A
TRINITY_DN20282_c1_g1_i9	GLE1_ARATH	-10.36	1.67	3.7E-23	1.8E-20	#N/A	#N/A	#N/A	#N/A	#N/A	#N/A	#N/A	#N/A
TRINITY_DN22263_c0_g2_i14	FH18_ORYSJ	-10.23	1.54	5.3E-23	2.5E-20	#N/A	#N/A	#N/A	#N/A	#N/A	#N/A	#N/A	#N/A
TRINITY_DN21065_c1_g5_i5	BIP3_MAIZE	-10.22	1.53	1.9E-04	8.1E-03	#N/A	#N/A	#N/A	#N/A	#N/A	#N/A	#N/A	#N/A
TRINITY_DN19942_c4_g2_i1	M2K2_ARATH	-9.99	1.31	2.7E-15	6.7E-13	#N/A	#N/A	#N/A	#N/A	#N/A	#N/A	#N/A	#N/A
TRINITY_DN16238_c1_g1_i10	GSTFB_ARATH	-9.96	1.27	1.2E-15	3.1E-13	#N/A	#N/A	#N/A	#N/A	#N/A	#N/A	#N/A	#N/A
TRINITY_DN21089_c0_g6_i2	GSTU1_ORYSJ	-9.95	1.26	2.3E-04	9.3E-03	#N/A	#N/A	#N/A	#N/A	#N/A	#N/A	#N/A	#N/A
TRINITY_DN18883_c0_g1_i4	ADF2_ORYSJ	-9.94	1.25	1.5E-14	3.5E-12	#N/A	#N/A	#N/A	#N/A	#N/A	#N/A	#N/A	#N/A
TRINITY_DN19333_c0_g1_i2	RS18_ARATH	-9.82	1.14	1.0E-17	3.1E-15	#N/A	#N/A	#N/A	#N/A	#N/A	#N/A	#N/A	#N/A
TRINITY_DN22289_c0_g1_i9	CDKG2_ORYSJ	-9.54	0.87	1.1E-17	3.5E-15	#N/A	#N/A	#N/A	#N/A	#N/A	#N/A	#N/A	#N/A
TRINITY_DN19574_c0_g2_i8	SBT37_ARATH	-9.49	0.82	4.9E-14	1.1E-11	#N/A	#N/A	#N/A	#N/A	#N/A	#N/A	#N/A	#N/A
TRINITY_DN17692_c2_g1_i20	OSB1_ARATH	-9.47	0.80	2.7E-16	7.4E-14	#N/A	#N/A	#N/A	#N/A	#N/A	#N/A	#N/A	#N/A
TRINITY_DN18565_c0_g1_i7	CKX4_ORYSJ	-9.37	0.70	6.2E-12	1.1E-09	#N/A	#N/A	#N/A	#N/A	#N/A	#N/A	#N/A	#N/A
TRINITY_DN19324_c1_g1_i4	SBT25_ARATH	-9.27	0.60	1.2E-05	7.4E-04	#N/A	#N/A	#N/A	#N/A	#N/A	#N/A	#N/A	#N/A
TRINITY_DN18483_c1_g1_i6	KASC1_HORVU	-9.26	0.60	1.1E-09	1.4E-07	#N/A	#N/A	#N/A	#N/A	#N/A	#N/A	#N/A	#N/A
TRINITY_DN20688_c1_g1_i16	AMSH3_ARATH	-9.25	0.60	1.7E-03	4.7E-02	#N/A	#N/A	#N/A	#N/A	#N/A	#N/A	#N/A	#N/A
TRINITY_DN22128_c0_g1_i31	MSH5_ORYSJ	-9.17	0.52	1.8E-03	4.8E-02	#N/A	#N/A	#N/A	#N/A	#N/A	#N/A	#N/A	#N/A
TRINITY_DN18806_c1_g1_i4	FDH1_ORYSJ	-9.02	0.37	2.0E-12	3.7E-10	#N/A	#N/A	#N/A	#N/A	#N/A	#N/A	#N/A	#N/A
TRINITY_DN20559_c0_g1_i13	RL4A_ARATH	-9.01	0.36	4.5E-12	7.8E-10	#N/A	#N/A	#N/A	#N/A	#N/A	#N/A	#N/A	#N/A
TRINITY_DN17394_c0_g2_i4	4CL2_ORYSJ	-8.94	0.30	1.8E-09	2.2E-07	#N/A	#N/A	#N/A	#N/A	#N/A	#N/A	#N/A	#N/A
TRINITY_DN21067_c0_g2_i14	MCM31_MAIZE	-8.94	0.30	6.2E-12	1.0E-09	#N/A	#N/A	#N/A	#N/A	#N/A	#N/A	#N/A	#N/A
TRINITY_DN13445_c0_g1_i1	POLX_TOBAC	-8.93	0.28	7.5E-04	2.5E-02	#N/A	#N/A	#N/A	#N/A	#N/A	#N/A	#N/A	#N/A
TRINITY_DN22006_c2_g1_i6	SALR_PAPBR	-8.92	0.28	4.4E-08	4.5E-06	#N/A	#N/A	#N/A	#N/A	#N/A	#N/A	#N/A	#N/A
TRINITY_DN22149_c0_g1_i8	PGMC1_MAIZE	-8.90	0.26	9.8E-14	2.0E-11	#N/A	#N/A	#N/A	#N/A	#N/A	#N/A	#N/A	#N/A
TRINITY_DN21670_c1_g1_i1	RNHX1_ARATH	-8.89	0.24	2.0E-07	1.8E-05	#N/A	#N/A	#N/A	#N/A	#N/A	#N/A	#N/A	#N/A
TRINITY_DN21067_c0_g2_i10	MCM31_MAIZE	-8.87	0.23	3.9E-13	7.6E-11	#N/A	#N/A	#N/A	#N/A	#N/A	#N/A	#N/A	#N/A
TRINITY_DN17649_c0_g1_i1	AP1S1_ARATH	-8.82	0.19	1.4E-11	2.3E-09	#N/A	#N/A	#N/A	#N/A	#N/A	#N/A	#N/A	#N/A
TRINITY_DN22059_c0_g2_i6	PPP7L_ARATH	-8.81	0.16	4.7E-08	4.7E-06	#N/A	#N/A	#N/A	#N/A	#N/A	#N/A	#N/A	#N/A
TRINITY_DN14735_c0_g6_i8	U84A1_ARATH	-8.77	0.13	8.2E-07	6.4E-05	#N/A	#N/A	#N/A	#N/A	#N/A	#N/A	#N/A	#N/A
TRINITY_DN22355_c2_g3_i4	EGY1_ORYSJ	-8.74	0.11	5.0E-10	6.6E-08	#N/A	#N/A	#N/A	#N/A	#N/A	#N/A	#N/A	#N/A
TRINITY_DN20236_c0_g2_i1	ADT_ORYSJ	-8.74	0.10	1.3E-11	2.2E-09	#N/A	#N/A	#N/A	#N/A	#N/A	#N/A	#N/A	#N/A
TRINITY_DN17522_c0_g1_i16	PPK15_SCHPO	-8.71	0.09	1.7E-10	2.4E-08	#N/A	#N/A	#N/A	#N/A	#N/A	#N/A	#N/A	#N/A
TRINITY_DN15488_c0_g1_i7	STR4A_ARATH	-8.69	0.06	2.7E-04	1.1E-02	#N/A	#N/A	#N/A	#N/A	#N/A	#N/A	#N/A	#N/A
TRINITY_DN16878_c1_g1_i12	SYT5_ARATH	-8.68	0.06	1.2E-08	1.3E-06	#N/A	#N/A	#N/A	#N/A	#N/A	#N/A	#N/A	#N/A
TRINITY_DN18213_c0_g1_i16	RQL3_ARATH	-8.65	0.03	2.1E-06	1.5E-04	#N/A	#N/A	#N/A	#N/A	#N/A	#N/A	#N/A	#N/A
TRINITY_DN16858_c0_g1_i5	PP127_ARATH	-8.63	0.01	2.6E-11	4.1E-09	#N/A	#N/A	#N/A	#N/A	#N/A	#N/A	#N/A	#N/A
TRINITY_DN21479_c0_g4_i5	ZDHC2_ARATH	-8.59	-0.03	7.4E-10	9.6E-08	#N/A	#N/A	#N/A	#N/A	#N/A	#N/A	#N/A	#N/A
TRINITY_DN17465_c1_g2_i2	OTU_ARATH	-8.54	-0.09	2.3E-04	9.5E-03	#N/A	#N/A	#N/A	#N/A	#N/A	#N/A	#N/A	#N/A
TRINITY_DN20328_c0_g1_i3	HYES_PIG	-8.53	-0.09	9.4E-11	1.4E-08	#N/A	#N/A	#N/A	#N/A	#N/A	#N/A	#N/A	#N/A
TRINITY_DN21180_c1_g1_i7	UNE12_ARATH	-8.39	-0.21	3.7E-09	4.4E-07	#N/A	#N/A	#N/A	#N/A	#N/A	#N/A	#N/A	#N/A
TRINITY_DN21525_c0_g2_i1	CPL3_ARATH	-8.35	-0.26	5.6E-07	4.5E-05	#N/A	#N/A	#N/A	#N/A	#N/A	#N/A	#N/A	#N/A
TRINITY_DN19942_c4_g1_i3	PLT6_ARATH	-8.35	-0.25	2.9E-07	2.5E-05	#N/A	#N/A	#N/A	#N/A	#N/A	#N/A	#N/A	#N/A
TRINITY_DN22513_c1_g2_i8	WRKY2_ARATH	-8.34	-0.26	7.4E-08	7.2E-06	#N/A	#N/A	#N/A	#N/A	#N/A	#N/A	#N/A	#N/A
TRINITY_DN16869_c1_g3_i16	ACR4_ARATH	-8.32	-0.27	9.9E-06	6.0E-04	#N/A	#N/A	#N/A	#N/A	#N/A	#N/A	#N/A	#N/A
TRINITY_DN20589_c1_g2_i21	GLTP2_ARATH	-8.26	1.42	5.3E-04	1.9E-02	#N/A	#N/A	#N/A	#N/A	#N/A	#N/A	#N/A	#N/A
TRINITY_DN19414_c0_g1_i8	ARFY_ORYSJ	-8.26	-0.33	4.0E-05	2.1E-03	#N/A	#N/A	#N/A	#N/A	#N/A	#N/A	#N/A	#N/A
TRINITY_DN21067_c0_g2_i17	MCM31_MAIZE	-8.25	-0.34	1.5E-06	1.1E-04	#N/A	#N/A	#N/A	#N/A	#N/A	#N/A	#N/A	#N/A
TRINITY_DN22396_c1_g3_i2	SALT_ORYSJ	-8.14	3.45	4.2E-06	2.7E-04	#N/A	#N/A	#N/A	#N/A	#N/A	#N/A	#N/A	#N/A
TRINITY_DN15238_c0_g1_i4	PGTB2_HUMAN	-8.12	-0.46	2.0E-07	1.8E-05	#N/A	#N/A	#N/A	#N/A	#N/A	#N/A	#N/A	#N/A
TRINITY_DN19045_c0_g4_i2	AB19B_ARATH	-8.12	-0.47	2.2E-08	2.3E-06	#N/A	#N/A	#N/A	#N/A	#N/A	#N/A	#N/A	#N/A
TRINITY_DN20804_c0_g1_i10	EGY1_ORYSJ	-8.08	-0.50	3.0E-07	2.6E-05	#N/A	#N/A	#N/A	#N/A	#N/A	#N/A	#N/A	#N/A
TRINITY_DN15713_c1_g1_i8	AAPC_CENCI	-8.06	-0.52	1.2E-07	1.2E-05	#N/A	#N/A	#N/A	#N/A	#N/A	#N/A	#N/A	#N/A
TRINITY_DN20040_c0_g1_i11	RS242_ARATH	-8.03	-0.53	3.9E-04	1.5E-02	#N/A	#N/A	#N/A	#N/A	#N/A	#N/A	#N/A	#N/A
TRINITY_DN22483_c1_g1_i5	EX84B_ARATH	-8.03	-0.54	3.9E-07	3.2E-05	#N/A	#N/A	#N/A	#N/A	#N/A	#N/A	#N/A	#N/A
TRINITY_DN20286_c0_g1_i16	CNR13_MAIZE	-8.02	-0.56	5.7E-08	5.6E-06	#N/A	#N/A	#N/A	#N/A	#N/A	#N/A	#N/A	#N/A
TRINITY_DN17014_c1_g4_i1	ACA10_ARATH	-8.00	-0.57	1.2E-07	1.2E-05	#N/A	#N/A	#N/A	#N/A	#N/A	#N/A	#N/A	#N/A
TRINITY_DN15307_c2_g1_i9	SSL2_ARATH	-7.92	-0.63	2.9E-05	1.6E-03	#N/A	#N/A	#N/A	#N/A	#N/A	#N/A	#N/A	#N/A

DET	Subject id	c9TR vs c7NT				c7TR vs c7NT				c7TR vs c9TR			
		Log_2	Log_2CPM	PValue	19FDR	Log_2	Log_2CPM	PValue	19FDR	Log_2	Log_2CPM	PValue	19FDR
TRINITY_DN21437_c0_g3_i3	STT3B_ORYSJ	-7.91	-0.64	5.4E-04	1.9E-02	#N/A	#N/A	#N/A	#N/A	#N/A	#N/A	#N/A	#N/A
TRINITY_DN14893_c2_g3_i1	SERK4_ARATH	-7.88	-0.68	5.8E-07	4.7E-05	#N/A	#N/A	#N/A	#N/A	#N/A	#N/A	#N/A	#N/A
TRINITY_DN18372_c0_g1_i15	SPIN1_ORYSJ	-7.71	-0.84	2.1E-05	1.2E-03	#N/A	#N/A	#N/A	#N/A	#N/A	#N/A	#N/A	#N/A
TRINITY_DN17649_c0_g1_i4	AP1S1_ARATH	-7.57	-0.94	3.0E-04	1.2E-02	#N/A	#N/A	#N/A	#N/A	#N/A	#N/A	#N/A	#N/A
TRINITY_DN9211_c0_g1_i1	CLH1_ARATH	-7.37	3.35	8.8E-05	4.2E-03	#N/A	#N/A	#N/A	#N/A	#N/A	#N/A	#N/A	#N/A
TRINITY_DN21067_c0_g2_i13	MCM31_MAIZE	-7.23	0.36	5.8E-11	8.8E-09	#N/A	#N/A	#N/A	#N/A	#N/A	#N/A	#N/A	#N/A
TRINITY_DN18561_c0_g1_i9	ABIL5_ORYSJ	-6.74	1.17	4.4E-10	5.9E-08	#N/A	#N/A	#N/A	#N/A	#N/A	#N/A	#N/A	#N/A
TRINITY_DN16092_c1_g7_i2	RF2A_ORYSJ	-6.16	-0.59	6.5E-07	5.1E-05	#N/A	#N/A	#N/A	#N/A	#N/A	#N/A	#N/A	#N/A
TRINITY_DN20040_c0_g1_i9	RS242_ARATH	-6.07	-0.66	3.6E-06	2.4E-04	#N/A	#N/A	#N/A	#N/A	#N/A	#N/A	#N/A	#N/A
TRINITY_DN16955_c0_g2_i7	SPAST_XENLA	-6.04	2.40	1.2E-03	3.6E-02	#N/A	#N/A	#N/A	#N/A	#N/A	#N/A	#N/A	#N/A
TRINITY_DN22387_c1_g2_i8	LGUL_ORYSJ	-5.99	-0.03	6.6E-07	5.2E-05	#N/A	#N/A	#N/A	#N/A	#N/A	#N/A	#N/A	#N/A
TRINITY_DN16790_c0_g1_i5	MA658_ARATH	-5.94	1.90	2.5E-04	1.0E-02	#N/A	#N/A	#N/A	#N/A	#N/A	#N/A	#N/A	#N/A
TRINITY_DN22198_c1_g4_i1	DDPS3_ARATH	-5.92	3.62	9.1E-04	2.9E-02	#N/A	#N/A	#N/A	#N/A	#N/A	#N/A	#N/A	#N/A
TRINITY_DN15384_c0_g1_i14	MP704_ORYSJ	-5.91	2.19	7.6E-04	2.5E-02	#N/A	#N/A	#N/A	#N/A	#N/A	#N/A	#N/A	#N/A
TRINITY_DN21067_c0_g2_i15	MCM31_MAIZE	-5.81	-0.24	3.5E-08	3.6E-06	#N/A	#N/A	#N/A	#N/A	#N/A	#N/A	#N/A	#N/A
TRINITY_DN16828_c0_g2_i2	TKPR1_ARATH	-5.54	-0.03	3.7E-07	3.1E-05	#N/A	#N/A	#N/A	#N/A	#N/A	#N/A	#N/A	#N/A
TRINITY_DN16346_c2_g3_i3	GLGB3_ARATH	-5.44	-0.52	5.2E-06	3.3E-04	#N/A	#N/A	#N/A	#N/A	#N/A	#N/A	#N/A	#N/A
TRINITY_DN22258_c0_g1_i14	BBP_NEUCR	-5.42	-0.05	2.7E-04	1.1E-02	#N/A	#N/A	#N/A	#N/A	#N/A	#N/A	#N/A	#N/A
TRINITY_DN18112_c1_g1_i2	NUD18_ARATH	-5.33	0.21	3.2E-04	1.2E-02	#N/A	#N/A	#N/A	#N/A	#N/A	#N/A	#N/A	#N/A
TRINITY_DN19649_c1_g2_i11	Y2446_ARATH	-5.31	0.47	6.3E-05	3.1E-03	#N/A	#N/A	#N/A	#N/A	#N/A	#N/A	#N/A	#N/A
TRINITY_DN21702_c0_g1_i4	TBL3_RAT	-5.16	-0.39	4.2E-04	1.5E-02	#N/A	#N/A	#N/A	#N/A	#N/A	#N/A	#N/A	#N/A
TRINITY_DN18339_c0_g1_i15	LOFG2_ARATH	-5.14	-0.09	3.2E-04	1.2E-02	#N/A	#N/A	#N/A	#N/A	#N/A	#N/A	#N/A	#N/A
TRINITY_DN17254_c0_g1_i9	NICA_ARATH	-5.03	0.93	3.8E-07	3.2E-05	#N/A	#N/A	#N/A	#N/A	#N/A	#N/A	#N/A	#N/A
TRINITY_DN22103_c0_g4_i1	CSLF3_ORYSJ	-4.94	3.56	9.9E-04	3.1E-02	#N/A	#N/A	#N/A	#N/A	#N/A	#N/A	#N/A	#N/A
TRINITY_DN20051_c0_g1_i5	MDHM_EUCGU	-4.83	2.98	7.8E-04	2.5E-02	#N/A	#N/A	#N/A	#N/A	#N/A	#N/A	#N/A	#N/A
TRINITY_DN18339_c0_g1_i21	LOFG2_ARATH	-4.69	-0.37	1.6E-04	6.8E-03	#N/A	#N/A	#N/A	#N/A	#N/A	#N/A	#N/A	#N/A
TRINITY_DN19733_c0_g3_i6	BAM1_ARATH	-4.21	1.33	1.5E-05	8.9E-04	#N/A	#N/A	#N/A	#N/A	#N/A	#N/A	#N/A	#N/A
TRINITY_DN19241_c1_g1_i8	DXS1_ORYSJ	-4.04	1.85	3.7E-04	1.4E-02	#N/A	#N/A	#N/A	#N/A	#N/A	#N/A	#N/A	#N/A
TRINITY_DN16545_c0_g2_i3	PARN_ARATH	-4.01	-0.15	2.1E-03	5.4E-02	#N/A	#N/A	#N/A	#N/A	#N/A	#N/A	#N/A	#N/A
TRINITY_DN17679_c1_g4_i2	FBK28_ARATH	-4.01	0.83	6.5E-10	8.4E-08	#N/A	#N/A	#N/A	#N/A	#N/A	#N/A	#N/A	#N/A
TRINITY_DN16452_c0_g1_i8	H1_MAIZE	-3.97	0.76	6.1E-07	4.8E-05	#N/A	#N/A	#N/A	#N/A	#N/A	#N/A	#N/A	#N/A
TRINITY_DN20820_c1_g1_i14	PIN3A_ORYSJ	-3.87	-0.63	5.4E-04	1.9E-02	#N/A	#N/A	#N/A	#N/A	#N/A	#N/A	#N/A	#N/A
TRINITY_DN20777_c0_g2_i15	YBEY_SYNPX	-3.69	1.11	7.1E-04	2.4E-02	#N/A	#N/A	#N/A	#N/A	#N/A	#N/A	#N/A	#N/A
TRINITY_DN12770_c0_g1_i1	CSE_ARATH	-3.69	-0.46	1.2E-03	3.7E-02	#N/A	#N/A	#N/A	#N/A	#N/A	#N/A	#N/A	#N/A
TRINITY_DN21439_c1_g1_i19	SR34_ARATH	-3.56	2.82	7.3E-04	2.4E-02	#N/A	#N/A	#N/A	#N/A	#N/A	#N/A	#N/A	#N/A
TRINITY_DN17050_c0_g2_i5	RK17_TOBAC	-3.56	5.02	1.0E-11	1.7E-09	#N/A	#N/A	#N/A	#N/A	#N/A	#N/A	#N/A	#N/A
TRINITY_DN17790_c0_g2_i10	BX9_MAIZE	-3.51	1.63	1.8E-06	1.3E-04	#N/A	#N/A	#N/A	#N/A	#N/A	#N/A	#N/A	#N/A
TRINITY_DN20591_c0_g1_i6	LOR6_ARATH	-3.50	-0.21	1.4E-03	4.0E-02	#N/A	#N/A	#N/A	#N/A	#N/A	#N/A	#N/A	#N/A
TRINITY_DN14753_c1_g1_i1	PTR2_ARATH	-3.46	1.25	1.7E-05	9.8E-04	#N/A	#N/A	#N/A	#N/A	#N/A	#N/A	#N/A	#N/A
TRINITY_DN12386_c0_g1_i1	TYDC4_PETCR	-3.22	2.80	3.1E-04	1.2E-02	#N/A	#N/A	#N/A	#N/A	#N/A	#N/A	#N/A	#N/A
TRINITY_DN3735_c0_g1_i1	APY3_ORYSJ	-3.18	-0.53	8.8E-04	2.8E-02	#N/A	#N/A	#N/A	#N/A	#N/A	#N/A	#N/A	#N/A
TRINITY_DN21735_c0_g1_i1	GSTU1_ORYSJ	-3.16	2.71	1.9E-09	2.3E-07	#N/A	#N/A	#N/A	#N/A	#N/A	#N/A	#N/A	#N/A
TRINITY_DN16731_c0_g1_i7	ARF1_CHLRE	-3.16	4.14	7.5E-15	1.8E-12	#N/A	#N/A	#N/A	#N/A	#N/A	#N/A	#N/A	#N/A
TRINITY_DN20283_c0_g7_i2	BH049_ARATH	-3.12	2.84	4.6E-04	1.7E-02	#N/A	#N/A	#N/A	#N/A	#N/A	#N/A	#N/A	#N/A
TRINITY_DN22275_c0_g1_i4	LOX4_ORYSJ	-3.12	2.17	1.3E-03	3.8E-02	#N/A	#N/A	#N/A	#N/A	#N/A	#N/A	#N/A	#N/A
TRINITY_DN21067_c0_g2_i16	MCM33_MAIZE	-2.94	0.14	1.7E-06	1.2E-04	#N/A	#N/A	#N/A	#N/A	#N/A	#N/A	#N/A	#N/A
TRINITY_DN17868_c3_g1_i4	BX9_MAIZE	-2.91	1.81	1.1E-03	3.4E-02	#N/A	#N/A	#N/A	#N/A	#N/A	#N/A	#N/A	#N/A
TRINITY_DN20694_c0_g4_i2	CESA6_ORYSJ	-2.83	1.17	5.8E-04	2.0E-02	#N/A	#N/A	#N/A	#N/A	#N/A	#N/A	#N/A	#N/A
TRINITY_DN21331_c0_g1_i10	AB12A_ARATH	-2.82	3.77	5.2E-11	7.9E-09	#N/A	#N/A	#N/A	#N/A	#N/A	#N/A	#N/A	#N/A
TRINITY_DN22256_c0_g1_i11	OMT2_SORBI	-2.77	1.19	1.0E-04	4.8E-03	#N/A	#N/A	#N/A	#N/A	#N/A	#N/A	#N/A	#N/A
TRINITY_DN15600_c1_g5_i1	ZDHC8_ARATH	-2.76	0.30	4.4E-05	2.3E-03	#N/A	#N/A	#N/A	#N/A	#N/A	#N/A	#N/A	#N/A
TRINITY_DN15553_c0_g4_i3	DPNP_ORYSJ	-2.76	1.83	1.7E-03	4.6E-02	#N/A	#N/A	#N/A	#N/A	#N/A	#N/A	#N/A	#N/A
TRINITY_DN19574_c0_g2_i12	SBT37_ARATH	-2.75	3.36	1.1E-05	6.5E-04	#N/A	#N/A	#N/A	#N/A	#N/A	#N/A	#N/A	#N/A
TRINITY_DN22275_c0_g1_i12	LOX4_ORYSJ	-2.74	2.06	7.1E-04	2.4E-02	#N/A	#N/A	#N/A	#N/A	#N/A	#N/A	#N/A	#N/A
TRINITY_DN17301_c0_g1_i2	LHCA3_ARATH	-2.70	6.11	1.0E-03	3.1E-02	#N/A	#N/A	#N/A	#N/A	#N/A	#N/A	#N/A	#N/A
TRINITY_DN17187_c0_g3_i7	ACR6_ARATH	-2.69	-0.46	8.2E-04	2.7E-02	#N/A	#N/A	#N/A	#N/A	#N/A	#N/A	#N/A	#N/A
TRINITY_DN18748_c0_g4_i6	RH27_ORYSJ	-2.65	2.30	5.1E-06	3.3E-04	#N/A	#N/A	#N/A	#N/A	#N/A	#N/A	#N/A	#N/A
TRINITY_DN15135_c1_g2_i1	HOX8_ORYSJ	-2.62	0.01	2.1E-03	5.4E-02	#N/A	#N/A	#N/A	#N/A	#N/A	#N/A	#N/A	#N/A
TRINITY_DN18434_c2_g1_i1	RADL6_ARATH	-2.58	7.41	1.4E-22	6.4E-20	#N/A	#N/A	#N/A	#N/A	#N/A	#N/A	#N/A	#N/A
TRINITY_DN18434_c2_g1_i2	RADL6_ARATH	-2.53	2.40	1.8E-04	7.5E-03	#N/A	#N/A	#N/A	#N/A	#N/A	#N/A	#N/A	#N/A
TRINITY_DN17196_c0_g5_i3	MAOC_ORYSJ	-2.51	4.08	2.6E-05	1.4E-03	#N/A	#N/A	#N/A	#N/A	#N/A	#N/A	#N/A	#N/A

DET	Subject id	c9TR vs c7NT				c7TR vs c7NT				c7TR vs c9TR			
		Log_2	Log_2CPM	PValue	19FDR	Log_2	Log_2CPM	PValue	19FDR	Log_2	Log_2CPM	PValue	19FDR
TRINITY_DN21510_c2_g2_i4	APR1_ORYSJ	-2.51	3.58	7.1E-07	5.5E-05	#N/A	#N/A	#N/A	#N/A	#N/A	#N/A	#N/A	#N/A
TRINITY_DN20254_c0_g1_i4	ACCR3_ARATH	-2.48	0.92	8.4E-05	4.0E-03	#N/A	#N/A	#N/A	#N/A	#N/A	#N/A	#N/A	#N/A
TRINITY_DN21372_c0_g2_i17	EBF1_ARATH	-2.47	0.46	2.1E-04	8.7E-03	#N/A	#N/A	#N/A	#N/A	#N/A	#N/A	#N/A	#N/A
TRINITY_DN15519_c2_g6_i10	GDL83_ARATH	-2.46	3.69	2.0E-03	5.2E-02	#N/A	#N/A	#N/A	#N/A	#N/A	#N/A	#N/A	#N/A
TRINITY_DN16753_c0_g5_i4	HOL3_ARATH	-2.43	0.82	6.0E-04	2.1E-02	#N/A	#N/A	#N/A	#N/A	#N/A	#N/A	#N/A	#N/A
TRINITY_DN16430_c0_g1_i7	RTNLH_ARATH	-2.37	0.85	1.6E-03	4.5E-02	#N/A	#N/A	#N/A	#N/A	#N/A	#N/A	#N/A	#N/A
TRINITY_DN22778_c4_g1_i5	POLX_TOBAC	-2.37	2.67	5.7E-04	2.0E-02	#N/A	#N/A	#N/A	#N/A	#N/A	#N/A	#N/A	#N/A
TRINITY_DN16113_c0_g2_i6	NPY1_ARATH	-2.35	1.36	1.1E-05	6.8E-04	#N/A	#N/A	#N/A	#N/A	#N/A	#N/A	#N/A	#N/A
TRINITY_DN18172_c0_g1_i20	GUAD_BACSU	-2.34	0.30	6.6E-04	2.2E-02	#N/A	#N/A	#N/A	#N/A	#N/A	#N/A	#N/A	#N/A
TRINITY_DN17394_c0_g3_i5	WTR29_ARATH	-2.33	2.91	3.6E-06	2.4E-04	#N/A	#N/A	#N/A	#N/A	#N/A	#N/A	#N/A	#N/A
TRINITY_DN20440_c0_g2_i8	Y5977_ARATH	-2.28	1.48	6.0E-04	2.1E-02	#N/A	#N/A	#N/A	#N/A	#N/A	#N/A	#N/A	#N/A
TRINITY_DN11206_c0_g2_i1	GL814_ORYSJ	-2.27	7.62	2.6E-05	1.4E-03	#N/A	#N/A	#N/A	#N/A	#N/A	#N/A	#N/A	#N/A
TRINITY_DN16452_c0_g1_i11	H1_MAIZE	-2.23	1.05	2.0E-05	1.1E-03	#N/A	#N/A	#N/A	#N/A	#N/A	#N/A	#N/A	#N/A
TRINITY_DN17212_c0_g3_i11	DHBK_SOLLC	-2.23	3.64	1.7E-03	4.5E-02	#N/A	#N/A	#N/A	#N/A	#N/A	#N/A	#N/A	#N/A
TRINITY_DN18434_c2_g1_i4	RADL6_ARATH	-2.22	2.41	5.9E-05	2.9E-03	#N/A	#N/A	#N/A	#N/A	#N/A	#N/A	#N/A	#N/A
TRINITY_DN9072_c0_g1_i1	URT1_FRAAN	-2.21	3.82	2.7E-04	1.1E-02	#N/A	#N/A	#N/A	#N/A	#N/A	#N/A	#N/A	#N/A
TRINITY_DN22609_c1_g1_i16	SERK2_ARATH	-2.20	1.23	1.4E-04	6.3E-03	#N/A	#N/A	#N/A	#N/A	#N/A	#N/A	#N/A	#N/A
TRINITY_DN20463_c0_g1_i7	Y2168_ARATH	-2.20	3.74	7.0E-07	5.5E-05	#N/A	#N/A	#N/A	#N/A	#N/A	#N/A	#N/A	#N/A
TRINITY_DN21308_c0_g5_i6	PP451_ARATH	-2.18	2.54	1.2E-03	3.5E-02	#N/A	#N/A	#N/A	#N/A	#N/A	#N/A	#N/A	#N/A
TRINITY_DN16003_c3_g3_i1	WTR45_ARATH	-2.17	1.21	5.5E-05	2.8E-03	#N/A	#N/A	#N/A	#N/A	#N/A	#N/A	#N/A	#N/A
TRINITY_DN17304_c0_g1_i1	RADL1_ARATH	-2.17	1.24	4.8E-04	1.7E-02	#N/A	#N/A	#N/A	#N/A	#N/A	#N/A	#N/A	#N/A
TRINITY_DN22314_c1_g1_i9	PAPS1_ARATH	-2.17	0.58	6.3E-04	2.1E-02	#N/A	#N/A	#N/A	#N/A	#N/A	#N/A	#N/A	#N/A
TRINITY_DN16019_c1_g1_i5	CESA5_ARATH	-2.16	0.27	6.4E-04	2.2E-02	#N/A	#N/A	#N/A	#N/A	#N/A	#N/A	#N/A	#N/A
TRINITY_DN15446_c0_g1_i3	CHI11_ORYSJ	-2.15	3.95	2.3E-04	9.4E-03	#N/A	#N/A	#N/A	#N/A	#N/A	#N/A	#N/A	#N/A
TRINITY_DN18924_c0_g1_i2	C86B1_ARATH	-2.14	3.80	2.0E-03	5.2E-02	#N/A	#N/A	#N/A	#N/A	#N/A	#N/A	#N/A	#N/A
TRINITY_DN21605_c0_g3_i8	AB11G_ARATH	-2.14	0.75	1.3E-03	3.8E-02	#N/A	#N/A	#N/A	#N/A	#N/A	#N/A	#N/A	#N/A
TRINITY_DN16853_c2_g1_i5	SALR_PAPBR	-2.11	4.61	9.5E-07	7.2E-05	#N/A	#N/A	#N/A	#N/A	#N/A	#N/A	#N/A	#N/A
TRINITY_DN20914_c1_g1_i8	PLT5_ARATH	-2.11	2.75	2.2E-08	2.3E-06	#N/A	#N/A	#N/A	#N/A	#N/A	#N/A	#N/A	#N/A
TRINITY_DN20894_c0_g4_i1	SBT17_ARATH	-2.09	1.22	8.2E-04	2.7E-02	#N/A	#N/A	#N/A	#N/A	#N/A	#N/A	#N/A	#N/A
TRINITY_DN20634_c0_g1_i2	PP312_ARATH	-2.07	3.42	2.7E-05	1.5E-03	#N/A	#N/A	#N/A	#N/A	#N/A	#N/A	#N/A	#N/A
TRINITY_DN15384_c0_g1_i1	RMT2_SCHPO	-2.07	0.12	5.5E-04	1.9E-02	#N/A	#N/A	#N/A	#N/A	#N/A	#N/A	#N/A	#N/A
TRINITY_DN13344_c0_g1_i4	PLCD2_ARATH	-2.06	2.94	1.6E-03	4.4E-02	#N/A	#N/A	#N/A	#N/A	#N/A	#N/A	#N/A	#N/A
TRINITY_DN19677_c0_g2_i11	MT127_ARATH	-2.06	0.56	2.5E-04	1.0E-02	#N/A	#N/A	#N/A	#N/A	#N/A	#N/A	#N/A	#N/A
TRINITY_DN19733_c0_g4_i6	AMYB_SOYBN	-2.05	2.70	2.8E-06	1.9E-04	#N/A	#N/A	#N/A	#N/A	#N/A	#N/A	#N/A	#N/A
TRINITY_DN20634_c0_g1_i13	PP312_ARATH	-2.04	0.74	9.3E-04	2.9E-02	#N/A	#N/A	#N/A	#N/A	#N/A	#N/A	#N/A	#N/A
TRINITY_DN16609_c0_g1_i4	YC20L_ARATH	-2.03	1.98	2.1E-05	1.2E-03	#N/A	#N/A	#N/A	#N/A	#N/A	#N/A	#N/A	#N/A
TRINITY_DN15176_c0_g3_i7	GLPT1_ARATH	-2.03	1.39	3.1E-04	1.2E-02	#N/A	#N/A	#N/A	#N/A	#N/A	#N/A	#N/A	#N/A
TRINITY_DN20179_c2_g4_i1	BEBT_CLABR	-2.03	4.13	2.5E-10	3.5E-08	#N/A	#N/A	#N/A	#N/A	#N/A	#N/A	#N/A	#N/A
TRINITY_DN17647_c0_g3_i2	YSL14_ORYSJ	-2.03	1.91	1.8E-04	7.6E-03	#N/A	#N/A	#N/A	#N/A	#N/A	#N/A	#N/A	#N/A
TRINITY_DN17025_c1_g1_i15	PAE3_ARATH	-1.97	1.37	1.7E-03	4.6E-02	#N/A	#N/A	#N/A	#N/A	#N/A	#N/A	#N/A	#N/A
TRINITY_DN15756_c0_g1_i9	GDL83_ARATH	-1.96	0.13	6.9E-04	2.3E-02	#N/A	#N/A	#N/A	#N/A	#N/A	#N/A	#N/A	#N/A
TRINITY_DN15615_c0_g1_i6	RS20_ORYSJ	-1.95	3.05	4.2E-04	1.6E-02	#N/A	#N/A	#N/A	#N/A	#N/A	#N/A	#N/A	#N/A
TRINITY_DN20538_c1_g5_i2	CSPLB_SORBI	-1.95	4.45	1.3E-03	3.7E-02	#N/A	#N/A	#N/A	#N/A	#N/A	#N/A	#N/A	#N/A
TRINITY_DN17707_c0_g1_i10	PPD2_ARATH	-1.95	1.47	1.5E-04	6.5E-03	#N/A	#N/A	#N/A	#N/A	#N/A	#N/A	#N/A	#N/A
TRINITY_DN22331_c0_g1_i18	RH40_ARATH	-1.95	1.62	1.2E-04	5.4E-03	#N/A	#N/A	#N/A	#N/A	#N/A	#N/A	#N/A	#N/A
TRINITY_DN14094_c0_g1_i4	CCR1_ARATH	-1.93	0.73	3.1E-04	1.2E-02	#N/A	#N/A	#N/A	#N/A	#N/A	#N/A	#N/A	#N/A
TRINITY_DN15226_c1_g3_i16	RISA_SCHPO	-1.91	1.04	1.1E-03	3.3E-02	#N/A	#N/A	#N/A	#N/A	#N/A	#N/A	#N/A	#N/A
TRINITY_DN15413_c0_g7_i3	OBGC1_ORYSJ	-1.90	4.04	2.1E-04	8.8E-03	#N/A	#N/A	#N/A	#N/A	#N/A	#N/A	#N/A	#N/A
TRINITY_DN22194_c1_g3_i15	CNGC2_ARATH	-1.90	0.90	6.8E-04	2.3E-02	#N/A	#N/A	#N/A	#N/A	#N/A	#N/A	#N/A	#N/A
TRINITY_DN17366_c0_g1_i2	MAD47_ORYSJ	-1.90	1.42	4.4E-04	1.6E-02	#N/A	#N/A	#N/A	#N/A	#N/A	#N/A	#N/A	#N/A
TRINITY_DN17199_c1_g2_i2	KASC2_ARATH	-1.90	2.14	5.9E-07	4.7E-05	#N/A	#N/A	#N/A	#N/A	#N/A	#N/A	#N/A	#N/A
TRINITY_DN18370_c1_g1_i10	PSAN_MAIZE	-1.89	2.28	1.5E-06	1.1E-04	#N/A	#N/A	#N/A	#N/A	#N/A	#N/A	#N/A	#N/A
TRINITY_DN17826_c0_g1_i9	APL_ARATH	-1.89	0.65	1.4E-03	4.0E-02	#N/A	#N/A	#N/A	#N/A	#N/A	#N/A	#N/A	#N/A
TRINITY_DN17652_c1_g1_i3	PLT1_ARATH	-1.88	2.88	1.1E-08	1.2E-06	#N/A	#N/A	#N/A	#N/A	#N/A	#N/A	#N/A	#N/A
TRINITY_DN16412_c0_g5_i4	CP20C_ARATH	-1.88	2.92	6.6E-07	5.2E-05	#N/A	#N/A	#N/A	#N/A	#N/A	#N/A	#N/A	#N/A
TRINITY_DN22114_c0_g1_i1	TRH22_ORYSJ	-1.88	4.29	2.0E-03	5.4E-02	#N/A	#N/A	#N/A	#N/A	#N/A	#N/A	#N/A	#N/A
TRINITY_DN14893_c2_g6_i3	SAU39_ORYSJ	-1.87	1.17	3.1E-05	1.7E-03	#N/A	#N/A	#N/A	#N/A	#N/A	#N/A	#N/A	#N/A
TRINITY_DN20495_c0_g1_i20	SCP17_ARATH	-1.87	2.24	4.7E-04	1.7E-02	#N/A	#N/A	#N/A	#N/A	#N/A	#N/A	#N/A	#N/A
TRINITY_DN16431_c0_g1_i39	PGP1B_ARATH	-1.87	3.46	1.3E-07	1.2E-05	#N/A	#N/A	#N/A	#N/A	#N/A	#N/A	#N/A	#N/A
TRINITY_DN15568_c0_g1_i9	DTD_DICDI	-1.85	0.93	2.5E-04	1.0E-02	#N/A	#N/A	#N/A	#N/A	#N/A	#N/A	#N/A	#N/A
TRINITY_DN19333_c0_g4_i1	APS2_ARATH	-1.84	6.72	5.4E-06	3.4E-04	#N/A	#N/A	#N/A	#N/A	#N/A	#N/A	#N/A	#N/A

DET	Subject id	c9TR vs c7NT				c7TR vs c7NT				c7TR vs c9TR			
		Log_2	Log_2CPM	PValue	19FDR	Log_2	Log_2CPM	PValue	19FDR	Log_2	Log_2CPM	PValue	19FDR
TRINITY_DN22346_c0_g1_i2	COB21_ORYSJ	-1.84	2.36	6.6E-05	3.2E-03	#N/A	#N/A	#N/A	#N/A	#N/A	#N/A	#N/A	#N/A
TRINITY_DN21521_c0_g2_i10	CERK1_ORYSJ	-1.83	0.44	6.2E-04	2.1E-02	#N/A	#N/A	#N/A	#N/A	#N/A	#N/A	#N/A	#N/A
TRINITY_DN15995_c0_g3_i2	LOX12_SOLTU	-1.83	3.88	1.6E-04	6.9E-03	#N/A	#N/A	#N/A	#N/A	#N/A	#N/A	#N/A	#N/A
TRINITY_DN18292_c2_g7_i1	FABI2_ORYSJ	-1.83	3.45	5.2E-06	3.4E-04	#N/A	#N/A	#N/A	#N/A	#N/A	#N/A	#N/A	#N/A
TRINITY_DN16626_c0_g3_i5	MAD50_ORYSJ	-1.83	1.90	1.0E-05	6.3E-04	#N/A	#N/A	#N/A	#N/A	#N/A	#N/A	#N/A	#N/A
TRINITY_DN22301_c2_g2_i17	APL_ARATH	-1.83	2.38	5.4E-04	1.9E-02	#N/A	#N/A	#N/A	#N/A	#N/A	#N/A	#N/A	#N/A
TRINITY_DN13999_c0_g1_i2	TRA1_MAIZE	-1.83	0.11	2.0E-03	5.3E-02	#N/A	#N/A	#N/A	#N/A	#N/A	#N/A	#N/A	#N/A
TRINITY_DN17781_c3_g1_i8	FMT_KOSOT	-1.82	3.23	1.8E-06	1.3E-04	#N/A	#N/A	#N/A	#N/A	#N/A	#N/A	#N/A	#N/A
TRINITY_DN18447_c0_g3_i1	RS27A_MAIZE	-1.81	1.45	1.1E-03	3.2E-02	#N/A	#N/A	#N/A	#N/A	#N/A	#N/A	#N/A	#N/A
TRINITY_DN19171_c0_g1_i5	BGAL9_ORYSJ	-1.81	4.91	1.4E-03	3.9E-02	#N/A	#N/A	#N/A	#N/A	#N/A	#N/A	#N/A	#N/A
TRINITY_DN15235_c0_g3_i2	BX6_MAIZE	-1.81	4.27	1.8E-03	4.8E-02	#N/A	#N/A	#N/A	#N/A	#N/A	#N/A	#N/A	#N/A
TRINITY_DN20927_c0_g1_i4	GT644_ARATH	-1.80	7.34	1.4E-13	2.8E-11	#N/A	#N/A	#N/A	#N/A	#N/A	#N/A	#N/A	#N/A
TRINITY_DN15503_c1_g5_i1	PLT5_ARATH	-1.79	1.18	8.9E-04	2.8E-02	#N/A	#N/A	#N/A	#N/A	#N/A	#N/A	#N/A	#N/A
TRINITY_DN21709_c0_g2_i4	ARID2_ARATH	-1.79	0.82	2.1E-03	5.4E-02	#N/A	#N/A	#N/A	#N/A	#N/A	#N/A	#N/A	#N/A
TRINITY_DN15711_c0_g2_i11	FER3_MAIZE	-1.78	4.85	8.9E-04	2.8E-02	#N/A	#N/A	#N/A	#N/A	#N/A	#N/A	#N/A	#N/A
TRINITY_DN15330_c0_g1_i1	R13A4_ARATH	-1.78	3.80	2.7E-09	3.2E-07	#N/A	#N/A	#N/A	#N/A	#N/A	#N/A	#N/A	#N/A
TRINITY_DN16112_c0_g1_i1	HMOX2_ORYSJ	-1.77	3.09	1.1E-06	8.6E-05	#N/A	#N/A	#N/A	#N/A	#N/A	#N/A	#N/A	#N/A
TRINITY_DN21994_c0_g1_i20	YCHF_BACSU	-1.77	4.56	2.0E-04	8.2E-03	#N/A	#N/A	#N/A	#N/A	#N/A	#N/A	#N/A	#N/A
TRINITY_DN20497_c1_g2_i12	RMR1_ORYSJ	-1.77	1.46	2.6E-04	1.0E-02	#N/A	#N/A	#N/A	#N/A	#N/A	#N/A	#N/A	#N/A
TRINITY_DN17991_c0_g2_i3	MORF2_ARATH	-1.76	2.10	1.2E-03	3.6E-02	#N/A	#N/A	#N/A	#N/A	#N/A	#N/A	#N/A	#N/A
TRINITY_DN14113_c0_g1_i5	S38A1_RAT	-1.76	4.19	4.4E-07	3.6E-05	#N/A	#N/A	#N/A	#N/A	#N/A	#N/A	#N/A	#N/A
TRINITY_DN19057_c0_g1_i6	CB121_HORVU	-1.76	5.89	1.0E-07	9.9E-06	#N/A	#N/A	#N/A	#N/A	#N/A	#N/A	#N/A	#N/A
TRINITY_DN21000_c0_g2_i14	PI5K2_ARATH	-1.75	1.94	5.4E-05	2.7E-03	#N/A	#N/A	#N/A	#N/A	#N/A	#N/A	#N/A	#N/A
TRINITY_DN14647_c0_g1_i1	PP186_ARATH	-1.75	3.54	1.8E-03	5.0E-02	#N/A	#N/A	#N/A	#N/A	#N/A	#N/A	#N/A	#N/A
TRINITY_DN18036_c0_g1_i11	Y202_CLOB8	-1.75	2.29	3.2E-05	1.7E-03	#N/A	#N/A	#N/A	#N/A	#N/A	#N/A	#N/A	#N/A
TRINITY_DN14698_c2_g4_i3	TRNHC_ARATH	-1.74	2.05	2.6E-04	1.0E-02	#N/A	#N/A	#N/A	#N/A	#N/A	#N/A	#N/A	#N/A
TRINITY_DN16002_c0_g3_i3	PP124_ARATH	-1.74	1.42	1.1E-03	3.2E-02	#N/A	#N/A	#N/A	#N/A	#N/A	#N/A	#N/A	#N/A
TRINITY_DN15106_c0_g2_i1	ACP1_HORVU	-1.73	2.47	4.2E-04	1.5E-02	#N/A	#N/A	#N/A	#N/A	#N/A	#N/A	#N/A	#N/A
TRINITY_DN17101_c1_g3_i5	RS141_MAIZE	-1.73	1.61	1.9E-04	7.9E-03	#N/A	#N/A	#N/A	#N/A	#N/A	#N/A	#N/A	#N/A
TRINITY_DN18467_c0_g2_i1	TL17_ARATH	-1.73	5.45	3.7E-12	6.4E-10	#N/A	#N/A	#N/A	#N/A	#N/A	#N/A	#N/A	#N/A
TRINITY_DN15357_c0_g1_i2	GDPD6_ARATH	-1.73	1.99	1.0E-03	3.1E-02	#N/A	#N/A	#N/A	#N/A	#N/A	#N/A	#N/A	#N/A
TRINITY_DN20766_c1_g1_i2	RSSA_VITVI	-1.72	2.41	1.1E-04	5.1E-03	#N/A	#N/A	#N/A	#N/A	#N/A	#N/A	#N/A	#N/A
TRINITY_DN17141_c0_g1_i7	C85A1_ORYSJ	-1.72	0.20	1.4E-03	4.0E-02	#N/A	#N/A	#N/A	#N/A	#N/A	#N/A	#N/A	#N/A
TRINITY_DN15503_c1_g5_i2	PLT5_ARATH	-1.71	3.38	2.4E-07	2.1E-05	#N/A	#N/A	#N/A	#N/A	#N/A	#N/A	#N/A	#N/A
TRINITY_DN19057_c0_g2_i1	CB121_HORVU	-1.70	7.56	3.2E-12	5.6E-10	#N/A	#N/A	#N/A	#N/A	#N/A	#N/A	#N/A	#N/A
TRINITY_DN20015_c0_g1_i22	CGEP_ORYSJ	-1.70	0.59	7.0E-04	2.3E-02	#N/A	#N/A	#N/A	#N/A	#N/A	#N/A	#N/A	#N/A
TRINITY_DN21281_c1_g2_i2	RL31_PERFR	-1.70	1.83	2.6E-05	1.4E-03	#N/A	#N/A	#N/A	#N/A	#N/A	#N/A	#N/A	#N/A
TRINITY_DN18169_c1_g1_i5	HEM3_ORYSJ	-1.70	6.74	3.2E-09	3.8E-07	#N/A	#N/A	#N/A	#N/A	#N/A	#N/A	#N/A	#N/A
TRINITY_DN19345_c0_g1_i14	MLOH1_ORYSJ	-1.69	3.90	2.1E-03	5.5E-02	#N/A	#N/A	#N/A	#N/A	#N/A	#N/A	#N/A	#N/A
TRINITY_DN17182_c0_g2_i4	HGGL2_MAIZE	-1.69	5.23	1.9E-03	5.0E-02	#N/A	#N/A	#N/A	#N/A	#N/A	#N/A	#N/A	#N/A
TRINITY_DN22006_c2_g1_i1	SALR_PAPBR	-1.68	3.22	1.1E-04	5.1E-03	#N/A	#N/A	#N/A	#N/A	#N/A	#N/A	#N/A	#N/A
TRINITY_DN20834_c0_g1_i4	RUBB_PEA	-1.68	6.18	6.9E-05	3.3E-03	#N/A	#N/A	#N/A	#N/A	#N/A	#N/A	#N/A	#N/A
TRINITY_DN20318_c0_g2_i3	CCB4_ARATH	-1.68	2.60	3.6E-06	2.4E-04	#N/A	#N/A	#N/A	#N/A	#N/A	#N/A	#N/A	#N/A
TRINITY_DN15553_c0_g3_i1	ROC4_NICSY	-1.67	3.69	1.8E-03	4.8E-02	#N/A	#N/A	#N/A	#N/A	#N/A	#N/A	#N/A	#N/A
TRINITY_DN21089_c0_g6_i3	GSTU1_ORYSJ	-1.65	3.73	6.8E-04	2.3E-02	#N/A	#N/A	#N/A	#N/A	#N/A	#N/A	#N/A	#N/A
TRINITY_DN19994_c1_g6_i4	CP33_ARATH	-1.65	5.43	7.8E-15	1.8E-12	#N/A	#N/A	#N/A	#N/A	#N/A	#N/A	#N/A	#N/A
TRINITY_DN20572_c3_g1_i3	BOLA4_ARATH	-1.65	4.39	3.2E-10	4.4E-08	#N/A	#N/A	#N/A	#N/A	#N/A	#N/A	#N/A	#N/A
TRINITY_DN20256_c1_g1_i9	RRFC_ORYSJ	-1.65	2.29	8.2E-04	2.7E-02	#N/A	#N/A	#N/A	#N/A	#N/A	#N/A	#N/A	#N/A
TRINITY_DN17454_c1_g1_i3	CPS3B_ARATH	-1.64	2.44	5.9E-04	2.0E-02	#N/A	#N/A	#N/A	#N/A	#N/A	#N/A	#N/A	#N/A
TRINITY_DN16868_c3_g1_i2	RK21_SPIOL	-1.64	3.65	1.8E-08	1.9E-06	#N/A	#N/A	#N/A	#N/A	#N/A	#N/A	#N/A	#N/A
TRINITY_DN21823_c1_g1_i15	ADCK1_CHICK	-1.64	3.22	1.1E-04	4.9E-03	#N/A	#N/A	#N/A	#N/A	#N/A	#N/A	#N/A	#N/A
TRINITY_DN19812_c1_g4_i1	CB48_MAIZE	-1.64	7.30	2.7E-06	1.9E-04	#N/A	#N/A	#N/A	#N/A	#N/A	#N/A	#N/A	#N/A
TRINITY_DN20142_c1_g4_i3	PR2E1_ORYSJ	-1.63	4.94	1.3E-06	9.9E-05	#N/A	#N/A	#N/A	#N/A	#N/A	#N/A	#N/A	#N/A
TRINITY_DN18432_c0_g1_i20	MCA1_ARATH	-1.62	1.01	7.0E-04	2.3E-02	#N/A	#N/A	#N/A	#N/A	#N/A	#N/A	#N/A	#N/A
TRINITY_DN21077_c1_g6_i1	CH10C_ARATH	-1.62	5.24	1.5E-10	2.1E-08	#N/A	#N/A	#N/A	#N/A	#N/A	#N/A	#N/A	#N/A
TRINITY_DN14663_c1_g1_i1	DCUP2_ORYSJ	-1.62	4.69	7.9E-05	3.8E-03	#N/A	#N/A	#N/A	#N/A	#N/A	#N/A	#N/A	#N/A
TRINITY_DN15535_c1_g6_i2	C74B2_ARATH	-1.60	3.91	1.3E-04	6.0E-03	#N/A	#N/A	#N/A	#N/A	#N/A	#N/A	#N/A	#N/A
TRINITY_DN15106_c0_g2_i4	ACP1_HORVU	-1.60	4.82	5.2E-10	6.8E-08	#N/A	#N/A	#N/A	#N/A	#N/A	#N/A	#N/A	#N/A
TRINITY_DN18418_c0_g1_i9	CHS2_MAIZE	-1.60	7.71	3.6E-04	1.3E-02	#N/A	#N/A	#N/A	#N/A	#N/A	#N/A	#N/A	#N/A
TRINITY_DN15250_c0_g2_i4	SFH9_ARATH	-1.59	1.26	3.9E-04	1.5E-02	#N/A	#N/A	#N/A	#N/A	#N/A	#N/A	#N/A	#N/A
TRINITY_DN22315_c0_g1_i4	HEM1_ORYSJ	-1.59	5.18	6.1E-06	3.8E-04	#N/A	#N/A	#N/A	#N/A	#N/A	#N/A	#N/A	#N/A

DET	Subject id	c9TR vs c7NT				c7TR vs c7NT				c7TR vs c9TR			
		Log_2	Log_2CPM	PValue	19FDR	Log_2	Log_2CPM	PValue	19FDR	Log_2	Log_2CPM	PValue	19FDR
TRINITY_DN19808_c1_g2_i6	GDL9_ARATH	-1.59	3.29	2.9E-07	2.5E-05	#N/A	#N/A	#N/A	#N/A	#N/A	#N/A	#N/A	#N/A
TRINITY_DN17182_c0_g3_i1	HGGL2_MAIZE	-1.58	6.19	3.6E-04	1.3E-02	#N/A	#N/A	#N/A	#N/A	#N/A	#N/A	#N/A	#N/A
TRINITY_DN21010_c0_g1_i8	NEN1_ARATH	-1.57	2.42	3.0E-04	1.2E-02	#N/A	#N/A	#N/A	#N/A	#N/A	#N/A	#N/A	#N/A
TRINITY_DN15984_c1_g3_i5	PHO13_ORYSJ	-1.57	4.06	4.5E-06	2.9E-04	#N/A	#N/A	#N/A	#N/A	#N/A	#N/A	#N/A	#N/A
TRINITY_DN15514_c2_g1_i5	RK11_ARATH	-1.57	6.55	1.8E-07	1.6E-05	#N/A	#N/A	#N/A	#N/A	#N/A	#N/A	#N/A	#N/A
TRINITY_DN20137_c1_g2_i7	LBD6_MAIZE	-1.56	1.08	1.4E-03	4.0E-02	#N/A	#N/A	#N/A	#N/A	#N/A	#N/A	#N/A	#N/A
TRINITY_DN20411_c0_g1_i6	PGKH_WHEAT	-1.56	8.07	3.0E-05	1.6E-03	#N/A	#N/A	#N/A	#N/A	#N/A	#N/A	#N/A	#N/A
TRINITY_DN20194_c0_g2_i1	TRPA1_ARATH	-1.55	4.32	1.9E-04	7.9E-03	#N/A	#N/A	#N/A	#N/A	#N/A	#N/A	#N/A	#N/A
TRINITY_DN17576_c0_g1_i5	CLPT1_ARATH	-1.55	3.79	7.6E-08	7.4E-06	#N/A	#N/A	#N/A	#N/A	#N/A	#N/A	#N/A	#N/A
TRINITY_DN15690_c1_g1_i4	RBS_MAIZE	-1.55	11.08	5.1E-09	5.9E-07	#N/A	#N/A	#N/A	#N/A	#N/A	#N/A	#N/A	#N/A
TRINITY_DN17946_c0_g2_i2	DHAS_PROMA	-1.55	0.83	1.4E-03	4.0E-02	#N/A	#N/A	#N/A	#N/A	#N/A	#N/A	#N/A	#N/A
TRINITY_DN16412_c0_g5_i5	CP20C_ARATH	-1.54	1.97	1.6E-03	4.5E-02	#N/A	#N/A	#N/A	#N/A	#N/A	#N/A	#N/A	#N/A
TRINITY_DN20781_c0_g2_i7	FK163_ARATH	-1.54	2.07	1.9E-03	5.1E-02	#N/A	#N/A	#N/A	#N/A	#N/A	#N/A	#N/A	#N/A
TRINITY_DN18791_c1_g1_i20	GPDA_CUPLA	-1.53	1.26	1.7E-03	4.7E-02	#N/A	#N/A	#N/A	#N/A	#N/A	#N/A	#N/A	#N/A
TRINITY_DN17366_c0_g1_i5	MAD47_ORYSJ	-1.53	4.76	1.1E-05	6.6E-04	#N/A	#N/A	#N/A	#N/A	#N/A	#N/A	#N/A	#N/A
TRINITY_DN18420_c0_g1_i2	3MG_ARATH	-1.53	2.28	8.2E-04	2.7E-02	#N/A	#N/A	#N/A	#N/A	#N/A	#N/A	#N/A	#N/A
TRINITY_DN18917_c0_g2_i10	CER3_ARATH	-1.53	3.26	2.1E-05	1.2E-03	#N/A	#N/A	#N/A	#N/A	#N/A	#N/A	#N/A	#N/A
TRINITY_DN17752_c0_g1_i3	LTD_ORYSJ	-1.52	3.96	1.3E-08	1.4E-06	#N/A	#N/A	#N/A	#N/A	#N/A	#N/A	#N/A	#N/A
TRINITY_DN15630_c0_g5_i1	ACBP_RICCO	-1.51	3.67	2.4E-04	9.7E-03	#N/A	#N/A	#N/A	#N/A	#N/A	#N/A	#N/A	#N/A
TRINITY_DN15176_c0_g3_i1	GLPT1_ARATH	-1.51	2.85	1.2E-06	8.9E-05	#N/A	#N/A	#N/A	#N/A	#N/A	#N/A	#N/A	#N/A
TRINITY_DN17092_c0_g2_i10	RECAC_ARATH	-1.50	4.23	3.2E-04	1.2E-02	#N/A	#N/A	#N/A	#N/A	#N/A	#N/A	#N/A	#N/A
TRINITY_DN22113_c1_g8_i1	P5CS_ORYSJ	-1.50	2.57	3.1E-05	1.7E-03	#N/A	#N/A	#N/A	#N/A	#N/A	#N/A	#N/A	#N/A
TRINITY_DN16136_c0_g3_i5	SODCP_ORYSJ	-1.49	7.47	4.1E-07	3.4E-05	#N/A	#N/A	#N/A	#N/A	#N/A	#N/A	#N/A	#N/A
TRINITY_DN14115_c0_g1_i6	TL29_SOLLC	-1.48	8.28	4.8E-07	3.9E-05	#N/A	#N/A	#N/A	#N/A	#N/A	#N/A	#N/A	#N/A
TRINITY_DN20475_c0_g2_i4	RRP3_HORVU	-1.47	4.04	2.2E-04	9.1E-03	#N/A	#N/A	#N/A	#N/A	#N/A	#N/A	#N/A	#N/A
TRINITY_DN13355_c0_g1_i1	ACP1_CASGL	-1.47	6.47	3.6E-09	4.3E-07	#N/A	#N/A	#N/A	#N/A	#N/A	#N/A	#N/A	#N/A
TRINITY_DN17826_c0_g1_i5	APL_ARATH	-1.45	2.51	1.8E-03	4.8E-02	#N/A	#N/A	#N/A	#N/A	#N/A	#N/A	#N/A	#N/A
TRINITY_DN15469_c2_g2_i1	PNSL2_ARATH	-1.45	2.55	1.0E-05	6.1E-04	#N/A	#N/A	#N/A	#N/A	#N/A	#N/A	#N/A	#N/A
TRINITY_DN19542_c0_g1_i14	YCF23_PYRYE	-1.45	7.12	5.9E-04	2.0E-02	#N/A	#N/A	#N/A	#N/A	#N/A	#N/A	#N/A	#N/A
TRINITY_DN17059_c0_g1_i10	F3PH_ARATH	-1.45	4.61	9.3E-05	4.3E-03	#N/A	#N/A	#N/A	#N/A	#N/A	#N/A	#N/A	#N/A
TRINITY_DN21391_c0_g1_i7	LHCA6_ARATH	-1.44	7.94	8.4E-06	5.2E-04	#N/A	#N/A	#N/A	#N/A	#N/A	#N/A	#N/A	#N/A
TRINITY_DN19529_c0_g2_i17	XB32_ORYSJ	-1.44	2.23	1.6E-03	4.5E-02	#N/A	#N/A	#N/A	#N/A	#N/A	#N/A	#N/A	#N/A
TRINITY_DN17868_c3_g1_i7	BX8_MAIZE	-1.44	3.19	2.4E-04	9.8E-03	#N/A	#N/A	#N/A	#N/A	#N/A	#N/A	#N/A	#N/A
TRINITY_DN14439_c0_g3_i2	SLD2_ARATH	-1.43	2.93	4.1E-06	2.7E-04	#N/A	#N/A	#N/A	#N/A	#N/A	#N/A	#N/A	#N/A
TRINITY_DN14758_c0_g2_i7	PNSL5_ARATH	-1.43	7.33	7.3E-05	3.5E-03	#N/A	#N/A	#N/A	#N/A	#N/A	#N/A	#N/A	#N/A
TRINITY_DN16680_c0_g3_i9	BI1_ORYSJ	-1.42	2.79	6.0E-05	3.0E-03	#N/A	#N/A	#N/A	#N/A	#N/A	#N/A	#N/A	#N/A
TRINITY_DN17752_c0_g1_i1	LTD_ORYSJ	-1.42	4.59	3.9E-06	2.6E-04	#N/A	#N/A	#N/A	#N/A	#N/A	#N/A	#N/A	#N/A
TRINITY_DN19258_c3_g3_i1	TL19_ARATH	-1.42	5.46	5.6E-06	3.5E-04	#N/A	#N/A	#N/A	#N/A	#N/A	#N/A	#N/A	#N/A
TRINITY_DN16683_c0_g4_i7	COMT1_AMMMJ	-1.42	2.66	1.2E-03	3.6E-02	#N/A	#N/A	#N/A	#N/A	#N/A	#N/A	#N/A	#N/A
TRINITY_DN14805_c0_g1_i1	HS16A_ORYSJ	-1.42	1.45	1.1E-03	3.4E-02	#N/A	#N/A	#N/A	#N/A	#N/A	#N/A	#N/A	#N/A
TRINITY_DN19176_c4_g3_i2	RR10_MESCR	-1.41	1.84	7.7E-04	2.5E-02	#N/A	#N/A	#N/A	#N/A	#N/A	#N/A	#N/A	#N/A
TRINITY_DN15446_c0_g1_i1	CHI11_ORYSJ	-1.41	2.46	9.6E-04	3.0E-02	#N/A	#N/A	#N/A	#N/A	#N/A	#N/A	#N/A	#N/A
TRINITY_DN15520_c0_g1_i5	GDPD1_ARATH	-1.41	2.16	3.1E-04	1.2E-02	#N/A	#N/A	#N/A	#N/A	#N/A	#N/A	#N/A	#N/A
TRINITY_DN20429_c0_g2_i3	ACT_PINCO	-1.41	2.20	5.9E-05	2.9E-03	#N/A	#N/A	#N/A	#N/A	#N/A	#N/A	#N/A	#N/A
TRINITY_DN20414_c1_g3_i3	TRXH5_ORYSJ	-1.40	2.07	4.9E-04	1.8E-02	#N/A	#N/A	#N/A	#N/A	#N/A	#N/A	#N/A	#N/A
TRINITY_DN19333_c0_g4_i2	APS2_ARATH	-1.39	6.00	1.5E-03	4.2E-02	#N/A	#N/A	#N/A	#N/A	#N/A	#N/A	#N/A	#N/A
TRINITY_DN18062_c1_g6_i3	PRF1_SOLLC	-1.38	6.57	6.6E-04	2.2E-02	#N/A	#N/A	#N/A	#N/A	#N/A	#N/A	#N/A	#N/A
TRINITY_DN20894_c0_g3_i2	SBT17_ARATH	-1.38	2.71	8.4E-04	2.7E-02	#N/A	#N/A	#N/A	#N/A	#N/A	#N/A	#N/A	#N/A
TRINITY_DN18965_c1_g4_i6	CALR3_ARATH	-1.38	3.11	6.3E-05	3.1E-03	#N/A	#N/A	#N/A	#N/A	#N/A	#N/A	#N/A	#N/A
TRINITY_DN15274_c3_g1_i3	BAS1_ORYSJ	-1.38	8.84	5.2E-06	3.3E-04	#N/A	#N/A	#N/A	#N/A	#N/A	#N/A	#N/A	#N/A
TRINITY_DN21523_c0_g1_i1	TAL_HISS2	-1.38	3.06	4.4E-04	1.6E-02	#N/A	#N/A	#N/A	#N/A	#N/A	#N/A	#N/A	#N/A
TRINITY_DN18132_c1_g3_i2	RRP2_SPIOL	-1.37	6.47	7.2E-09	8.1E-07	#N/A	#N/A	#N/A	#N/A	#N/A	#N/A	#N/A	#N/A
TRINITY_DN20169_c0_g1_i6	TR120_ORYSJ	-1.37	1.73	4.3E-04	1.6E-02	#N/A	#N/A	#N/A	#N/A	#N/A	#N/A	#N/A	#N/A
TRINITY_DN16782_c0_g2_i5	Y4141_ARATH	-1.37	5.56	1.1E-03	3.2E-02	#N/A	#N/A	#N/A	#N/A	#N/A	#N/A	#N/A	#N/A
TRINITY_DN15106_c0_g2_i2	ACP1_HORVU	-1.37	3.14	2.1E-05	1.2E-03	#N/A	#N/A	#N/A	#N/A	#N/A	#N/A	#N/A	#N/A
TRINITY_DN18316_c0_g1_i9	ACT1_ORYSJ	-1.36	3.37	1.2E-04	5.6E-03	#N/A	#N/A	#N/A	#N/A	#N/A	#N/A	#N/A	#N/A
TRINITY_DN17541_c1_g1_i3	H1_MAIZE	-1.36	3.38	1.0E-03	3.2E-02	#N/A	#N/A	#N/A	#N/A	#N/A	#N/A	#N/A	#N/A
TRINITY_DN16930_c0_g1_i4	ISPD_ORYSJ	-1.36	3.65	1.7E-06	1.3E-04	#N/A	#N/A	#N/A	#N/A	#N/A	#N/A	#N/A	#N/A
TRINITY_DN20766_c1_g1_i5	RSSA_VITVI	-1.35	2.87	1.8E-04	7.5E-03	#N/A	#N/A	#N/A	#N/A	#N/A	#N/A	#N/A	#N/A
TRINITY_DN15026_c0_g1_i2	AAT1_ARATH	-1.35	4.87	2.7E-08	2.8E-06	#N/A	#N/A	#N/A	#N/A	#N/A	#N/A	#N/A	#N/A
TRINITY_DN15434_c0_g1_i4	MENG_ARATH	-1.35	5.02	1.0E-05	6.2E-04	#N/A	#N/A	#N/A	#N/A	#N/A	#N/A	#N/A	#N/A

DET	Subject id	c9TR vs c7NT				c7TR vs c7NT				c7TR vs c9TR			
		Log_2	Log_2CPM	PValue	19FDR	Log_2	Log_2CPM	PValue	19FDR	Log_2	Log_2CPM	PValue	19FDR
TRINITY_DN20328_c0_g1_i5	HYES_PIG	-1.35	3.34	1.4E-05	8.3E-04	#N/A	#N/A	#N/A	#N/A	#N/A	#N/A	#N/A	#N/A
TRINITY_DN17289_c0_g7_i1	CML20_ORYSJ	-1.35	2.42	8.2E-04	2.7E-02	#N/A	#N/A	#N/A	#N/A	#N/A	#N/A	#N/A	#N/A
TRINITY_DN18474_c1_g1_i2	CB4B_ARATH	-1.34	5.31	4.5E-08	4.6E-06	#N/A	#N/A	#N/A	#N/A	#N/A	#N/A	#N/A	#N/A
TRINITY_DN15690_c1_g1_i2	RBS_MAIZE	-1.34	3.27	4.0E-04	1.5E-02	#N/A	#N/A	#N/A	#N/A	#N/A	#N/A	#N/A	#N/A
TRINITY_DN18169_c1_g1_i23	HEM3_ORYSJ	-1.34	2.14	1.5E-04	6.8E-03	#N/A	#N/A	#N/A	#N/A	#N/A	#N/A	#N/A	#N/A
TRINITY_DN20653_c0_g1_i4	SK1_ORYSJ	-1.33	4.77	8.6E-04	2.8E-02	#N/A	#N/A	#N/A	#N/A	#N/A	#N/A	#N/A	#N/A
TRINITY_DN18172_c0_g1_i15	GUAD_BACSU	-1.33	3.69	3.0E-06	2.0E-04	#N/A	#N/A	#N/A	#N/A	#N/A	#N/A	#N/A	#N/A
TRINITY_DN16820_c1_g2_i2	PER51_ARATH	-1.33	4.76	2.3E-04	9.4E-03	#N/A	#N/A	#N/A	#N/A	#N/A	#N/A	#N/A	#N/A
TRINITY_DN15825_c1_g2_i7	TRPC_ARATH	-1.33	5.19	2.1E-03	5.5E-02	#N/A	#N/A	#N/A	#N/A	#N/A	#N/A	#N/A	#N/A
TRINITY_DN15627_c0_g3_i3	CB4A_ARATH	-1.33	6.26	1.0E-06	7.8E-05	#N/A	#N/A	#N/A	#N/A	#N/A	#N/A	#N/A	#N/A
TRINITY_DN18247_c1_g1_i2	STR10_ARATH	-1.33	4.50	2.0E-08	2.2E-06	#N/A	#N/A	#N/A	#N/A	#N/A	#N/A	#N/A	#N/A
TRINITY_DN20040_c0_g1_i8	RS242_ARATH	-1.32	3.62	3.1E-06	2.1E-04	#N/A	#N/A	#N/A	#N/A	#N/A	#N/A	#N/A	#N/A
TRINITY_DN18231_c0_g1_i1	PB27A_ARATH	-1.32	6.04	1.2E-04	5.4E-03	#N/A	#N/A	#N/A	#N/A	#N/A	#N/A	#N/A	#N/A
TRINITY_DN17786_c0_g1_i2	ZSS1_ZINZE	-1.32	5.41	4.7E-04	1.7E-02	#N/A	#N/A	#N/A	#N/A	#N/A	#N/A	#N/A	#N/A
TRINITY_DN19383_c0_g2_i3	RR9_ARATH	-1.32	6.83	4.9E-05	2.5E-03	#N/A	#N/A	#N/A	#N/A	#N/A	#N/A	#N/A	#N/A
TRINITY_DN20806_c0_g1_i7	BGAL5_ORYSJ	-1.32	2.91	1.0E-03	3.2E-02	#N/A	#N/A	#N/A	#N/A	#N/A	#N/A	#N/A	#N/A
TRINITY_DN20781_c0_g2_i3	FK163_ARATH	-1.31	5.67	1.2E-06	9.3E-05	#N/A	#N/A	#N/A	#N/A	#N/A	#N/A	#N/A	#N/A
TRINITY_DN21143_c0_g2_i12	DAPB3_ARATH	-1.31	4.09	9.3E-05	4.3E-03	#N/A	#N/A	#N/A	#N/A	#N/A	#N/A	#N/A	#N/A
TRINITY_DN22206_c1_g2_i12	PP332_ARATH	-1.30	2.59	4.7E-04	1.7E-02	#N/A	#N/A	#N/A	#N/A	#N/A	#N/A	#N/A	#N/A
TRINITY_DN19446_c0_g1_i5	SAMC1_ARATH	-1.30	3.59	1.3E-03	3.8E-02	#N/A	#N/A	#N/A	#N/A	#N/A	#N/A	#N/A	#N/A
TRINITY_DN19196_c1_g4_i3	RK24_TOBAC	-1.30	8.10	2.0E-05	1.1E-03	#N/A	#N/A	#N/A	#N/A	#N/A	#N/A	#N/A	#N/A
TRINITY_DN19029_c0_g3_i3	AB12I_ARATH	-1.30	2.06	1.6E-03	4.4E-02	#N/A	#N/A	#N/A	#N/A	#N/A	#N/A	#N/A	#N/A
TRINITY_DN13714_c0_g1_i5	CUT1B_ARATH	-1.30	7.61	9.0E-04	2.9E-02	#N/A	#N/A	#N/A	#N/A	#N/A	#N/A	#N/A	#N/A
TRINITY_DN16165_c0_g1_i5	Y2766_ARATH	-1.29	7.08	1.1E-03	3.3E-02	#N/A	#N/A	#N/A	#N/A	#N/A	#N/A	#N/A	#N/A
TRINITY_DN14636_c0_g1_i15	CAHC_HORVU	-1.29	5.23	1.8E-05	1.0E-03	#N/A	#N/A	#N/A	#N/A	#N/A	#N/A	#N/A	#N/A
TRINITY_DN18569_c0_g1_i10	FER5_MAIZE	-1.29	6.52	7.6E-07	5.9E-05	#N/A	#N/A	#N/A	#N/A	#N/A	#N/A	#N/A	#N/A
TRINITY_DN22812_c3_g1_i7	NDK2_TOBAC	-1.28	3.99	3.2E-07	2.7E-05	#N/A	#N/A	#N/A	#N/A	#N/A	#N/A	#N/A	#N/A
TRINITY_DN21815_c0_g1_i1	GLB3_ARATH	-1.28	3.81	1.4E-03	3.9E-02	#N/A	#N/A	#N/A	#N/A	#N/A	#N/A	#N/A	#N/A
TRINITY_DN18484_c5_g4_i1	PTA16_ARATH	-1.28	2.46	5.0E-04	1.8E-02	#N/A	#N/A	#N/A	#N/A	#N/A	#N/A	#N/A	#N/A
TRINITY_DN15014_c0_g2_i5	SYVM2_ARATH	-1.28	2.49	1.7E-03	4.7E-02	#N/A	#N/A	#N/A	#N/A	#N/A	#N/A	#N/A	#N/A
TRINITY_DN17852_c1_g1_i1	HIS1_ORYSJ	-1.27	4.84	1.0E-06	7.8E-05	#N/A	#N/A	#N/A	#N/A	#N/A	#N/A	#N/A	#N/A
TRINITY_DN16623_c0_g2_i2	ASA1_ORYSI	-1.27	3.29	1.7E-04	7.2E-03	#N/A	#N/A	#N/A	#N/A	#N/A	#N/A	#N/A	#N/A
TRINITY_DN15627_c0_g3_i5	CB4A_ARATH	-1.27	9.52	1.1E-05	6.7E-04	#N/A	#N/A	#N/A	#N/A	#N/A	#N/A	#N/A	#N/A
TRINITY_DN15379_c0_g1_i5	SR43C_ORYSJ	-1.27	3.93	7.4E-05	3.6E-03	#N/A	#N/A	#N/A	#N/A	#N/A	#N/A	#N/A	#N/A
TRINITY_DN16920_c1_g2_i2	F16P1_SPIOL	-1.27	3.79	3.6E-06	2.4E-04	#N/A	#N/A	#N/A	#N/A	#N/A	#N/A	#N/A	#N/A
TRINITY_DN21955_c1_g2_i7	ERDL6_ARATH	-1.27	4.76	2.8E-04	1.1E-02	#N/A	#N/A	#N/A	#N/A	#N/A	#N/A	#N/A	#N/A
TRINITY_DN19870_c0_g1_i9	GGT2_ARATH	-1.27	6.05	9.3E-05	4.3E-03	#N/A	#N/A	#N/A	#N/A	#N/A	#N/A	#N/A	#N/A
TRINITY_DN19176_c4_g3_i3	RR10_MESCR	-1.26	4.41	1.4E-06	1.0E-04	#N/A	#N/A	#N/A	#N/A	#N/A	#N/A	#N/A	#N/A
TRINITY_DN17160_c1_g3_i1	MAVI_CUCPE	-1.26	4.97	3.4E-06	2.3E-04	#N/A	#N/A	#N/A	#N/A	#N/A	#N/A	#N/A	#N/A
TRINITY_DN16828_c0_g2_i5	CCR1_ARATH	-1.25	4.25	3.1E-04	1.2E-02	#N/A	#N/A	#N/A	#N/A	#N/A	#N/A	#N/A	#N/A
TRINITY_DN17772_c0_g1_i8	GPDL4_ARATH	-1.25	3.68	5.1E-05	2.6E-03	#N/A	#N/A	#N/A	#N/A	#N/A	#N/A	#N/A	#N/A
TRINITY_DN21760_c2_g1_i6	KEA6_ARATH	-1.24	2.17	1.9E-03	5.1E-02	#N/A	#N/A	#N/A	#N/A	#N/A	#N/A	#N/A	#N/A
TRINITY_DN17754_c0_g1_i2	SPA_SOLLC	-1.24	7.73	1.4E-05	8.0E-04	#N/A	#N/A	#N/A	#N/A	#N/A	#N/A	#N/A	#N/A
TRINITY_DN18103_c0_g3_i6	FABG3_BRANA	-1.24	5.27	3.0E-06	2.0E-04	#N/A	#N/A	#N/A	#N/A	#N/A	#N/A	#N/A	#N/A
TRINITY_DN20029_c2_g1_i1	DIT1_SPIOL	-1.24	6.91	1.8E-06	1.3E-04	#N/A	#N/A	#N/A	#N/A	#N/A	#N/A	#N/A	#N/A
TRINITY_DN22028_c0_g1_i5	CNIF1_ARATH	-1.24	4.10	1.1E-04	5.2E-03	#N/A	#N/A	#N/A	#N/A	#N/A	#N/A	#N/A	#N/A
TRINITY_DN22058_c1_g1_i4	HSP7S_PEA	-1.24	3.77	2.4E-04	9.7E-03	#N/A	#N/A	#N/A	#N/A	#N/A	#N/A	#N/A	#N/A
TRINITY_DN19851_c2_g4_i1	FAD3C_RICCO	-1.24	7.00	4.9E-07	4.0E-05	#N/A	#N/A	#N/A	#N/A	#N/A	#N/A	#N/A	#N/A
TRINITY_DN14888_c0_g1_i3	FRS5_ARATH	-1.24	2.18	1.1E-03	3.3E-02	#N/A	#N/A	#N/A	#N/A	#N/A	#N/A	#N/A	#N/A
TRINITY_DN19525_c0_g1_i3	PEAM1_ARATH	-1.23	2.60	4.8E-04	1.7E-02	#N/A	#N/A	#N/A	#N/A	#N/A	#N/A	#N/A	#N/A
TRINITY_DN20141_c3_g2_i14	FZL_ARATH	-1.23	5.59	5.2E-04	1.8E-02	#N/A	#N/A	#N/A	#N/A	#N/A	#N/A	#N/A	#N/A
TRINITY_DN21653_c1_g2_i6	STRAP_DICDI	-1.23	2.73	2.3E-04	9.5E-03	#N/A	#N/A	#N/A	#N/A	#N/A	#N/A	#N/A	#N/A
TRINITY_DN13792_c0_g1_i2	PPR3_ARATH	-1.23	2.79	1.3E-03	3.8E-02	#N/A	#N/A	#N/A	#N/A	#N/A	#N/A	#N/A	#N/A
TRINITY_DN21207_c4_g2_i15	ARF_DUGJA	-1.23	3.05	3.7E-04	1.4E-02	#N/A	#N/A	#N/A	#N/A	#N/A	#N/A	#N/A	#N/A
TRINITY_DN16293_c4_g2_i13	RR6_ARATH	-1.23	5.19	1.4E-05	8.1E-04	#N/A	#N/A	#N/A	#N/A	#N/A	#N/A	#N/A	#N/A
TRINITY_DN16910_c1_g2_i4	TL15A_ARATH	-1.22	5.82	4.1E-07	3.4E-05	#N/A	#N/A	#N/A	#N/A	#N/A	#N/A	#N/A	#N/A
TRINITY_DN22142_c1_g1_i17	STR4_ARATH	-1.22	8.72	2.1E-07	1.9E-05	#N/A	#N/A	#N/A	#N/A	#N/A	#N/A	#N/A	#N/A
TRINITY_DN20678_c0_g1_i11	ERG1_PANGI	-1.22	6.72	1.9E-07	1.7E-05	#N/A	#N/A	#N/A	#N/A	#N/A	#N/A	#N/A	#N/A
TRINITY_DN18959_c0_g1_i5	NPR4_ARATH	-1.22	3.22	1.7E-03	4.7E-02	#N/A	#N/A	#N/A	#N/A	#N/A	#N/A	#N/A	#N/A
TRINITY_DN17668_c1_g1_i1	PP145_ARATH	-1.22	2.27	6.1E-04	2.1E-02	#N/A	#N/A	#N/A	#N/A	#N/A	#N/A	#N/A	#N/A
TRINITY_DN13792_c0_g1_i1	PPR3_ARATH	-1.22	4.58	1.7E-06	1.2E-04	#N/A	#N/A	#N/A	#N/A	#N/A	#N/A	#N/A	#N/A

DET	Subject id	c9TR vs c7NT				c7TR vs c7NT				c7TR vs c9TR			
		Log_2	Log_2CPM	PValue	19FDR	Log_2	Log_2CPM	PValue	19FDR	Log_2	Log_2CPM	PValue	19FDR
TRINITY_DN18169_c1_g1_i16	HEM3_ORYSJ	-1.22	5.17	5.2E-06	3.4E-04	#N/A	#N/A	#N/A	#N/A	#N/A	#N/A	#N/A	#N/A
TRINITY_DN16153_c0_g1_i15	SYWM_ARATH	-1.22	4.59	1.5E-03	4.2E-02	#N/A	#N/A	#N/A	#N/A	#N/A	#N/A	#N/A	#N/A
TRINITY_DN19281_c1_g1_i3	P4H3_ARATH	-1.21	3.46	1.1E-05	6.7E-04	#N/A	#N/A	#N/A	#N/A	#N/A	#N/A	#N/A	#N/A
TRINITY_DN14636_c0_g1_i4	CAHC_HORVU	-1.21	5.72	4.5E-08	4.5E-06	#N/A	#N/A	#N/A	#N/A	#N/A	#N/A	#N/A	#N/A
TRINITY_DN22461_c1_g5_i1	PGKH_WHEAT	-1.21	4.48	1.1E-03	3.4E-02	#N/A	#N/A	#N/A	#N/A	#N/A	#N/A	#N/A	#N/A
TRINITY_DN18187_c2_g1_i2	CLPR2_ARATH	-1.21	6.19	4.2E-04	1.5E-02	#N/A	#N/A	#N/A	#N/A	#N/A	#N/A	#N/A	#N/A
TRINITY_DN20605_c1_g3_i1	CP29B_ARATH	-1.21	6.29	6.5E-07	5.2E-05	#N/A	#N/A	#N/A	#N/A	#N/A	#N/A	#N/A	#N/A
TRINITY_DN17576_c0_g1_i3	CLPT1_ARATH	-1.21	3.54	4.8E-05	2.5E-03	#N/A	#N/A	#N/A	#N/A	#N/A	#N/A	#N/A	#N/A
TRINITY_DN15690_c1_g1_i1	RBS_MAIZE	-1.20	10.40	2.9E-04	1.1E-02	#N/A	#N/A	#N/A	#N/A	#N/A	#N/A	#N/A	#N/A
TRINITY_DN16153_c0_g1_i5	SYWM_ARATH	-1.20	2.95	1.2E-03	3.6E-02	#N/A	#N/A	#N/A	#N/A	#N/A	#N/A	#N/A	#N/A
TRINITY_DN16183_c1_g1_i5	RK6_ARATH	-1.20	6.26	1.6E-05	9.0E-04	#N/A	#N/A	#N/A	#N/A	#N/A	#N/A	#N/A	#N/A
TRINITY_DN21543_c0_g1_i13	Y005_SYNY3	-1.20	9.83	2.0E-03	5.4E-02	#N/A	#N/A	#N/A	#N/A	#N/A	#N/A	#N/A	#N/A
TRINITY_DN17148_c1_g2_i13	PSBY_SPIOL	-1.20	4.11	9.7E-05	4.5E-03	#N/A	#N/A	#N/A	#N/A	#N/A	#N/A	#N/A	#N/A
TRINITY_DN18572_c0_g1_i12	AB25B_ORYSJ	-1.19	3.51	3.0E-05	1.6E-03	#N/A	#N/A	#N/A	#N/A	#N/A	#N/A	#N/A	#N/A
TRINITY_DN22404_c1_g2_i9	HEI10_ORYSJ	-1.19	2.68	1.5E-04	6.5E-03	#N/A	#N/A	#N/A	#N/A	#N/A	#N/A	#N/A	#N/A
TRINITY_DN15935_c1_g2_i7	RK9_WHEAT	-1.19	8.27	2.4E-04	9.9E-03	#N/A	#N/A	#N/A	#N/A	#N/A	#N/A	#N/A	#N/A
TRINITY_DN19707_c2_g1_i17	HPSE3_ARATH	-1.19	3.48	1.4E-03	3.9E-02	#N/A	#N/A	#N/A	#N/A	#N/A	#N/A	#N/A	#N/A
TRINITY_DN17481_c1_g4_i1	RK29_MAIZE	-1.19	6.86	1.8E-04	7.7E-03	#N/A	#N/A	#N/A	#N/A	#N/A	#N/A	#N/A	#N/A
TRINITY_DN15274_c3_g1_i5	BAS1_ORYSJ	-1.18	7.11	1.0E-05	6.1E-04	#N/A	#N/A	#N/A	#N/A	#N/A	#N/A	#N/A	#N/A
TRINITY_DN19110_c0_g2_i2	TR164_ORYSJ	-1.18	6.17	1.7E-04	7.4E-03	#N/A	#N/A	#N/A	#N/A	#N/A	#N/A	#N/A	#N/A
TRINITY_DN13344_c0_g1_i5	PLCD2_ARATH	-1.18	3.43	9.4E-04	2.9E-02	#N/A	#N/A	#N/A	#N/A	#N/A	#N/A	#N/A	#N/A
TRINITY_DN14758_c0_g2_i8	PNSL5_ARATH	-1.18	6.36	1.5E-03	4.1E-02	#N/A	#N/A	#N/A	#N/A	#N/A	#N/A	#N/A	#N/A
TRINITY_DN15469_c2_g2_i4	PNSL2_ARATH	-1.18	4.83	2.9E-05	1.6E-03	#N/A	#N/A	#N/A	#N/A	#N/A	#N/A	#N/A	#N/A
TRINITY_DN22270_c0_g2_i4	PLGG1_ARATH	-1.18	3.92	3.0E-05	1.6E-03	#N/A	#N/A	#N/A	#N/A	#N/A	#N/A	#N/A	#N/A
TRINITY_DN17017_c1_g2_i1	CP41A_ARATH	-1.18	5.38	1.7E-04	7.2E-03	#N/A	#N/A	#N/A	#N/A	#N/A	#N/A	#N/A	#N/A
TRINITY_DN19376_c1_g7_i2	RK18_ORYSJ	-1.18	7.25	1.9E-05	1.1E-03	#N/A	#N/A	#N/A	#N/A	#N/A	#N/A	#N/A	#N/A
TRINITY_DN21597_c2_g1_i3	CHLI_ORYSJ	-1.17	7.97	6.3E-05	3.1E-03	#N/A	#N/A	#N/A	#N/A	#N/A	#N/A	#N/A	#N/A
TRINITY_DN17386_c0_g2_i1	EFP_MICAN	-1.17	6.58	7.6E-04	2.5E-02	#N/A	#N/A	#N/A	#N/A	#N/A	#N/A	#N/A	#N/A
TRINITY_DN16452_c0_g1_i7	H1_MAIZE	-1.17	3.89	2.4E-04	9.9E-03	#N/A	#N/A	#N/A	#N/A	#N/A	#N/A	#N/A	#N/A
TRINITY_DN19462_c0_g1_i9	TM1L2_MOUSE	-1.17	2.79	1.0E-04	4.8E-03	#N/A	#N/A	#N/A	#N/A	#N/A	#N/A	#N/A	#N/A
TRINITY_DN21694_c1_g1_i5	CIPK9_ORYSJ	-1.17	5.47	2.9E-04	1.1E-02	#N/A	#N/A	#N/A	#N/A	#N/A	#N/A	#N/A	#N/A
TRINITY_DN20605_c1_g4_i2	ROC2_NICSY	-1.16	4.69	9.3E-05	4.3E-03	#N/A	#N/A	#N/A	#N/A	#N/A	#N/A	#N/A	#N/A
TRINITY_DN17030_c3_g2_i2	FAD3D_ARATH	-1.16	5.92	5.5E-06	3.5E-04	#N/A	#N/A	#N/A	#N/A	#N/A	#N/A	#N/A	#N/A
TRINITY_DN17148_c0_g1_i1	GUN4C_ARATH	-1.16	8.12	5.6E-05	2.8E-03	#N/A	#N/A	#N/A	#N/A	#N/A	#N/A	#N/A	#N/A
TRINITY_DN18705_c0_g1_i14	BGL05_ORYSJ	-1.16	4.17	9.4E-05	4.4E-03	#N/A	#N/A	#N/A	#N/A	#N/A	#N/A	#N/A	#N/A
TRINITY_DN18355_c0_g1_i3	CHLM_ARATH	-1.16	4.77	3.0E-04	1.2E-02	#N/A	#N/A	#N/A	#N/A	#N/A	#N/A	#N/A	#N/A
TRINITY_DN19997_c0_g2_i8	PPR68_ARATH	-1.16	5.55	4.7E-04	1.7E-02	#N/A	#N/A	#N/A	#N/A	#N/A	#N/A	#N/A	#N/A
TRINITY_DN19411_c1_g2_i1	PTA10_ARATH	-1.16	4.81	2.3E-07	2.0E-05	#N/A	#N/A	#N/A	#N/A	#N/A	#N/A	#N/A	#N/A
TRINITY_DN22460_c1_g1_i2	ENO1_MAIZE	-1.16	8.80	1.4E-04	6.1E-03	#N/A	#N/A	#N/A	#N/A	#N/A	#N/A	#N/A	#N/A
TRINITY_DN20009_c0_g2_i7	SCE1_ARATH	-1.16	5.36	6.6E-06	4.1E-04	#N/A	#N/A	#N/A	#N/A	#N/A	#N/A	#N/A	#N/A
TRINITY_DN20029_c2_g1_i4	DIT1_SPIOL	-1.15	7.17	1.9E-04	7.9E-03	#N/A	#N/A	#N/A	#N/A	#N/A	#N/A	#N/A	#N/A
TRINITY_DN15633_c0_g1_i1	CCB1_ARATH	-1.15	6.29	3.0E-04	1.2E-02	#N/A	#N/A	#N/A	#N/A	#N/A	#N/A	#N/A	#N/A
TRINITY_DN4307_c0_g1_i1	IBBR_ORYSI	-1.15	6.48	1.7E-06	1.2E-04	#N/A	#N/A	#N/A	#N/A	#N/A	#N/A	#N/A	#N/A
TRINITY_DN17648_c0_g7_i1	RR17_MAIZE	-1.15	6.95	4.1E-05	2.1E-03	#N/A	#N/A	#N/A	#N/A	#N/A	#N/A	#N/A	#N/A
TRINITY_DN20054_c0_g1_i16	PMTF_ARATH	-1.15	3.16	1.8E-03	4.8E-02	#N/A	#N/A	#N/A	#N/A	#N/A	#N/A	#N/A	#N/A
TRINITY_DN21418_c0_g3_i1	GLN11_ORYSJ	-1.14	6.47	1.1E-03	3.3E-02	#N/A	#N/A	#N/A	#N/A	#N/A	#N/A	#N/A	#N/A
TRINITY_DN16108_c0_g1_i8	PNSB1_ARATH	-1.14	6.20	3.5E-07	3.0E-05	#N/A	#N/A	#N/A	#N/A	#N/A	#N/A	#N/A	#N/A
TRINITY_DN20411_c0_g1_i1	PGKH_WHEAT	-1.14	7.57	1.4E-04	6.2E-03	#N/A	#N/A	#N/A	#N/A	#N/A	#N/A	#N/A	#N/A
TRINITY_DN15158_c1_g5_i2	WAK2_ARATH	-1.14	4.27	1.0E-04	4.8E-03	#N/A	#N/A	#N/A	#N/A	#N/A	#N/A	#N/A	#N/A
TRINITY_DN14482_c0_g1_i3	PPD1_ARATH	-1.14	6.23	5.4E-05	2.7E-03	#N/A	#N/A	#N/A	#N/A	#N/A	#N/A	#N/A	#N/A
TRINITY_DN16583_c0_g6_i2	RUBA_WHEAT	-1.14	7.54	3.3E-05	1.8E-03	#N/A	#N/A	#N/A	#N/A	#N/A	#N/A	#N/A	#N/A
TRINITY_DN21994_c0_g1_i23	YCHF_BACSU	-1.14	3.84	1.4E-03	3.9E-02	#N/A	#N/A	#N/A	#N/A	#N/A	#N/A	#N/A	#N/A
TRINITY_DN14578_c0_g1_i3	RL9_ORYSJ	-1.14	5.22	9.9E-07	7.5E-05	#N/A	#N/A	#N/A	#N/A	#N/A	#N/A	#N/A	#N/A
TRINITY_DN19812_c2_g1_i1	CB26_PETSP	-1.14	3.27	1.3E-04	5.8E-03	#N/A	#N/A	#N/A	#N/A	#N/A	#N/A	#N/A	#N/A
TRINITY_DN17007_c0_g5_i1	PLSP1_ARATH	-1.14	5.08	4.1E-07	3.4E-05	#N/A	#N/A	#N/A	#N/A	#N/A	#N/A	#N/A	#N/A
TRINITY_DN22315_c0_g1_i12	HEM1_ORYSJ	-1.13	3.38	1.9E-04	8.2E-03	#N/A	#N/A	#N/A	#N/A	#N/A	#N/A	#N/A	#N/A
TRINITY_DN17370_c0_g1_i5	CLPP5_ARATH	-1.13	4.19	5.9E-05	2.9E-03	#N/A	#N/A	#N/A	#N/A	#N/A	#N/A	#N/A	#N/A
TRINITY_DN15106_c0_g1_i2	ACP1_HORVU	-1.13	3.56	5.2E-05	2.6E-03	#N/A	#N/A	#N/A	#N/A	#N/A	#N/A	#N/A	#N/A
TRINITY_DN18341_c0_g2_i1	RMLCD_ARATH	-1.12	5.19	1.3E-04	6.0E-03	#N/A	#N/A	#N/A	#N/A	#N/A	#N/A	#N/A	#N/A
TRINITY_DN16730_c0_g1_i5	RL23_ARATH	-1.12	3.99	3.9E-06	2.6E-04	#N/A	#N/A	#N/A	#N/A	#N/A	#N/A	#N/A	#N/A
TRINITY_DN15037_c0_g1_i6	ELOC_RAT	-1.12	2.50	8.8E-04	2.8E-02	#N/A	#N/A	#N/A	#N/A	#N/A	#N/A	#N/A	#N/A

DET	Subject id	c9TR vs c7NT				c7TR vs c7NT				c7TR vs c9TR			
		Log_2	Log_2CPM	PValue	19FDR	Log_2	Log_2CPM	PValue	19FDR	Log_2	Log_2CPM	PValue	19FDR
TRINITY_DN17108_c0_g1_i6	PGKH1_ARATH	-1.12	5.11	1.2E-05	7.2E-04	#N/A	#N/A	#N/A	#N/A	#N/A	#N/A	#N/A	#N/A
TRINITY_DN22279_c2_g2_i5	K502_ACTDE	-1.12	6.59	1.4E-06	1.0E-04	#N/A	#N/A	#N/A	#N/A	#N/A	#N/A	#N/A	#N/A
TRINITY_DN19812_c1_g3_i2	CB23_ORYSJ	-1.11	11.83	2.2E-06	1.5E-04	#N/A	#N/A	#N/A	#N/A	#N/A	#N/A	#N/A	#N/A
TRINITY_DN16031_c0_g6_i1	GSA_ORYSJ	-1.11	7.60	6.3E-07	5.0E-05	#N/A	#N/A	#N/A	#N/A	#N/A	#N/A	#N/A	#N/A
TRINITY_DN20462_c0_g2_i1	RS92_ARATH	-1.11	3.30	5.9E-04	2.0E-02	#N/A	#N/A	#N/A	#N/A	#N/A	#N/A	#N/A	#N/A
TRINITY_DN18725_c1_g3_i2	RL321_ARATH	-1.11	3.51	4.7E-04	1.7E-02	#N/A	#N/A	#N/A	#N/A	#N/A	#N/A	#N/A	#N/A
TRINITY_DN22135_c0_g1_i20	PHL1_ARATH	-1.10	7.09	1.5E-03	4.3E-02	#N/A	#N/A	#N/A	#N/A	#N/A	#N/A	#N/A	#N/A
TRINITY_DN22795_c6_g1_i7	PORB_HORVU	-1.10	8.20	1.8E-06	1.3E-04	#N/A	#N/A	#N/A	#N/A	#N/A	#N/A	#N/A	#N/A
TRINITY_DN20017_c1_g4_i10	RK13_SPIOL	-1.10	5.83	5.4E-05	2.7E-03	#N/A	#N/A	#N/A	#N/A	#N/A	#N/A	#N/A	#N/A
TRINITY_DN16383_c3_g4_i2	RK15_PEA	-1.10	6.98	6.3E-05	3.1E-03	#N/A	#N/A	#N/A	#N/A	#N/A	#N/A	#N/A	#N/A
TRINITY_DN21597_c2_g1_i7	CHLI_ORYSJ	-1.10	4.92	2.7E-05	1.5E-03	#N/A	#N/A	#N/A	#N/A	#N/A	#N/A	#N/A	#N/A
TRINITY_DN20141_c3_g2_i1	FZL_ARATH	-1.10	4.13	3.1E-04	1.2E-02	#N/A	#N/A	#N/A	#N/A	#N/A	#N/A	#N/A	#N/A
TRINITY_DN15735_c0_g3_i2	PPR32_ARATH	-1.10	3.11	2.4E-04	9.7E-03	#N/A	#N/A	#N/A	#N/A	#N/A	#N/A	#N/A	#N/A
TRINITY_DN15773_c2_g1_i6	CB21_MAIZE	-1.10	3.87	1.9E-04	7.9E-03	#N/A	#N/A	#N/A	#N/A	#N/A	#N/A	#N/A	#N/A
TRINITY_DN19906_c0_g1_i3	STR9_ARATH	-1.09	6.48	3.2E-06	2.1E-04	#N/A	#N/A	#N/A	#N/A	#N/A	#N/A	#N/A	#N/A
TRINITY_DN16650_c1_g12_i1	U85A2_ARATH	-1.09	4.73	1.8E-04	7.6E-03	#N/A	#N/A	#N/A	#N/A	#N/A	#N/A	#N/A	#N/A
TRINITY_DN17946_c0_g2_i1	DHAS_SYNY3	-1.09	2.98	5.4E-04	1.9E-02	#N/A	#N/A	#N/A	#N/A	#N/A	#N/A	#N/A	#N/A
TRINITY_DN19210_c1_g3_i1	GAP1_ORYSJ	-1.09	3.77	5.3E-04	1.9E-02	#N/A	#N/A	#N/A	#N/A	#N/A	#N/A	#N/A	#N/A
TRINITY_DN15934_c3_g3_i15	IAA10_ORYSJ	-1.09	3.66	2.2E-04	9.2E-03	#N/A	#N/A	#N/A	#N/A	#N/A	#N/A	#N/A	#N/A
TRINITY_DN14619_c0_g2_i1	RS16_ORYSJ	-1.09	4.59	4.8E-06	3.1E-04	#N/A	#N/A	#N/A	#N/A	#N/A	#N/A	#N/A	#N/A
TRINITY_DN16305_c0_g1_i7	YLMG2_ARATH	-1.09	4.57	6.7E-06	4.2E-04	#N/A	#N/A	#N/A	#N/A	#N/A	#N/A	#N/A	#N/A
TRINITY_DN16286_c0_g4_i1	HPT1_ORYSJ	-1.09	5.05	7.2E-07	5.6E-05	#N/A	#N/A	#N/A	#N/A	#N/A	#N/A	#N/A	#N/A
TRINITY_DN17030_c3_g2_i1	FAD3C_SESIN	-1.09	5.44	2.3E-06	1.6E-04	#N/A	#N/A	#N/A	#N/A	#N/A	#N/A	#N/A	#N/A
TRINITY_DN17030_c3_g2_i5	FAD3C_SESIN	-1.09	5.43	5.1E-06	3.3E-04	#N/A	#N/A	#N/A	#N/A	#N/A	#N/A	#N/A	#N/A
TRINITY_DN18701_c0_g1_i14	RK12_ORYSJ	-1.09	4.86	6.4E-04	2.2E-02	#N/A	#N/A	#N/A	#N/A	#N/A	#N/A	#N/A	#N/A
TRINITY_DN15407_c0_g3_i5	DIT21_ARATH	-1.09	7.56	1.2E-05	7.3E-04	#N/A	#N/A	#N/A	#N/A	#N/A	#N/A	#N/A	#N/A
TRINITY_DN20572_c3_g1_i4	BOLA4_ARATH	-1.08	4.44	1.9E-04	8.2E-03	#N/A	#N/A	#N/A	#N/A	#N/A	#N/A	#N/A	#N/A
TRINITY_DN19202_c0_g1_i17	ATPD_SORBI	-1.08	6.10	4.8E-04	1.7E-02	#N/A	#N/A	#N/A	#N/A	#N/A	#N/A	#N/A	#N/A
TRINITY_DN16108_c0_g1_i6	PNSB1_ARATH	-1.08	4.36	3.0E-04	1.2E-02	#N/A	#N/A	#N/A	#N/A	#N/A	#N/A	#N/A	#N/A
TRINITY_DN20499_c0_g2_i1	SGRL_ORYSJ	-1.08	7.00	1.9E-05	1.1E-03	#N/A	#N/A	#N/A	#N/A	#N/A	#N/A	#N/A	#N/A
TRINITY_DN15176_c0_g5_i2	GLPT1_ARATH	-1.08	2.76	1.4E-03	4.0E-02	#N/A	#N/A	#N/A	#N/A	#N/A	#N/A	#N/A	#N/A
TRINITY_DN19434_c0_g2_i2	MTN1_ARATH	-1.08	3.02	9.0E-04	2.9E-02	#N/A	#N/A	#N/A	#N/A	#N/A	#N/A	#N/A	#N/A
TRINITY_DN13274_c0_g1_i2	GRPE_THEEB	-1.08	4.39	1.3E-05	7.6E-04	#N/A	#N/A	#N/A	#N/A	#N/A	#N/A	#N/A	#N/A
TRINITY_DN20609_c1_g1_i20	MSP1_YEAST	-1.08	3.28	1.7E-03	4.7E-02	#N/A	#N/A	#N/A	#N/A	#N/A	#N/A	#N/A	#N/A
TRINITY_DN19237_c0_g1_i10	PPD5_ARATH	-1.07	5.54	5.3E-04	1.9E-02	#N/A	#N/A	#N/A	#N/A	#N/A	#N/A	#N/A	#N/A
TRINITY_DN19678_c0_g1_i18	TON2_ARATH	-1.07	4.42	3.6E-04	1.4E-02	#N/A	#N/A	#N/A	#N/A	#N/A	#N/A	#N/A	#N/A
TRINITY_DN22026_c0_g4_i8	FAD3C_SESIN	-1.07	4.51	6.8E-04	2.3E-02	#N/A	#N/A	#N/A	#N/A	#N/A	#N/A	#N/A	#N/A
TRINITY_DN20411_c0_g1_i2	PGKH_WHEAT	-1.07	7.61	5.5E-06	3.5E-04	#N/A	#N/A	#N/A	#N/A	#N/A	#N/A	#N/A	#N/A
TRINITY_DN17676_c0_g1_i19	SERA1_ARATH	-1.07	4.91	3.0E-06	2.0E-04	#N/A	#N/A	#N/A	#N/A	#N/A	#N/A	#N/A	#N/A
TRINITY_DN21597_c2_g1_i1	CHLI_ORYSJ	-1.06	4.74	3.1E-05	1.7E-03	#N/A	#N/A	#N/A	#N/A	#N/A	#N/A	#N/A	#N/A
TRINITY_DN20165_c0_g5_i1	DIT21_ARATH	-1.06	6.06	2.2E-04	9.0E-03	#N/A	#N/A	#N/A	#N/A	#N/A	#N/A	#N/A	#N/A
TRINITY_DN15091_c0_g2_i2	NACA2_ARATH	-1.06	4.43	3.2E-04	1.2E-02	#N/A	#N/A	#N/A	#N/A	#N/A	#N/A	#N/A	#N/A
TRINITY_DN10925_c0_g1_i1	DBR_TOBAC	-1.06	5.24	1.2E-05	7.3E-04	#N/A	#N/A	#N/A	#N/A	#N/A	#N/A	#N/A	#N/A
TRINITY_DN22803_c2_g4_i1	OMT1_ORYSJ	-1.06	2.74	1.6E-03	4.5E-02	#N/A	#N/A	#N/A	#N/A	#N/A	#N/A	#N/A	#N/A
TRINITY_DN15013_c0_g3_i5	CK5P1_ORYSJ	-1.06	5.83	2.3E-06	1.6E-04	#N/A	#N/A	#N/A	#N/A	#N/A	#N/A	#N/A	#N/A
TRINITY_DN19477_c1_g2_i1	CB4B_SOLLC	-1.06	7.84	6.1E-04	2.1E-02	#N/A	#N/A	#N/A	#N/A	#N/A	#N/A	#N/A	#N/A
TRINITY_DN20165_c0_g6_i1	DIT21_ARATH	-1.06	5.52	2.8E-05	1.6E-03	#N/A	#N/A	#N/A	#N/A	#N/A	#N/A	#N/A	#N/A
TRINITY_DN20017_c1_g4_i7	RK13_ARATH	-1.06	7.78	8.5E-04	2.7E-02	#N/A	#N/A	#N/A	#N/A	#N/A	#N/A	#N/A	#N/A
TRINITY_DN18653_c0_g4_i13	APX2_ORYSJ	-1.06	6.30	2.2E-05	1.2E-03	#N/A	#N/A	#N/A	#N/A	#N/A	#N/A	#N/A	#N/A
TRINITY_DN17787_c0_g1_i2	RH25_ORYSJ	-1.05	4.66	3.2E-04	1.2E-02	#N/A	#N/A	#N/A	#N/A	#N/A	#N/A	#N/A	#N/A
TRINITY_DN17522_c0_g2_i5	PSAG_HORVU	-1.05	7.41	1.9E-06	1.4E-04	#N/A	#N/A	#N/A	#N/A	#N/A	#N/A	#N/A	#N/A
TRINITY_DN19477_c1_g2_i17	CB4B_SOLLC	-1.05	9.14	2.1E-05	1.2E-03	#N/A	#N/A	#N/A	#N/A	#N/A	#N/A	#N/A	#N/A
TRINITY_DN18965_c1_g4_i1	CALR3_ARATH	-1.05	7.96	3.0E-04	1.2E-02	#N/A	#N/A	#N/A	#N/A	#N/A	#N/A	#N/A	#N/A
TRINITY_DN22280_c1_g2_i6	DNJH2_ALLPO	-1.05	6.91	6.7E-04	2.2E-02	#N/A	#N/A	#N/A	#N/A	#N/A	#N/A	#N/A	#N/A
TRINITY_DN21114_c0_g2_i3	HEM6_ORYSJ	-1.04	5.54	2.9E-05	1.6E-03	#N/A	#N/A	#N/A	#N/A	#N/A	#N/A	#N/A	#N/A
TRINITY_DN16322_c0_g1_i7	CYP38_ARATH	-1.04	8.03	4.9E-05	2.5E-03	#N/A	#N/A	#N/A	#N/A	#N/A	#N/A	#N/A	#N/A
TRINITY_DN16969_c1_g4_i2	C99A2_ORYSJ	-1.04	5.95	3.5E-05	1.9E-03	#N/A	#N/A	#N/A	#N/A	#N/A	#N/A	#N/A	#N/A
TRINITY_DN20046_c1_g1_i2	H2B2_WHEAT	-1.04	3.41	8.0E-05	3.8E-03	#N/A	#N/A	#N/A	#N/A	#N/A	#N/A	#N/A	#N/A
TRINITY_DN20524_c0_g4_i2	CB2D_SOLLC	-1.04	9.67	7.6E-05	3.6E-03	#N/A	#N/A	#N/A	#N/A	#N/A	#N/A	#N/A	#N/A
TRINITY_DN18725_c1_g3_i5	RL321_ARATH	-1.04	4.79	2.1E-06	1.5E-04	#N/A	#N/A	#N/A	#N/A	#N/A	#N/A	#N/A	#N/A
TRINITY_DN18772_c2_g1_i17	THF1_ORYSJ	-1.04	4.31	7.3E-04	2.4E-02	#N/A	#N/A	#N/A	#N/A	#N/A	#N/A	#N/A	#N/A

DET	Subject id	c9TR vs c7NT				c7TR vs c7NT				c7TR vs c9TR			
		Log_2	Log_2CPM	PValue	19FDR	Log_2	Log_2CPM	PValue	19FDR	Log_2	Log_2CPM	PValue	19FDR
TRINITY_DN21662_c2_g3_i2	CB2G_SOLLC	-1.04	9.34	1.4E-05	8.2E-04	#N/A	#N/A	#N/A	#N/A	#N/A	#N/A	#N/A	#N/A
TRINITY_DN12832_c0_g1_i2	SG1_ARATH	-1.03	4.93	1.6E-06	1.2E-04	#N/A	#N/A	#N/A	#N/A	#N/A	#N/A	#N/A	#N/A
TRINITY_DN20303_c0_g3_i2	U73C1_ARATH	-1.03	5.50	6.4E-04	2.2E-02	#N/A	#N/A	#N/A	#N/A	#N/A	#N/A	#N/A	#N/A
TRINITY_DN18612_c0_g1_i4	PNSB2_ARATH	-1.03	5.22	6.6E-04	2.2E-02	#N/A	#N/A	#N/A	#N/A	#N/A	#N/A	#N/A	#N/A
TRINITY_DN15360_c0_g1_i6	GPPL1_ARATH	-1.03	5.03	1.1E-03	3.3E-02	#N/A	#N/A	#N/A	#N/A	#N/A	#N/A	#N/A	#N/A
TRINITY_DN18496_c0_g1_i11	HEM2_ORYSJ	-1.02	7.42	8.9E-04	2.8E-02	#N/A	#N/A	#N/A	#N/A	#N/A	#N/A	#N/A	#N/A
TRINITY_DN22725_c1_g1_i1	EF1A_ORYSJ	-1.02	4.71	1.2E-04	5.3E-03	#N/A	#N/A	#N/A	#N/A	#N/A	#N/A	#N/A	#N/A
TRINITY_DN22127_c2_g1_i3	RL12_PRUAR	-1.02	4.96	2.1E-05	1.2E-03	#N/A	#N/A	#N/A	#N/A	#N/A	#N/A	#N/A	#N/A
TRINITY_DN18226_c0_g1_i12	GPT2_ARATH	-1.02	6.33	1.3E-06	9.5E-05	#N/A	#N/A	#N/A	#N/A	#N/A	#N/A	#N/A	#N/A
TRINITY_DN16247_c0_g1_i1	PLAS_ORYSJ	-1.02	10.15	1.8E-03	4.9E-02	#N/A	#N/A	#N/A	#N/A	#N/A	#N/A	#N/A	#N/A
TRINITY_DN20992_c0_g8_i1	CPP1_ARATH	-1.01	4.64	3.2E-05	1.7E-03	#N/A	#N/A	#N/A	#N/A	#N/A	#N/A	#N/A	#N/A
TRINITY_DN20834_c0_g1_i10	CPNB2_ARATH	-1.01	7.56	6.7E-06	4.2E-04	#N/A	#N/A	#N/A	#N/A	#N/A	#N/A	#N/A	#N/A
TRINITY_DN21859_c0_g2_i6	NSI_ORYSJ	-1.01	4.92	1.9E-05	1.1E-03	#N/A	#N/A	#N/A	#N/A	#N/A	#N/A	#N/A	#N/A
TRINITY_DN16108_c0_g1_i1	PNSB1_ARATH	-1.01	7.24	2.9E-05	1.6E-03	#N/A	#N/A	#N/A	#N/A	#N/A	#N/A	#N/A	#N/A
TRINITY_DN19273_c1_g2_i1	IF2B_WHEAT	-1.01	4.84	2.7E-06	1.8E-04	#N/A	#N/A	#N/A	#N/A	#N/A	#N/A	#N/A	#N/A
TRINITY_DN20965_c1_g3_i5	RK5_ORYSJ	-1.00	8.08	8.9E-04	2.8E-02	#N/A	#N/A	#N/A	#N/A	#N/A	#N/A	#N/A	#N/A
TRINITY_DN18594_c0_g1_i3	UTP11_ORYSJ	-1.00	3.21	3.9E-04	1.5E-02	#N/A	#N/A	#N/A	#N/A	#N/A	#N/A	#N/A	#N/A
TRINITY_DN20684_c1_g1_i14	PPOC_ORYSJ	-1.00	5.76	3.6E-05	1.9E-03	#N/A	#N/A	#N/A	#N/A	#N/A	#N/A	#N/A	#N/A
TRINITY_DN15488_c0_g1_i3	STR4A_ARATH	-1.00	5.66	4.2E-06	2.8E-04	#N/A	#N/A	#N/A	#N/A	#N/A	#N/A	#N/A	#N/A
TRINITY_DN18569_c0_g3_i4	RSMH_PARUW	-1.00	3.46	6.7E-04	2.2E-02	#N/A	#N/A	#N/A	#N/A	#N/A	#N/A	#N/A	#N/A
TRINITY_DN18065_c0_g3_i5	HEM4_ORYSJ	-1.00	3.22	1.0E-03	3.1E-02	#N/A	#N/A	#N/A	#N/A	#N/A	#N/A	#N/A	#N/A
TRINITY_DN20462_c0_g11_i2	RS92_ARATH	-1.00	5.25	3.8E-05	2.0E-03	#N/A	#N/A	#N/A	#N/A	#N/A	#N/A	#N/A	#N/A
TRINITY_DN19212_c1_g2_i10	YXEH_BACSU	-1.00	3.89	4.9E-04	1.7E-02	#N/A	#N/A	#N/A	#N/A	#N/A	#N/A	#N/A	#N/A
TRINITY_DN21056_c0_g2_i7	BAK1_ARATH	-0.99	4.82	7.1E-05	3.4E-03	#N/A	#N/A	#N/A	#N/A	#N/A	#N/A	#N/A	#N/A
TRINITY_DN21625_c0_g3_i1	CBP23_HORVU	-0.99	4.06	1.8E-04	7.8E-03	#N/A	#N/A	#N/A	#N/A	#N/A	#N/A	#N/A	#N/A
TRINITY_DN15553_c0_g3_i4	ROC4_NICSY	-0.99	7.63	2.5E-06	1.7E-04	#N/A	#N/A	#N/A	#N/A	#N/A	#N/A	#N/A	#N/A
TRINITY_DN18524_c0_g1_i4	ISPG_ORYSJ	-0.99	6.27	1.2E-04	5.2E-03	#N/A	#N/A	#N/A	#N/A	#N/A	#N/A	#N/A	#N/A
TRINITY_DN17019_c1_g1_i10	CHLP_ORYSJ	-0.99	9.81	7.9E-04	2.6E-02	#N/A	#N/A	#N/A	#N/A	#N/A	#N/A	#N/A	#N/A
TRINITY_DN15342_c0_g1_i6	QCR7_SOLTU	-0.99	4.59	2.4E-05	1.3E-03	#N/A	#N/A	#N/A	#N/A	#N/A	#N/A	#N/A	#N/A
TRINITY_DN22357_c1_g1_i6	CUT1A_ARATH	-0.99	9.31	4.1E-04	1.5E-02	#N/A	#N/A	#N/A	#N/A	#N/A	#N/A	#N/A	#N/A
TRINITY_DN15254_c2_g3_i2	CRR7_ARATH	-0.99	5.96	1.7E-03	4.6E-02	#N/A	#N/A	#N/A	#N/A	#N/A	#N/A	#N/A	#N/A
TRINITY_DN17165_c0_g1_i28	EBFC2_ARATH	-0.99	5.96	7.2E-04	2.4E-02	#N/A	#N/A	#N/A	#N/A	#N/A	#N/A	#N/A	#N/A
TRINITY_DN17522_c0_g2_i4	PSAG_SPIOL	-0.98	5.82	2.5E-05	1.4E-03	#N/A	#N/A	#N/A	#N/A	#N/A	#N/A	#N/A	#N/A
TRINITY_DN16930_c0_g1_i7	ISPD_ORYSJ	-0.98	4.82	3.0E-04	1.2E-02	#N/A	#N/A	#N/A	#N/A	#N/A	#N/A	#N/A	#N/A
TRINITY_DN21203_c1_g8_i2	SOT8_ARATH	-0.98	3.59	1.9E-03	5.1E-02	#N/A	#N/A	#N/A	#N/A	#N/A	#N/A	#N/A	#N/A
TRINITY_DN20823_c0_g1_i3	ROC1_NICSY	-0.98	5.46	4.3E-04	1.6E-02	#N/A	#N/A	#N/A	#N/A	#N/A	#N/A	#N/A	#N/A
TRINITY_DN18710_c0_g1_i5	CALR3_ARATH	-0.98	7.49	1.6E-04	6.8E-03	#N/A	#N/A	#N/A	#N/A	#N/A	#N/A	#N/A	#N/A
TRINITY_DN14595_c0_g1_i2	ACFR2_MAIZE	-0.98	4.57	4.5E-04	1.6E-02	#N/A	#N/A	#N/A	#N/A	#N/A	#N/A	#N/A	#N/A
TRINITY_DN14977_c0_g1_i2	AB6I_ARATH	-0.98	6.65	2.9E-04	1.1E-02	#N/A	#N/A	#N/A	#N/A	#N/A	#N/A	#N/A	#N/A
TRINITY_DN16326_c1_g3_i4	PPL1_ARATH	-0.98	7.38	8.3E-04	2.7E-02	#N/A	#N/A	#N/A	#N/A	#N/A	#N/A	#N/A	#N/A
TRINITY_DN19912_c0_g3_i8	RK3_TOBAC	-0.97	7.76	9.2E-04	2.9E-02	#N/A	#N/A	#N/A	#N/A	#N/A	#N/A	#N/A	#N/A
TRINITY_DN22026_c0_g2_i1	FAD3C_SOYBN	-0.97	4.54	4.8E-04	1.7E-02	#N/A	#N/A	#N/A	#N/A	#N/A	#N/A	#N/A	#N/A
TRINITY_DN20686_c1_g6_i2	RAF1_MAIZE	-0.97	3.46	2.0E-03	5.2E-02	#N/A	#N/A	#N/A	#N/A	#N/A	#N/A	#N/A	#N/A
TRINITY_DN18772_c2_g1_i4	THF1_ORYSJ	-0.97	7.05	5.5E-05	2.8E-03	#N/A	#N/A	#N/A	#N/A	#N/A	#N/A	#N/A	#N/A
TRINITY_DN15903_c0_g1_i6	ACCO_MUSAC	-0.97	3.20	1.4E-03	3.9E-02	#N/A	#N/A	#N/A	#N/A	#N/A	#N/A	#N/A	#N/A
TRINITY_DN20900_c1_g1_i1	R10A1_ARATH	-0.97	4.74	1.2E-04	5.5E-03	#N/A	#N/A	#N/A	#N/A	#N/A	#N/A	#N/A	#N/A
TRINITY_DN20280_c2_g1_i14	YQXC_BACSU	-0.96	4.19	5.9E-05	2.9E-03	#N/A	#N/A	#N/A	#N/A	#N/A	#N/A	#N/A	#N/A
TRINITY_DN14739_c0_g2_i1	RH39_ORYSJ	-0.96	6.38	3.1E-06	2.1E-04	#N/A	#N/A	#N/A	#N/A	#N/A	#N/A	#N/A	#N/A
TRINITY_DN15553_c0_g6_i1	ROC3_NICSY	-0.96	5.94	7.1E-05	3.4E-03	#N/A	#N/A	#N/A	#N/A	#N/A	#N/A	#N/A	#N/A
TRINITY_DN21143_c0_g2_i3	DAPB3_ARATH	-0.96	5.55	5.6E-05	2.8E-03	#N/A	#N/A	#N/A	#N/A	#N/A	#N/A	#N/A	#N/A
TRINITY_DN18172_c0_g1_i21	GUAD_BACSU	-0.96	3.86	6.0E-04	2.1E-02	#N/A	#N/A	#N/A	#N/A	#N/A	#N/A	#N/A	#N/A
TRINITY_DN18941_c2_g4_i6	NDK4_ARATH	-0.96	7.35	5.8E-06	3.7E-04	#N/A	#N/A	#N/A	#N/A	#N/A	#N/A	#N/A	#N/A
TRINITY_DN14829_c3_g4_i4	RK4_TOBAC	-0.96	8.26	1.1E-03	3.2E-02	#N/A	#N/A	#N/A	#N/A	#N/A	#N/A	#N/A	#N/A
TRINITY_DN19623_c3_g1_i2	DIV_ANTMA	-0.95	4.43	3.2E-04	1.2E-02	#N/A	#N/A	#N/A	#N/A	#N/A	#N/A	#N/A	#N/A
TRINITY_DN22765_c0_g1_i1	PNSB1_ARATH	-0.95	5.07	9.6E-04	3.0E-02	#N/A	#N/A	#N/A	#N/A	#N/A	#N/A	#N/A	#N/A
TRINITY_DN21258_c0_g2_i3	SODF1_ORYSJ	-0.95	5.44	5.4E-05	2.7E-03	#N/A	#N/A	#N/A	#N/A	#N/A	#N/A	#N/A	#N/A
TRINITY_DN19843_c1_g3_i2	DCVR_MAIZE	-0.95	4.13	8.1E-04	2.6E-02	#N/A	#N/A	#N/A	#N/A	#N/A	#N/A	#N/A	#N/A
TRINITY_DN19040_c1_g1_i13	GRPE_THEEB	-0.95	5.48	7.5E-04	2.5E-02	#N/A	#N/A	#N/A	#N/A	#N/A	#N/A	#N/A	#N/A
TRINITY_DN14418_c0_g1_i4	GGPPS_HEVBR	-0.95	4.96	5.5E-04	1.9E-02	#N/A	#N/A	#N/A	#N/A	#N/A	#N/A	#N/A	#N/A
TRINITY_DN17101_c1_g1_i1	ROC2_NICPL	-0.95	4.59	1.2E-03	3.6E-02	#N/A	#N/A	#N/A	#N/A	#N/A	#N/A	#N/A	#N/A
TRINITY_DN14405_c0_g3_i1	PSBO_TOBAC	-0.95	8.67	2.3E-04	9.3E-03	#N/A	#N/A	#N/A	#N/A	#N/A	#N/A	#N/A	#N/A

DET	Subject id	c9TR vs c7NT				c7TR vs c7NT				c7TR vs c9TR			
		Log_2	Log_2CPM	PValue	19FDR	Log_2	Log_2CPM	PValue	19FDR	Log_2	Log_2CPM	PValue	19FDR
TRINITY_DN22315_c0_g1_i16	HEM1_ORYSJ	-0.95	9.02	7.5E-05	3.6E-03	#N/A	#N/A	#N/A	#N/A	#N/A	#N/A	#N/A	#N/A
TRINITY_DN14557_c0_g2_i3	ACT1_ORYSJ	-0.94	4.73	5.0E-05	2.5E-03	#N/A	#N/A	#N/A	#N/A	#N/A	#N/A	#N/A	#N/A
TRINITY_DN18294_c1_g3_i8	TIC21_ARATH	-0.94	7.70	6.1E-04	2.1E-02	#N/A	#N/A	#N/A	#N/A	#N/A	#N/A	#N/A	#N/A
TRINITY_DN15176_c0_g3_i5	GLPT1_ARATH	-0.94	3.18	2.1E-03	5.4E-02	#N/A	#N/A	#N/A	#N/A	#N/A	#N/A	#N/A	#N/A
TRINITY_DN19045_c0_g1_i11	CAS_ORYSJ	-0.94	5.90	2.9E-05	1.6E-03	#N/A	#N/A	#N/A	#N/A	#N/A	#N/A	#N/A	#N/A
TRINITY_DN21059_c0_g1_i4	C3H41_ORYSJ	-0.94	3.10	1.4E-03	4.1E-02	#N/A	#N/A	#N/A	#N/A	#N/A	#N/A	#N/A	#N/A
TRINITY_DN20830_c0_g2_i3	Y3589_ARATH	-0.93	3.15	1.4E-03	4.0E-02	#N/A	#N/A	#N/A	#N/A	#N/A	#N/A	#N/A	#N/A
TRINITY_DN22427_c1_g2_i7	PNSB3_ARATH	-0.93	7.68	1.7E-03	4.7E-02	#N/A	#N/A	#N/A	#N/A	#N/A	#N/A	#N/A	#N/A
TRINITY_DN22315_c0_g1_i11	HEM1_ORYSJ	-0.93	4.80	3.7E-04	1.4E-02	#N/A	#N/A	#N/A	#N/A	#N/A	#N/A	#N/A	#N/A
TRINITY_DN16607_c0_g4_i1	LGUC_ARATH	-0.93	6.39	6.9E-04	2.3E-02	#N/A	#N/A	#N/A	#N/A	#N/A	#N/A	#N/A	#N/A
TRINITY_DN20923_c0_g2_i1	RR20_ARATH	-0.93	8.54	4.0E-04	1.5E-02	#N/A	#N/A	#N/A	#N/A	#N/A	#N/A	#N/A	#N/A
TRINITY_DN12886_c0_g1_i2	PORB_ORYSJ	-0.93	4.39	3.4E-04	1.3E-02	#N/A	#N/A	#N/A	#N/A	#N/A	#N/A	#N/A	#N/A
TRINITY_DN18654_c3_g3_i2	ANX4_FRAAN	-0.93	4.73	1.2E-03	3.6E-02	#N/A	#N/A	#N/A	#N/A	#N/A	#N/A	#N/A	#N/A
TRINITY_DN16631_c0_g6_i2	UBC28_ARATH	-0.93	5.35	5.2E-05	2.6E-03	#N/A	#N/A	#N/A	#N/A	#N/A	#N/A	#N/A	#N/A
TRINITY_DN13589_c0_g1_i3	PSAH_MAIZE	-0.93	10.77	1.7E-04	7.5E-03	#N/A	#N/A	#N/A	#N/A	#N/A	#N/A	#N/A	#N/A
TRINITY_DN14578_c0_g1_i8	RL9_ORYSJ	-0.92	6.21	2.2E-05	1.2E-03	#N/A	#N/A	#N/A	#N/A	#N/A	#N/A	#N/A	#N/A
TRINITY_DN13788_c0_g1_i3	ZB14_MAIZE	-0.92	4.99	8.6E-04	2.8E-02	#N/A	#N/A	#N/A	#N/A	#N/A	#N/A	#N/A	#N/A
TRINITY_DN15432_c2_g6_i3	CH10C_ARATH	-0.92	7.20	1.1E-04	5.2E-03	#N/A	#N/A	#N/A	#N/A	#N/A	#N/A	#N/A	#N/A
TRINITY_DN17199_c1_g2_i7	KASC2_ARATH	-0.92	4.79	6.1E-05	3.0E-03	#N/A	#N/A	#N/A	#N/A	#N/A	#N/A	#N/A	#N/A
TRINITY_DN21055_c0_g2_i5	GLNAC_MAIZE	-0.92	8.26	3.5E-04	1.3E-02	#N/A	#N/A	#N/A	#N/A	#N/A	#N/A	#N/A	#N/A
TRINITY_DN16043_c2_g1_i2	CCS_ORYSJ	-0.92	4.70	4.5E-05	2.3E-03	#N/A	#N/A	#N/A	#N/A	#N/A	#N/A	#N/A	#N/A
TRINITY_DN18522_c2_g2_i6	GSO1_ARATH	-0.92	3.90	1.6E-03	4.4E-02	#N/A	#N/A	#N/A	#N/A	#N/A	#N/A	#N/A	#N/A
TRINITY_DN16626_c0_g3_i1	MAD50_ORYSJ	-0.92	4.62	1.8E-04	7.8E-03	#N/A	#N/A	#N/A	#N/A	#N/A	#N/A	#N/A	#N/A
TRINITY_DN21825_c0_g1_i3	SODF2_ORYSJ	-0.91	4.82	6.3E-05	3.1E-03	#N/A	#N/A	#N/A	#N/A	#N/A	#N/A	#N/A	#N/A
TRINITY_DN10648_c0_g1_i1	TI223_ARATH	-0.91	3.92	3.5E-04	1.3E-02	#N/A	#N/A	#N/A	#N/A	#N/A	#N/A	#N/A	#N/A
TRINITY_DN22315_c0_g1_i3	HEM1_ORYSJ	-0.91	5.55	8.6E-05	4.1E-03	#N/A	#N/A	#N/A	#N/A	#N/A	#N/A	#N/A	#N/A
TRINITY_DN21651_c2_g4_i6	ILVH2_ARATH	-0.91	5.91	2.1E-05	1.2E-03	#N/A	#N/A	#N/A	#N/A	#N/A	#N/A	#N/A	#N/A
TRINITY_DN20256_c1_g1_i14	RRFC_ORYSJ	-0.91	5.57	1.3E-03	3.8E-02	#N/A	#N/A	#N/A	#N/A	#N/A	#N/A	#N/A	#N/A
TRINITY_DN14837_c3_g9_i2	SCO2_ARATH	-0.91	4.42	2.8E-04	1.1E-02	#N/A	#N/A	#N/A	#N/A	#N/A	#N/A	#N/A	#N/A
TRINITY_DN16855_c0_g7_i1	GRS10_ORYSJ	-0.90	4.53	1.3E-04	5.8E-03	#N/A	#N/A	#N/A	#N/A	#N/A	#N/A	#N/A	#N/A
TRINITY_DN18200_c0_g4_i1	CB2D_SOLLC	-0.90	9.61	1.2E-04	5.4E-03	#N/A	#N/A	#N/A	#N/A	#N/A	#N/A	#N/A	#N/A
TRINITY_DN15833_c2_g6_i1	Y1609_ARATH	-0.90	3.98	7.6E-04	2.5E-02	#N/A	#N/A	#N/A	#N/A	#N/A	#N/A	#N/A	#N/A
TRINITY_DN15773_c2_g1_i1	CB29_MAIZE	-0.90	11.26	4.6E-04	1.7E-02	#N/A	#N/A	#N/A	#N/A	#N/A	#N/A	#N/A	#N/A
TRINITY_DN15793_c0_g2_i9	XYLL3_ARATH	-0.90	4.90	8.4E-04	2.7E-02	#N/A	#N/A	#N/A	#N/A	#N/A	#N/A	#N/A	#N/A
TRINITY_DN18292_c2_g4_i3	FABI1_ORYSJ	-0.90	3.97	1.2E-03	3.5E-02	#N/A	#N/A	#N/A	#N/A	#N/A	#N/A	#N/A	#N/A
TRINITY_DN18245_c2_g3_i5	RISB_ARATH	-0.89	4.21	7.8E-04	2.6E-02	#N/A	#N/A	#N/A	#N/A	#N/A	#N/A	#N/A	#N/A
TRINITY_DN17522_c0_g2_i1	PSAG_HORVU	-0.89	8.36	1.0E-03	3.2E-02	#N/A	#N/A	#N/A	#N/A	#N/A	#N/A	#N/A	#N/A
TRINITY_DN21806_c2_g3_i8	RK28_ARATH	-0.89	6.41	1.5E-03	4.3E-02	#N/A	#N/A	#N/A	#N/A	#N/A	#N/A	#N/A	#N/A
TRINITY_DN15302_c0_g1_i1	INT4_ARATH	-0.89	4.52	8.6E-04	2.7E-02	#N/A	#N/A	#N/A	#N/A	#N/A	#N/A	#N/A	#N/A
TRINITY_DN19245_c0_g2_i1	RR13_SPIOL	-0.89	8.15	2.8E-04	1.1E-02	#N/A	#N/A	#N/A	#N/A	#N/A	#N/A	#N/A	#N/A
TRINITY_DN18655_c1_g5_i1	RR5_ARATH	-0.89	6.87	1.7E-03	4.7E-02	#N/A	#N/A	#N/A	#N/A	#N/A	#N/A	#N/A	#N/A
TRINITY_DN19679_c1_g1_i18	RH58_ORYSJ	-0.89	4.16	1.3E-03	3.9E-02	#N/A	#N/A	#N/A	#N/A	#N/A	#N/A	#N/A	#N/A
TRINITY_DN15860_c0_g1_i2	STR11_ARATH	-0.89	5.98	9.8E-05	4.5E-03	#N/A	#N/A	#N/A	#N/A	#N/A	#N/A	#N/A	#N/A
TRINITY_DN16305_c0_g1_i8	YLMG2_ARATH	-0.89	5.15	3.4E-04	1.3E-02	#N/A	#N/A	#N/A	#N/A	#N/A	#N/A	#N/A	#N/A
TRINITY_DN19932_c0_g1_i12	AGAL_ORYSJ	-0.88	4.20	2.2E-04	9.2E-03	#N/A	#N/A	#N/A	#N/A	#N/A	#N/A	#N/A	#N/A
TRINITY_DN21114_c0_g2_i14	HEM6_ORYSJ	-0.88	6.40	9.3E-04	2.9E-02	#N/A	#N/A	#N/A	#N/A	#N/A	#N/A	#N/A	#N/A
TRINITY_DN21716_c0_g1_i5	SCMC1_DANRE	-0.88	4.09	1.1E-03	3.4E-02	#N/A	#N/A	#N/A	#N/A	#N/A	#N/A	#N/A	#N/A
TRINITY_DN17505_c0_g1_i14	DTX45_ARATH	-0.88	3.88	3.7E-04	1.4E-02	#N/A	#N/A	#N/A	#N/A	#N/A	#N/A	#N/A	#N/A
TRINITY_DN16305_c0_g1_i6	YLMG2_ARATH	-0.88	5.24	8.3E-05	3.9E-03	#N/A	#N/A	#N/A	#N/A	#N/A	#N/A	#N/A	#N/A
TRINITY_DN20319_c1_g2_i1	GLYR2_ARATH	-0.88	5.93	2.1E-05	1.2E-03	#N/A	#N/A	#N/A	#N/A	#N/A	#N/A	#N/A	#N/A
TRINITY_DN19513_c0_g3_i1	RL354_ARATH	-0.88	5.22	1.1E-04	4.9E-03	#N/A	#N/A	#N/A	#N/A	#N/A	#N/A	#N/A	#N/A
TRINITY_DN17505_c0_g1_i11	DTX45_ARATH	-0.88	4.29	2.0E-03	5.2E-02	#N/A	#N/A	#N/A	#N/A	#N/A	#N/A	#N/A	#N/A
TRINITY_DN17165_c0_g1_i1	EBFC2_ARATH	-0.88	5.94	1.1E-03	3.4E-02	#N/A	#N/A	#N/A	#N/A	#N/A	#N/A	#N/A	#N/A
TRINITY_DN14428_c0_g1_i2	HS905_ARATH	-0.88	5.08	7.6E-05	3.6E-03	#N/A	#N/A	#N/A	#N/A	#N/A	#N/A	#N/A	#N/A
TRINITY_DN18048_c0_g1_i10	AK1H_ECOLI	-0.88	4.62	5.2E-04	1.8E-02	#N/A	#N/A	#N/A	#N/A	#N/A	#N/A	#N/A	#N/A
TRINITY_DN19432_c1_g1_i2	HERC4_MOUSE	-0.87	4.13	2.6E-04	1.0E-02	#N/A	#N/A	#N/A	#N/A	#N/A	#N/A	#N/A	#N/A
TRINITY_DN19477_c1_g1_i2	CB4A_SOLLC	-0.87	7.08	3.4E-05	1.8E-03	#N/A	#N/A	#N/A	#N/A	#N/A	#N/A	#N/A	#N/A
TRINITY_DN21100_c0_g2_i1	PSAL_HORVU	-0.87	6.95	5.0E-04	1.8E-02	#N/A	#N/A	#N/A	#N/A	#N/A	#N/A	#N/A	#N/A
TRINITY_DN21521_c0_g4_i4	RL30_MAIZE	-0.87	5.43	3.4E-04	1.3E-02	#N/A	#N/A	#N/A	#N/A	#N/A	#N/A	#N/A	#N/A
TRINITY_DN15483_c0_g3_i1	MGDG1_ORYSJ	-0.87	5.32	9.9E-04	3.1E-02	#N/A	#N/A	#N/A	#N/A	#N/A	#N/A	#N/A	#N/A
TRINITY_DN16974_c0_g5_i1	RS20_ORYSJ	-0.87	4.54	2.0E-04	8.3E-03	#N/A	#N/A	#N/A	#N/A	#N/A	#N/A	#N/A	#N/A

DET	Subject id	c9TR vs c7NT				c7TR vs c7NT				c7TR vs c9TR			
		Log_2	Log_2CPM	PValue	19FDR	Log_2	Log_2CPM	PValue	19FDR	Log_2	Log_2CPM	PValue	19FDR
TRINITY_DN21100_c0_g2_i5	PSAL_HORVU	-0.87	7.49	4.7E-04	1.7E-02	#N/A	#N/A	#N/A	#N/A	#N/A	#N/A	#N/A	#N/A
TRINITY_DN16340_c3_g1_i3	RS51_ARATH	-0.86	4.32	1.6E-04	7.1E-03	#N/A	#N/A	#N/A	#N/A	#N/A	#N/A	#N/A	#N/A
TRINITY_DN15627_c0_g2_i3	CH10C_ARATH	-0.86	4.58	5.9E-04	2.0E-02	#N/A	#N/A	#N/A	#N/A	#N/A	#N/A	#N/A	#N/A
TRINITY_DN19657_c2_g4_i18	ARP8_ORYSJ	-0.86	3.70	1.1E-03	3.4E-02	#N/A	#N/A	#N/A	#N/A	#N/A	#N/A	#N/A	#N/A
TRINITY_DN15358_c0_g2_i4	RL142_ARATH	-0.86	5.11	1.4E-04	6.1E-03	#N/A	#N/A	#N/A	#N/A	#N/A	#N/A	#N/A	#N/A
TRINITY_DN16461_c0_g1_i8	TATA_MAIZE	-0.86	4.04	2.7E-04	1.1E-02	#N/A	#N/A	#N/A	#N/A	#N/A	#N/A	#N/A	#N/A
TRINITY_DN19932_c0_g1_i4	AGAL_ORYSJ	-0.85	5.73	5.6E-05	2.8E-03	#N/A	#N/A	#N/A	#N/A	#N/A	#N/A	#N/A	#N/A
TRINITY_DN21960_c2_g3_i1	RL273_ARATH	-0.85	4.22	1.3E-03	3.8E-02	#N/A	#N/A	#N/A	#N/A	#N/A	#N/A	#N/A	#N/A
TRINITY_DN14809_c1_g1_i18	CEST_ORYSI	-0.85	6.96	7.8E-04	2.5E-02	#N/A	#N/A	#N/A	#N/A	#N/A	#N/A	#N/A	#N/A
TRINITY_DN20524_c0_g5_i1	CB22_CUCSA	-0.85	9.43	8.5E-05	4.0E-03	#N/A	#N/A	#N/A	#N/A	#N/A	#N/A	#N/A	#N/A
TRINITY_DN18484_c5_g4_i5	PTA16_ARATH	-0.85	9.46	1.1E-03	3.4E-02	#N/A	#N/A	#N/A	#N/A	#N/A	#N/A	#N/A	#N/A
TRINITY_DN18555_c1_g1_i9	UPP_TOBAC	-0.85	5.04	1.7E-04	7.4E-03	#N/A	#N/A	#N/A	#N/A	#N/A	#N/A	#N/A	#N/A
TRINITY_DN16705_c0_g1_i4	CB5_ARATH	-0.85	11.29	1.3E-03	3.8E-02	#N/A	#N/A	#N/A	#N/A	#N/A	#N/A	#N/A	#N/A
TRINITY_DN19392_c1_g2_i15	RL23_ARATH	-0.85	4.87	2.3E-04	9.4E-03	#N/A	#N/A	#N/A	#N/A	#N/A	#N/A	#N/A	#N/A
TRINITY_DN15773_c2_g1_i2	CB21_MAIZE	-0.84	8.80	1.2E-04	5.6E-03	#N/A	#N/A	#N/A	#N/A	#N/A	#N/A	#N/A	#N/A
TRINITY_DN15143_c0_g4_i2	HDDC2_DANRE	-0.84	5.49	1.2E-03	3.6E-02	#N/A	#N/A	#N/A	#N/A	#N/A	#N/A	#N/A	#N/A
TRINITY_DN18429_c0_g1_i28	TGD2_ARATH	-0.84	4.07	1.5E-03	4.2E-02	#N/A	#N/A	#N/A	#N/A	#N/A	#N/A	#N/A	#N/A
TRINITY_DN19865_c0_g1_i5	HSP7S_PEA	-0.84	7.73	8.0E-04	2.6E-02	#N/A	#N/A	#N/A	#N/A	#N/A	#N/A	#N/A	#N/A
TRINITY_DN14728_c0_g7_i3	H2A1_ORYSJ	-0.84	4.76	1.9E-03	5.0E-02	#N/A	#N/A	#N/A	#N/A	#N/A	#N/A	#N/A	#N/A
TRINITY_DN18370_c1_g1_i3	PSAN_MAIZE	-0.83	9.90	5.1E-04	1.8E-02	#N/A	#N/A	#N/A	#N/A	#N/A	#N/A	#N/A	#N/A
TRINITY_DN17737_c0_g1_i2	CB24_PEA	-0.83	9.42	6.6E-05	3.2E-03	#N/A	#N/A	#N/A	#N/A	#N/A	#N/A	#N/A	#N/A
TRINITY_DN19247_c0_g4_i1	TIM9_ORYSJ	-0.83	3.36	1.8E-03	4.8E-02	#N/A	#N/A	#N/A	#N/A	#N/A	#N/A	#N/A	#N/A
TRINITY_DN14512_c0_g5_i1	RL37A_ORYSJ	-0.83	3.73	1.4E-03	3.9E-02	#N/A	#N/A	#N/A	#N/A	#N/A	#N/A	#N/A	#N/A
TRINITY_DN19077_c0_g4_i2	SIGC_ARATH	-0.82	4.45	1.4E-03	3.9E-02	#N/A	#N/A	#N/A	#N/A	#N/A	#N/A	#N/A	#N/A
TRINITY_DN18701_c0_g1_i1	RK12_ORYSJ	-0.82	5.59	3.5E-04	1.3E-02	#N/A	#N/A	#N/A	#N/A	#N/A	#N/A	#N/A	#N/A
TRINITY_DN20474_c2_g5_i6	FLU_ARATH	-0.82	6.26	1.1E-04	5.2E-03	#N/A	#N/A	#N/A	#N/A	#N/A	#N/A	#N/A	#N/A
TRINITY_DN19477_c1_g1_i1	CB4A_SOLLC	-0.82	6.92	2.9E-04	1.1E-02	#N/A	#N/A	#N/A	#N/A	#N/A	#N/A	#N/A	#N/A
TRINITY_DN18772_c2_g1_i7	THF1_ORYSJ	-0.82	6.82	2.7E-04	1.1E-02	#N/A	#N/A	#N/A	#N/A	#N/A	#N/A	#N/A	#N/A
TRINITY_DN20604_c0_g6_i8	MORF9_ARATH	-0.82	5.72	1.8E-04	7.8E-03	#N/A	#N/A	#N/A	#N/A	#N/A	#N/A	#N/A	#N/A
TRINITY_DN15773_c2_g4_i1	CB2D_SOLLC	-0.81	6.01	1.2E-03	3.5E-02	#N/A	#N/A	#N/A	#N/A	#N/A	#N/A	#N/A	#N/A
TRINITY_DN18705_c0_g1_i39	BGL05_ORYSJ	-0.81	5.24	1.5E-04	6.7E-03	#N/A	#N/A	#N/A	#N/A	#N/A	#N/A	#N/A	#N/A
TRINITY_DN12766_c0_g1_i2	CPP1_ARATH	-0.81	4.15	1.3E-03	3.8E-02	#N/A	#N/A	#N/A	#N/A	#N/A	#N/A	#N/A	#N/A
TRINITY_DN17199_c1_g2_i4	KASC2_ARATH	-0.81	6.49	3.6E-04	1.4E-02	#N/A	#N/A	#N/A	#N/A	#N/A	#N/A	#N/A	#N/A
TRINITY_DN18325_c0_g3_i4	YNBB_BACSU	-0.81	4.24	5.5E-04	1.9E-02	#N/A	#N/A	#N/A	#N/A	#N/A	#N/A	#N/A	#N/A
TRINITY_DN18966_c1_g2_i13	PSAE_HORVU	-0.81	7.50	1.0E-03	3.2E-02	#N/A	#N/A	#N/A	#N/A	#N/A	#N/A	#N/A	#N/A
TRINITY_DN19094_c0_g1_i11	GH35_ORYSJ	-0.81	5.60	1.1E-03	3.4E-02	#N/A	#N/A	#N/A	#N/A	#N/A	#N/A	#N/A	#N/A
TRINITY_DN22487_c1_g2_i1	TI201_ARATH	-0.80	5.95	2.7E-04	1.1E-02	#N/A	#N/A	#N/A	#N/A	#N/A	#N/A	#N/A	#N/A
TRINITY_DN22487_c1_g2_i3	TI201_ARATH	-0.80	4.57	5.5E-04	1.9E-02	#N/A	#N/A	#N/A	#N/A	#N/A	#N/A	#N/A	#N/A
TRINITY_DN19615_c0_g2_i15	DES2_SORBI	-0.80	8.24	9.3E-04	2.9E-02	#N/A	#N/A	#N/A	#N/A	#N/A	#N/A	#N/A	#N/A
TRINITY_DN19588_c0_g1_i10	FER5_MAIZE	-0.80	5.44	1.1E-03	3.2E-02	#N/A	#N/A	#N/A	#N/A	#N/A	#N/A	#N/A	#N/A
TRINITY_DN18537_c2_g2_i11	Y1815_ARATH	-0.80	4.60	1.4E-03	4.0E-02	#N/A	#N/A	#N/A	#N/A	#N/A	#N/A	#N/A	#N/A
TRINITY_DN18285_c0_g2_i20	MENA_ARATH	-0.80	7.09	1.4E-03	3.9E-02	#N/A	#N/A	#N/A	#N/A	#N/A	#N/A	#N/A	#N/A
TRINITY_DN21788_c1_g1_i1	PSAO_ARATH	-0.79	10.89	9.3E-04	2.9E-02	#N/A	#N/A	#N/A	#N/A	#N/A	#N/A	#N/A	#N/A
TRINITY_DN21041_c0_g2_i5	CB12_PETHY	-0.79	8.23	1.6E-04	6.9E-03	#N/A	#N/A	#N/A	#N/A	#N/A	#N/A	#N/A	#N/A
TRINITY_DN19965_c0_g4_i1	DXS1_ORYSJ	-0.79	7.70	1.5E-04	6.8E-03	#N/A	#N/A	#N/A	#N/A	#N/A	#N/A	#N/A	#N/A
TRINITY_DN19256_c0_g1_i7	RL18A_ORYSJ	-0.78	4.98	5.3E-04	1.9E-02	#N/A	#N/A	#N/A	#N/A	#N/A	#N/A	#N/A	#N/A
TRINITY_DN17228_c0_g1_i4	PROT2_ORYSJ	-0.78	5.59	2.1E-03	5.5E-02	#N/A	#N/A	#N/A	#N/A	#N/A	#N/A	#N/A	#N/A
TRINITY_DN19653_c0_g2_i1	GDT12_ORYSJ	-0.78	5.77	2.1E-04	8.7E-03	#N/A	#N/A	#N/A	#N/A	#N/A	#N/A	#N/A	#N/A
TRINITY_DN15358_c0_g2_i5	RL141_ARATH	-0.78	4.14	1.3E-03	3.7E-02	#N/A	#N/A	#N/A	#N/A	#N/A	#N/A	#N/A	#N/A
TRINITY_DN17819_c0_g3_i3	RK34_ARATH	-0.78	4.76	1.4E-03	4.0E-02	#N/A	#N/A	#N/A	#N/A	#N/A	#N/A	#N/A	#N/A
TRINITY_DN17950_c2_g2_i9	PSAD_CUCSA	-0.78	9.77	1.5E-03	4.3E-02	#N/A	#N/A	#N/A	#N/A	#N/A	#N/A	#N/A	#N/A
TRINITY_DN15553_c0_g1_i1	ROC3_NICSY	-0.77	8.08	1.2E-03	3.5E-02	#N/A	#N/A	#N/A	#N/A	#N/A	#N/A	#N/A	#N/A
TRINITY_DN18653_c0_g4_i3	APX2_ORYSJ	-0.77	4.80	3.7E-04	1.4E-02	#N/A	#N/A	#N/A	#N/A	#N/A	#N/A	#N/A	#N/A
TRINITY_DN18710_c0_g1_i16	CALR3_ARATH	-0.77	6.94	4.9E-04	1.8E-02	#N/A	#N/A	#N/A	#N/A	#N/A	#N/A	#N/A	#N/A
TRINITY_DN18727_c0_g2_i3	TIC32_ARATH	-0.77	7.60	1.0E-04	4.7E-03	#N/A	#N/A	#N/A	#N/A	#N/A	#N/A	#N/A	#N/A
TRINITY_DN16705_c0_g1_i5	CB5_ARATH	-0.77	9.53	6.4E-04	2.2E-02	#N/A	#N/A	#N/A	#N/A	#N/A	#N/A	#N/A	#N/A
TRINITY_DN17165_c0_g1_i35	EBFC2_ARATH	-0.77	5.27	6.1E-04	2.1E-02	#N/A	#N/A	#N/A	#N/A	#N/A	#N/A	#N/A	#N/A
TRINITY_DN15552_c0_g1_i1	G3PA_MAIZE	-0.77	9.21	2.1E-03	5.4E-02	#N/A	#N/A	#N/A	#N/A	#N/A	#N/A	#N/A	#N/A
TRINITY_DN14997_c0_g4_i3	RS23_FRAAN	-0.77	4.95	9.5E-04	3.0E-02	#N/A	#N/A	#N/A	#N/A	#N/A	#N/A	#N/A	#N/A
TRINITY_DN13226_c0_g1_i1	RS142_MAIZE	-0.76	4.34	5.6E-04	1.9E-02	#N/A	#N/A	#N/A	#N/A	#N/A	#N/A	#N/A	#N/A
TRINITY_DN14915_c0_g1_i4	RER4_ARATH	-0.76	6.82	1.2E-03	3.5E-02	#N/A	#N/A	#N/A	#N/A	#N/A	#N/A	#N/A	#N/A

DET	Subject id	c9TR vs c7NT				c7TR vs c7NT				c7TR vs c9TR			
		Log_2	Log_2CPM	PValue	19FDR	Log_2	Log_2CPM	PValue	19FDR	Log_2	Log_2CPM	PValue	19FDR
TRINITY_DN22378_c1_g6_i1	RS30_ARATH	-0.76	4.71	5.4E-04	1.9E-02	#N/A	#N/A	#N/A	#N/A	#N/A	#N/A	#N/A	#N/A
TRINITY_DN20717_c2_g1_i1	CX5B2_ARATH	-0.76	4.75	9.8E-04	3.0E-02	#N/A	#N/A	#N/A	#N/A	#N/A	#N/A	#N/A	#N/A
TRINITY_DN18976_c0_g2_i4	TIC62_PEA	-0.76	5.07	3.4E-04	1.3E-02	#N/A	#N/A	#N/A	#N/A	#N/A	#N/A	#N/A	#N/A
TRINITY_DN18466_c1_g3_i1	PHSL_IPOBA	-0.75	5.55	2.4E-04	9.7E-03	#N/A	#N/A	#N/A	#N/A	#N/A	#N/A	#N/A	#N/A
TRINITY_DN20287_c0_g5_i8	R35A3_ARATH	-0.75	4.54	1.3E-03	3.9E-02	#N/A	#N/A	#N/A	#N/A	#N/A	#N/A	#N/A	#N/A
TRINITY_DN14755_c0_g1_i5	RH50_ORYSJ	-0.74	5.62	1.0E-03	3.2E-02	#N/A	#N/A	#N/A	#N/A	#N/A	#N/A	#N/A	#N/A
TRINITY_DN21439_c1_g1_i25	SR34_ARATH	-0.74	4.35	1.6E-03	4.5E-02	#N/A	#N/A	#N/A	#N/A	#N/A	#N/A	#N/A	#N/A
TRINITY_DN15574_c4_g2_i13	PTA14_ARATH	-0.74	5.44	1.4E-03	4.1E-02	#N/A	#N/A	#N/A	#N/A	#N/A	#N/A	#N/A	#N/A
TRINITY_DN16108_c0_g1_i5	PNSB1_ARATH	-0.73	7.70	3.3E-04	1.3E-02	#N/A	#N/A	#N/A	#N/A	#N/A	#N/A	#N/A	#N/A
TRINITY_DN20800_c0_g3_i9	RS26_ORYSJ	-0.73	4.47	1.1E-03	3.3E-02	#N/A	#N/A	#N/A	#N/A	#N/A	#N/A	#N/A	#N/A
TRINITY_DN15627_c0_g2_i2	CH10C_ARATH	-0.73	7.22	3.1E-04	1.2E-02	#N/A	#N/A	#N/A	#N/A	#N/A	#N/A	#N/A	#N/A
TRINITY_DN15197_c1_g1_i1	OEP24_ORYSI	-0.73	5.46	1.5E-03	4.2E-02	#N/A	#N/A	#N/A	#N/A	#N/A	#N/A	#N/A	#N/A
TRINITY_DN20417_c1_g5_i1	QCR6_SOLTU	-0.73	4.67	1.4E-03	4.0E-02	#N/A	#N/A	#N/A	#N/A	#N/A	#N/A	#N/A	#N/A
TRINITY_DN16672_c0_g1_i14	EF1A_MAIZE	-0.72	5.34	1.7E-03	4.6E-02	#N/A	#N/A	#N/A	#N/A	#N/A	#N/A	#N/A	#N/A
TRINITY_DN21956_c1_g9_i1	R15A1_ARATH	-0.72	4.69	1.6E-03	4.4E-02	#N/A	#N/A	#N/A	#N/A	#N/A	#N/A	#N/A	#N/A
TRINITY_DN13342_c0_g1_i1	SYMM_ARATH	-0.72	5.63	6.3E-04	2.1E-02	#N/A	#N/A	#N/A	#N/A	#N/A	#N/A	#N/A	#N/A
TRINITY_DN17045_c0_g3_i2	RS7_SECCE	-0.71	5.54	1.4E-03	3.9E-02	#N/A	#N/A	#N/A	#N/A	#N/A	#N/A	#N/A	#N/A
TRINITY_DN19513_c0_g1_i3	RL354_ARATH	-0.71	4.96	6.7E-04	2.3E-02	#N/A	#N/A	#N/A	#N/A	#N/A	#N/A	#N/A	#N/A
TRINITY_DN19522_c0_g1_i6	RL151_ARATH	-0.71	4.81	1.6E-03	4.5E-02	#N/A	#N/A	#N/A	#N/A	#N/A	#N/A	#N/A	#N/A
TRINITY_DN20151_c0_g1_i6	RH3_ORYSJ	-0.70	8.07	1.4E-03	3.9E-02	#N/A	#N/A	#N/A	#N/A	#N/A	#N/A	#N/A	#N/A
TRINITY_DN17641_c0_g1_i8	KPYC1_ORYSJ	-0.70	7.19	2.1E-03	5.4E-02	#N/A	#N/A	#N/A	#N/A	#N/A	#N/A	#N/A	#N/A
TRINITY_DN16971_c0_g3_i3	OTP51_ORYSJ	-0.70	4.61	2.0E-03	5.2E-02	#N/A	#N/A	#N/A	#N/A	#N/A	#N/A	#N/A	#N/A
TRINITY_DN17935_c0_g1_i2	RL212_ARATH	-0.70	5.24	1.4E-03	4.1E-02	#N/A	#N/A	#N/A	#N/A	#N/A	#N/A	#N/A	#N/A
TRINITY_DN15989_c0_g1_i4	GLUBP_ARATH	-0.69	5.46	1.3E-03	3.7E-02	#N/A	#N/A	#N/A	#N/A	#N/A	#N/A	#N/A	#N/A
TRINITY_DN18971_c1_g1_i9	RL6_MESCR	-0.69	5.29	1.5E-03	4.1E-02	#N/A	#N/A	#N/A	#N/A	#N/A	#N/A	#N/A	#N/A
TRINITY_DN13122_c0_g1_i1	PP344_ARATH	-0.69	4.74	1.8E-03	4.9E-02	#N/A	#N/A	#N/A	#N/A	#N/A	#N/A	#N/A	#N/A
TRINITY_DN20834_c0_g1_i13	RUBB_PEA	-0.69	7.56	5.8E-04	2.0E-02	#N/A	#N/A	#N/A	#N/A	#N/A	#N/A	#N/A	#N/A
TRINITY_DN17346_c0_g7_i5	GYRB_ORYSJ	-0.68	5.30	1.5E-03	4.2E-02	#N/A	#N/A	#N/A	#N/A	#N/A	#N/A	#N/A	#N/A
TRINITY_DN14650_c1_g5_i4	KPYC_TOBAC	-0.68	8.42	1.6E-03	4.3E-02	#N/A	#N/A	#N/A	#N/A	#N/A	#N/A	#N/A	#N/A
TRINITY_DN21041_c0_g2_i6	CB12_PETHY	-0.68	5.77	8.2E-04	2.6E-02	#N/A	#N/A	#N/A	#N/A	#N/A	#N/A	#N/A	#N/A
TRINITY_DN22725_c1_g1_i8	EF1A_ORYSJ	-0.68	6.93	1.7E-03	4.6E-02	#N/A	#N/A	#N/A	#N/A	#N/A	#N/A	#N/A	#N/A
TRINITY_DN19095_c0_g1_i4	T184C_XENLA	-0.67	6.44	1.0E-03	3.2E-02	#N/A	#N/A	#N/A	#N/A	#N/A	#N/A	#N/A	#N/A
TRINITY_DN22418_c0_g2_i13	DCUP1_ORYSJ	-0.67	5.38	1.8E-03	4.8E-02	#N/A	#N/A	#N/A	#N/A	#N/A	#N/A	#N/A	#N/A
TRINITY_DN18763_c0_g4_i6	DTC_ARATH	-0.66	7.22	1.5E-03	4.2E-02	#N/A	#N/A	#N/A	#N/A	#N/A	#N/A	#N/A	#N/A
TRINITY_DN17417_c0_g1_i7	ODO2A_ARATH	-0.66	5.60	1.5E-03	4.1E-02	#N/A	#N/A	#N/A	#N/A	#N/A	#N/A	#N/A	#N/A
TRINITY_DN16513_c1_g2_i11	D14L_ORYSJ	0.64	5.54	2.0E-03	5.4E-02	#N/A	#N/A	#N/A	#N/A	#N/A	#N/A	#N/A	#N/A
TRINITY_DN18555_c1_g3_i2	YGBJ_ECOLI	0.71	5.34	1.3E-03	3.7E-02	#N/A	#N/A	#N/A	#N/A	#N/A	#N/A	#N/A	#N/A
TRINITY_DN17396_c3_g3_i6	TMN1_ARATH	0.71	5.22	1.0E-03	3.1E-02	#N/A	#N/A	#N/A	#N/A	#N/A	#N/A	#N/A	#N/A
TRINITY_DN20522_c0_g5_i2	CYB5_ORYSJ	0.72	4.22	2.0E-03	5.2E-02	#N/A	#N/A	#N/A	#N/A	#N/A	#N/A	#N/A	#N/A
TRINITY_DN15169_c0_g1_i3	SCP19_ARATH	0.72	4.95	1.9E-03	5.1E-02	#N/A	#N/A	#N/A	#N/A	#N/A	#N/A	#N/A	#N/A
TRINITY_DN20887_c0_g2_i1	PLDG1_ARATH	0.72	5.29	7.5E-04	2.5E-02	#N/A	#N/A	#N/A	#N/A	#N/A	#N/A	#N/A	#N/A
TRINITY_DN18557_c0_g1_i3	ACR4_ARATH	0.73	7.23	1.7E-03	4.7E-02	#N/A	#N/A	#N/A	#N/A	#N/A	#N/A	#N/A	#N/A
TRINITY_DN18398_c3_g1_i1	U88F4_MALDO	0.73	6.49	1.1E-03	3.4E-02	#N/A	#N/A	#N/A	#N/A	#N/A	#N/A	#N/A	#N/A
TRINITY_DN15682_c0_g2_i19	NLP1_ORYSJ	0.74	7.06	8.0E-04	2.6E-02	#N/A	#N/A	#N/A	#N/A	#N/A	#N/A	#N/A	#N/A
TRINITY_DN19319_c0_g3_i2	MANA_CANEN	0.75	4.48	1.5E-03	4.3E-02	#N/A	#N/A	#N/A	#N/A	#N/A	#N/A	#N/A	#N/A
TRINITY_DN17830_c1_g2_i2	P2C55_ARATH	0.75	4.64	1.9E-03	5.0E-02	#N/A	#N/A	#N/A	#N/A	#N/A	#N/A	#N/A	#N/A
TRINITY_DN21269_c0_g1_i29	BOR4_ARATH	0.75	7.27	2.6E-04	1.1E-02	#N/A	#N/A	#N/A	#N/A	#N/A	#N/A	#N/A	#N/A
TRINITY_DN15690_c1_g2_i7	TNG2_MOUSE	0.76	4.22	1.3E-03	3.7E-02	#N/A	#N/A	#N/A	#N/A	#N/A	#N/A	#N/A	#N/A
TRINITY_DN18394_c0_g1_i28	IN21B_ORYSJ	0.76	8.75	2.3E-04	9.4E-03	#N/A	#N/A	#N/A	#N/A	#N/A	#N/A	#N/A	#N/A
TRINITY_DN18470_c1_g1_i14	MTP1_ORYSJ	0.76	5.24	5.3E-04	1.9E-02	#N/A	#N/A	#N/A	#N/A	#N/A	#N/A	#N/A	#N/A
TRINITY_DN18539_c0_g2_i1	AAH_ORYSJ	0.76	5.47	3.2E-04	1.2E-02	#N/A	#N/A	#N/A	#N/A	#N/A	#N/A	#N/A	#N/A
TRINITY_DN22362_c2_g1_i8	MOC31_MAIZE	0.77	4.42	9.4E-04	2.9E-02	#N/A	#N/A	#N/A	#N/A	#N/A	#N/A	#N/A	#N/A
TRINITY_DN22066_c0_g2_i12	TIC_ARATH	0.77	5.58	1.5E-03	4.3E-02	#N/A	#N/A	#N/A	#N/A	#N/A	#N/A	#N/A	#N/A
TRINITY_DN21139_c0_g4_i2	U74F2_ARATH	0.78	5.79	1.4E-03	4.1E-02	#N/A	#N/A	#N/A	#N/A	#N/A	#N/A	#N/A	#N/A
TRINITY_DN21269_c0_g1_i8	BOR4_ARATH	0.79	5.75	1.2E-03	3.6E-02	#N/A	#N/A	#N/A	#N/A	#N/A	#N/A	#N/A	#N/A
TRINITY_DN18982_c0_g1_i4	HMCS_ARATH	0.79	4.15	2.0E-03	5.3E-02	#N/A	#N/A	#N/A	#N/A	#N/A	#N/A	#N/A	#N/A
TRINITY_DN17279_c0_g1_i8	SWT3A_ORYSJ	0.80	6.26	6.0E-04	2.1E-02	#N/A	#N/A	#N/A	#N/A	#N/A	#N/A	#N/A	#N/A
TRINITY_DN18861_c1_g2_i8	GCSP_SOLTU	0.80	8.55	1.5E-04	6.8E-03	#N/A	#N/A	#N/A	#N/A	#N/A	#N/A	#N/A	#N/A
TRINITY_DN18557_c0_g1_i5	ACR5_ARATH	0.84	4.58	2.1E-04	8.6E-03	#N/A	#N/A	#N/A	#N/A	#N/A	#N/A	#N/A	#N/A
TRINITY_DN21574_c0_g1_i5	HST_TOBAC	0.85	3.92	1.3E-03	3.7E-02	#N/A	#N/A	#N/A	#N/A	#N/A	#N/A	#N/A	#N/A
TRINITY_DN18394_c0_g1_i4	IN21B_ORYSJ	0.85	8.02	7.5E-05	3.6E-03	#N/A	#N/A	#N/A	#N/A	#N/A	#N/A	#N/A	#N/A

DET	Subject id	c9TR vs c7NT				c7TR vs c7NT				c7TR vs c9TR			
		Log_2	Log_2CPM	PValue	19FDR	Log_2	Log_2CPM	PValue	19FDR	Log_2	Log_2CPM	PValue	19FDR
TRINITY_DN18539_c0_g2_i2	AAH_ORYSJ	0.86	4.15	8.5E-04	2.7E-02	#N/A	#N/A	#N/A	#N/A	#N/A	#N/A	#N/A	#N/A
TRINITY_DN20521_c0_g1_i4	PUM5_ARATH	0.86	5.22	1.1E-03	3.4E-02	#N/A	#N/A	#N/A	#N/A	#N/A	#N/A	#N/A	#N/A
TRINITY_DN19035_c1_g1_i2	GLR31_ORYSJ	0.86	5.06	1.4E-03	4.0E-02	#N/A	#N/A	#N/A	#N/A	#N/A	#N/A	#N/A	#N/A
TRINITY_DN20067_c1_g1_i1	GIGAN_ORYSJ	0.87	5.67	2.0E-03	5.4E-02	#N/A	#N/A	#N/A	#N/A	#N/A	#N/A	#N/A	#N/A
TRINITY_DN15135_c1_g4_i2	HOX5_ORYSJ	0.87	5.05	2.5E-04	1.0E-02	#N/A	#N/A	#N/A	#N/A	#N/A	#N/A	#N/A	#N/A
TRINITY_DN18539_c0_g1_i6	AAH_ORYSJ	0.87	7.19	1.4E-03	4.0E-02	#N/A	#N/A	#N/A	#N/A	#N/A	#N/A	#N/A	#N/A
TRINITY_DN18851_c1_g2_i1	TMN1_ARATH	0.88	3.53	1.2E-03	3.6E-02	#N/A	#N/A	#N/A	#N/A	#N/A	#N/A	#N/A	#N/A
TRINITY_DN14712_c0_g3_i2	CHMP1_ORYSJ	0.88	5.20	4.7E-05	2.4E-03	#N/A	#N/A	#N/A	#N/A	#N/A	#N/A	#N/A	#N/A
TRINITY_DN21255_c0_g1_i6	AVT1_YEAST	0.88	4.76	1.3E-03	3.8E-02	#N/A	#N/A	#N/A	#N/A	#N/A	#N/A	#N/A	#N/A
TRINITY_DN15652_c0_g1_i7	Y2309_ARATH	0.88	5.00	7.0E-04	2.3E-02	#N/A	#N/A	#N/A	#N/A	#N/A	#N/A	#N/A	#N/A
TRINITY_DN15876_c0_g1_i17	AAP7_ARATH	0.90	3.31	1.3E-03	3.9E-02	#N/A	#N/A	#N/A	#N/A	#N/A	#N/A	#N/A	#N/A
TRINITY_DN16224_c0_g1_i6	Y4958_ARATH	0.90	5.24	6.5E-05	3.2E-03	#N/A	#N/A	#N/A	#N/A	#N/A	#N/A	#N/A	#N/A
TRINITY_DN15652_c0_g1_i1	Y2309_ARATH	0.90	5.96	2.0E-04	8.3E-03	#N/A	#N/A	#N/A	#N/A	#N/A	#N/A	#N/A	#N/A
TRINITY_DN18728_c2_g3_i2	ORR6_ORYSI	0.91	5.16	1.0E-04	4.6E-03	#N/A	#N/A	#N/A	#N/A	#N/A	#N/A	#N/A	#N/A
TRINITY_DN21372_c0_g2_i1	EBF1_ARATH	0.91	4.44	1.2E-04	5.4E-03	#N/A	#N/A	#N/A	#N/A	#N/A	#N/A	#N/A	#N/A
TRINITY_DN18050_c0_g1_i1	NAC41_ARATH	0.91	7.64	1.1E-04	5.2E-03	#N/A	#N/A	#N/A	#N/A	#N/A	#N/A	#N/A	#N/A
TRINITY_DN18539_c0_g1_i9	AAH_ORYSJ	0.91	4.10	6.3E-04	2.1E-02	#N/A	#N/A	#N/A	#N/A	#N/A	#N/A	#N/A	#N/A
TRINITY_DN16224_c0_g1_i9	Y4958_ARATH	0.91	5.61	7.6E-04	2.5E-02	#N/A	#N/A	#N/A	#N/A	#N/A	#N/A	#N/A	#N/A
TRINITY_DN17212_c0_g3_i7	DHBK_SOLLC	0.91	4.15	3.9E-04	1.5E-02	#N/A	#N/A	#N/A	#N/A	#N/A	#N/A	#N/A	#N/A
TRINITY_DN18519_c0_g1_i8	TIP42_MAIZE	0.92	6.88	1.3E-03	3.7E-02	#N/A	#N/A	#N/A	#N/A	#N/A	#N/A	#N/A	#N/A
TRINITY_DN17984_c0_g1_i2	NAC67_ORYSJ	0.92	5.44	6.3E-04	2.1E-02	#N/A	#N/A	#N/A	#N/A	#N/A	#N/A	#N/A	#N/A
TRINITY_DN19154_c0_g1_i3	RING1_GOSHI	0.93	5.82	9.7E-06	5.9E-04	#N/A	#N/A	#N/A	#N/A	#N/A	#N/A	#N/A	#N/A
TRINITY_DN17466_c0_g1_i8	RADL3_ARATH	0.93	4.43	3.0E-04	1.2E-02	#N/A	#N/A	#N/A	#N/A	#N/A	#N/A	#N/A	#N/A
TRINITY_DN14732_c0_g4_i1	MAD14_ORYSJ	0.94	4.36	1.2E-03	3.6E-02	#N/A	#N/A	#N/A	#N/A	#N/A	#N/A	#N/A	#N/A
TRINITY_DN16022_c0_g1_i10	CIPKV_ORYSJ	0.95	5.02	2.1E-03	5.5E-02	#N/A	#N/A	#N/A	#N/A	#N/A	#N/A	#N/A	#N/A
TRINITY_DN20219_c0_g1_i11	UBL5_ARATH	0.95	5.16	1.0E-05	6.3E-04	#N/A	#N/A	#N/A	#N/A	#N/A	#N/A	#N/A	#N/A
TRINITY_DN19334_c2_g3_i3	HNRPQ_RAT	0.96	3.88	4.7E-04	1.7E-02	#N/A	#N/A	#N/A	#N/A	#N/A	#N/A	#N/A	#N/A
TRINITY_DN15827_c1_g3_i5	SSL13_ARATH	0.96	2.93	1.5E-03	4.3E-02	#N/A	#N/A	#N/A	#N/A	#N/A	#N/A	#N/A	#N/A
TRINITY_DN14516_c2_g1_i4	HA22A_ARATH	0.97	4.76	4.3E-05	2.2E-03	#N/A	#N/A	#N/A	#N/A	#N/A	#N/A	#N/A	#N/A
TRINITY_DN19955_c0_g1_i15	F16P2_ORYCO	0.98	4.55	4.2E-04	1.5E-02	#N/A	#N/A	#N/A	#N/A	#N/A	#N/A	#N/A	#N/A
TRINITY_DN15567_c1_g1_i2	FLK_ARATH	0.98	3.21	1.0E-03	3.1E-02	#N/A	#N/A	#N/A	#N/A	#N/A	#N/A	#N/A	#N/A
TRINITY_DN22823_c3_g4_i2	C7A15_ARATH	0.99	7.39	1.4E-03	4.0E-02	#N/A	#N/A	#N/A	#N/A	#N/A	#N/A	#N/A	#N/A
TRINITY_DN21761_c3_g1_i2	CERK1_ORYSJ	0.99	3.82	4.6E-04	1.7E-02	#N/A	#N/A	#N/A	#N/A	#N/A	#N/A	#N/A	#N/A
TRINITY_DN22642_c2_g2_i7	ATG2_ARATH	0.99	4.55	5.7E-04	2.0E-02	#N/A	#N/A	#N/A	#N/A	#N/A	#N/A	#N/A	#N/A
TRINITY_DN18539_c0_g1_i11	AAH_ORYSJ	1.00	4.51	6.1E-04	2.1E-02	#N/A	#N/A	#N/A	#N/A	#N/A	#N/A	#N/A	#N/A
TRINITY_DN17283_c1_g3_i3	AB14C_ARATH	1.00	2.86	1.5E-03	4.2E-02	#N/A	#N/A	#N/A	#N/A	#N/A	#N/A	#N/A	#N/A
TRINITY_DN21413_c1_g1_i21	DTX27_ARATH	1.00	3.71	3.5E-04	1.3E-02	#N/A	#N/A	#N/A	#N/A	#N/A	#N/A	#N/A	#N/A
TRINITY_DN16944_c0_g1_i3	IQD1_ARATH	1.00	3.68	5.7E-04	2.0E-02	#N/A	#N/A	#N/A	#N/A	#N/A	#N/A	#N/A	#N/A
TRINITY_DN19989_c0_g2_i20	CRK10_ARATH	1.01	5.50	6.6E-06	4.1E-04	#N/A	#N/A	#N/A	#N/A	#N/A	#N/A	#N/A	#N/A
TRINITY_DN14609_c0_g7_i1	C81E1_GLYEC	1.01	4.39	9.1E-04	2.9E-02	#N/A	#N/A	#N/A	#N/A	#N/A	#N/A	#N/A	#N/A
TRINITY_DN22334_c1_g1_i6	PEX6_ARATH	1.01	3.98	4.9E-04	1.8E-02	#N/A	#N/A	#N/A	#N/A	#N/A	#N/A	#N/A	#N/A
TRINITY_DN22281_c1_g1_i2	ACBP5_ARATH	1.01	3.44	6.0E-04	2.1E-02	#N/A	#N/A	#N/A	#N/A	#N/A	#N/A	#N/A	#N/A
TRINITY_DN22669_c0_g2_i2	GIGAN_ORYSJ	1.02	6.19	5.4E-05	2.7E-03	#N/A	#N/A	#N/A	#N/A	#N/A	#N/A	#N/A	#N/A
TRINITY_DN20943_c0_g2_i4	YQ77_SCHPO	1.02	6.63	5.2E-04	1.9E-02	#N/A	#N/A	#N/A	#N/A	#N/A	#N/A	#N/A	#N/A
TRINITY_DN16377_c1_g2_i15	BGL31_ORYSJ	1.02	3.44	1.6E-04	6.9E-03	#N/A	#N/A	#N/A	#N/A	#N/A	#N/A	#N/A	#N/A
TRINITY_DN18316_c0_g1_i3	ACT2_ORYSJ	1.03	4.08	1.7E-04	7.3E-03	#N/A	#N/A	#N/A	#N/A	#N/A	#N/A	#N/A	#N/A
TRINITY_DN15878_c0_g1_i13	Y5564_ARATH	1.03	3.09	1.8E-03	4.9E-02	#N/A	#N/A	#N/A	#N/A	#N/A	#N/A	#N/A	#N/A
TRINITY_DN21922_c0_g4_i5	BLH6_ARATH	1.03	6.85	1.6E-03	4.4E-02	#N/A	#N/A	#N/A	#N/A	#N/A	#N/A	#N/A	#N/A
TRINITY_DN16022_c0_g1_i1	CIPKV_ORYSJ	1.04	8.19	3.2E-05	1.7E-03	#N/A	#N/A	#N/A	#N/A	#N/A	#N/A	#N/A	#N/A
TRINITY_DN15876_c0_g1_i13	AAP7_ARATH	1.04	5.87	4.7E-07	3.9E-05	#N/A	#N/A	#N/A	#N/A	#N/A	#N/A	#N/A	#N/A
TRINITY_DN17435_c1_g1_i1	IBTK_HUMAN	1.05	5.19	1.1E-03	3.4E-02	#N/A	#N/A	#N/A	#N/A	#N/A	#N/A	#N/A	#N/A
TRINITY_DN18050_c0_g1_i2	NAC41_ARATH	1.06	4.81	2.8E-05	1.5E-03	#N/A	#N/A	#N/A	#N/A	#N/A	#N/A	#N/A	#N/A
TRINITY_DN19473_c2_g1_i2	ANTL1_ARATH	1.07	3.01	1.7E-03	4.7E-02	#N/A	#N/A	#N/A	#N/A	#N/A	#N/A	#N/A	#N/A
TRINITY_DN16833_c2_g2_i3	GSTU6_ORYSJ	1.07	3.17	6.8E-04	2.3E-02	#N/A	#N/A	#N/A	#N/A	#N/A	#N/A	#N/A	#N/A
TRINITY_DN22669_c0_g2_i10	GIGAN_ORYSJ	1.07	4.26	2.9E-05	1.6E-03	#N/A	#N/A	#N/A	#N/A	#N/A	#N/A	#N/A	#N/A
TRINITY_DN18861_c1_g2_i1	GCSP_SOLTU	1.07	3.13	6.0E-04	2.1E-02	#N/A	#N/A	#N/A	#N/A	#N/A	#N/A	#N/A	#N/A
TRINITY_DN15899_c0_g2_i4	C81E1_GLYEC	1.08	5.16	4.2E-04	1.5E-02	#N/A	#N/A	#N/A	#N/A	#N/A	#N/A	#N/A	#N/A
TRINITY_DN18360_c1_g1_i4	ARFA_ORYSJ	1.10	3.81	2.0E-04	8.3E-03	#N/A	#N/A	#N/A	#N/A	#N/A	#N/A	#N/A	#N/A
TRINITY_DN19182_c0_g1_i5	PAO_ARATH	1.11	5.62	2.1E-05	1.2E-03	#N/A	#N/A	#N/A	#N/A	#N/A	#N/A	#N/A	#N/A
TRINITY_DN19955_c0_g1_i8	F16P2_ORYCO	1.12	3.51	3.9E-04	1.5E-02	#N/A	#N/A	#N/A	#N/A	#N/A	#N/A	#N/A	#N/A
TRINITY_DN15780_c2_g1_i3	ACDH_ARATH	1.12	3.21	1.7E-04	7.2E-03	#N/A	#N/A	#N/A	#N/A	#N/A	#N/A	#N/A	#N/A

DET	Subject id	c9TR vs c7NT				c7TR vs c7NT				c7TR vs c9TR			
		Log_2	Log_2CPM	PValue	19FDR	Log_2	Log_2CPM	PValue	19FDR	Log_2	Log_2CPM	PValue	19FDR
TRINITY_DN21021_c0_g2_i1	CALSC_ARATH	1.13	2.87	9.1E-04	2.9E-02	#N/A	#N/A	#N/A	#N/A	#N/A	#N/A	#N/A	#N/A
TRINITY_DN16634_c0_g1_i1	PGLT1_HUMAN	1.13	2.37	9.5E-04	3.0E-02	#N/A	#N/A	#N/A	#N/A	#N/A	#N/A	#N/A	#N/A
TRINITY_DN17279_c0_g1_i5	SWT3A_ORYSJ	1.14	2.54	7.0E-04	2.3E-02	#N/A	#N/A	#N/A	#N/A	#N/A	#N/A	#N/A	#N/A
TRINITY_DN17659_c0_g1_i1	APT2_ARATH	1.14	5.92	1.0E-03	3.1E-02	#N/A	#N/A	#N/A	#N/A	#N/A	#N/A	#N/A	#N/A
TRINITY_DN18519_c0_g1_i5	TIP42_MAIZE	1.14	5.14	1.6E-05	9.0E-04	#N/A	#N/A	#N/A	#N/A	#N/A	#N/A	#N/A	#N/A
TRINITY_DN17770_c0_g2_i15	PEX31_ARATH	1.14	4.36	1.0E-05	6.2E-04	#N/A	#N/A	#N/A	#N/A	#N/A	#N/A	#N/A	#N/A
TRINITY_DN16690_c2_g10_i2	C7A15_ARATH	1.14	7.08	7.6E-05	3.6E-03	#N/A	#N/A	#N/A	#N/A	#N/A	#N/A	#N/A	#N/A
TRINITY_DN17850_c0_g1_i3	DTX40_ARATH	1.14	5.11	6.7E-07	5.3E-05	#N/A	#N/A	#N/A	#N/A	#N/A	#N/A	#N/A	#N/A
TRINITY_DN16869_c1_g1_i7	CZOG1_MAIZE	1.15	2.24	1.3E-03	3.7E-02	#N/A	#N/A	#N/A	#N/A	#N/A	#N/A	#N/A	#N/A
TRINITY_DN17407_c0_g3_i8	CLPX1_ARATH	1.15	2.95	1.6E-03	4.5E-02	#N/A	#N/A	#N/A	#N/A	#N/A	#N/A	#N/A	#N/A
TRINITY_DN19919_c1_g1_i19	RPM1_ARATH	1.15	2.46	2.1E-03	5.5E-02	#N/A	#N/A	#N/A	#N/A	#N/A	#N/A	#N/A	#N/A
TRINITY_DN19418_c1_g1_i5	PSAA_SORBI	1.15	4.17	7.5E-04	2.5E-02	#N/A	#N/A	#N/A	#N/A	#N/A	#N/A	#N/A	#N/A
TRINITY_DN16757_c0_g2_i7	MON2_DROPS	1.15	4.35	3.9E-04	1.5E-02	#N/A	#N/A	#N/A	#N/A	#N/A	#N/A	#N/A	#N/A
TRINITY_DN18539_c0_g1_i7	AAH_ORYSJ	1.15	3.62	1.2E-05	7.1E-04	#N/A	#N/A	#N/A	#N/A	#N/A	#N/A	#N/A	#N/A
TRINITY_DN22871_c7_g3_i1	POLX_TOBAC	1.16	3.77	1.7E-03	4.6E-02	#N/A	#N/A	#N/A	#N/A	#N/A	#N/A	#N/A	#N/A
TRINITY_DN16022_c0_g1_i19	CIPKV_ORYSJ	1.17	5.52	1.7E-04	7.3E-03	#N/A	#N/A	#N/A	#N/A	#N/A	#N/A	#N/A	#N/A
TRINITY_DN19955_c0_g1_i7	F16P2_ORYCO	1.17	3.51	1.3E-04	5.9E-03	#N/A	#N/A	#N/A	#N/A	#N/A	#N/A	#N/A	#N/A
TRINITY_DN21309_c1_g1_i20	SMG8_BOVIN	1.18	2.91	1.3E-03	3.7E-02	#N/A	#N/A	#N/A	#N/A	#N/A	#N/A	#N/A	#N/A
TRINITY_DN18578_c0_g1_i5	APG_ORYSJ	1.18	3.04	4.7E-05	2.4E-03	#N/A	#N/A	#N/A	#N/A	#N/A	#N/A	#N/A	#N/A
TRINITY_DN18539_c0_g1_i3	AAH_ORYSJ	1.18	3.27	1.5E-04	6.8E-03	#N/A	#N/A	#N/A	#N/A	#N/A	#N/A	#N/A	#N/A
TRINITY_DN16232_c1_g2_i11	ODBA2_ARATH	1.19	4.76	2.6E-04	1.0E-02	#N/A	#N/A	#N/A	#N/A	#N/A	#N/A	#N/A	#N/A
TRINITY_DN20282_c1_g1_i4	GLE1_ARATH	1.19	4.00	3.2E-06	2.1E-04	#N/A	#N/A	#N/A	#N/A	#N/A	#N/A	#N/A	#N/A
TRINITY_DN20763_c0_g1_i1	TAF1_ORYSJ	1.19	4.30	2.5E-04	1.0E-02	#N/A	#N/A	#N/A	#N/A	#N/A	#N/A	#N/A	#N/A
TRINITY_DN15712_c0_g2_i17	PUP3_ARATH	1.19	2.59	8.3E-04	2.7E-02	#N/A	#N/A	#N/A	#N/A	#N/A	#N/A	#N/A	#N/A
TRINITY_DN18539_c0_g1_i1	AAH_ORYSJ	1.20	5.65	4.3E-07	3.5E-05	#N/A	#N/A	#N/A	#N/A	#N/A	#N/A	#N/A	#N/A
TRINITY_DN19496_c0_g2_i12	PEX14_ARATH	1.20	3.27	6.8E-05	3.3E-03	#N/A	#N/A	#N/A	#N/A	#N/A	#N/A	#N/A	#N/A
TRINITY_DN15411_c0_g1_i11	CYSKP_SOLTU	1.20	2.89	8.9E-04	2.8E-02	#N/A	#N/A	#N/A	#N/A	#N/A	#N/A	#N/A	#N/A
TRINITY_DN19465_c1_g9_i2	NAC48_ORYSJ	1.21	5.90	2.9E-08	3.0E-06	#N/A	#N/A	#N/A	#N/A	#N/A	#N/A	#N/A	#N/A
TRINITY_DN22530_c2_g1_i7	LIN1_NYCCO	1.22	2.83	1.6E-03	4.3E-02	#N/A	#N/A	#N/A	#N/A	#N/A	#N/A	#N/A	#N/A
TRINITY_DN15876_c0_g1_i4	AAP7_ARATH	1.23	2.51	6.2E-04	2.1E-02	#N/A	#N/A	#N/A	#N/A	#N/A	#N/A	#N/A	#N/A
TRINITY_DN16097_c0_g1_i6	MSSP2_ARATH	1.23	5.90	4.6E-08	4.6E-06	#N/A	#N/A	#N/A	#N/A	#N/A	#N/A	#N/A	#N/A
TRINITY_DN17032_c1_g2_i1	DTX39_ARATH	1.23	5.91	5.8E-09	6.6E-07	#N/A	#N/A	#N/A	#N/A	#N/A	#N/A	#N/A	#N/A
TRINITY_DN16296_c0_g3_i1	FARS_ZEADI	1.24	2.98	2.0E-03	5.2E-02	#N/A	#N/A	#N/A	#N/A	#N/A	#N/A	#N/A	#N/A
TRINITY_DN15791_c0_g3_i4	ERF71_ARATH	1.24	1.81	1.9E-03	5.2E-02	#N/A	#N/A	#N/A	#N/A	#N/A	#N/A	#N/A	#N/A
TRINITY_DN18236_c0_g1_i4	Y3078_ARATH	1.24	4.10	1.1E-03	3.3E-02	#N/A	#N/A	#N/A	#N/A	#N/A	#N/A	#N/A	#N/A
TRINITY_DN20789_c0_g3_i9	MAN8_ORYSJ	1.26	1.80	1.7E-03	4.6E-02	#N/A	#N/A	#N/A	#N/A	#N/A	#N/A	#N/A	#N/A
TRINITY_DN19465_c1_g9_i3	NAC48_ORYSJ	1.26	5.83	7.6E-09	8.5E-07	#N/A	#N/A	#N/A	#N/A	#N/A	#N/A	#N/A	#N/A
TRINITY_DN22778_c4_g1_i6	POLX_TOBAC	1.26	5.14	3.8E-05	2.0E-03	#N/A	#N/A	#N/A	#N/A	#N/A	#N/A	#N/A	#N/A
TRINITY_DN19322_c0_g3_i2	MAD14_ORYSJ	1.27	4.86	7.4E-07	5.8E-05	#N/A	#N/A	#N/A	#N/A	#N/A	#N/A	#N/A	#N/A
TRINITY_DN22678_c1_g1_i16	CLASP_ARATH	1.27	2.40	1.7E-03	4.7E-02	#N/A	#N/A	#N/A	#N/A	#N/A	#N/A	#N/A	#N/A
TRINITY_DN18539_c0_g1_i19	AAH_ORYSJ	1.28	2.76	8.3E-05	4.0E-03	#N/A	#N/A	#N/A	#N/A	#N/A	#N/A	#N/A	#N/A
TRINITY_DN15903_c0_g1_i13	ACCO1_ORYSI	1.28	4.23	1.3E-04	5.7E-03	#N/A	#N/A	#N/A	#N/A	#N/A	#N/A	#N/A	#N/A
TRINITY_DN15638_c1_g1_i10	DTX40_ARATH	1.29	3.71	3.3E-04	1.2E-02	#N/A	#N/A	#N/A	#N/A	#N/A	#N/A	#N/A	#N/A
TRINITY_DN19680_c0_g2_i1	UGT1_GARJA	1.30	4.24	2.3E-04	9.6E-03	#N/A	#N/A	#N/A	#N/A	#N/A	#N/A	#N/A	#N/A
TRINITY_DN15960_c0_g1_i11	ZIP7_ORYSJ	1.31	6.15	3.5E-10	4.8E-08	#N/A	#N/A	#N/A	#N/A	#N/A	#N/A	#N/A	#N/A
TRINITY_DN22457_c1_g1_i8	TBB2_ORYSJ	1.31	1.38	1.4E-03	4.1E-02	#N/A	#N/A	#N/A	#N/A	#N/A	#N/A	#N/A	#N/A
TRINITY_DN22705_c0_g2_i3	ALDO2_MAIZE	1.31	6.87	9.3E-04	2.9E-02	#N/A	#N/A	#N/A	#N/A	#N/A	#N/A	#N/A	#N/A
TRINITY_DN18040_c3_g5_i1	ASPGB_ARATH	1.31	5.07	7.1E-08	7.0E-06	#N/A	#N/A	#N/A	#N/A	#N/A	#N/A	#N/A	#N/A
TRINITY_DN15273_c1_g1_i10	2NPD_BACSU	1.32	3.20	1.6E-03	4.5E-02	#N/A	#N/A	#N/A	#N/A	#N/A	#N/A	#N/A	#N/A
TRINITY_DN20586_c0_g2_i8	NIR_MAIZE	1.32	2.48	1.8E-03	4.9E-02	#N/A	#N/A	#N/A	#N/A	#N/A	#N/A	#N/A	#N/A
TRINITY_DN15213_c1_g5_i1	U83A1_ARATH	1.32	2.18	4.5E-04	1.7E-02	#N/A	#N/A	#N/A	#N/A	#N/A	#N/A	#N/A	#N/A
TRINITY_DN16022_c0_g1_i21	CIPKV_ORYSJ	1.32	3.74	1.5E-05	8.5E-04	#N/A	#N/A	#N/A	#N/A	#N/A	#N/A	#N/A	#N/A
TRINITY_DN14718_c0_g6_i1	SRG1_ARATH	1.33	2.39	3.1E-04	1.2E-02	#N/A	#N/A	#N/A	#N/A	#N/A	#N/A	#N/A	#N/A
TRINITY_DN21808_c1_g3_i5	ARF_VIGUN	1.33	1.92	3.3E-04	1.3E-02	#N/A	#N/A	#N/A	#N/A	#N/A	#N/A	#N/A	#N/A
TRINITY_DN19906_c0_g2_i2	CB60C_ARATH	1.34	2.50	1.0E-03	3.1E-02	#N/A	#N/A	#N/A	#N/A	#N/A	#N/A	#N/A	#N/A
TRINITY_DN19035_c1_g1_i8	GLR31_ORYSJ	1.34	2.34	1.5E-03	4.3E-02	#N/A	#N/A	#N/A	#N/A	#N/A	#N/A	#N/A	#N/A
TRINITY_DN16873_c0_g2_i6	U83A1_ARATH	1.34	2.99	1.5E-03	4.2E-02	#N/A	#N/A	#N/A	#N/A	#N/A	#N/A	#N/A	#N/A
TRINITY_DN21179_c0_g2_i17	CDT1A_ARATH	1.36	3.16	5.0E-06	3.3E-04	#N/A	#N/A	#N/A	#N/A	#N/A	#N/A	#N/A	#N/A
TRINITY_DN19465_c1_g9_i1	NAC48_ORYSJ	1.36	2.37	3.4E-05	1.8E-03	#N/A	#N/A	#N/A	#N/A	#N/A	#N/A	#N/A	#N/A
TRINITY_DN22454_c0_g1_i8	OHK4_ORYSJ	1.36	4.64	5.7E-05	2.8E-03	#N/A	#N/A	#N/A	#N/A	#N/A	#N/A	#N/A	#N/A
TRINITY_DN19064_c0_g2_i6	U85A8_STERE	1.37	2.19	7.3E-04	2.4E-02	#N/A	#N/A	#N/A	#N/A	#N/A	#N/A	#N/A	#N/A

DET	Subject id	c9TR vs c7NT				c7TR vs c7NT				c7TR vs c9TR			
		Log_2	Log_2CPM	PValue	19FDR	Log_2	Log_2CPM	PValue	19FDR	Log_2	Log_2CPM	PValue	19FDR
TRINITY_DN16650_c1_g8_i1	UGT1_GARJA	1.37	3.75	1.6E-04	6.9E-03	#N/A	#N/A	#N/A	#N/A	#N/A	#N/A	#N/A	#N/A
TRINITY_DN20536_c0_g1_i11	SOL1_ARATH	1.37	3.17	5.2E-05	2.6E-03	#N/A	#N/A	#N/A	#N/A	#N/A	#N/A	#N/A	#N/A
TRINITY_DN17441_c0_g3_i2	YIPL1_ARATH	1.37	2.84	5.7E-05	2.9E-03	#N/A	#N/A	#N/A	#N/A	#N/A	#N/A	#N/A	#N/A
TRINITY_DN18557_c0_g1_i1	ACR6_ARATH	1.38	2.92	6.0E-04	2.1E-02	#N/A	#N/A	#N/A	#N/A	#N/A	#N/A	#N/A	#N/A
TRINITY_DN17258_c0_g4_i7	GLR34_ARATH	1.38	3.60	2.6E-04	1.0E-02	#N/A	#N/A	#N/A	#N/A	#N/A	#N/A	#N/A	#N/A
TRINITY_DN18541_c0_g1_i29	AB2C_ARATH	1.38	3.40	4.7E-05	2.4E-03	#N/A	#N/A	#N/A	#N/A	#N/A	#N/A	#N/A	#N/A
TRINITY_DN17296_c2_g6_i4	CALSA_ARATH	1.39	3.39	2.0E-03	5.2E-02	#N/A	#N/A	#N/A	#N/A	#N/A	#N/A	#N/A	#N/A
TRINITY_DN15381_c1_g3_i4	CBSX3_ARATH	1.42	4.79	5.8E-04	2.0E-02	#N/A	#N/A	#N/A	#N/A	#N/A	#N/A	#N/A	#N/A
TRINITY_DN20729_c0_g1_i4	EXLA2_ORYSJ	1.42	2.04	2.1E-04	8.8E-03	#N/A	#N/A	#N/A	#N/A	#N/A	#N/A	#N/A	#N/A
TRINITY_DN21132_c1_g2_i4	CAX1A_ORYSJ	1.44	3.94	4.5E-06	2.9E-04	#N/A	#N/A	#N/A	#N/A	#N/A	#N/A	#N/A	#N/A
TRINITY_DN16017_c0_g1_i4	TPS6_ARATH	1.44	3.76	3.1E-05	1.7E-03	#N/A	#N/A	#N/A	#N/A	#N/A	#N/A	#N/A	#N/A
TRINITY_DN12090_c0_g1_i2	SM3L3_ARATH	1.44	1.07	1.9E-03	5.1E-02	#N/A	#N/A	#N/A	#N/A	#N/A	#N/A	#N/A	#N/A
TRINITY_DN20445_c0_g2_i14	ALN_ORYSJ	1.44	2.04	5.8E-04	2.0E-02	#N/A	#N/A	#N/A	#N/A	#N/A	#N/A	#N/A	#N/A
TRINITY_DN15411_c0_g2_i4	CYSKP_SOLTU	1.45	1.45	1.0E-03	3.1E-02	#N/A	#N/A	#N/A	#N/A	#N/A	#N/A	#N/A	#N/A
TRINITY_DN18950_c0_g3_i3	DRE2B_ORYSJ	1.45	2.49	1.9E-05	1.1E-03	#N/A	#N/A	#N/A	#N/A	#N/A	#N/A	#N/A	#N/A
TRINITY_DN22028_c0_g1_i11	CNIF1_ARATH	1.46	5.42	5.9E-09	6.7E-07	#N/A	#N/A	#N/A	#N/A	#N/A	#N/A	#N/A	#N/A
TRINITY_DN22385_c5_g10_i1	M810_ARATH	1.46	2.25	1.4E-04	6.2E-03	#N/A	#N/A	#N/A	#N/A	#N/A	#N/A	#N/A	#N/A
TRINITY_DN21276_c0_g1_i2	AB10C_ARATH	1.47	5.74	7.3E-04	2.4E-02	#N/A	#N/A	#N/A	#N/A	#N/A	#N/A	#N/A	#N/A
TRINITY_DN19340_c1_g6_i3	APX6_ARATH	1.47	3.19	2.1E-03	5.4E-02	#N/A	#N/A	#N/A	#N/A	#N/A	#N/A	#N/A	#N/A
TRINITY_DN22334_c1_g1_i16	PEX6_ARATH	1.48	2.30	7.0E-04	2.3E-02	#N/A	#N/A	#N/A	#N/A	#N/A	#N/A	#N/A	#N/A
TRINITY_DN16022_c0_g1_i12	CIPKV_ORYSJ	1.48	2.01	6.8E-04	2.3E-02	#N/A	#N/A	#N/A	#N/A	#N/A	#N/A	#N/A	#N/A
TRINITY_DN17872_c1_g8_i1	YG31B_YEAST	1.48	2.63	6.3E-06	4.0E-04	#N/A	#N/A	#N/A	#N/A	#N/A	#N/A	#N/A	#N/A
TRINITY_DN18145_c0_g3_i2	Y2060_ARATH	1.49	3.18	1.5E-04	6.7E-03	#N/A	#N/A	#N/A	#N/A	#N/A	#N/A	#N/A	#N/A
TRINITY_DN16071_c0_g2_i13	AGO1B_ORYSJ	1.49	2.14	5.7E-05	2.8E-03	#N/A	#N/A	#N/A	#N/A	#N/A	#N/A	#N/A	#N/A
TRINITY_DN21030_c0_g1_i15	F135B_XENLA	1.49	1.17	1.2E-03	3.5E-02	#N/A	#N/A	#N/A	#N/A	#N/A	#N/A	#N/A	#N/A
TRINITY_DN20067_c1_g1_i29	GIGAN_ORYSJ	1.50	3.82	1.8E-05	1.0E-03	#N/A	#N/A	#N/A	#N/A	#N/A	#N/A	#N/A	#N/A
TRINITY_DN22823_c3_g4_i4	C7A15_ARATH	1.50	5.52	2.0E-10	2.8E-08	#N/A	#N/A	#N/A	#N/A	#N/A	#N/A	#N/A	#N/A
TRINITY_DN20155_c0_g2_i2	UMPS1_ORYSJ	1.50	3.49	1.7E-06	1.2E-04	#N/A	#N/A	#N/A	#N/A	#N/A	#N/A	#N/A	#N/A
TRINITY_DN17989_c1_g1_i2	SALR_PAPBR	1.51	1.73	1.5E-04	6.5E-03	#N/A	#N/A	#N/A	#N/A	#N/A	#N/A	#N/A	#N/A
TRINITY_DN19827_c2_g2_i2	XRN4_ARATH	1.51	5.59	1.6E-12	2.9E-10	#N/A	#N/A	#N/A	#N/A	#N/A	#N/A	#N/A	#N/A
TRINITY_DN19035_c1_g1_i17	GLR31_ORYSJ	1.52	3.39	5.1E-06	3.3E-04	#N/A	#N/A	#N/A	#N/A	#N/A	#N/A	#N/A	#N/A
TRINITY_DN15638_c1_g1_i3	DTX36_ARATH	1.52	1.97	8.3E-04	2.7E-02	#N/A	#N/A	#N/A	#N/A	#N/A	#N/A	#N/A	#N/A
TRINITY_DN19182_c0_g1_i2	PAO_ARATH	1.52	1.35	4.0E-04	1.5E-02	#N/A	#N/A	#N/A	#N/A	#N/A	#N/A	#N/A	#N/A
TRINITY_DN20657_c0_g1_i5	VA721_ARATH	1.52	2.18	3.8E-05	2.0E-03	#N/A	#N/A	#N/A	#N/A	#N/A	#N/A	#N/A	#N/A
TRINITY_DN22705_c1_g7_i2	ALDO2_MAIZE	1.53	6.92	7.4E-06	4.6E-04	#N/A	#N/A	#N/A	#N/A	#N/A	#N/A	#N/A	#N/A
TRINITY_DN22869_c10_g1_i5	POL3_DROME	1.54	4.01	9.2E-08	8.9E-06	#N/A	#N/A	#N/A	#N/A	#N/A	#N/A	#N/A	#N/A
TRINITY_DN19779_c0_g1_i4	HAT1_MAIZE	1.55	2.07	4.4E-05	2.3E-03	#N/A	#N/A	#N/A	#N/A	#N/A	#N/A	#N/A	#N/A
TRINITY_DN20265_c0_g1_i4	MNS5_ARATH	1.55	2.71	8.2E-04	2.6E-02	#N/A	#N/A	#N/A	#N/A	#N/A	#N/A	#N/A	#N/A
TRINITY_DN21180_c1_g1_i6	UNE12_ARATH	1.56	1.64	6.4E-04	2.2E-02	#N/A	#N/A	#N/A	#N/A	#N/A	#N/A	#N/A	#N/A
TRINITY_DN21903_c0_g1_i16	Y1491_ARATH	1.56	0.93	1.5E-03	4.2E-02	#N/A	#N/A	#N/A	#N/A	#N/A	#N/A	#N/A	#N/A
TRINITY_DN18883_c0_g1_i1	ADF2_ORYSJ	1.56	1.22	1.6E-03	4.4E-02	#N/A	#N/A	#N/A	#N/A	#N/A	#N/A	#N/A	#N/A
TRINITY_DN21694_c1_g1_i31	CIPK9_ORYSJ	1.58	6.17	4.4E-04	1.6E-02	#N/A	#N/A	#N/A	#N/A	#N/A	#N/A	#N/A	#N/A
TRINITY_DN21584_c0_g1_i5	HLP_ARATH	1.58	1.59	5.8E-04	2.0E-02	#N/A	#N/A	#N/A	#N/A	#N/A	#N/A	#N/A	#N/A
TRINITY_DN25304_c0_g1_i1	BEBT_CLABR	1.59	0.92	1.8E-03	4.9E-02	#N/A	#N/A	#N/A	#N/A	#N/A	#N/A	#N/A	#N/A
TRINITY_DN15449_c0_g2_i9	WNK7_ORYSJ	1.59	3.57	1.6E-07	1.5E-05	#N/A	#N/A	#N/A	#N/A	#N/A	#N/A	#N/A	#N/A
TRINITY_DN21644_c0_g2_i19	SSG1B_HORVU	1.60	1.27	5.9E-04	2.0E-02	#N/A	#N/A	#N/A	#N/A	#N/A	#N/A	#N/A	#N/A
TRINITY_DN14992_c0_g1_i7	BGL08_ORYSJ	1.61	0.99	2.0E-03	5.3E-02	#N/A	#N/A	#N/A	#N/A	#N/A	#N/A	#N/A	#N/A
TRINITY_DN17850_c0_g1_i2	DTX36_ARATH	1.61	1.56	8.7E-05	4.1E-03	#N/A	#N/A	#N/A	#N/A	#N/A	#N/A	#N/A	#N/A
TRINITY_DN22443_c0_g3_i2	Y1684_ARATH	1.62	2.17	8.9E-05	4.2E-03	#N/A	#N/A	#N/A	#N/A	#N/A	#N/A	#N/A	#N/A
TRINITY_DN18360_c1_g1_i2	ARFA_ORYSJ	1.62	1.74	1.4E-03	4.1E-02	#N/A	#N/A	#N/A	#N/A	#N/A	#N/A	#N/A	#N/A
TRINITY_DN19270_c1_g2_i5	GSTUH_ARATH	1.63	1.77	2.4E-04	9.9E-03	#N/A	#N/A	#N/A	#N/A	#N/A	#N/A	#N/A	#N/A
TRINITY_DN20521_c0_g1_i12	PUM5_ARATH	1.63	2.16	1.4E-05	8.0E-04	#N/A	#N/A	#N/A	#N/A	#N/A	#N/A	#N/A	#N/A
TRINITY_DN22194_c1_g3_i14	CNGC2_ARATH	1.63	3.89	3.6E-05	1.9E-03	#N/A	#N/A	#N/A	#N/A	#N/A	#N/A	#N/A	#N/A
TRINITY_DN22705_c1_g5_i2	ALDO2_MAIZE	1.64	3.23	2.0E-05	1.1E-03	#N/A	#N/A	#N/A	#N/A	#N/A	#N/A	#N/A	#N/A
TRINITY_DN15213_c1_g1_i7	CGT_FRAAN	1.65	6.99	6.0E-08	6.0E-06	#N/A	#N/A	#N/A	#N/A	#N/A	#N/A	#N/A	#N/A
TRINITY_DN22760_c1_g1_i2	VIP2_NICBE	1.65	1.29	1.2E-03	3.7E-02	#N/A	#N/A	#N/A	#N/A	#N/A	#N/A	#N/A	#N/A
TRINITY_DN19647_c1_g2_i5	APT1_WHEAT	1.66	1.41	4.4E-04	1.6E-02	#N/A	#N/A	#N/A	#N/A	#N/A	#N/A	#N/A	#N/A
TRINITY_DN22705_c0_g2_i1	ALDO2_MAIZE	1.67	1.08	3.7E-04	1.4E-02	#N/A	#N/A	#N/A	#N/A	#N/A	#N/A	#N/A	#N/A
TRINITY_DN22086_c0_g2_i5	VP35B_ARATH	1.67	2.00	4.5E-05	2.3E-03	#N/A	#N/A	#N/A	#N/A	#N/A	#N/A	#N/A	#N/A
TRINITY_DN16583_c0_g3_i5	GATP3_ORYSI	1.69	1.59	9.4E-04	2.9E-02	#N/A	#N/A	#N/A	#N/A	#N/A	#N/A	#N/A	#N/A
TRINITY_DN21224_c0_g1_i2	M3K1_ARATH	1.69	2.90	4.9E-04	1.8E-02	#N/A	#N/A	#N/A	#N/A	#N/A	#N/A	#N/A	#N/A

DET	Subject id	c9TR vs c7NT				c7TR vs c7NT				c7TR vs c9TR			
		Log_2	Log_2CPM	PValue	19FDR	Log_2	Log_2CPM	PValue	19FDR	Log_2	Log_2CPM	PValue	19FDR
TRINITY_DN17917_c1_g1_i4	IDH1_ARATH	1.70	0.29	1.6E-03	4.5E-02	#N/A	#N/A	#N/A	#N/A	#N/A	#N/A	#N/A	#N/A
TRINITY_DN11113_c0_g2_i2	AGCT1_HORVU	1.70	2.41	2.4E-04	9.8E-03	#N/A	#N/A	#N/A	#N/A	#N/A	#N/A	#N/A	#N/A
TRINITY_DN13949_c0_g1_i1	C71Z6_ORYSJ	1.70	0.90	1.1E-03	3.3E-02	#N/A	#N/A	#N/A	#N/A	#N/A	#N/A	#N/A	#N/A
TRINITY_DN19407_c0_g4_i1	ERF1Z_ARATH	1.70	0.71	1.0E-03	3.1E-02	#N/A	#N/A	#N/A	#N/A	#N/A	#N/A	#N/A	#N/A
TRINITY_DN40487_c0_g1_i1	SD25_ARATH	1.71	0.46	8.8E-04	2.8E-02	#N/A	#N/A	#N/A	#N/A	#N/A	#N/A	#N/A	#N/A
TRINITY_DN22335_c1_g1_i10	KAT2_ORYSJ	1.71	0.65	1.7E-03	4.7E-02	#N/A	#N/A	#N/A	#N/A	#N/A	#N/A	#N/A	#N/A
TRINITY_DN15926_c0_g1_i2	ALDO2_MAIZE	1.71	4.04	1.7E-06	1.2E-04	#N/A	#N/A	#N/A	#N/A	#N/A	#N/A	#N/A	#N/A
TRINITY_DN16385_c0_g4_i7	COR2_PAPSO	1.72	3.70	1.3E-04	5.8E-03	#N/A	#N/A	#N/A	#N/A	#N/A	#N/A	#N/A	#N/A
TRINITY_DN18282_c1_g6_i1	ALDO2_MAIZE	1.72	6.10	2.6E-07	2.3E-05	#N/A	#N/A	#N/A	#N/A	#N/A	#N/A	#N/A	#N/A
TRINITY_DN14494_c0_g1_i12	Y5573_ARATH	1.72	4.82	5.5E-05	2.8E-03	#N/A	#N/A	#N/A	#N/A	#N/A	#N/A	#N/A	#N/A
TRINITY_DN19392_c0_g1_i2	COX1_MAIZE	1.73	1.62	6.2E-04	2.1E-02	#N/A	#N/A	#N/A	#N/A	#N/A	#N/A	#N/A	#N/A
TRINITY_DN16022_c0_g1_i3	CIPKV_ORYSJ	1.73	0.96	2.8E-04	1.1E-02	#N/A	#N/A	#N/A	#N/A	#N/A	#N/A	#N/A	#N/A
TRINITY_DN18541_c0_g1_i36	AB2C_ARATH	1.73	2.03	6.8E-04	2.3E-02	#N/A	#N/A	#N/A	#N/A	#N/A	#N/A	#N/A	#N/A
TRINITY_DN17308_c0_g1_i1	BCAT3_ARATH	1.74	2.18	6.7E-04	2.2E-02	#N/A	#N/A	#N/A	#N/A	#N/A	#N/A	#N/A	#N/A
TRINITY_DN22705_c1_g6_i3	ALDO2_MAIZE	1.74	5.32	3.9E-07	3.2E-05	#N/A	#N/A	#N/A	#N/A	#N/A	#N/A	#N/A	#N/A
TRINITY_DN18673_c0_g1_i3	LOXC2_ORYSJ	1.75	1.86	1.9E-03	5.1E-02	#N/A	#N/A	#N/A	#N/A	#N/A	#N/A	#N/A	#N/A
TRINITY_DN16232_c1_g2_i9	ODBA2_ARATH	1.77	6.09	3.4E-07	2.9E-05	#N/A	#N/A	#N/A	#N/A	#N/A	#N/A	#N/A	#N/A
TRINITY_DN18539_c0_g1_i13	AAH_ORYSJ	1.78	2.95	2.8E-08	2.9E-06	#N/A	#N/A	#N/A	#N/A	#N/A	#N/A	#N/A	#N/A
TRINITY_DN18556_c2_g2_i2	UBP5_ARATH	1.80	2.17	8.9E-04	2.8E-02	#N/A	#N/A	#N/A	#N/A	#N/A	#N/A	#N/A	#N/A
TRINITY_DN19707_c2_g1_i30	HPSE2_ARATH	1.80	1.33	1.7E-03	4.6E-02	#N/A	#N/A	#N/A	#N/A	#N/A	#N/A	#N/A	#N/A
TRINITY_DN19496_c0_g2_i22	PEX14_ARATH	1.81	1.03	9.8E-04	3.0E-02	#N/A	#N/A	#N/A	#N/A	#N/A	#N/A	#N/A	#N/A
TRINITY_DN20499_c0_g1_i4	RS4_ORYSJ	1.81	1.34	5.4E-04	1.9E-02	#N/A	#N/A	#N/A	#N/A	#N/A	#N/A	#N/A	#N/A
TRINITY_DN18500_c2_g3_i3	MAD26_ORYSJ	1.81	0.71	5.3E-04	1.9E-02	#N/A	#N/A	#N/A	#N/A	#N/A	#N/A	#N/A	#N/A
TRINITY_DN18515_c2_g2_i2	LORF2_MOUSE	1.82	1.61	1.9E-04	7.9E-03	#N/A	#N/A	#N/A	#N/A	#N/A	#N/A	#N/A	#N/A
TRINITY_DN20948_c0_g1_i7	LHT1_ARATH	1.83	0.53	5.6E-04	1.9E-02	#N/A	#N/A	#N/A	#N/A	#N/A	#N/A	#N/A	#N/A
TRINITY_DN19322_c0_g3_i1	MAD14_ORYSJ	1.84	4.28	1.7E-05	9.9E-04	#N/A	#N/A	#N/A	#N/A	#N/A	#N/A	#N/A	#N/A
TRINITY_DN21237_c1_g4_i4	GLR29_ARATH	1.84	0.33	1.8E-03	4.9E-02	#N/A	#N/A	#N/A	#N/A	#N/A	#N/A	#N/A	#N/A
TRINITY_DN14592_c0_g4_i5	WAK3_ARATH	1.84	2.05	2.7E-04	1.1E-02	#N/A	#N/A	#N/A	#N/A	#N/A	#N/A	#N/A	#N/A
TRINITY_DN14972_c0_g2_i7	U83A1_ARATH	1.85	2.28	7.0E-06	4.4E-04	#N/A	#N/A	#N/A	#N/A	#N/A	#N/A	#N/A	#N/A
TRINITY_DN18130_c0_g1_i12	ALLN_ALLCG	1.85	3.12	9.5E-09	1.1E-06	#N/A	#N/A	#N/A	#N/A	#N/A	#N/A	#N/A	#N/A
TRINITY_DN18130_c0_g1_i5	ALLN_ALLCE	1.85	4.61	1.6E-07	1.5E-05	#N/A	#N/A	#N/A	#N/A	#N/A	#N/A	#N/A	#N/A
TRINITY_DN17659_c0_g1_i3	APT2_ARATH	1.85	3.22	4.1E-04	1.5E-02	#N/A	#N/A	#N/A	#N/A	#N/A	#N/A	#N/A	#N/A
TRINITY_DN16093_c1_g1_i4	PYRG_DICDI	1.86	1.84	9.3E-06	5.7E-04	#N/A	#N/A	#N/A	#N/A	#N/A	#N/A	#N/A	#N/A
TRINITY_DN15926_c0_g1_i9	ALDO1_MAIZE	1.88	2.14	3.0E-04	1.2E-02	#N/A	#N/A	#N/A	#N/A	#N/A	#N/A	#N/A	#N/A
TRINITY_DN19177_c0_g1_i11	THIC1_ARATH	1.89	2.01	6.0E-04	2.1E-02	#N/A	#N/A	#N/A	#N/A	#N/A	#N/A	#N/A	#N/A
TRINITY_DN20155_c0_g2_i1	UMPS1_ORYSJ	1.90	0.41	5.2E-04	1.8E-02	#N/A	#N/A	#N/A	#N/A	#N/A	#N/A	#N/A	#N/A
TRINITY_DN22621_c3_g6_i2	POLX_TOBAC	1.90	1.50	3.7E-04	1.4E-02	#N/A	#N/A	#N/A	#N/A	#N/A	#N/A	#N/A	#N/A
TRINITY_DN22324_c0_g1_i3	OHK3_ORYSI	1.92	2.42	7.3E-05	3.5E-03	#N/A	#N/A	#N/A	#N/A	#N/A	#N/A	#N/A	#N/A
TRINITY_DN15899_c0_g2_i3	C81E1_GLYEC	1.92	2.99	4.5E-08	4.5E-06	#N/A	#N/A	#N/A	#N/A	#N/A	#N/A	#N/A	#N/A
TRINITY_DN18112_c1_g1_i6	NUD18_ARATH	1.93	1.08	1.1E-03	3.4E-02	#N/A	#N/A	#N/A	#N/A	#N/A	#N/A	#N/A	#N/A
TRINITY_DN17258_c0_g4_i10	GLR35_ARATH	1.93	2.49	1.2E-03	3.5E-02	#N/A	#N/A	#N/A	#N/A	#N/A	#N/A	#N/A	#N/A
TRINITY_DN17308_c0_g1_i2	BCAT3_ARATH	1.94	4.24	1.5E-06	1.1E-04	#N/A	#N/A	#N/A	#N/A	#N/A	#N/A	#N/A	#N/A
TRINITY_DN20758_c0_g1_i5	SFH8_ARATH	1.97	1.50	5.7E-05	2.9E-03	#N/A	#N/A	#N/A	#N/A	#N/A	#N/A	#N/A	#N/A
TRINITY_DN20536_c0_g1_i5	SOL1_ARATH	1.98	0.91	1.2E-03	3.5E-02	#N/A	#N/A	#N/A	#N/A	#N/A	#N/A	#N/A	#N/A
TRINITY_DN21413_c1_g3_i2	DTX27_ARATH	1.98	5.79	2.0E-03	5.3E-02	#N/A	#N/A	#N/A	#N/A	#N/A	#N/A	#N/A	#N/A
TRINITY_DN16990_c0_g2_i2	CYSP4_BRANA	1.98	0.35	2.2E-04	9.0E-03	#N/A	#N/A	#N/A	#N/A	#N/A	#N/A	#N/A	#N/A
TRINITY_DN18787_c0_g1_i1	PRNL1_ARATH	1.98	0.53	1.6E-03	4.5E-02	#N/A	#N/A	#N/A	#N/A	#N/A	#N/A	#N/A	#N/A
TRINITY_DN20527_c0_g2_i9	AB3C_ARATH	1.99	2.79	2.4E-06	1.6E-04	#N/A	#N/A	#N/A	#N/A	#N/A	#N/A	#N/A	#N/A
TRINITY_DN17035_c0_g3_i3	PTR14_ARATH	2.01	1.79	1.8E-03	4.8E-02	#N/A	#N/A	#N/A	#N/A	#N/A	#N/A	#N/A	#N/A
TRINITY_DN16828_c0_g2_i13	TKPR1_ARATH	2.02	5.77	1.2E-09	1.5E-07	#N/A	#N/A	#N/A	#N/A	#N/A	#N/A	#N/A	#N/A
TRINITY_DN20713_c0_g2_i16	RPN2_ORYSJ	2.02	0.62	1.4E-03	4.1E-02	#N/A	#N/A	#N/A	#N/A	#N/A	#N/A	#N/A	#N/A
TRINITY_DN17692_c2_g1_i5	OSB1_ARATH	2.03	2.16	1.6E-04	7.1E-03	#N/A	#N/A	#N/A	#N/A	#N/A	#N/A	#N/A	#N/A
TRINITY_DN14425_c0_g1_i1	UBA5_ORYSJ	2.05	0.55	7.3E-05	3.5E-03	#N/A	#N/A	#N/A	#N/A	#N/A	#N/A	#N/A	#N/A
TRINITY_DN14485_c1_g8_i2	GSTX4_TOBAC	2.07	3.86	7.7E-04	2.5E-02	#N/A	#N/A	#N/A	#N/A	#N/A	#N/A	#N/A	#N/A
TRINITY_DN21945_c0_g4_i6	Y3565_ARATH	2.07	2.16	4.5E-04	1.6E-02	#N/A	#N/A	#N/A	#N/A	#N/A	#N/A	#N/A	#N/A
TRINITY_DN14609_c0_g1_i5	C81E9_MEDTR	2.08	3.77	6.2E-04	2.1E-02	#N/A	#N/A	#N/A	#N/A	#N/A	#N/A	#N/A	#N/A
TRINITY_DN20764_c0_g1_i2	WEB1_ARATH	2.08	0.45	3.7E-04	1.4E-02	#N/A	#N/A	#N/A	#N/A	#N/A	#N/A	#N/A	#N/A
TRINITY_DN17607_c0_g2_i1	CRSP_ARATH	2.08	2.66	1.4E-03	3.9E-02	#N/A	#N/A	#N/A	#N/A	#N/A	#N/A	#N/A	#N/A
TRINITY_DN14208_c0_g1_i8	ZIP4_ARATH	2.08	1.68	8.3E-06	5.1E-04	#N/A	#N/A	#N/A	#N/A	#N/A	#N/A	#N/A	#N/A
TRINITY_DN22449_c1_g1_i5	PMAT2_ARATH	2.09	3.00	3.3E-04	1.3E-02	#N/A	#N/A	#N/A	#N/A	#N/A	#N/A	#N/A	#N/A
TRINITY_DN21355_c1_g2_i4	ITPK3_ORYSJ	2.09	1.57	1.9E-06	1.4E-04	#N/A	#N/A	#N/A	#N/A	#N/A	#N/A	#N/A	#N/A

DET	Subject id	c9TR vs c7NT				c7TR vs c7NT				c7TR vs c9TR			
		Log_2	Log_2CPM	PValue	19FDR	Log_2	Log_2CPM	PValue	19FDR	Log_2	Log_2CPM	PValue	19FDR
TRINITY_DN20586_c0_g2_i13	NIR_MAIZE	2.13	2.27	4.1E-08	4.1E-06	#N/A	#N/A	#N/A	#N/A	#N/A	#N/A	#N/A	#N/A
TRINITY_DN22340_c1_g1_i12	OML4_ORYSJ	2.16	2.48	5.5E-09	6.4E-07	#N/A	#N/A	#N/A	#N/A	#N/A	#N/A	#N/A	#N/A
TRINITY_DN19270_c1_g2_i1	GSTUH_ARATH	2.16	0.49	2.5E-04	1.0E-02	#N/A	#N/A	#N/A	#N/A	#N/A	#N/A	#N/A	#N/A
TRINITY_DN19537_c1_g7_i7	VILI4_ARATH	2.16	0.39	1.2E-03	3.7E-02	#N/A	#N/A	#N/A	#N/A	#N/A	#N/A	#N/A	#N/A
TRINITY_DN18470_c1_g1_i2	MTP1_ORYSJ	2.17	3.04	3.2E-05	1.7E-03	#N/A	#N/A	#N/A	#N/A	#N/A	#N/A	#N/A	#N/A
TRINITY_DN22379_c0_g1_i17	GH35_ORYSJ	2.18	1.25	9.7E-04	3.0E-02	#N/A	#N/A	#N/A	#N/A	#N/A	#N/A	#N/A	#N/A
TRINITY_DN20555_c0_g2_i4	E134_MAIZE	2.20	2.48	7.2E-07	5.6E-05	#N/A	#N/A	#N/A	#N/A	#N/A	#N/A	#N/A	#N/A
TRINITY_DN14790_c0_g1_i3	STAD7_ORYSJ	2.20	1.02	9.2E-05	4.3E-03	#N/A	#N/A	#N/A	#N/A	#N/A	#N/A	#N/A	#N/A
TRINITY_DN18472_c0_g2_i9	SRF6_ARATH	2.20	1.98	3.9E-05	2.1E-03	#N/A	#N/A	#N/A	#N/A	#N/A	#N/A	#N/A	#N/A
TRINITY_DN20493_c0_g3_i2	AGAL1_ARATH	2.21	1.02	2.7E-05	1.5E-03	#N/A	#N/A	#N/A	#N/A	#N/A	#N/A	#N/A	#N/A
TRINITY_DN22387_c1_g2_i14	LGUL_ORYSJ	2.21	0.49	1.2E-04	5.3E-03	#N/A	#N/A	#N/A	#N/A	#N/A	#N/A	#N/A	#N/A
TRINITY_DN22280_c1_g2_i13	DNJH2_ALLPO	2.23	0.75	8.0E-04	2.6E-02	#N/A	#N/A	#N/A	#N/A	#N/A	#N/A	#N/A	#N/A
TRINITY_DN17408_c0_g1_i3	ZERSY_ZINZE	2.24	0.69	2.0E-03	5.2E-02	#N/A	#N/A	#N/A	#N/A	#N/A	#N/A	#N/A	#N/A
TRINITY_DN15308_c0_g1_i1	DBNBT_TAXCA	2.24	1.17	1.5E-03	4.2E-02	#N/A	#N/A	#N/A	#N/A	#N/A	#N/A	#N/A	#N/A
TRINITY_DN22030_c3_g1_i14	RFC3_ARATH	2.27	2.37	9.9E-07	7.6E-05	#N/A	#N/A	#N/A	#N/A	#N/A	#N/A	#N/A	#N/A
TRINITY_DN17984_c0_g1_i3	NAC67_ORYSJ	2.27	1.06	1.9E-05	1.1E-03	#N/A	#N/A	#N/A	#N/A	#N/A	#N/A	#N/A	#N/A
TRINITY_DN18653_c0_g1_i3	ADCS_ORYSJ	2.29	1.08	2.3E-05	1.3E-03	#N/A	#N/A	#N/A	#N/A	#N/A	#N/A	#N/A	#N/A
TRINITY_DN17340_c1_g3_i2	E1313_ARATH	2.30	1.94	1.3E-05	7.8E-04	#N/A	#N/A	#N/A	#N/A	#N/A	#N/A	#N/A	#N/A
TRINITY_DN20777_c0_g2_i14	YBEY_SYNPX	2.32	1.58	1.3E-06	9.6E-05	#N/A	#N/A	#N/A	#N/A	#N/A	#N/A	#N/A	#N/A
TRINITY_DN16735_c0_g1_i8	GSH1B_ORYSJ	2.33	1.92	1.7E-04	7.4E-03	#N/A	#N/A	#N/A	#N/A	#N/A	#N/A	#N/A	#N/A
TRINITY_DN16858_c0_g1_i10	PP127_ARATH	2.35	3.13	4.2E-08	4.2E-06	#N/A	#N/A	#N/A	#N/A	#N/A	#N/A	#N/A	#N/A
TRINITY_DN17314_c0_g2_i1	MDAR2_ARATH	2.35	0.34	9.6E-04	3.0E-02	#N/A	#N/A	#N/A	#N/A	#N/A	#N/A	#N/A	#N/A
TRINITY_DN19372_c1_g2_i6	ZPR1_MOUSE	2.36	1.21	1.4E-03	4.0E-02	#N/A	#N/A	#N/A	#N/A	#N/A	#N/A	#N/A	#N/A
TRINITY_DN14494_c0_g1_i6	Y2921_ARATH	2.38	1.79	1.2E-05	7.4E-04	#N/A	#N/A	#N/A	#N/A	#N/A	#N/A	#N/A	#N/A
TRINITY_DN15681_c2_g3_i5	NADO2_ORYSJ	2.38	0.18	1.3E-04	5.7E-03	#N/A	#N/A	#N/A	#N/A	#N/A	#N/A	#N/A	#N/A
TRINITY_DN19835_c0_g1_i3	APC4_ARATH	2.38	-0.12	5.1E-04	1.8E-02	#N/A	#N/A	#N/A	#N/A	#N/A	#N/A	#N/A	#N/A
TRINITY_DN17766_c1_g1_i6	WTR45_ARATH	2.44	0.28	4.6E-05	2.3E-03	#N/A	#N/A	#N/A	#N/A	#N/A	#N/A	#N/A	#N/A
TRINITY_DN18888_c0_g2_i19	ARSB_DICDI	2.44	5.18	1.2E-04	5.4E-03	#N/A	#N/A	#N/A	#N/A	#N/A	#N/A	#N/A	#N/A
TRINITY_DN21355_c1_g2_i7	ITPK3_ORYSJ	2.45	0.06	2.5E-04	1.0E-02	#N/A	#N/A	#N/A	#N/A	#N/A	#N/A	#N/A	#N/A
TRINITY_DN19937_c3_g1_i1	THIC_ARATH	2.48	5.61	1.2E-15	3.2E-13	#N/A	#N/A	#N/A	#N/A	#N/A	#N/A	#N/A	#N/A
TRINITY_DN21413_c1_g3_i3	DTX27_ARATH	2.49	1.30	6.0E-08	5.9E-06	#N/A	#N/A	#N/A	#N/A	#N/A	#N/A	#N/A	#N/A
TRINITY_DN16232_c1_g2_i16	ODBA2_ARATH	2.50	3.34	3.3E-10	4.5E-08	#N/A	#N/A	#N/A	#N/A	#N/A	#N/A	#N/A	#N/A
TRINITY_DN17318_c0_g1_i14	KMS1_ARATH	2.50	3.35	1.3E-09	1.6E-07	#N/A	#N/A	#N/A	#N/A	#N/A	#N/A	#N/A	#N/A
TRINITY_DN15451_c0_g1_i6	SCP18_ARATH	2.51	2.40	3.0E-08	3.1E-06	#N/A	#N/A	#N/A	#N/A	#N/A	#N/A	#N/A	#N/A
TRINITY_DN19560_c2_g1_i4	LIN1_NYCCO	2.54	0.87	2.1E-06	1.5E-04	#N/A	#N/A	#N/A	#N/A	#N/A	#N/A	#N/A	#N/A
TRINITY_DN18362_c0_g1_i2	NAATA_HORVU	2.55	1.75	5.2E-04	1.8E-02	#N/A	#N/A	#N/A	#N/A	#N/A	#N/A	#N/A	#N/A
TRINITY_DN18748_c0_g4_i3	RH27_ORYSJ	2.56	3.20	2.3E-07	2.0E-05	#N/A	#N/A	#N/A	#N/A	#N/A	#N/A	#N/A	#N/A
TRINITY_DN16232_c1_g2_i17	ODBA2_ARATH	2.58	0.63	2.9E-05	1.6E-03	#N/A	#N/A	#N/A	#N/A	#N/A	#N/A	#N/A	#N/A
TRINITY_DN16609_c0_g1_i2	YC20L_ARATH	2.59	2.87	8.3E-15	1.9E-12	#N/A	#N/A	#N/A	#N/A	#N/A	#N/A	#N/A	#N/A
TRINITY_DN18362_c0_g1_i11	NAATA_HORVU	2.60	5.24	1.3E-13	2.6E-11	#N/A	#N/A	#N/A	#N/A	#N/A	#N/A	#N/A	#N/A
TRINITY_DN19602_c0_g2_i15	GGP5_ARATH	2.61	3.98	2.7E-04	1.1E-02	#N/A	#N/A	#N/A	#N/A	#N/A	#N/A	#N/A	#N/A
TRINITY_DN14485_c1_g8_i1	GSTX4_TOBAC	2.63	-0.11	1.3E-04	5.7E-03	#N/A	#N/A	#N/A	#N/A	#N/A	#N/A	#N/A	#N/A
TRINITY_DN14494_c0_g1_i10	Y5188_ARATH	2.64	1.03	1.4E-05	8.3E-04	#N/A	#N/A	#N/A	#N/A	#N/A	#N/A	#N/A	#N/A
TRINITY_DN20847_c0_g2_i8	FPP6_ARATH	2.64	0.89	9.8E-04	3.0E-02	#N/A	#N/A	#N/A	#N/A	#N/A	#N/A	#N/A	#N/A
TRINITY_DN14398_c0_g1_i1	SAG39_ORYSJ	2.66	0.40	9.6E-04	3.0E-02	#N/A	#N/A	#N/A	#N/A	#N/A	#N/A	#N/A	#N/A
TRINITY_DN20275_c1_g6_i1	U73D1_ARATH	2.69	4.08	1.5E-03	4.2E-02	#N/A	#N/A	#N/A	#N/A	#N/A	#N/A	#N/A	#N/A
TRINITY_DN18362_c0_g1_i5	NAATA_HORVU	2.71	3.18	3.2E-05	1.7E-03	#N/A	#N/A	#N/A	#N/A	#N/A	#N/A	#N/A	#N/A
TRINITY_DN19102_c0_g1_i2	BOR2_ARATH	2.73	2.47	3.1E-04	1.2E-02	#N/A	#N/A	#N/A	#N/A	#N/A	#N/A	#N/A	#N/A
TRINITY_DN21064_c0_g1_i5	RH1_ORYSJ	2.74	-0.48	7.3E-04	2.4E-02	#N/A	#N/A	#N/A	#N/A	#N/A	#N/A	#N/A	#N/A
TRINITY_DN15735_c0_g1_i7	STT7_ARATH	2.76	1.33	2.8E-04	1.1E-02	#N/A	#N/A	#N/A	#N/A	#N/A	#N/A	#N/A	#N/A
TRINITY_DN22449_c1_g1_i8	ANTA_GENTR	2.80	1.81	5.9E-05	2.9E-03	#N/A	#N/A	#N/A	#N/A	#N/A	#N/A	#N/A	#N/A
TRINITY_DN17659_c0_g1_i2	APT2_ARATH	2.81	2.15	6.6E-06	4.1E-04	#N/A	#N/A	#N/A	#N/A	#N/A	#N/A	#N/A	#N/A
TRINITY_DN18123_c1_g1_i3	HEL2_SCHPO	2.86	1.77	8.6E-06	5.3E-04	#N/A	#N/A	#N/A	#N/A	#N/A	#N/A	#N/A	#N/A
TRINITY_DN21413_c1_g1_i2	DTX27_ARATH	2.87	1.27	1.4E-04	6.0E-03	#N/A	#N/A	#N/A	#N/A	#N/A	#N/A	#N/A	#N/A
TRINITY_DN18330_c0_g1_i1	NAA15_PONAB	2.90	-0.34	6.0E-04	2.1E-02	#N/A	#N/A	#N/A	#N/A	#N/A	#N/A	#N/A	#N/A
TRINITY_DN22590_c1_g1_i34	FTSH9_ORYSJ	2.92	1.33	1.8E-07	1.7E-05	#N/A	#N/A	#N/A	#N/A	#N/A	#N/A	#N/A	#N/A
TRINITY_DN18362_c0_g1_i9	NAATA_HORVU	2.92	5.74	4.0E-06	2.6E-04	#N/A	#N/A	#N/A	#N/A	#N/A	#N/A	#N/A	#N/A
TRINITY_DN18472_c0_g2_i8	SRF6_ARATH	2.96	-0.04	9.6E-05	4.5E-03	#N/A	#N/A	#N/A	#N/A	#N/A	#N/A	#N/A	#N/A
TRINITY_DN17679_c1_g4_i5	FBK28_ARATH	2.98	2.59	5.3E-06	3.4E-04	#N/A	#N/A	#N/A	#N/A	#N/A	#N/A	#N/A	#N/A
TRINITY_DN17659_c0_g1_i13	APT2_ARATH	3.01	0.48	6.5E-04	2.2E-02	#N/A	#N/A	#N/A	#N/A	#N/A	#N/A	#N/A	#N/A
TRINITY_DN15470_c0_g1_i13	PAP6_ARATH	3.03	4.98	8.0E-07	6.2E-05	#N/A	#N/A	#N/A	#N/A	#N/A	#N/A	#N/A	#N/A

DET	Subject id	c9TR vs c7NT				c7TR vs c7NT				c7TR vs c9TR			
		Log_2	Log_2CPM	PValue	19FDR	Log_2	Log_2CPM	PValue	19FDR	Log_2	Log_2CPM	PValue	19FDR
TRINITY_DN21794_c0_g9_i3	PIP24_MAIZE	3.03	0.01	4.6E-04	1.7E-02	#N/A	#N/A	#N/A	#N/A	#N/A	#N/A	#N/A	#N/A
TRINITY_DN19733_c0_g3_i2	BAM1_ARATH	3.05	1.62	4.4E-10	5.9E-08	#N/A	#N/A	#N/A	#N/A	#N/A	#N/A	#N/A	#N/A
TRINITY_DN21355_c1_g2_i18	ITPK3_ORYSJ	3.06	1.45	8.0E-09	9.0E-07	#N/A	#N/A	#N/A	#N/A	#N/A	#N/A	#N/A	#N/A
TRINITY_DN15158_c1_g1_i10	ADT4_ARATH	3.15	3.07	8.4E-14	1.8E-11	#N/A	#N/A	#N/A	#N/A	#N/A	#N/A	#N/A	#N/A
TRINITY_DN21309_c1_g1_i17	SMG8_BOVIN	3.19	2.46	1.2E-03	3.5E-02	#N/A	#N/A	#N/A	#N/A	#N/A	#N/A	#N/A	#N/A
TRINITY_DN21166_c0_g8_i2	TTL1_ARATH	3.21	0.17	7.9E-05	3.8E-03	#N/A	#N/A	#N/A	#N/A	#N/A	#N/A	#N/A	#N/A
TRINITY_DN16529_c1_g1_i5	ETFA_ORYSJ	3.23	-0.27	6.4E-04	2.2E-02	#N/A	#N/A	#N/A	#N/A	#N/A	#N/A	#N/A	#N/A
TRINITY_DN14853_c0_g1_i3	SERB1_ARATH	3.24	-0.25	1.5E-03	4.3E-02	#N/A	#N/A	#N/A	#N/A	#N/A	#N/A	#N/A	#N/A
TRINITY_DN18500_c2_g1_i4	MAD26_ORYSJ	3.25	-0.27	3.1E-05	1.7E-03	#N/A	#N/A	#N/A	#N/A	#N/A	#N/A	#N/A	#N/A
TRINITY_DN17345_c0_g1_i6	SALR_PAPBR	3.29	1.67	8.9E-05	4.2E-03	#N/A	#N/A	#N/A	#N/A	#N/A	#N/A	#N/A	#N/A
TRINITY_DN17766_c1_g1_i1	WTR45_ARATH	3.32	-0.41	1.3E-03	3.9E-02	#N/A	#N/A	#N/A	#N/A	#N/A	#N/A	#N/A	#N/A
TRINITY_DN16242_c0_g1_i15	IN21_MAIZE	3.33	2.00	3.1E-06	2.1E-04	#N/A	#N/A	#N/A	#N/A	#N/A	#N/A	#N/A	#N/A
TRINITY_DN15262_c0_g1_i10	BAT1_ORYSJ	3.35	0.42	3.9E-04	1.5E-02	#N/A	#N/A	#N/A	#N/A	#N/A	#N/A	#N/A	#N/A
TRINITY_DN21298_c0_g1_i2	ODP22_ARATH	3.43	1.14	1.0E-03	3.2E-02	#N/A	#N/A	#N/A	#N/A	#N/A	#N/A	#N/A	#N/A
TRINITY_DN21060_c1_g2_i5	CAP10_ARATH	3.43	-0.01	5.1E-07	4.1E-05	#N/A	#N/A	#N/A	#N/A	#N/A	#N/A	#N/A	#N/A
TRINITY_DN20041_c0_g1_i10	ALMT9_ARATH	3.52	0.19	1.7E-03	4.6E-02	#N/A	#N/A	#N/A	#N/A	#N/A	#N/A	#N/A	#N/A
TRINITY_DN22270_c0_g1_i15	PIGB_BOVIN	3.56	0.48	1.7E-05	9.9E-04	#N/A	#N/A	#N/A	#N/A	#N/A	#N/A	#N/A	#N/A
TRINITY_DN18362_c0_g1_i8	NAATB_HORVU	3.58	2.94	7.4E-06	4.6E-04	#N/A	#N/A	#N/A	#N/A	#N/A	#N/A	#N/A	#N/A
TRINITY_DN20586_c0_g2_i10	NIR_MAIZE	3.59	0.48	2.1E-04	8.8E-03	#N/A	#N/A	#N/A	#N/A	#N/A	#N/A	#N/A	#N/A
TRINITY_DN21100_c0_g3_i2	HD3A_ORYSJ	3.61	1.46	1.8E-04	7.7E-03	#N/A	#N/A	#N/A	#N/A	#N/A	#N/A	#N/A	#N/A
TRINITY_DN16131_c2_g3_i4	SY131_ARATH	3.61	-0.38	1.4E-03	3.9E-02	#N/A	#N/A	#N/A	#N/A	#N/A	#N/A	#N/A	#N/A
TRINITY_DN22590_c1_g1_i12	FTSH9_ORYSJ	3.63	-0.24	6.3E-04	2.1E-02	#N/A	#N/A	#N/A	#N/A	#N/A	#N/A	#N/A	#N/A
TRINITY_DN22035_c0_g1_i3	SKI35_ARATH	3.63	1.03	5.1E-04	1.8E-02	#N/A	#N/A	#N/A	#N/A	#N/A	#N/A	#N/A	#N/A
TRINITY_DN19496_c0_g2_i24	PEX14_ARATH	3.79	0.35	6.0E-05	3.0E-03	#N/A	#N/A	#N/A	#N/A	#N/A	#N/A	#N/A	#N/A
TRINITY_DN22114_c0_g1_i13	TRH22_ORYSJ	3.82	2.23	1.1E-03	3.4E-02	#N/A	#N/A	#N/A	#N/A	#N/A	#N/A	#N/A	#N/A
TRINITY_DN20948_c0_g1_i9	LHT1_ARATH	3.85	0.13	1.6E-04	6.9E-03	#N/A	#N/A	#N/A	#N/A	#N/A	#N/A	#N/A	#N/A
TRINITY_DN15598_c0_g1_i10	RL38_ARATH	3.90	0.31	3.9E-05	2.0E-03	#N/A	#N/A	#N/A	#N/A	#N/A	#N/A	#N/A	#N/A
TRINITY_DN17766_c1_g1_i2	WTR45_ARATH	3.91	2.13	7.3E-04	2.4E-02	#N/A	#N/A	#N/A	#N/A	#N/A	#N/A	#N/A	#N/A
TRINITY_DN15903_c0_g1_i12	ACCO1_ORYSI	3.92	3.73	1.2E-06	8.8E-05	#N/A	#N/A	#N/A	#N/A	#N/A	#N/A	#N/A	#N/A
TRINITY_DN19503_c0_g1_i1	CKX2_ORYSJ	3.95	-0.59	1.7E-04	7.3E-03	#N/A	#N/A	#N/A	#N/A	#N/A	#N/A	#N/A	#N/A
TRINITY_DN18953_c0_g1_i2	DHAR3_ARATH	4.08	0.72	8.6E-06	5.3E-04	#N/A	#N/A	#N/A	#N/A	#N/A	#N/A	#N/A	#N/A
TRINITY_DN20214_c1_g2_i2	CPY28_ARATH	4.16	1.93	2.3E-04	9.5E-03	#N/A	#N/A	#N/A	#N/A	#N/A	#N/A	#N/A	#N/A
TRINITY_DN16274_c0_g1_i8	DPH6_SCHPO	4.23	1.26	1.8E-03	4.9E-02	#N/A	#N/A	#N/A	#N/A	#N/A	#N/A	#N/A	#N/A
TRINITY_DN16001_c0_g3_i1	PP347_ARATH	4.27	-0.11	1.6E-03	4.3E-02	#N/A	#N/A	#N/A	#N/A	#N/A	#N/A	#N/A	#N/A
TRINITY_DN19227_c1_g1_i1	NRPC1_ARATH	4.32	-0.57	1.5E-03	4.2E-02	#N/A	#N/A	#N/A	#N/A	#N/A	#N/A	#N/A	#N/A
TRINITY_DN16628_c0_g3_i14	PGLR_VITVI	4.45	0.72	3.4E-04	1.3E-02	#N/A	#N/A	#N/A	#N/A	#N/A	#N/A	#N/A	#N/A
TRINITY_DN16283_c0_g1_i25	CRLK2_ARATH	4.47	2.20	8.0E-07	6.2E-05	#N/A	#N/A	#N/A	#N/A	#N/A	#N/A	#N/A	#N/A
TRINITY_DN15514_c2_g5_i9	RH10_ORYSJ	4.48	0.34	1.5E-05	8.7E-04	#N/A	#N/A	#N/A	#N/A	#N/A	#N/A	#N/A	#N/A
TRINITY_DN22678_c1_g1_i8	CLASP_ARATH	4.55	0.59	9.8E-10	1.2E-07	#N/A	#N/A	#N/A	#N/A	#N/A	#N/A	#N/A	#N/A
TRINITY_DN18018_c0_g1_i18	QKIL2_ARATH	4.64	-0.45	6.3E-04	2.1E-02	#N/A	#N/A	#N/A	#N/A	#N/A	#N/A	#N/A	#N/A
TRINITY_DN13634_c0_g1_i1	U73D1_ARATH	4.70	0.05	1.4E-05	8.3E-04	#N/A	#N/A	#N/A	#N/A	#N/A	#N/A	#N/A	#N/A
TRINITY_DN12882_c0_g1_i3	E13B_MAIZE	4.74	0.84	4.5E-09	5.3E-07	#N/A	#N/A	#N/A	#N/A	#N/A	#N/A	#N/A	#N/A
TRINITY_DN20210_c2_g2_i15	CKX11_ORYSJ	4.75	0.31	1.0E-03	3.1E-02	#N/A	#N/A	#N/A	#N/A	#N/A	#N/A	#N/A	#N/A
TRINITY_DN21121_c1_g1_i8	SC11C_PONAB	4.81	0.14	3.1E-05	1.7E-03	#N/A	#N/A	#N/A	#N/A	#N/A	#N/A	#N/A	#N/A
TRINITY_DN21159_c0_g1_i6	SNF4_ARATH	4.84	-0.11	4.3E-04	1.6E-02	#N/A	#N/A	#N/A	#N/A	#N/A	#N/A	#N/A	#N/A
TRINITY_DN20256_c1_g1_i11	RRFC_ORYSJ	4.85	-0.28	2.4E-04	9.8E-03	#N/A	#N/A	#N/A	#N/A	#N/A	#N/A	#N/A	#N/A
TRINITY_DN17398_c1_g2_i4	RHD32_ORYSJ	4.86	-0.74	1.2E-03	3.7E-02	#N/A	#N/A	#N/A	#N/A	#N/A	#N/A	#N/A	#N/A
TRINITY_DN15344_c0_g2_i3	GT51_ORYSJ	5.28	0.09	3.1E-05	1.7E-03	#N/A	#N/A	#N/A	#N/A	#N/A	#N/A	#N/A	#N/A
TRINITY_DN19835_c0_g1_i20	APC4_ARATH	5.32	-0.48	7.3E-05	3.5E-03	#N/A	#N/A	#N/A	#N/A	#N/A	#N/A	#N/A	#N/A
TRINITY_DN14962_c0_g1_i19	ILR3_ARATH	5.50	2.56	4.6E-08	4.6E-06	#N/A	#N/A	#N/A	#N/A	#N/A	#N/A	#N/A	#N/A
TRINITY_DN18600_c1_g1_i5	PGMP_ARATH	5.89	0.58	3.1E-12	5.4E-10	#N/A	#N/A	#N/A	#N/A	#N/A	#N/A	#N/A	#N/A
TRINITY_DN20930_c0_g1_i5	MTP8_ORYSJ	5.91	-0.61	1.9E-06	1.3E-04	#N/A	#N/A	#N/A	#N/A	#N/A	#N/A	#N/A	#N/A
TRINITY_DN20694_c0_g1_i6	CESA8_ORYSJ	6.42	1.35	2.1E-03	5.4E-02	#N/A	#N/A	#N/A	#N/A	#N/A	#N/A	#N/A	#N/A
TRINITY_DN22149_c0_g1_i1	PGMC1_MAIZE	6.43	2.35	1.7E-06	1.2E-04	#N/A	#N/A	#N/A	#N/A	#N/A	#N/A	#N/A	#N/A
TRINITY_DN20044_c1_g1_i9	NPC1_HUMAN	6.58	-0.06	2.4E-06	1.7E-04	#N/A	#N/A	#N/A	#N/A	#N/A	#N/A	#N/A	#N/A
TRINITY_DN20533_c0_g1_i7	AB22G_ARATH	6.61	2.44	1.1E-08	1.2E-06	#N/A	#N/A	#N/A	#N/A	#N/A	#N/A	#N/A	#N/A
TRINITY_DN20009_c0_g2_i8	SCE1_ARATH	6.68	3.27	3.2E-23	1.6E-20	#N/A	#N/A	#N/A	#N/A	#N/A	#N/A	#N/A	#N/A
TRINITY_DN19762_c0_g1_i8	PTR53_ARATH	6.81	1.41	4.6E-17	1.3E-14	#N/A	#N/A	#N/A	#N/A	#N/A	#N/A	#N/A	#N/A
TRINITY_DN20927_c0_g1_i11	GT644_ARATH	7.12	2.81	2.0E-32	1.9E-29	#N/A	#N/A	#N/A	#N/A	#N/A	#N/A	#N/A	#N/A
TRINITY_DN20024_c4_g2_i5	CFA20_PARTE	7.48	-0.99	7.1E-04	2.3E-02	#N/A	#N/A	#N/A	#N/A	#N/A	#N/A	#N/A	#N/A
TRINITY_DN21964_c0_g1_i14	ILVD_ARATH	7.51	0.85	1.2E-09	1.5E-07	#N/A	#N/A	#N/A	#N/A	#N/A	#N/A	#N/A	#N/A

DET	Subject id	c9TR vs c7NT				c7TR vs c7NT				c7TR vs c9TR			
		Log_2	Log_2CPM	PValue	19FDR	Log_2	Log_2CPM	PValue	19FDR	Log_2	Log_2CPM	PValue	19FDR
TRINITY_DN16699_c2_g1_i3	DER_SYNR3	7.58	-0.91	2.4E-05	1.3E-03	#N/A	#N/A	#N/A	#N/A	#N/A	#N/A	#N/A	#N/A
TRINITY_DN18103_c0_g3_i5	FABG3_BRANA	7.58	2.16	1.1E-22	5.2E-20	#N/A	#N/A	#N/A	#N/A	#N/A	#N/A	#N/A	#N/A
TRINITY_DN18852_c0_g1_i23	Y5573_ARATH	7.69	-0.82	1.2E-06	8.7E-05	#N/A	#N/A	#N/A	#N/A	#N/A	#N/A	#N/A	#N/A
TRINITY_DN20559_c0_g1_i9	RL4A_ARATH	7.75	-0.76	1.3E-06	9.6E-05	#N/A	#N/A	#N/A	#N/A	#N/A	#N/A	#N/A	#N/A
TRINITY_DN16683_c0_g1_i5	THI42_MAIZE	7.76	-0.76	4.8E-07	3.9E-05	#N/A	#N/A	#N/A	#N/A	#N/A	#N/A	#N/A	#N/A
TRINITY_DN18348_c3_g3_i1	GAUT8_ARATH	7.76	-0.75	3.8E-07	3.2E-05	#N/A	#N/A	#N/A	#N/A	#N/A	#N/A	#N/A	#N/A
TRINITY_DN15434_c0_g1_i3	MENG_ARATH	7.81	-0.71	6.4E-06	4.0E-04	#N/A	#N/A	#N/A	#N/A	#N/A	#N/A	#N/A	#N/A
TRINITY_DN15333_c0_g5_i5	TAF6_ARATH	7.81	-0.72	8.6E-05	4.1E-03	#N/A	#N/A	#N/A	#N/A	#N/A	#N/A	#N/A	#N/A
TRINITY_DN22267_c0_g1_i2	RH21_ORYSJ	7.82	-0.70	5.3E-07	4.2E-05	#N/A	#N/A	#N/A	#N/A	#N/A	#N/A	#N/A	#N/A
TRINITY_DN19615_c0_g2_i11	DES2_SORBI	7.83	-0.69	1.9E-05	1.1E-03	#N/A	#N/A	#N/A	#N/A	#N/A	#N/A	#N/A	#N/A
TRINITY_DN16551_c0_g1_i11	LC7L3_PONAB	7.83	-0.69	3.5E-05	1.8E-03	#N/A	#N/A	#N/A	#N/A	#N/A	#N/A	#N/A	#N/A
TRINITY_DN15324_c0_g1_i1	BET11_ARATH	7.85	-0.68	8.3E-07	6.4E-05	#N/A	#N/A	#N/A	#N/A	#N/A	#N/A	#N/A	#N/A
TRINITY_DN20201_c8_g5_i1	LORF2_MOUSE	7.86	-0.66	3.7E-07	3.1E-05	#N/A	#N/A	#N/A	#N/A	#N/A	#N/A	#N/A	#N/A
TRINITY_DN16600_c0_g2_i18	SCRK1_MAIZE	7.89	-0.64	1.9E-06	1.3E-04	#N/A	#N/A	#N/A	#N/A	#N/A	#N/A	#N/A	#N/A
TRINITY_DN21121_c1_g1_i32	SC11C_PONAB	7.98	-0.56	3.9E-06	2.6E-04	#N/A	#N/A	#N/A	#N/A	#N/A	#N/A	#N/A	#N/A
TRINITY_DN15851_c2_g1_i1	AT13A_ARATH	7.98	-0.56	1.2E-06	9.3E-05	#N/A	#N/A	#N/A	#N/A	#N/A	#N/A	#N/A	#N/A
TRINITY_DN21554_c0_g1_i7	DWRF8_MAIZE	8.03	-0.52	3.0E-06	2.0E-04	#N/A	#N/A	#N/A	#N/A	#N/A	#N/A	#N/A	#N/A
TRINITY_DN20126_c0_g1_i19	COB21_ORYSJ	8.04	-0.50	3.2E-06	2.1E-04	#N/A	#N/A	#N/A	#N/A	#N/A	#N/A	#N/A	#N/A
TRINITY_DN15934_c3_g3_i4	IAA10_ORYSJ	8.04	-0.50	1.8E-03	4.9E-02	#N/A	#N/A	#N/A	#N/A	#N/A	#N/A	#N/A	#N/A
TRINITY_DN15450_c0_g1_i8	CSPLC_MAIZE	8.05	-0.50	1.7E-07	1.6E-05	#N/A	#N/A	#N/A	#N/A	#N/A	#N/A	#N/A	#N/A
TRINITY_DN17287_c1_g1_i14	CTF77_ARATH	8.06	-0.49	6.8E-05	3.3E-03	#N/A	#N/A	#N/A	#N/A	#N/A	#N/A	#N/A	#N/A
TRINITY_DN18102_c1_g1_i9	PUX10_ARATH	8.06	-0.49	2.4E-07	2.1E-05	#N/A	#N/A	#N/A	#N/A	#N/A	#N/A	#N/A	#N/A
TRINITY_DN19052_c0_g1_i1	Y3228_ARATH	8.07	-0.49	1.1E-06	8.3E-05	#N/A	#N/A	#N/A	#N/A	#N/A	#N/A	#N/A	#N/A
TRINITY_DN20993_c0_g1_i1	CARB_ORYSJ	8.08	-0.47	2.6E-07	2.3E-05	#N/A	#N/A	#N/A	#N/A	#N/A	#N/A	#N/A	#N/A
TRINITY_DN15838_c0_g1_i8	ATG10_ARATH	8.08	-0.47	9.6E-07	7.3E-05	#N/A	#N/A	#N/A	#N/A	#N/A	#N/A	#N/A	#N/A
TRINITY_DN21519_c0_g3_i15	C3H5_ORYSJ	8.09	-0.46	2.0E-06	1.4E-04	#N/A	#N/A	#N/A	#N/A	#N/A	#N/A	#N/A	#N/A
TRINITY_DN21040_c0_g1_i7	LUT1_ARATH	8.15	-0.41	4.5E-08	4.5E-06	#N/A	#N/A	#N/A	#N/A	#N/A	#N/A	#N/A	#N/A
TRINITY_DN13572_c0_g1_i2	UBP19_ARATH	8.17	-0.39	9.7E-08	9.4E-06	#N/A	#N/A	#N/A	#N/A	#N/A	#N/A	#N/A	#N/A
TRINITY_DN12056_c0_g1_i2	P2C47_ORYSJ	8.24	-0.33	2.0E-07	1.8E-05	#N/A	#N/A	#N/A	#N/A	#N/A	#N/A	#N/A	#N/A
TRINITY_DN16756_c1_g1_i7	U587_CAEEL	8.25	-0.32	1.7E-08	1.8E-06	#N/A	#N/A	#N/A	#N/A	#N/A	#N/A	#N/A	#N/A
TRINITY_DN19103_c0_g1_i4	MYOB2_ARATH	8.31	-0.26	2.2E-09	2.6E-07	#N/A	#N/A	#N/A	#N/A	#N/A	#N/A	#N/A	#N/A
TRINITY_DN18702_c1_g2_i1	CIPK9_ORYSJ	8.32	-0.25	6.5E-08	6.4E-06	#N/A	#N/A	#N/A	#N/A	#N/A	#N/A	#N/A	#N/A
TRINITY_DN21489_c0_g2_i7	P2C11_ORYSJ	8.37	2.44	2.1E-33	2.2E-30	#N/A	#N/A	#N/A	#N/A	#N/A	#N/A	#N/A	#N/A
TRINITY_DN16355_c0_g1_i30	P2A13_ARATH	8.39	-0.19	1.3E-04	5.8E-03	#N/A	#N/A	#N/A	#N/A	#N/A	#N/A	#N/A	#N/A
TRINITY_DN14981_c0_g1_i6	UBP21_ARATH	8.40	-0.18	1.5E-06	1.1E-04	#N/A	#N/A	#N/A	#N/A	#N/A	#N/A	#N/A	#N/A
TRINITY_DN16911_c7_g1_i6	FTSH9_ORYSJ	8.44	-0.15	4.2E-10	5.7E-08	#N/A	#N/A	#N/A	#N/A	#N/A	#N/A	#N/A	#N/A
TRINITY_DN17243_c1_g3_i5	NUD13_ARATH	8.46	-0.13	1.2E-09	1.5E-07	#N/A	#N/A	#N/A	#N/A	#N/A	#N/A	#N/A	#N/A
TRINITY_DN18228_c0_g2_i7	PIGG_HUMAN	8.47	-0.13	3.6E-06	2.4E-04	#N/A	#N/A	#N/A	#N/A	#N/A	#N/A	#N/A	#N/A
TRINITY_DN19075_c0_g1_i26	DHE4_GIAIN	8.48	-0.11	7.3E-10	9.3E-08	#N/A	#N/A	#N/A	#N/A	#N/A	#N/A	#N/A	#N/A
TRINITY_DN16355_c0_g1_i5	P2A13_ARATH	8.49	-0.10	1.9E-07	1.7E-05	#N/A	#N/A	#N/A	#N/A	#N/A	#N/A	#N/A	#N/A
TRINITY_DN13989_c0_g1_i2	DRM1_ARATH	8.51	-0.09	1.9E-06	1.3E-04	#N/A	#N/A	#N/A	#N/A	#N/A	#N/A	#N/A	#N/A
TRINITY_DN20141_c3_g1_i8	POD1_ARATH	8.53	-0.06	1.1E-10	1.7E-08	#N/A	#N/A	#N/A	#N/A	#N/A	#N/A	#N/A	#N/A
TRINITY_DN16677_c0_g2_i5	MED16_ARATH	8.54	-0.06	8.6E-07	6.6E-05	#N/A	#N/A	#N/A	#N/A	#N/A	#N/A	#N/A	#N/A
TRINITY_DN21495_c0_g1_i1	AGD14_ARATH	8.57	-0.03	2.8E-08	2.9E-06	#N/A	#N/A	#N/A	#N/A	#N/A	#N/A	#N/A	#N/A
TRINITY_DN17787_c0_g5_i1	RH26_ORYSJ	8.57	-0.03	3.6E-07	3.0E-05	#N/A	#N/A	#N/A	#N/A	#N/A	#N/A	#N/A	#N/A
TRINITY_DN21543_c0_g1_i1	Y005_SYNY3	8.60	7.74	1.1E-03	3.3E-02	#N/A	#N/A	#N/A	#N/A	#N/A	#N/A	#N/A	#N/A
TRINITY_DN18561_c0_g1_i11	ABIL5_ORYSJ	8.62	0.02	6.5E-06	4.1E-04	#N/A	#N/A	#N/A	#N/A	#N/A	#N/A	#N/A	#N/A
TRINITY_DN20039_c1_g2_i11	RNP1_ARATH	8.65	0.05	3.3E-12	5.8E-10	#N/A	#N/A	#N/A	#N/A	#N/A	#N/A	#N/A	#N/A
TRINITY_DN16688_c1_g2_i7	GBF2_ARATH	8.66	0.05	1.1E-08	1.3E-06	#N/A	#N/A	#N/A	#N/A	#N/A	#N/A	#N/A	#N/A
TRINITY_DN16735_c0_g1_i10	GSH1A_ORYSI	8.67	0.06	2.0E-07	1.8E-05	#N/A	#N/A	#N/A	#N/A	#N/A	#N/A	#N/A	#N/A
TRINITY_DN21010_c0_g1_i20	MAP1B_ARATH	8.71	0.10	1.2E-07	1.1E-05	#N/A	#N/A	#N/A	#N/A	#N/A	#N/A	#N/A	#N/A
TRINITY_DN21643_c1_g2_i6	GBA2_MOUSE	8.71	0.11	7.8E-13	1.5E-10	#N/A	#N/A	#N/A	#N/A	#N/A	#N/A	#N/A	#N/A
TRINITY_DN17351_c0_g2_i3	MYO6_ARATH	8.73	0.12	1.0E-06	7.9E-05	#N/A	#N/A	#N/A	#N/A	#N/A	#N/A	#N/A	#N/A
TRINITY_DN18154_c0_g1_i11	Y4554_ARATH	8.73	0.12	4.4E-09	5.1E-07	#N/A	#N/A	#N/A	#N/A	#N/A	#N/A	#N/A	#N/A
TRINITY_DN21018_c0_g1_i11	NPC1_ARATH	8.76	0.15	9.4E-07	7.2E-05	#N/A	#N/A	#N/A	#N/A	#N/A	#N/A	#N/A	#N/A
TRINITY_DN21506_c1_g1_i5	NUP88_ARATH	8.85	0.24	3.3E-09	3.9E-07	#N/A	#N/A	#N/A	#N/A	#N/A	#N/A	#N/A	#N/A
TRINITY_DN18372_c0_g1_i13	SPIN1_ORYSJ	8.90	0.28	4.3E-09	5.0E-07	#N/A	#N/A	#N/A	#N/A	#N/A	#N/A	#N/A	#N/A
TRINITY_DN17592_c0_g2_i5	WRKY1_MAIZE	8.91	0.28	4.6E-12	7.9E-10	#N/A	#N/A	#N/A	#N/A	#N/A	#N/A	#N/A	#N/A
TRINITY_DN20996_c2_g2_i4	MED13_ARATH	8.92	0.30	2.0E-07	1.7E-05	#N/A	#N/A	#N/A	#N/A	#N/A	#N/A	#N/A	#N/A
TRINITY_DN20040_c0_g1_i19	RS242_ARATH	8.93	0.30	4.0E-14	8.9E-12	#N/A	#N/A	#N/A	#N/A	#N/A	#N/A	#N/A	#N/A
TRINITY_DN21670_c1_g1_i6	RNHX1_ARATH	8.96	0.33	3.8E-07	3.2E-05	#N/A	#N/A	#N/A	#N/A	#N/A	#N/A	#N/A	#N/A

DET	Subject id	c9TR vs c7NT				c7TR vs c7NT				c7TR vs c9TR			
		Log_2	Log_2CPM	PValue	19FDR	Log_2	Log_2CPM	PValue	19FDR	Log_2	Log_2CPM	PValue	19FDR
TRINITY_DN22274_c1_g1_i5	FRS5_ARATH	8.96	0.33	6.6E-06	4.1E-04	#N/A	#N/A	#N/A	#N/A	#N/A	#N/A	#N/A	#N/A
TRINITY_DN21795_c0_g1_i8	HIP1_ORYSJ	8.99	0.37	2.4E-07	2.1E-05	#N/A	#N/A	#N/A	#N/A	#N/A	#N/A	#N/A	#N/A
TRINITY_DN14356_c0_g1_i1	P1_ARATH	9.00	0.37	2.0E-14	4.4E-12	#N/A	#N/A	#N/A	#N/A	#N/A	#N/A	#N/A	#N/A
TRINITY_DN16105_c1_g2_i2	BCHB_ARATH	9.04	0.41	4.5E-11	6.9E-09	#N/A	#N/A	#N/A	#N/A	#N/A	#N/A	#N/A	#N/A
TRINITY_DN17110_c0_g1_i12	AB28G_ARATH	9.08	0.45	5.8E-12	1.0E-09	#N/A	#N/A	#N/A	#N/A	#N/A	#N/A	#N/A	#N/A
TRINITY_DN22222_c0_g1_i7	PHYC_SORBI	9.10	0.47	5.0E-10	6.6E-08	#N/A	#N/A	#N/A	#N/A	#N/A	#N/A	#N/A	#N/A
TRINITY_DN21625_c0_g3_i2	CBP23_HORVU	9.13	0.49	6.7E-09	7.6E-07	#N/A	#N/A	#N/A	#N/A	#N/A	#N/A	#N/A	#N/A
TRINITY_DN16232_c1_g2_i14	ODBA2_ARATH	9.33	0.68	1.4E-04	6.1E-03	#N/A	#N/A	#N/A	#N/A	#N/A	#N/A	#N/A	#N/A
TRINITY_DN21474_c0_g1_i2	GT14_ORYSJ	9.35	0.70	1.6E-10	2.3E-08	#N/A	#N/A	#N/A	#N/A	#N/A	#N/A	#N/A	#N/A
TRINITY_DN16858_c0_g1_i7	PP127_ARATH	9.36	0.71	2.4E-16	6.6E-14	#N/A	#N/A	#N/A	#N/A	#N/A	#N/A	#N/A	#N/A
TRINITY_DN20646_c1_g3_i11	SKI3_ARATH	9.39	0.74	4.7E-14	1.0E-11	#N/A	#N/A	#N/A	#N/A	#N/A	#N/A	#N/A	#N/A
TRINITY_DN15514_c2_g1_i4	RK11_ARATH	9.40	0.75	1.3E-13	2.6E-11	#N/A	#N/A	#N/A	#N/A	#N/A	#N/A	#N/A	#N/A
TRINITY_DN20777_c0_g2_i1	YBEY_SYNPX	9.42	0.77	7.8E-17	2.2E-14	#N/A	#N/A	#N/A	#N/A	#N/A	#N/A	#N/A	#N/A
TRINITY_DN20039_c1_g2_i9	RNP1_ARATH	9.44	0.79	2.5E-16	6.7E-14	#N/A	#N/A	#N/A	#N/A	#N/A	#N/A	#N/A	#N/A
TRINITY_DN17663_c0_g1_i5	GDL83_ARATH	9.48	0.82	2.1E-07	1.9E-05	#N/A	#N/A	#N/A	#N/A	#N/A	#N/A	#N/A	#N/A
TRINITY_DN22515_c1_g1_i16	NIPA4_ARATH	9.48	0.83	3.0E-12	5.3E-10	#N/A	#N/A	#N/A	#N/A	#N/A	#N/A	#N/A	#N/A
TRINITY_DN20763_c0_g1_i5	TAF1_ORYSJ	9.56	0.91	5.7E-10	7.5E-08	#N/A	#N/A	#N/A	#N/A	#N/A	#N/A	#N/A	#N/A
TRINITY_DN19545_c1_g1_i25	DEGP9_ARATH	9.58	0.92	1.1E-12	2.1E-10	#N/A	#N/A	#N/A	#N/A	#N/A	#N/A	#N/A	#N/A
TRINITY_DN18313_c0_g2_i2	PP281_ARATH	9.60	0.94	6.7E-06	4.2E-04	#N/A	#N/A	#N/A	#N/A	#N/A	#N/A	#N/A	#N/A
TRINITY_DN14322_c0_g1_i6	UEV1A_ARATH	9.64	0.98	7.6E-19	2.6E-16	#N/A	#N/A	#N/A	#N/A	#N/A	#N/A	#N/A	#N/A
TRINITY_DN22289_c0_g1_i2	CDKG2_ORYSJ	9.64	0.98	2.1E-18	7.0E-16	#N/A	#N/A	#N/A	#N/A	#N/A	#N/A	#N/A	#N/A
TRINITY_DN19714_c0_g1_i8	PBS1_ARATH	9.76	1.10	4.4E-18	1.4E-15	#N/A	#N/A	#N/A	#N/A	#N/A	#N/A	#N/A	#N/A
TRINITY_DN19237_c0_g1_i18	PPD5_ARATH	9.80	1.13	2.2E-13	4.4E-11	#N/A	#N/A	#N/A	#N/A	#N/A	#N/A	#N/A	#N/A
TRINITY_DN14888_c0_g2_i5	AMT32_ORYSJ	9.83	1.16	1.7E-07	1.5E-05	#N/A	#N/A	#N/A	#N/A	#N/A	#N/A	#N/A	#N/A
TRINITY_DN20126_c0_g1_i4	COB21_ARATH	9.83	1.16	2.5E-19	8.8E-17	#N/A	#N/A	#N/A	#N/A	#N/A	#N/A	#N/A	#N/A
TRINITY_DN22604_c0_g1_i2	VP13A_DICDI	9.85	1.18	2.8E-08	2.9E-06	#N/A	#N/A	#N/A	#N/A	#N/A	#N/A	#N/A	#N/A
TRINITY_DN17787_c0_g5_i4	RH25_ORYSJ	9.86	1.19	2.8E-18	8.9E-16	#N/A	#N/A	#N/A	#N/A	#N/A	#N/A	#N/A	#N/A
TRINITY_DN21496_c1_g1_i3	GYP7_YARLI	9.87	1.20	4.6E-15	1.1E-12	#N/A	#N/A	#N/A	#N/A	#N/A	#N/A	#N/A	#N/A
TRINITY_DN20126_c0_g1_i7	COB21_ORYSJ	9.93	1.26	1.7E-19	6.0E-17	#N/A	#N/A	#N/A	#N/A	#N/A	#N/A	#N/A	#N/A
TRINITY_DN22683_c1_g1_i3	LORF2_MOUSE	9.94	1.27	4.0E-18	1.3E-15	#N/A	#N/A	#N/A	#N/A	#N/A	#N/A	#N/A	#N/A
TRINITY_DN15536_c0_g3_i5	PRMS_MAIZE	10.03	1.36	1.2E-06	9.1E-05	#N/A	#N/A	#N/A	#N/A	#N/A	#N/A	#N/A	#N/A
TRINITY_DN21331_c0_g1_i13	AB12A_ARATH	10.05	1.38	2.0E-19	6.9E-17	#N/A	#N/A	#N/A	#N/A	#N/A	#N/A	#N/A	#N/A
TRINITY_DN17264_c0_g2_i5	SSY4_ARATH	10.07	1.40	8.4E-21	3.3E-18	#N/A	#N/A	#N/A	#N/A	#N/A	#N/A	#N/A	#N/A
TRINITY_DN19937_c3_g1_i18	THIC_ARATH	10.09	1.41	3.9E-22	1.7E-19	#N/A	#N/A	#N/A	#N/A	#N/A	#N/A	#N/A	#N/A
TRINITY_DN22604_c0_g1_i1	VP13C_HUMAN	10.12	1.45	1.5E-06	1.1E-04	#N/A	#N/A	#N/A	#N/A	#N/A	#N/A	#N/A	#N/A
TRINITY_DN15428_c0_g1_i6	PMP22_ARATH	10.16	1.49	5.6E-21	2.3E-18	#N/A	#N/A	#N/A	#N/A	#N/A	#N/A	#N/A	#N/A
TRINITY_DN20927_c0_g1_i12	GT644_ARATH	10.34	1.66	5.3E-15	1.3E-12	#N/A	#N/A	#N/A	#N/A	#N/A	#N/A	#N/A	#N/A
TRINITY_DN20126_c0_g1_i14	COB21_ORYSJ	10.41	1.73	1.6E-21	6.4E-19	#N/A	#N/A	#N/A	#N/A	#N/A	#N/A	#N/A	#N/A
TRINITY_DN20556_c0_g1_i1	RER6_ARATH	10.55	1.86	2.0E-22	9.2E-20	#N/A	#N/A	#N/A	#N/A	#N/A	#N/A	#N/A	#N/A
TRINITY_DN16355_c0_g1_i14	P2A13_ARATH	10.74	2.04	2.1E-03	5.4E-02	#N/A	#N/A	#N/A	#N/A	#N/A	#N/A	#N/A	#N/A
TRINITY_DN19292_c0_g2_i3	ILL1_ORYSJ	10.80	2.10	5.6E-29	4.3E-26	#N/A	#N/A	#N/A	#N/A	#N/A	#N/A	#N/A	#N/A
TRINITY_DN19658_c0_g1_i5	RPK2_ARATH	10.83	2.13	1.1E-16	3.0E-14	#N/A	#N/A	#N/A	#N/A	#N/A	#N/A	#N/A	#N/A
TRINITY_DN19513_c0_g3_i2	RL354_ARATH	11.12	2.42	3.2E-31	2.9E-28	#N/A	#N/A	#N/A	#N/A	#N/A	#N/A	#N/A	#N/A
TRINITY_DN22502_c1_g1_i19	EDR1_ARATH	11.16	2.46	1.9E-19	6.8E-17	#N/A	#N/A	#N/A	#N/A	#N/A	#N/A	#N/A	#N/A
TRINITY_DN18888_c0_g2_i12	ARSB_DICDI	11.44	2.74	4.5E-38	5.6E-35	#N/A	#N/A	#N/A	#N/A	#N/A	#N/A	#N/A	#N/A
TRINITY_DN22491_c1_g2_i3	Y1669_ARATH	11.54	2.83	1.6E-03	4.4E-02	#N/A	#N/A	#N/A	#N/A	#N/A	#N/A	#N/A	#N/A
TRINITY_DN19210_c1_g3_i2	GAP1_ORYSJ	11.55	2.84	3.6E-44	5.8E-41	#N/A	#N/A	#N/A	#N/A	#N/A	#N/A	#N/A	#N/A
TRINITY_DN19392_c1_g2_i9	RL23_ARATH	11.57	2.87	1.5E-03	4.3E-02	#N/A	#N/A	#N/A	#N/A	#N/A	#N/A	#N/A	#N/A
TRINITY_DN16687_c0_g2_i9	BGL31_ORYSJ	11.89	3.18	1.7E-03	4.7E-02	#N/A	#N/A	#N/A	#N/A	#N/A	#N/A	#N/A	#N/A
TRINITY_DN22242_c0_g1_i1	TSS_ARATH	12.09	3.38	1.3E-03	3.9E-02	#N/A	#N/A	#N/A	#N/A	#N/A	#N/A	#N/A	#N/A
TRINITY_DN16687_c0_g2_i2	BGL31_ORYSJ	12.16	3.45	1.3E-03	3.8E-02	#N/A	#N/A	#N/A	#N/A	#N/A	#N/A	#N/A	#N/A
TRINITY_DN16957_c0_g2_i1	CSCLD_ARATH	13.15	4.43	1.1E-03	3.3E-02	#N/A	#N/A	#N/A	#N/A	#N/A	#N/A	#N/A	#N/A
TRINITY_DN19851_c2_g3_i10	PGMP_PEA	13.91	5.19	2.4E-111	5.3E-107	#N/A	#N/A	#N/A	#N/A	#N/A	#N/A	#N/A	#N/A
TRINITY_DN15273_c1_g1_i15	2NPD_BACSU	#N/A	#N/A	#N/A	#N/A	#N/A	#N/A	#N/A	#N/A	-9.90	1.19	1.2E-17	5.1E-15
TRINITY_DN17075_c1_g5_i6	AB11I_ARATH	#N/A	#N/A	#N/A	#N/A	#N/A	#N/A	#N/A	#N/A	-1.56	3.37	2.6E-04	1.8E-02
TRINITY_DN18541_c0_g1_i81	AB2C_ARATH	#N/A	#N/A	#N/A	#N/A	#N/A	#N/A	#N/A	#N/A	-8.67	0.03	1.5E-10	2.9E-08
TRINITY_DN21067_c0_g1_i4	ACA9_ARATH	#N/A	#N/A	#N/A	#N/A	#N/A	#N/A	#N/A	#N/A	8.41	-0.23	4.7E-07	5.6E-05
TRINITY_DN16255_c1_g2_i2	AFC2_ARATH	#N/A	#N/A	#N/A	#N/A	#N/A	#N/A	#N/A	#N/A	-9.36	0.67	3.9E-05	3.3E-03
TRINITY_DN22344_c2_g3_i1	AGT23_ARATH	#N/A	#N/A	#N/A	#N/A	#N/A	#N/A	#N/A	#N/A	-11.59	2.84	8.4E-38	1.8E-34
TRINITY_DN20066_c0_g3_i21	AKRC9_ARATH	#N/A	#N/A	#N/A	#N/A	#N/A	#N/A	#N/A	#N/A	8.32	-0.31	2.5E-04	1.7E-02
TRINITY_DN20066_c0_g3_i8	AKRC9_ARATH	#N/A	#N/A	#N/A	#N/A	#N/A	#N/A	#N/A	#N/A	1.86	1.57	4.9E-04	3.2E-02

DET	Subject id	c9TR vs c7NT				c7TR vs c7NT				c7TR vs c9TR			
		Log_2	Log_2CPM	PValue	19FDR	Log_2	Log_2CPM	PValue	19FDR	Log_2	Log_2CPM	PValue	19FDR
TRINITY_DN15268_c0_g1_i3	AKRC9_ARATH	#N/A	#N/A	#N/A	#N/A	#N/A	#N/A	#N/A	#N/A	-2.38	0.03	8.6E-04	5.2E-02
TRINITY_DN20066_c0_g3_i19	AKRCA_ARATH	#N/A	#N/A	#N/A	#N/A	#N/A	#N/A	#N/A	#N/A	3.09	2.11	3.3E-07	4.0E-05
TRINITY_DN15761_c0_g1_i1	AL3F1_ARATH	#N/A	#N/A	#N/A	#N/A	#N/A	#N/A	#N/A	#N/A	1.95	0.09	9.1E-04	5.4E-02
TRINITY_DN22489_c1_g1_i10	ALA9_ARATH	#N/A	#N/A	#N/A	#N/A	#N/A	#N/A	#N/A	#N/A	-1.83	1.12	1.3E-04	9.7E-03
TRINITY_DN22705_c1_g6_i2	ALDO2_MAIZE	#N/A	#N/A	#N/A	#N/A	#N/A	#N/A	#N/A	#N/A	-3.85	0.97	3.6E-10	6.8E-08
TRINITY_DN18282_c1_g7_i1	ALDO2_MAIZE	#N/A	#N/A	#N/A	#N/A	#N/A	#N/A	#N/A	#N/A	-2.80	1.27	1.0E-08	1.5E-06
TRINITY_DN22705_c1_g14_i1	ALDO2_MAIZE	#N/A	#N/A	#N/A	#N/A	#N/A	#N/A	#N/A	#N/A	-1.92	2.25	4.0E-06	4.0E-04
TRINITY_DN21349_c0_g3_i2	ALIX_DICDI	#N/A	#N/A	#N/A	#N/A	#N/A	#N/A	#N/A	#N/A	9.08	0.39	2.3E-08	3.4E-06
TRINITY_DN22373_c0_g1_i23	ANM7_ORYSI	#N/A	#N/A	#N/A	#N/A	#N/A	#N/A	#N/A	#N/A	8.32	-0.30	1.2E-04	8.7E-03
TRINITY_DN18749_c1_g3_i2	APC2_ARATH	#N/A	#N/A	#N/A	#N/A	#N/A	#N/A	#N/A	#N/A	9.22	0.53	1.4E-11	3.1E-09
TRINITY_DN17612_c0_g1_i7	AROD1_ARATH	#N/A	#N/A	#N/A	#N/A	#N/A	#N/A	#N/A	#N/A	8.79	0.12	7.8E-08	1.1E-05
TRINITY_DN16213_c0_g1_i7	ATG16_ARATH	#N/A	#N/A	#N/A	#N/A	#N/A	#N/A	#N/A	#N/A	-2.28	2.05	9.2E-05	7.1E-03
TRINITY_DN18152_c1_g1_i10	ATK1_ARATH	#N/A	#N/A	#N/A	#N/A	#N/A	#N/A	#N/A	#N/A	2.65	2.81	3.5E-04	2.3E-02
TRINITY_DN20094_c2_g2_i6	B120_ARATH	#N/A	#N/A	#N/A	#N/A	#N/A	#N/A	#N/A	#N/A	-7.98	-0.60	9.5E-07	1.0E-04
TRINITY_DN19905_c0_g1_i4	BGA12_ORYSJ	#N/A	#N/A	#N/A	#N/A	#N/A	#N/A	#N/A	#N/A	-2.89	1.15	5.0E-04	3.2E-02
TRINITY_DN19411_c1_g3_i3	BGH3B_BACO1	#N/A	#N/A	#N/A	#N/A	#N/A	#N/A	#N/A	#N/A	-1.69	0.95	5.2E-04	3.4E-02
TRINITY_DN18705_c0_g1_i10	BGL05_ORYSJ	#N/A	#N/A	#N/A	#N/A	#N/A	#N/A	#N/A	#N/A	-10.17	1.46	1.1E-09	1.8E-07
TRINITY_DN18705_c0_g1_i15	BGL05_ORYSJ	#N/A	#N/A	#N/A	#N/A	#N/A	#N/A	#N/A	#N/A	-8.98	0.32	9.9E-09	1.5E-06
TRINITY_DN17518_c0_g3_i13	BH074_ARATH	#N/A	#N/A	#N/A	#N/A	#N/A	#N/A	#N/A	#N/A	8.59	-0.05	4.1E-06	4.1E-04
TRINITY_DN17786_c0_g1_i3	BISS_ZINOF	#N/A	#N/A	#N/A	#N/A	#N/A	#N/A	#N/A	#N/A	-6.42	0.46	2.9E-09	4.8E-07
TRINITY_DN18420_c0_g5_i1	BLH9_ARATH	#N/A	#N/A	#N/A	#N/A	#N/A	#N/A	#N/A	#N/A	3.92	0.08	1.6E-04	1.2E-02
TRINITY_DN20669_c0_g3_i6	BX7_MAIZE	#N/A	#N/A	#N/A	#N/A	#N/A	#N/A	#N/A	#N/A	-12.68	3.93	7.5E-47	2.2E-43
TRINITY_DN18614_c1_g2_i6	BZW2_MACFA	#N/A	#N/A	#N/A	#N/A	#N/A	#N/A	#N/A	#N/A	-4.72	0.24	6.7E-07	7.5E-05
TRINITY_DN20358_c0_g1_i20	C3H6_ORYSJ	#N/A	#N/A	#N/A	#N/A	#N/A	#N/A	#N/A	#N/A	-7.24	1.27	5.8E-12	1.3E-09
TRINITY_DN20358_c0_g1_i10	C3H6_ORYSJ	#N/A	#N/A	#N/A	#N/A	#N/A	#N/A	#N/A	#N/A	4.04	-0.31	2.0E-04	1.4E-02
TRINITY_DN15234_c0_g2_i1	C7A14_ARATH	#N/A	#N/A	#N/A	#N/A	#N/A	#N/A	#N/A	#N/A	-4.74	0.36	8.8E-04	5.3E-02
TRINITY_DN22360_c1_g2_i4	C7A15_ARATH	#N/A	#N/A	#N/A	#N/A	#N/A	#N/A	#N/A	#N/A	-2.41	0.88	1.8E-05	1.7E-03
TRINITY_DN16690_c2_g10_i1	C7A15_ARATH	#N/A	#N/A	#N/A	#N/A	#N/A	#N/A	#N/A	#N/A	-1.78	2.30	2.0E-04	1.4E-02
TRINITY_DN22360_c1_g2_i1	C7A29_PANGI	#N/A	#N/A	#N/A	#N/A	#N/A	#N/A	#N/A	#N/A	-3.19	1.44	8.9E-10	1.6E-07
TRINITY_DN22360_c1_g2_i2	C7A29_PANGI	#N/A	#N/A	#N/A	#N/A	#N/A	#N/A	#N/A	#N/A	-2.94	3.67	8.5E-08	1.1E-05
TRINITY_DN22360_c1_g2_i3	C7A29_PANGI	#N/A	#N/A	#N/A	#N/A	#N/A	#N/A	#N/A	#N/A	-1.94	4.91	2.5E-05	2.2E-03
TRINITY_DN19479_c1_g1_i4	C7D55_HYOMU	#N/A	#N/A	#N/A	#N/A	#N/A	#N/A	#N/A	#N/A	0.93	5.04	8.9E-04	5.3E-02
TRINITY_DN14609_c0_g1_i3	C81E1_GLYEC	#N/A	#N/A	#N/A	#N/A	#N/A	#N/A	#N/A	#N/A	-1.75	4.02	1.6E-04	1.2E-02
TRINITY_DN16167_c0_g2_i3	C84A1_ARATH	#N/A	#N/A	#N/A	#N/A	#N/A	#N/A	#N/A	#N/A	8.40	-0.23	2.9E-07	3.6E-05
TRINITY_DN15112_c2_g3_i25	CADH1_ORYSJ	#N/A	#N/A	#N/A	#N/A	#N/A	#N/A	#N/A	#N/A	-1.02	6.34	6.4E-04	4.0E-02
TRINITY_DN16891_c0_g1_i6	CAF1G_ARATH	#N/A	#N/A	#N/A	#N/A	#N/A	#N/A	#N/A	#N/A	1.78	2.51	7.5E-04	4.6E-02
TRINITY_DN17634_c0_g2_i5	CCH_ARATH	#N/A	#N/A	#N/A	#N/A	#N/A	#N/A	#N/A	#N/A	2.16	8.48	2.9E-10	5.5E-08
TRINITY_DN17634_c0_g2_i2	CCH_ARATH	#N/A	#N/A	#N/A	#N/A	#N/A	#N/A	#N/A	#N/A	2.00	1.95	5.5E-06	5.4E-04
TRINITY_DN18629_c0_g1_i11	CCX4_ARATH	#N/A	#N/A	#N/A	#N/A	#N/A	#N/A	#N/A	#N/A	-7.69	-0.85	1.1E-05	1.0E-03
TRINITY_DN21761_c3_g1_i1	CERK1_ORYSJ	#N/A	#N/A	#N/A	#N/A	#N/A	#N/A	#N/A	#N/A	7.61	-0.94	9.5E-05	7.3E-03
TRINITY_DN18098_c1_g1_i4	CHR28_ARATH	#N/A	#N/A	#N/A	#N/A	#N/A	#N/A	#N/A	#N/A	-2.31	0.51	5.4E-05	4.5E-03
TRINITY_DN20397_c2_g3_i2	CHUP1_ARATH	#N/A	#N/A	#N/A	#N/A	#N/A	#N/A	#N/A	#N/A	13.82	5.07	7.1E-04	4.4E-02
TRINITY_DN22739_c2_g3_i3	CIPK2_ORYSJ	#N/A	#N/A	#N/A	#N/A	#N/A	#N/A	#N/A	#N/A	1.76	1.49	2.5E-04	1.7E-02
TRINITY_DN18469_c0_g3_i12	CLPD1_ORYSJ	#N/A	#N/A	#N/A	#N/A	#N/A	#N/A	#N/A	#N/A	-1.12	5.36	2.2E-06	2.4E-04
TRINITY_DN16860_c2_g1_i5	COL16_ARATH	#N/A	#N/A	#N/A	#N/A	#N/A	#N/A	#N/A	#N/A	-10.49	1.77	3.5E-05	3.0E-03
TRINITY_DN16427_c0_g11_i4	CRIPT_RAT	#N/A	#N/A	#N/A	#N/A	#N/A	#N/A	#N/A	#N/A	-9.41	0.72	1.6E-14	4.9E-12
TRINITY_DN17145_c2_g1_i4	CRK10_ARATH	#N/A	#N/A	#N/A	#N/A	#N/A	#N/A	#N/A	#N/A	-8.00	-0.58	4.3E-07	5.1E-05
TRINITY_DN15466_c0_g4_i1	CRS1_MAIZE	#N/A	#N/A	#N/A	#N/A	#N/A	#N/A	#N/A	#N/A	-10.07	1.36	3.8E-18	1.7E-15
TRINITY_DN16819_c0_g1_i12	CYSKP_SPIOL	#N/A	#N/A	#N/A	#N/A	#N/A	#N/A	#N/A	#N/A	-8.76	0.10	2.1E-08	3.2E-06
TRINITY_DN16819_c0_g1_i2	CYSKP_SPIOL	#N/A	#N/A	#N/A	#N/A	#N/A	#N/A	#N/A	#N/A	-1.11	3.96	3.7E-05	3.2E-03
TRINITY_DN20846_c2_g1_i11	DCL4_ORYSJ	#N/A	#N/A	#N/A	#N/A	#N/A	#N/A	#N/A	#N/A	2.55	2.53	1.2E-05	1.1E-03
TRINITY_DN20924_c1_g1_i11	DDPS2_ARATH	#N/A	#N/A	#N/A	#N/A	#N/A	#N/A	#N/A	#N/A	-1.14	4.54	2.0E-05	1.8E-03
TRINITY_DN18795_c0_g1_i5	DEAHD_ARATH	#N/A	#N/A	#N/A	#N/A	#N/A	#N/A	#N/A	#N/A	-7.71	0.97	1.2E-06	1.3E-04
TRINITY_DN18855_c0_g1_i5	DGDG1_SOYBN	#N/A	#N/A	#N/A	#N/A	#N/A	#N/A	#N/A	#N/A	-1.64	1.54	3.5E-04	2.4E-02
TRINITY_DN22635_c1_g3_i6	DME_ARATH	#N/A	#N/A	#N/A	#N/A	#N/A	#N/A	#N/A	#N/A	-8.31	-0.30	9.6E-09	1.5E-06
TRINITY_DN12491_c0_g1_i3	DNJ16_ARATH	#N/A	#N/A	#N/A	#N/A	#N/A	#N/A	#N/A	#N/A	-8.64	0.00	5.0E-09	8.1E-07
TRINITY_DN18398_c3_g4_i4	DNJ63_ARATH	#N/A	#N/A	#N/A	#N/A	#N/A	#N/A	#N/A	#N/A	10.42	1.70	3.6E-07	4.4E-05
TRINITY_DN17609_c0_g2_i6	DRE2_MAIZE	#N/A	#N/A	#N/A	#N/A	#N/A	#N/A	#N/A	#N/A	2.68	2.51	2.5E-04	1.8E-02
TRINITY_DN19510_c0_g1_i17	DSP8_ARATH	#N/A	#N/A	#N/A	#N/A	#N/A	#N/A	#N/A	#N/A	7.99	-0.62	3.0E-06	3.0E-04
TRINITY_DN16002_c0_g4_i2	DST1_DICDI	#N/A	#N/A	#N/A	#N/A	#N/A	#N/A	#N/A	#N/A	-8.97	0.31	8.6E-13	2.2E-10
TRINITY_DN16867_c0_g3_i3	DTX16_ARATH	#N/A	#N/A	#N/A	#N/A	#N/A	#N/A	#N/A	#N/A	-3.36	3.42	7.5E-09	1.2E-06

DET	Subject id	c9TR vs c7NT				c7TR vs c7NT				c7TR vs c9TR			
		Log_2	Log_2CPM	PValue	19FDR	Log_2	Log_2CPM	PValue	19FDR	Log_2	Log_2CPM	PValue	19FDR
TRINITY_DN16867_c0_g3_i4	DTX16_ARATH	#N/A	#N/A	#N/A	#N/A	#N/A	#N/A	#N/A	#N/A	1.45	2.89	1.8E-04	1.3E-02
TRINITY_DN16915_c0_g2_i1	E1313_ARATH	#N/A	#N/A	#N/A	#N/A	#N/A	#N/A	#N/A	#N/A	-8.68	0.04	8.6E-10	1.5E-07
TRINITY_DN19452_c0_g1_i8	E1314_ARATH	#N/A	#N/A	#N/A	#N/A	#N/A	#N/A	#N/A	#N/A	-6.13	-0.52	5.6E-07	6.4E-05
TRINITY_DN20294_c0_g1_i11	EIL3_ARATH	#N/A	#N/A	#N/A	#N/A	#N/A	#N/A	#N/A	#N/A	10.35	1.63	6.6E-12	1.5E-09
TRINITY_DN21218_c0_g1_i1	EMF2_ARATH	#N/A	#N/A	#N/A	#N/A	#N/A	#N/A	#N/A	#N/A	5.93	-0.09	4.7E-06	4.7E-04
TRINITY_DN21329_c1_g1_i4	EXEC2_ORYSJ	#N/A	#N/A	#N/A	#N/A	#N/A	#N/A	#N/A	#N/A	1.25	5.54	1.2E-04	8.7E-03
TRINITY_DN19257_c0_g1_i2	FBL3_ARATH	#N/A	#N/A	#N/A	#N/A	#N/A	#N/A	#N/A	#N/A	-1.42	4.31	2.3E-05	2.0E-03
TRINITY_DN18806_c1_g1_i5	FDH1_ORYSJ	#N/A	#N/A	#N/A	#N/A	#N/A	#N/A	#N/A	#N/A	8.58	-0.07	2.6E-10	5.0E-08
TRINITY_DN19135_c2_g2_i21	FRI1_MAIZE	#N/A	#N/A	#N/A	#N/A	#N/A	#N/A	#N/A	#N/A	1.81	5.87	4.7E-04	3.1E-02
TRINITY_DN8454_c0_g1_i1	FRS5_ARATH	#N/A	#N/A	#N/A	#N/A	#N/A	#N/A	#N/A	#N/A	-5.03	-0.32	2.2E-06	2.3E-04
TRINITY_DN16271_c6_g1_i7	FUCO2_ARATH	#N/A	#N/A	#N/A	#N/A	#N/A	#N/A	#N/A	#N/A	8.65	-0.01	4.6E-09	7.5E-07
TRINITY_DN15660_c0_g3_i5	FY_ARATH	#N/A	#N/A	#N/A	#N/A	#N/A	#N/A	#N/A	#N/A	6.72	0.66	2.9E-06	3.0E-04
TRINITY_DN18926_c0_g5_i2	GAT18_ORYSJ	#N/A	#N/A	#N/A	#N/A	#N/A	#N/A	#N/A	#N/A	4.92	0.57	4.5E-04	2.9E-02
TRINITY_DN21264_c1_g1_i1	GATP1_SOLLC	#N/A	#N/A	#N/A	#N/A	#N/A	#N/A	#N/A	#N/A	1.13	2.64	8.6E-04	5.2E-02
TRINITY_DN20483_c0_g2_i4	GAUT4_ARATH	#N/A	#N/A	#N/A	#N/A	#N/A	#N/A	#N/A	#N/A	-10.47	1.75	3.4E-22	2.2E-19
TRINITY_DN19421_c1_g2_i1	GDT13_ORYSJ	#N/A	#N/A	#N/A	#N/A	#N/A	#N/A	#N/A	#N/A	-10.08	1.37	1.5E-17	6.4E-15
TRINITY_DN19421_c1_g2_i5	GDT13_ORYSJ	#N/A	#N/A	#N/A	#N/A	#N/A	#N/A	#N/A	#N/A	-9.18	0.51	1.5E-11	3.3E-09
TRINITY_DN17258_c0_g4_i5	GLR35_ARATH	#N/A	#N/A	#N/A	#N/A	#N/A	#N/A	#N/A	#N/A	-8.56	-0.08	1.2E-06	1.3E-04
TRINITY_DN21253_c1_g2_i3	GP107_MOUSE	#N/A	#N/A	#N/A	#N/A	#N/A	#N/A	#N/A	#N/A	9.12	0.44	6.4E-11	1.3E-08
TRINITY_DN20107_c1_g1_i12	GPT2_ARATH	#N/A	#N/A	#N/A	#N/A	#N/A	#N/A	#N/A	#N/A	10.93	2.20	1.9E-05	1.7E-03
TRINITY_DN20107_c1_g1_i14	GPT2_ARATH	#N/A	#N/A	#N/A	#N/A	#N/A	#N/A	#N/A	#N/A	-8.16	-0.44	3.1E-04	2.1E-02
TRINITY_DN20158_c1_g1_i5	GSH1B_ORYSI	#N/A	#N/A	#N/A	#N/A	#N/A	#N/A	#N/A	#N/A	-1.57	4.62	3.8E-04	2.5E-02
TRINITY_DN15883_c0_g2_i3	GSTF4_MAIZE	#N/A	#N/A	#N/A	#N/A	#N/A	#N/A	#N/A	#N/A	-1.63	4.97	4.4E-12	1.0E-09
TRINITY_DN15883_c0_g2_i2	GSTF4_MAIZE	#N/A	#N/A	#N/A	#N/A	#N/A	#N/A	#N/A	#N/A	-2.32	3.28	7.9E-08	1.1E-05
TRINITY_DN15883_c0_g2_i5	GSTF4_MAIZE	#N/A	#N/A	#N/A	#N/A	#N/A	#N/A	#N/A	#N/A	-1.40	5.31	4.6E-04	3.0E-02
TRINITY_DN21735_c0_g4_i4	GSTX1_SOLTU	#N/A	#N/A	#N/A	#N/A	#N/A	#N/A	#N/A	#N/A	-2.58	5.27	1.2E-04	9.3E-03
TRINITY_DN21361_c0_g1_i3	GTE7_ARATH	#N/A	#N/A	#N/A	#N/A	#N/A	#N/A	#N/A	#N/A	-6.80	1.93	1.1E-08	1.7E-06
TRINITY_DN19136_c0_g5_i2	GUN10_ORYSJ	#N/A	#N/A	#N/A	#N/A	#N/A	#N/A	#N/A	#N/A	-1.95	4.37	5.8E-05	4.7E-03
TRINITY_DN18452_c0_g1_i21	HAK22_ORYSJ	#N/A	#N/A	#N/A	#N/A	#N/A	#N/A	#N/A	#N/A	1.18	3.25	6.3E-04	4.0E-02
TRINITY_DN22700_c4_g3_i2	HFA2E_ORYSJ	#N/A	#N/A	#N/A	#N/A	#N/A	#N/A	#N/A	#N/A	9.81	1.10	7.1E-09	1.1E-06
TRINITY_DN20070_c1_g3_i4	HLS1L_ARATH	#N/A	#N/A	#N/A	#N/A	#N/A	#N/A	#N/A	#N/A	1.62	5.39	6.6E-04	4.1E-02
TRINITY_DN20802_c0_g1_i7	HMOX1_ORYSJ	#N/A	#N/A	#N/A	#N/A	#N/A	#N/A	#N/A	#N/A	2.65	1.11	1.3E-05	1.2E-03
TRINITY_DN18792_c0_g2_i10	HOX11_ORYSJ	#N/A	#N/A	#N/A	#N/A	#N/A	#N/A	#N/A	#N/A	-8.25	-0.36	4.5E-08	6.3E-06
TRINITY_DN19043_c0_g1_i10	HSD1B_ARATH	#N/A	#N/A	#N/A	#N/A	#N/A	#N/A	#N/A	#N/A	-9.32	0.63	9.8E-12	2.2E-09
TRINITY_DN19043_c0_g1_i16	HSD1B_ARATH	#N/A	#N/A	#N/A	#N/A	#N/A	#N/A	#N/A	#N/A	1.54	4.04	3.2E-07	3.9E-05
TRINITY_DN19043_c0_g1_i9	HSD2_ARATH	#N/A	#N/A	#N/A	#N/A	#N/A	#N/A	#N/A	#N/A	1.21	3.07	1.9E-04	1.3E-02
TRINITY_DN21574_c0_g1_i4	HST_TOBAC	#N/A	#N/A	#N/A	#N/A	#N/A	#N/A	#N/A	#N/A	-8.99	0.33	7.2E-14	2.1E-11
TRINITY_DN21574_c0_g1_i20	HST_TOBAC	#N/A	#N/A	#N/A	#N/A	#N/A	#N/A	#N/A	#N/A	8.93	0.26	4.6E-10	8.5E-08
TRINITY_DN15065_c0_g4_i1	IN22_MAIZE	#N/A	#N/A	#N/A	#N/A	#N/A	#N/A	#N/A	#N/A	-3.47	0.15	2.2E-06	2.3E-04
TRINITY_DN15412_c0_g1_i2	INT1_ARATH	#N/A	#N/A	#N/A	#N/A	#N/A	#N/A	#N/A	#N/A	2.14	0.22	3.3E-04	2.2E-02
TRINITY_DN18118_c0_g3_i6	IQD31_ARATH	#N/A	#N/A	#N/A	#N/A	#N/A	#N/A	#N/A	#N/A	-1.20	3.89	5.4E-04	3.5E-02
TRINITY_DN22545_c0_g1_i7	KEG_ARATH	#N/A	#N/A	#N/A	#N/A	#N/A	#N/A	#N/A	#N/A	-7.55	1.53	6.1E-09	9.7E-07
TRINITY_DN22041_c1_g1_i24	KP1_ARATH	#N/A	#N/A	#N/A	#N/A	#N/A	#N/A	#N/A	#N/A	-2.83	0.21	5.0E-05	4.2E-03
TRINITY_DN21678_c1_g1_i8	LCB1A_ORYSJ	#N/A	#N/A	#N/A	#N/A	#N/A	#N/A	#N/A	#N/A	1.36	2.86	7.3E-04	4.5E-02
TRINITY_DN16803_c0_g1_i4	LCB2A_ORYSJ	#N/A	#N/A	#N/A	#N/A	#N/A	#N/A	#N/A	#N/A	-10.01	1.29	1.1E-15	3.8E-13
TRINITY_DN16803_c0_g1_i8	LCB2A_ORYSJ	#N/A	#N/A	#N/A	#N/A	#N/A	#N/A	#N/A	#N/A	-8.24	-0.37	3.0E-07	3.7E-05
TRINITY_DN21428_c0_g1_i7	LEU1A_SOLPN	#N/A	#N/A	#N/A	#N/A	#N/A	#N/A	#N/A	#N/A	8.08	-0.52	1.5E-04	1.1E-02
TRINITY_DN17012_c0_g1_i7	LEUC_ARATH	#N/A	#N/A	#N/A	#N/A	#N/A	#N/A	#N/A	#N/A	-1.35	3.99	1.9E-05	1.7E-03
TRINITY_DN20848_c0_g2_i6	LHTL8_ARATH	#N/A	#N/A	#N/A	#N/A	#N/A	#N/A	#N/A	#N/A	2.08	0.62	1.9E-04	1.4E-02
TRINITY_DN20743_c61_g1_i5	LHY_ARATH	#N/A	#N/A	#N/A	#N/A	#N/A	#N/A	#N/A	#N/A	-8.21	-0.40	8.4E-06	8.1E-04
TRINITY_DN19656_c1_g4_i6	LIRP1_ORYSJ	#N/A	#N/A	#N/A	#N/A	#N/A	#N/A	#N/A	#N/A	0.98	6.34	4.8E-04	3.1E-02
TRINITY_DN19656_c1_g4_i3	LIRP1_ORYSJ	#N/A	#N/A	#N/A	#N/A	#N/A	#N/A	#N/A	#N/A	0.86	7.08	5.7E-04	3.6E-02
TRINITY_DN22275_c0_g1_i3	LOX1_HORVU	#N/A	#N/A	#N/A	#N/A	#N/A	#N/A	#N/A	#N/A	2.16	1.16	8.5E-05	6.6E-03
TRINITY_DN15995_c0_g4_i1	LOX3_ORYSJ	#N/A	#N/A	#N/A	#N/A	#N/A	#N/A	#N/A	#N/A	2.00	2.07	1.5E-04	1.1E-02
TRINITY_DN14836_c0_g1_i3	LOX6_ORYSJ	#N/A	#N/A	#N/A	#N/A	#N/A	#N/A	#N/A	#N/A	-9.36	0.67	1.2E-13	3.5E-11
TRINITY_DN18132_c1_g1_i4	LTPG1_ARATH	#N/A	#N/A	#N/A	#N/A	#N/A	#N/A	#N/A	#N/A	2.63	2.91	1.2E-05	1.1E-03
TRINITY_DN16790_c0_g1_i16	MA658_ARATH	#N/A	#N/A	#N/A	#N/A	#N/A	#N/A	#N/A	#N/A	-1.58	4.77	1.9E-05	1.7E-03
TRINITY_DN16241_c4_g2_i2	MAD18_ORYSJ	#N/A	#N/A	#N/A	#N/A	#N/A	#N/A	#N/A	#N/A	-9.50	0.81	3.3E-12	7.8E-10
TRINITY_DN18197_c1_g2_i5	MBF1A_ARATH	#N/A	#N/A	#N/A	#N/A	#N/A	#N/A	#N/A	#N/A	-13.51	4.76	9.1E-38	1.9E-34
TRINITY_DN18197_c1_g2_i2	MBF1A_ARATH	#N/A	#N/A	#N/A	#N/A	#N/A	#N/A	#N/A	#N/A	-8.63	-0.01	2.0E-09	3.4E-07
TRINITY_DN18197_c1_g2_i1	MBF1A_ARATH	#N/A	#N/A	#N/A	#N/A	#N/A	#N/A	#N/A	#N/A	1.02	7.03	1.6E-04	1.2E-02

DET	Subject id	c9TR vs c7NT				c7TR vs c7NT				c7TR vs c9TR			
		Log_2	Log_2CPM	PValue	19FDR	Log_2	Log_2CPM	PValue	19FDR	Log_2	Log_2CPM	PValue	19FDR
TRINITY_DN21311_c0_g3_i1	MES7_ARATH	#N/A	#N/A	#N/A	#N/A	#N/A	#N/A	#N/A	#N/A	-5.27	-0.05	4.1E-06	4.1E-04
TRINITY_DN19345_c0_g1_i8	MLOH1_HORVU	#N/A	#N/A	#N/A	#N/A	#N/A	#N/A	#N/A	#N/A	-2.28	0.33	3.5E-04	2.4E-02
TRINITY_DN19345_c0_g1_i16	MLOH1_ORYSJ	#N/A	#N/A	#N/A	#N/A	#N/A	#N/A	#N/A	#N/A	-2.36	0.03	2.1E-04	1.5E-02
TRINITY_DN15739_c0_g1_i1	MOSA_MAIZE	#N/A	#N/A	#N/A	#N/A	#N/A	#N/A	#N/A	#N/A	-2.31	0.52	6.8E-05	5.5E-03
TRINITY_DN16667_c1_g4_i5	MRH1_ARATH	#N/A	#N/A	#N/A	#N/A	#N/A	#N/A	#N/A	#N/A	2.15	0.31	6.2E-04	3.9E-02
TRINITY_DN17782_c0_g1_i2	MSRB1_ORYSJ	#N/A	#N/A	#N/A	#N/A	#N/A	#N/A	#N/A	#N/A	1.35	3.22	2.8E-04	1.9E-02
TRINITY_DN18735_c1_g1_i4	MYB44_ARATH	#N/A	#N/A	#N/A	#N/A	#N/A	#N/A	#N/A	#N/A	4.93	-0.15	8.2E-05	6.5E-03
TRINITY_DN19096_c0_g2_i4	MYBL_DICDI	#N/A	#N/A	#N/A	#N/A	#N/A	#N/A	#N/A	#N/A	-2.74	0.17	4.3E-04	2.8E-02
TRINITY_DN15683_c3_g4_i5	MYBP_MAIZE	#N/A	#N/A	#N/A	#N/A	#N/A	#N/A	#N/A	#N/A	-9.00	0.33	3.4E-06	3.5E-04
TRINITY_DN15869_c0_g7_i3	NAC68_ORYSJ	#N/A	#N/A	#N/A	#N/A	#N/A	#N/A	#N/A	#N/A	-1.96	2.07	3.7E-04	2.5E-02
TRINITY_DN18316_c0_g3_i3	NADO1_ORYSJ	#N/A	#N/A	#N/A	#N/A	#N/A	#N/A	#N/A	#N/A	1.01	5.39	1.6E-04	1.2E-02
TRINITY_DN16256_c1_g3_i1	NET3A_ARATH	#N/A	#N/A	#N/A	#N/A	#N/A	#N/A	#N/A	#N/A	1.23	3.47	5.9E-04	3.7E-02
TRINITY_DN18619_c2_g2_i7	NIN3_ORYSJ	#N/A	#N/A	#N/A	#N/A	#N/A	#N/A	#N/A	#N/A	3.88	-0.11	5.5E-04	3.5E-02
TRINITY_DN20732_c0_g1_i10	NIP22_MAIZE	#N/A	#N/A	#N/A	#N/A	#N/A	#N/A	#N/A	#N/A	-9.67	0.97	2.0E-12	4.8E-10
TRINITY_DN20319_c1_g1_i21	NLE1_ARATH	#N/A	#N/A	#N/A	#N/A	#N/A	#N/A	#N/A	#N/A	-9.06	0.39	3.3E-08	4.8E-06
TRINITY_DN16969_c1_g3_i4	NRL4_ORYSJ	#N/A	#N/A	#N/A	#N/A	#N/A	#N/A	#N/A	#N/A	7.68	0.82	5.9E-08	8.2E-06
TRINITY_DN14545_c0_g2_i7	NUCL1_ORYSJ	#N/A	#N/A	#N/A	#N/A	#N/A	#N/A	#N/A	#N/A	-10.33	1.61	4.2E-22	2.7E-19
TRINITY_DN15832_c0_g4_i1	OCT7_ARATH	#N/A	#N/A	#N/A	#N/A	#N/A	#N/A	#N/A	#N/A	8.88	1.97	4.8E-25	3.8E-22
TRINITY_DN15832_c0_g4_i2	OCT7_ARATH	#N/A	#N/A	#N/A	#N/A	#N/A	#N/A	#N/A	#N/A	-6.53	1.41	5.2E-14	1.5E-11
TRINITY_DN16936_c0_g1_i9	ODPB1_ORYSJ	#N/A	#N/A	#N/A	#N/A	#N/A	#N/A	#N/A	#N/A	3.30	1.91	3.2E-04	2.2E-02
TRINITY_DN16936_c0_g1_i7	ODPB1_ORYSJ	#N/A	#N/A	#N/A	#N/A	#N/A	#N/A	#N/A	#N/A	2.24	0.13	5.5E-04	3.5E-02
TRINITY_DN18461_c1_g1_i1	OOPDA_ARATH	#N/A	#N/A	#N/A	#N/A	#N/A	#N/A	#N/A	#N/A	-9.25	0.57	3.0E-13	8.2E-11
TRINITY_DN15393_c0_g2_i2	OPR1_ORYSJ	#N/A	#N/A	#N/A	#N/A	#N/A	#N/A	#N/A	#N/A	-1.78	4.04	7.7E-07	8.5E-05
TRINITY_DN15393_c0_g2_i3	OPR1_ORYSJ	#N/A	#N/A	#N/A	#N/A	#N/A	#N/A	#N/A	#N/A	-1.74	3.42	1.1E-05	1.0E-03
TRINITY_DN15393_c0_g2_i4	OPR1_ORYSJ	#N/A	#N/A	#N/A	#N/A	#N/A	#N/A	#N/A	#N/A	-1.45	3.51	3.4E-05	2.9E-03
TRINITY_DN16355_c0_g1_i27	P2A13_ARATH	#N/A	#N/A	#N/A	#N/A	#N/A	#N/A	#N/A	#N/A	-1.56	4.32	1.6E-04	1.1E-02
TRINITY_DN19717_c0_g1_i15	P2C15_ORYSJ	#N/A	#N/A	#N/A	#N/A	#N/A	#N/A	#N/A	#N/A	-4.46	-0.01	6.3E-05	5.1E-03
TRINITY_DN19717_c0_g1_i3	P2C15_ORYSJ	#N/A	#N/A	#N/A	#N/A	#N/A	#N/A	#N/A	#N/A	4.83	-0.59	7.9E-05	6.2E-03
TRINITY_DN17242_c0_g2_i9	P4KB1_ARATH	#N/A	#N/A	#N/A	#N/A	#N/A	#N/A	#N/A	#N/A	-2.57	1.51	1.1E-07	1.4E-05
TRINITY_DN20644_c0_g1_i4	PABP8_ARATH	#N/A	#N/A	#N/A	#N/A	#N/A	#N/A	#N/A	#N/A	-1.59	4.69	7.6E-06	7.3E-04
TRINITY_DN20644_c0_g1_i7	PABP8_ARATH	#N/A	#N/A	#N/A	#N/A	#N/A	#N/A	#N/A	#N/A	-1.02	5.20	8.0E-05	6.3E-03
TRINITY_DN18916_c1_g2_i6	PAP13_ARATH	#N/A	#N/A	#N/A	#N/A	#N/A	#N/A	#N/A	#N/A	2.51	1.47	1.3E-05	1.2E-03
TRINITY_DN19293_c0_g2_i9	PDI_MAIZE	#N/A	#N/A	#N/A	#N/A	#N/A	#N/A	#N/A	#N/A	-8.67	0.03	2.9E-11	6.2E-09
TRINITY_DN19293_c0_g2_i3	PDI_MAIZE	#N/A	#N/A	#N/A	#N/A	#N/A	#N/A	#N/A	#N/A	-7.87	-0.69	3.7E-07	4.4E-05
TRINITY_DN22845_c1_g1_i15	PDI51_ORYSJ	#N/A	#N/A	#N/A	#N/A	#N/A	#N/A	#N/A	#N/A	-9.89	1.18	8.7E-17	3.5E-14
TRINITY_DN22845_c1_g1_i16	PDI51_ORYSJ	#N/A	#N/A	#N/A	#N/A	#N/A	#N/A	#N/A	#N/A	0.90	4.36	5.8E-04	3.6E-02
TRINITY_DN19496_c0_g2_i23	PEX14_ARATH	#N/A	#N/A	#N/A	#N/A	#N/A	#N/A	#N/A	#N/A	-4.46	-0.44	5.5E-04	3.5E-02
TRINITY_DN16431_c0_g1_i15	PGP1B_ARATH	#N/A	#N/A	#N/A	#N/A	#N/A	#N/A	#N/A	#N/A	-10.20	1.49	9.7E-13	2.4E-10
TRINITY_DN16431_c0_g1_i14	PGP1B_ARATH	#N/A	#N/A	#N/A	#N/A	#N/A	#N/A	#N/A	#N/A	-7.82	-0.74	2.7E-05	2.4E-03
TRINITY_DN19784_c0_g1_i25	PGTA_HUMAN	#N/A	#N/A	#N/A	#N/A	#N/A	#N/A	#N/A	#N/A	-10.14	1.42	6.4E-15	2.1E-12
TRINITY_DN19784_c0_g1_i23	PGTA_HUMAN	#N/A	#N/A	#N/A	#N/A	#N/A	#N/A	#N/A	#N/A	-9.01	0.34	3.7E-09	6.2E-07
TRINITY_DN18651_c1_g4_i6	PIAL2_ARATH	#N/A	#N/A	#N/A	#N/A	#N/A	#N/A	#N/A	#N/A	-9.80	1.10	1.1E-18	5.2E-16
TRINITY_DN18782_c1_g1_i12	PIN6_ORYSJ	#N/A	#N/A	#N/A	#N/A	#N/A	#N/A	#N/A	#N/A	0.94	4.81	3.1E-04	2.1E-02
TRINITY_DN21717_c3_g2_i19	PIP_ARATH	#N/A	#N/A	#N/A	#N/A	#N/A	#N/A	#N/A	#N/A	11.62	2.88	9.0E-04	5.4E-02
TRINITY_DN15886_c2_g1_i19	PIP13_MAIZE	#N/A	#N/A	#N/A	#N/A	#N/A	#N/A	#N/A	#N/A	2.59	2.14	1.8E-04	1.3E-02
TRINITY_DN20528_c1_g1_i3	PLDD1_ARATH	#N/A	#N/A	#N/A	#N/A	#N/A	#N/A	#N/A	#N/A	1.66	0.75	6.7E-04	4.1E-02
TRINITY_DN21448_c2_g1_i1	POLX_TOBAC	#N/A	#N/A	#N/A	#N/A	#N/A	#N/A	#N/A	#N/A	-1.64	1.82	1.1E-04	8.4E-03
TRINITY_DN19903_c0_g1_i5	POM1_SCHPO	#N/A	#N/A	#N/A	#N/A	#N/A	#N/A	#N/A	#N/A	-7.77	-0.78	2.2E-05	1.9E-03
TRINITY_DN22133_c2_g2_i7	POT1A_ARATH	#N/A	#N/A	#N/A	#N/A	#N/A	#N/A	#N/A	#N/A	-8.69	0.05	5.7E-09	9.2E-07
TRINITY_DN18819_c0_g1_i3	PP219_ARATH	#N/A	#N/A	#N/A	#N/A	#N/A	#N/A	#N/A	#N/A	-7.89	-0.67	8.7E-05	6.8E-03
TRINITY_DN15511_c0_g1_i2	PP279_ARATH	#N/A	#N/A	#N/A	#N/A	#N/A	#N/A	#N/A	#N/A	10.79	2.05	4.0E-06	4.0E-04
TRINITY_DN16449_c0_g5_i1	PPA27_ARATH	#N/A	#N/A	#N/A	#N/A	#N/A	#N/A	#N/A	#N/A	-1.35	4.17	8.1E-04	4.9E-02
TRINITY_DN21787_c6_g1_i11	PPDK1_MAIZE	#N/A	#N/A	#N/A	#N/A	#N/A	#N/A	#N/A	#N/A	0.86	6.13	8.9E-04	5.3E-02
TRINITY_DN15735_c0_g3_i1	PPR32_ARATH	#N/A	#N/A	#N/A	#N/A	#N/A	#N/A	#N/A	#N/A	-4.70	1.42	4.1E-07	4.9E-05
TRINITY_DN21123_c0_g1_i14	PRP39_HUMAN	#N/A	#N/A	#N/A	#N/A	#N/A	#N/A	#N/A	#N/A	-8.61	-0.03	4.7E-07	5.5E-05
TRINITY_DN20703_c1_g1_i16	PRR37_ORYSI	#N/A	#N/A	#N/A	#N/A	#N/A	#N/A	#N/A	#N/A	6.60	-0.19	4.1E-05	3.4E-03
TRINITY_DN18757_c0_g2_i1	PSBR_TOBAC	#N/A	#N/A	#N/A	#N/A	#N/A	#N/A	#N/A	#N/A	1.01	11.93	1.6E-05	1.5E-03
TRINITY_DN16548_c1_g3_i5	PSME4_ARATH	#N/A	#N/A	#N/A	#N/A	#N/A	#N/A	#N/A	#N/A	8.23	-0.40	5.4E-05	4.4E-03
TRINITY_DN20854_c2_g3_i4	PTR3_ARATH	#N/A	#N/A	#N/A	#N/A	#N/A	#N/A	#N/A	#N/A	-2.19	0.26	7.6E-04	4.6E-02
TRINITY_DN14979_c0_g4_i1	PTR36_ARATH	#N/A	#N/A	#N/A	#N/A	#N/A	#N/A	#N/A	#N/A	-3.27	0.49	2.5E-07	3.1E-05
TRINITY_DN14979_c0_g4_i4	PTR36_ARATH	#N/A	#N/A	#N/A	#N/A	#N/A	#N/A	#N/A	#N/A	-1.47	2.55	6.9E-04	4.3E-02

DET	Subject id	c9TR vs c7NT				c7TR vs c7NT				c7TR vs c9TR			
		Log_2	Log_2CPM	PValue	19FDR	Log_2	Log_2CPM	PValue	19FDR	Log_2	Log_2CPM	PValue	19FDR
TRINITY_DN17084_c0_g1_i8	PTRB_MORLA	#N/A	#N/A	#N/A	#N/A	#N/A	#N/A	#N/A	#N/A	-5.65	-0.19	1.1E-05	1.1E-03
TRINITY_DN16566_c1_g1_i17	PUM24_ARATH	#N/A	#N/A	#N/A	#N/A	#N/A	#N/A	#N/A	#N/A	-1.90	0.40	2.4E-04	1.7E-02
TRINITY_DN16566_c1_g1_i1	PUM24_ARATH	#N/A	#N/A	#N/A	#N/A	#N/A	#N/A	#N/A	#N/A	-1.82	1.83	6.4E-04	4.0E-02
TRINITY_DN18592_c1_g2_i8	PUR2_ARATH	#N/A	#N/A	#N/A	#N/A	#N/A	#N/A	#N/A	#N/A	8.65	-0.02	1.9E-05	1.7E-03
TRINITY_DN19101_c0_g1_i8	PWD_ORYSJ	#N/A	#N/A	#N/A	#N/A	#N/A	#N/A	#N/A	#N/A	1.69	2.80	3.3E-04	2.2E-02
TRINITY_DN13559_c0_g1_i3	PX111_ORYSJ	#N/A	#N/A	#N/A	#N/A	#N/A	#N/A	#N/A	#N/A	10.51	1.78	5.0E-04	3.2E-02
TRINITY_DN21150_c0_g1_i9	PXM16_ARATH	#N/A	#N/A	#N/A	#N/A	#N/A	#N/A	#N/A	#N/A	-2.26	3.32	7.9E-05	6.3E-03
TRINITY_DN16565_c0_g1_i15	RFS1_ARATH	#N/A	#N/A	#N/A	#N/A	#N/A	#N/A	#N/A	#N/A	-7.56	-0.97	2.2E-05	2.0E-03
TRINITY_DN22098_c1_g2_i21	RGA2_SOLBU	#N/A	#N/A	#N/A	#N/A	#N/A	#N/A	#N/A	#N/A	8.48	-0.17	8.6E-07	9.4E-05
TRINITY_DN21776_c0_g1_i9	RHD3_ORYSJ	#N/A	#N/A	#N/A	#N/A	#N/A	#N/A	#N/A	#N/A	-5.35	0.25	1.3E-04	9.9E-03
TRINITY_DN21900_c0_g2_i1	RIBA1_ORYSJ	#N/A	#N/A	#N/A	#N/A	#N/A	#N/A	#N/A	#N/A	-1.54	2.52	5.5E-04	3.5E-02
TRINITY_DN17831_c0_g2_i9	RIK_MAIZE	#N/A	#N/A	#N/A	#N/A	#N/A	#N/A	#N/A	#N/A	-5.06	1.46	1.5E-06	1.6E-04
TRINITY_DN16730_c0_g1_i6	RL23_ARATH	#N/A	#N/A	#N/A	#N/A	#N/A	#N/A	#N/A	#N/A	8.77	0.11	1.2E-10	2.4E-08
TRINITY_DN18725_c1_g3_i6	RL321_ARATH	#N/A	#N/A	#N/A	#N/A	#N/A	#N/A	#N/A	#N/A	-2.78	0.04	4.9E-05	4.1E-03
TRINITY_DN19513_c0_g1_i12	RL354_ARATH	#N/A	#N/A	#N/A	#N/A	#N/A	#N/A	#N/A	#N/A	8.24	-0.38	4.3E-08	6.1E-06
TRINITY_DN20559_c0_g1_i5	RL4A_ARATH	#N/A	#N/A	#N/A	#N/A	#N/A	#N/A	#N/A	#N/A	-10.12	1.41	5.4E-19	2.7E-16
TRINITY_DN16678_c0_g1_i4	RL7A_ORYSJ	#N/A	#N/A	#N/A	#N/A	#N/A	#N/A	#N/A	#N/A	8.28	-0.34	1.6E-08	2.4E-06
TRINITY_DN21043_c0_g3_i7	RLF_ARATH	#N/A	#N/A	#N/A	#N/A	#N/A	#N/A	#N/A	#N/A	8.51	-0.14	5.1E-10	9.3E-08
TRINITY_DN18462_c1_g2_i11	RMI1_ARATH	#N/A	#N/A	#N/A	#N/A	#N/A	#N/A	#N/A	#N/A	2.67	0.62	7.5E-04	4.6E-02
TRINITY_DN18462_c1_g2_i1	RMI1_ORYSJ	#N/A	#N/A	#N/A	#N/A	#N/A	#N/A	#N/A	#N/A	-4.01	0.05	5.5E-06	5.4E-04
TRINITY_DN14813_c0_g4_i4	ROC1_ORYSJ	#N/A	#N/A	#N/A	#N/A	#N/A	#N/A	#N/A	#N/A	-7.80	-0.76	5.2E-07	6.0E-05
TRINITY_DN20670_c3_g1_i2	RP44A_ARATH	#N/A	#N/A	#N/A	#N/A	#N/A	#N/A	#N/A	#N/A	-0.92	4.77	5.7E-04	3.6E-02
TRINITY_DN17475_c1_g3_i3	RPOA_MAIZE	#N/A	#N/A	#N/A	#N/A	#N/A	#N/A	#N/A	#N/A	7.83	-0.75	2.0E-06	2.2E-04
TRINITY_DN17629_c0_g1_i11	S35F1_HUMAN	#N/A	#N/A	#N/A	#N/A	#N/A	#N/A	#N/A	#N/A	7.95	-0.65	6.6E-06	6.5E-04
TRINITY_DN21850_c1_g2_i6	SAE1A_ARATH	#N/A	#N/A	#N/A	#N/A	#N/A	#N/A	#N/A	#N/A	-8.20	-0.40	1.8E-05	1.6E-03
TRINITY_DN15949_c0_g2_i5	SAHH_WHEAT	#N/A	#N/A	#N/A	#N/A	#N/A	#N/A	#N/A	#N/A	-9.16	0.49	3.4E-08	4.9E-06
TRINITY_DN17345_c0_g1_i9	SALR_PAPBR	#N/A	#N/A	#N/A	#N/A	#N/A	#N/A	#N/A	#N/A	-3.16	3.81	1.2E-04	9.1E-03
TRINITY_DN17345_c0_g1_i11	SALR_PAPBR	#N/A	#N/A	#N/A	#N/A	#N/A	#N/A	#N/A	#N/A	-3.07	2.74	1.5E-04	1.1E-02
TRINITY_DN17345_c0_g1_i7	SALR_PAPBR	#N/A	#N/A	#N/A	#N/A	#N/A	#N/A	#N/A	#N/A	-2.71	6.79	2.8E-04	1.9E-02
TRINITY_DN17495_c2_g1_i6	SAR1A_BRACM	#N/A	#N/A	#N/A	#N/A	#N/A	#N/A	#N/A	#N/A	-3.24	-0.37	4.0E-05	3.4E-03
TRINITY_DN15518_c4_g1_i3	SCGT_TOBAC	#N/A	#N/A	#N/A	#N/A	#N/A	#N/A	#N/A	#N/A	-1.04	5.34	8.7E-05	6.8E-03
TRINITY_DN20867_c0_g1_i4	SDC1_ORYSJ	#N/A	#N/A	#N/A	#N/A	#N/A	#N/A	#N/A	#N/A	-1.08	3.75	8.5E-05	6.6E-03
TRINITY_DN12748_c0_g1_i4	SERK1_ARATH	#N/A	#N/A	#N/A	#N/A	#N/A	#N/A	#N/A	#N/A	-7.18	2.40	7.4E-22	4.6E-19
TRINITY_DN12748_c0_g1_i2	SERK1_ARATH	#N/A	#N/A	#N/A	#N/A	#N/A	#N/A	#N/A	#N/A	-8.78	0.12	4.8E-07	5.6E-05
TRINITY_DN15478_c0_g1_i11	SIGB_ARATH	#N/A	#N/A	#N/A	#N/A	#N/A	#N/A	#N/A	#N/A	-3.52	0.07	6.0E-04	3.8E-02
TRINITY_DN19933_c0_g1_i8	SKI11_ARATH	#N/A	#N/A	#N/A	#N/A	#N/A	#N/A	#N/A	#N/A	5.87	1.23	9.8E-07	1.1E-04
TRINITY_DN22018_c0_g1_i10	SPSA4_ORYSJ	#N/A	#N/A	#N/A	#N/A	#N/A	#N/A	#N/A	#N/A	1.49	1.99	2.4E-04	1.7E-02
TRINITY_DN16287_c1_g1_i4	SPSA5_ORYSJ	#N/A	#N/A	#N/A	#N/A	#N/A	#N/A	#N/A	#N/A	2.26	3.28	4.7E-04	3.0E-02
TRINITY_DN16424_c1_g2_i2	SPSY_ARATH	#N/A	#N/A	#N/A	#N/A	#N/A	#N/A	#N/A	#N/A	1.64	4.32	4.3E-04	2.8E-02
TRINITY_DN14816_c1_g4_i1	SR34_ARATH	#N/A	#N/A	#N/A	#N/A	#N/A	#N/A	#N/A	#N/A	8.51	-0.14	2.5E-07	3.1E-05
TRINITY_DN21463_c0_g1_i7	SR54C_ARATH	#N/A	#N/A	#N/A	#N/A	#N/A	#N/A	#N/A	#N/A	-8.43	1.66	1.1E-18	5.1E-16
TRINITY_DN14939_c0_g1_i14	SUI1_ORYSJ	#N/A	#N/A	#N/A	#N/A	#N/A	#N/A	#N/A	#N/A	8.35	-0.28	4.4E-04	2.9E-02
TRINITY_DN15045_c3_g2_i2	SUT2_STYHA	#N/A	#N/A	#N/A	#N/A	#N/A	#N/A	#N/A	#N/A	-9.21	0.53	1.2E-07	1.6E-05
TRINITY_DN16878_c1_g1_i6	SYT5_ARATH	#N/A	#N/A	#N/A	#N/A	#N/A	#N/A	#N/A	#N/A	-3.18	3.81	9.2E-11	1.8E-08
TRINITY_DN15054_c1_g3_i1	THO4A_ARATH	#N/A	#N/A	#N/A	#N/A	#N/A	#N/A	#N/A	#N/A	-7.44	-1.06	4.1E-04	2.7E-02
TRINITY_DN21256_c1_g1_i3	TKI1_ARATH	#N/A	#N/A	#N/A	#N/A	#N/A	#N/A	#N/A	#N/A	-9.27	0.59	7.5E-14	2.1E-11
TRINITY_DN21256_c1_g1_i4	TKI1_ARATH	#N/A	#N/A	#N/A	#N/A	#N/A	#N/A	#N/A	#N/A	8.11	-0.50	1.9E-07	2.4E-05
TRINITY_DN15398_c1_g3_i5	TPPC8_HUMAN	#N/A	#N/A	#N/A	#N/A	#N/A	#N/A	#N/A	#N/A	-2.05	0.23	5.4E-04	3.4E-02
TRINITY_DN19125_c0_g1_i4	TRA1_MAIZE	#N/A	#N/A	#N/A	#N/A	#N/A	#N/A	#N/A	#N/A	-2.95	0.04	2.2E-04	1.5E-02
TRINITY_DN15749_c0_g1_i1	TTL_ARATH	#N/A	#N/A	#N/A	#N/A	#N/A	#N/A	#N/A	#N/A	2.54	1.74	3.2E-05	2.8E-03
TRINITY_DN20303_c0_g2_i7	U73C2_ARATH	#N/A	#N/A	#N/A	#N/A	#N/A	#N/A	#N/A	#N/A	-8.69	0.05	5.3E-09	8.5E-07
TRINITY_DN20303_c0_g2_i1	U73C5_ARATH	#N/A	#N/A	#N/A	#N/A	#N/A	#N/A	#N/A	#N/A	-6.27	2.97	2.2E-10	4.2E-08
TRINITY_DN20303_c0_g2_i10	U73C5_ARATH	#N/A	#N/A	#N/A	#N/A	#N/A	#N/A	#N/A	#N/A	1.29	4.62	2.3E-04	1.6E-02
TRINITY_DN20104_c0_g3_i3	U73C6_ARATH	#N/A	#N/A	#N/A	#N/A	#N/A	#N/A	#N/A	#N/A	-2.22	1.01	7.3E-05	5.8E-03
TRINITY_DN20754_c0_g1_i12	UBP1_NICPL	#N/A	#N/A	#N/A	#N/A	#N/A	#N/A	#N/A	#N/A	2.12	0.66	6.7E-05	5.4E-03
TRINITY_DN19390_c2_g1_i4	UCRIA_WHEAT	#N/A	#N/A	#N/A	#N/A	#N/A	#N/A	#N/A	#N/A	1.03	7.00	4.4E-04	2.9E-02
TRINITY_DN15213_c1_g1_i2	UGT_FRAAN	#N/A	#N/A	#N/A	#N/A	#N/A	#N/A	#N/A	#N/A	-2.68	2.55	5.1E-07	5.9E-05
TRINITY_DN20813_c0_g2_i6	UVB31_ARATH	#N/A	#N/A	#N/A	#N/A	#N/A	#N/A	#N/A	#N/A	-4.15	2.07	6.3E-10	1.1E-07
TRINITY_DN20813_c0_g2_i7	UVB31_ARATH	#N/A	#N/A	#N/A	#N/A	#N/A	#N/A	#N/A	#N/A	1.19	4.97	1.8E-04	1.3E-02
TRINITY_DN21555_c0_g2_i2	VAC14_ARATH	#N/A	#N/A	#N/A	#N/A	#N/A	#N/A	#N/A	#N/A	-6.07	0.95	7.5E-04	4.6E-02
TRINITY_DN22231_c0_g1_i6	VIL2_ARATH	#N/A	#N/A	#N/A	#N/A	#N/A	#N/A	#N/A	#N/A	-8.92	0.26	2.0E-10	3.9E-08

DET	Subject id	c9TR vs c7NT				c7TR vs c7NT				c7TR vs c9TR			
		Log_2	Log_2CPM	PValue	19FDR	Log_2	Log_2CPM	PValue	19FDR	Log_2	Log_2CPM	PValue	19FDR
TRINITY_DN22760_c1_g1_i5	VIP2_NICBE	#N/A	#N/A	#N/A	#N/A	#N/A	#N/A	#N/A	#N/A	4.91	0.36	8.0E-04	4.8E-02
TRINITY_DN17995_c0_g3_i4	VIT11_ORYSJ	#N/A	#N/A	#N/A	#N/A	#N/A	#N/A	#N/A	#N/A	-1.31	3.47	6.2E-04	3.9E-02
TRINITY_DN15449_c0_g2_i12	WNK7_ORYSJ	#N/A	#N/A	#N/A	#N/A	#N/A	#N/A	#N/A	#N/A	-10.46	1.74	2.8E-21	1.6E-18
TRINITY_DN21207_c3_g1_i9	WTR45_ARATH	#N/A	#N/A	#N/A	#N/A	#N/A	#N/A	#N/A	#N/A	2.35	3.50	4.9E-10	9.0E-08
TRINITY_DN19057_c0_g1_i8	Y1015_ARATH	#N/A	#N/A	#N/A	#N/A	#N/A	#N/A	#N/A	#N/A	1.94	5.54	1.3E-05	1.2E-03
TRINITY_DN18596_c1_g1_i1	Y1491_ARATH	#N/A	#N/A	#N/A	#N/A	#N/A	#N/A	#N/A	#N/A	-1.65	2.87	2.9E-04	2.0E-02
TRINITY_DN19981_c0_g2_i9	Y1500_ARATH	#N/A	#N/A	#N/A	#N/A	#N/A	#N/A	#N/A	#N/A	1.51	4.95	5.0E-04	3.2E-02
TRINITY_DN19981_c0_g2_i21	Y1500_ARATH	#N/A	#N/A	#N/A	#N/A	#N/A	#N/A	#N/A	#N/A	1.23	3.80	9.0E-04	5.4E-02
TRINITY_DN22491_c1_g2_i1	Y1669_ARATH	#N/A	#N/A	#N/A	#N/A	#N/A	#N/A	#N/A	#N/A	1.05	7.30	3.1E-05	2.7E-03
TRINITY_DN15350_c0_g6_i4	Y1669_ARATH	#N/A	#N/A	#N/A	#N/A	#N/A	#N/A	#N/A	#N/A	-8.87	0.21	8.4E-04	5.1E-02
TRINITY_DN22094_c0_g2_i4	Y1684_ARATH	#N/A	#N/A	#N/A	#N/A	#N/A	#N/A	#N/A	#N/A	-0.88	6.06	3.3E-04	2.2E-02
TRINITY_DN21458_c3_g2_i7	Y5457_ARATH	#N/A	#N/A	#N/A	#N/A	#N/A	#N/A	#N/A	#N/A	8.53	-0.12	6.0E-07	6.8E-05
TRINITY_DN16595_c0_g3_i2	Y6461_DICDI	#N/A	#N/A	#N/A	#N/A	#N/A	#N/A	#N/A	#N/A	-8.33	-0.29	4.9E-09	8.0E-07
TRINITY_DN18003_c0_g1_i6	Y8359_ORYSI	#N/A	#N/A	#N/A	#N/A	#N/A	#N/A	#N/A	#N/A	-8.64	2.65	2.6E-22	1.7E-19
TRINITY_DN15571_c4_g3_i4	YCIO_SHIFL	#N/A	#N/A	#N/A	#N/A	#N/A	#N/A	#N/A	#N/A	1.12	3.02	9.2E-04	5.5E-02
TRINITY_DN21525_c0_g1_i10	YF48_SCHPO	#N/A	#N/A	#N/A	#N/A	#N/A	#N/A	#N/A	#N/A	2.11	1.12	6.7E-05	5.4E-03
TRINITY_DN20754_c0_g1_i10	YI31B_YEAST	#N/A	#N/A	#N/A	#N/A	#N/A	#N/A	#N/A	#N/A	-2.81	1.82	5.0E-10	9.1E-08
TRINITY_DN21209_c1_g1_i13	YIW4_SCHPO	#N/A	#N/A	#N/A	#N/A	#N/A	#N/A	#N/A	#N/A	8.27	-0.36	8.2E-08	1.1E-05
TRINITY_DN19349_c2_g1_i1	YUID_BACSU	#N/A	#N/A	#N/A	#N/A	#N/A	#N/A	#N/A	#N/A	3.20	1.78	8.8E-05	6.8E-03
TRINITY_DN16534_c1_g1_i1	ZCD_CROSA	#N/A	#N/A	#N/A	#N/A	#N/A	#N/A	#N/A	#N/A	2.26	2.69	1.2E-05	1.1E-03
TRINITY_DN21479_c0_g4_i11	ZDHC2_ARATH	#N/A	#N/A	#N/A	#N/A	#N/A	#N/A	#N/A	#N/A	-5.46	0.12	4.8E-07	5.6E-05
TRINITY_DN21008_c0_g1_i1	ZEP_ORYSJ	#N/A	#N/A	#N/A	#N/A	#N/A	#N/A	#N/A	#N/A	1.83	2.78	3.1E-04	2.1E-02
TRINITY_DN17248_c2_g1_i3	ZIFL1_ARATH	#N/A	#N/A	#N/A	#N/A	#N/A	#N/A	#N/A	#N/A	-2.41	2.23	2.3E-07	2.9E-05
TRINITY_DN21837_c0_g1_i1	ZIFL1_ARATH	#N/A	#N/A	#N/A	#N/A	#N/A	#N/A	#N/A	#N/A	-2.66	1.26	1.7E-05	1.6E-03
TRINITY_DN17248_c2_g1_i5	ZIFL1_ARATH	#N/A	#N/A	#N/A	#N/A	#N/A	#N/A	#N/A	#N/A	-2.32	1.79	1.7E-04	1.2E-02

Table S6 Dry grain quality of the BC2 plants from the field test

Sample Number	genotype	density	DM	ADF	Ca	P	K	Mg	IVTDMD24	NDFD24	IVTDMD30	NDFD30	IVTDMD48	NDFD48	ASH	FAT	Lignin	Starch	ADP	NDF	PROTEIN	ACETIC	CL	CU	FE	LACTIC	MN	MOISTURE	NA	NDFCP	NFC.	NH4	S	SOL_CARBOS	SOL_PROTEIN	SIMPLE_SUGARS	UIP	ZN
C9-6	NT	i2H1	75.1	37.1	0.6	0.3	4.7	0.1	97.2	57.5	91.6	48.0	84.4	61.9	0.0	4.8	0.0	63.7	2.9	54.5	6.0	2.1	-0.3	3.5	158.0	1.1	3.0	24.9	0.0	0.1	45.6	-0.5	0.1	-102.9	39.3	2.0	34.1	21.0
C7-12	NT	c2H2	75.9	34.3	0.7	0.4	4.7	0.1	95.6	53.2	90.6	44.4	87.5	54.7	0.1	3.8	0.0	64.2	2.6	49.2	8.8	2.1	-0.1	3.4	242.3	2.1	2.3	24.1	0.0	0.0	47.9	-0.4	0.1	-99.4	37.7	1.9	33.2	21.0
C9-6	NT	i2H2	71.1	38.6	0.7	0.3	5.5	0.0	99.0	54.7	93.0	42.0	77.4	48.3	0.0	3.4	0.0	72.8	3.4	54.4	8.6	3.0	0.1	2.8	50.4	1.2	-3.9	28.9	-0.1	-0.6	47.1	-0.5	0.1	-137.2	37.8	1.4	34.8	20.6
C9-6	NT	i2H2	75.5	31.9	0.7	0.4	5.2	0.1	102.4	57.7	96.3	46.1	91.2	46.2	0.2	4.6	0.4	74.1	2.7	37.4	10.7	2.8	0.4	3.1	-62.8	1.0	0.0	24.5	0.0	-0.6	52.3	-0.3	0.1	-87.4	38.8	2.9	34.8	20.8
C9-1	NT	c3L2	75.9	33.3	0.7	0.4	4.7	0.1	94.9	51.3	90.2	42.8	85.0	51.3	1.1	3.5	0.0	65.8	2.8	48.0	10.5	2.3	0.1	3.4	136.1	0.9	3.5	24.1	0.0	0.0	46.6	-0.4	0.1	-100.4	36.6	0.7	34.1	21.0
C7-2	NT	c5L2	71.2	37.0	0.5	0.3	4.6	0.0	93.2	51.3	88.5	42.0	72.6	52.1	0.0	3.0	0.0	65.8	3.1	58.4	7.1	2.7	0.0	3.1	134.0	1.5	-1.7	28.8	-0.1	-0.3	45.2	-0.5	0.1	-134.5	38.3	3.4	33.9	20.7
C9-3	NT	i3L2	77.8	32.1	0.5	0.3	4.0	0.1	94.8	53.0	90.2	46.4	83.9	56.2	0.0	4.2	0.0	63.8	2.3	47.7	7.3	2.2	0.0	3.5	150.9	1.4	2.9	22.2	0.0	-0.1	47.0	-0.4	0.1	-82.9	38.9	2.7	33.2	21.0
C9-3	NT	i3l3	80.4	26.1	0.5	0.4	4.1	0.1	95.6	51.6	91.5	43.3	89.5	44.6	1.3	4.3	0.2	67.9	1.9	37.6	9.1	2.2	0.3	3.5	177.6	1.2	4.3	19.6	0.0	-0.2	49.2	-0.4	0.1	-67.9	38.3	1.6	33.5	20.9
C7-12	NT	c2H3	83.6	18.8	0.3	0.2	2.8	0.0	91.6	46.2	88.8	40.5	79.9	38.5	1.8	4.4	1.6	67.4	1.4	31.6	9.4	2.3	0.5	3.6	118.6	0.9	5.8	16.5	0.0	-0.3	49.3	-0.3	0.1	-50.7	39.8	1.4	33.2	20.9
C9-6	NT	i2L1	72.5	39.8	0.7	0.3	5.3	0.0	100.3	59.2	93.8	44.0	80.6	50.8	0.0	3.1	0.0	71.3	3.6	56.4	7.8	2.6	0.0	3.0	30.6	0.5	-1.6	27.5	-0.1	-0.5	46.5	-0.5	0.1	-129.2	36.7	1.0	35.1	20.8
C9-6	NT	i2L3	77.9	32.6	0.7	0.4	4.6	0.1	96.2	52.4	91.3	45.4	87.5	54.1	0.5	4.7	0.0	65.3	2.3	44.8	8.9	2.1	0.1	3.4	205.1	1.4	3.4	22.1	0.0	0.0	47.2	-0.4	0.1	-78.2	38.0	1.2	33.6	21.0
C7-8	NT	c1H1	69.6	45.0	0.7	0.3	5.9	0.0	100.8	61.3	93.7	45.5	80.2	58.0	0.0	3.6	0.0	68.7	4.1	64.2	5.5	2.6	-0.4	3.0	108.0	0.7	-2.7	30.4	-0.1	-0.3	44.8	-0.5	0.1	-155.4	38.4	1.2	34.9	20.9
C9-6	TR	i2H1	76.2	34.1	0.8	0.4	5.4	0.1	104.0	60.0	97.4	48.8	90.9	52.1	0.0	4.8	0.0	74.2	3.0	38.5	8.6	2.7	0.0	3.1	7.4	1.4	-1.4	23.8	0.0	-0.6	53.9	-0.3	0.1	-88.9	40.4	2.6	34.0	20.9
C9-6	TR	i2H1	77.9	28.4	0.5	0.4	4.3	0.1	99.2	55.4	94.1	48.2	91.1	53.6	0.3	5.4	0.0	69.1	2.1	39.2	8.7	2.3	0.1	3.5	129.4	1.5	3.4	22.1	0.0	-0.2	51.7	-0.4	0.1	-70.7	40.9	2.6	33.7	20.9
C9-6	TR	i2H1	79.6	25.8	0.5	0.4	4.1	0.1	95.5	50.0	91.3	43.1	88.6	45.0	1.5	4.3	0.6	67.8	2.0	36.3	10.1	2.3	0.2	3.5	145.9	1.5	4.6	20.4	0.0	-0.2	51.4	-0.3	0.1	-72.3	39.3	1.9	33.3	21.0
C9-6	TR	i2H1	78.2	31.1	0.5	0.3	4.3	0.0	97.3	55.6	92.1	45.4	85.0	51.9	0.1	4.5	0.0	67.9	2.6	45.0	7.0	2.1	-0.1	3.4	107.1	1.0	2.2	21.8	0.0	-0.2	49.7	-0.4	0.1	-87.3	39.3	2.1	33.7	20.9
C7-12	TR	c2H2	74.7	35.0	0.7	0.4	5.5	0.1	104.7	59.7	97.9	45.8	91.4	45.6	0.0	4.6	0.1	77.3	2.9	38.7	7.8	2.8	0.3	2.9	-78.5	1.1	-2.7	25.3	-0.1	-0.8	54.4	-0.4	0.1	-93.1	38.6	3.3	34.6	20.6
C7-12	TR	c2H2	72.5	39.6	0.8	0.5	6.4	0.1	106.7	60.6	99.6	46.2	89.4	43.6	0.0	3.9	0.1	81.2	3.5	42.1	7.7	3.1	0.1	2.6	-110.1	1.4	-6.3	27.5	-0.1	-1.0	54.9	-0.4	0.1	-116.6	39.2	2.7	35.0	20.5
C7-12	TR	c2H2	76.8	32.0	0.6	0.3	4.4	0.1	94.4	53.2	89.5	44.2	82.6	52.8	0.9	3.8	0.0	63.9	2.6	49.8	9.4	2.1	0.0	3.4	182.7	0.6	3.7	23.3	0.0	0.1	45.2	-0.4	0.1	-98.5	37.4	0.7	33.9	21.0
C9-6	TR	i2H2	68.5	40.9	0.6	0.3	6.0	0.0	101.9	57.8	95.3	44.3	71.7	49.3	0.0	3.8	0.4	77.9	3.9	54.1	5.6	3.7	0.0	2.5	-207.0	0.2	-7.3	31.5	-0.1	-1.1	49.0	-0.4	0.1	-157.3	41.1	3.6	35.5	20.5
C9-6	TR	i2H2	75.7	36.1	0.7	0.4	5.4	0.1	102.5	57.3	96.2	46.5	95.6	52.7	0.0	4.7	0.0	72.5	2.8	43.6	7.9	2.2	0.0	3.2	145.7	1.8	-0.1	24.3	-0.1	-0.3	53.2	-0.5	0.1	-91.0	38.6	1.9	33.9	20.8
C9-6	TR	i2H2	72.7	38.2	0.8	0.4	5.7	0.1	103.4	60.9	96.4	46.5	88.2	53.2	0.0	4.3	0.0	73.3	3.6	49.5	7.9	2.5	-0.2	3.1	41.0	1.1	-1.1	27.3	0.0	-0.4	51.4	-0.4	0.1	-123.0	39.2	1.9	34.8	20.8
C9-1	TR	c3L2	72.5	36.0	0.6	0.3	5.4	0.0	100.9	59.0	94.6	46.9	83.2	52.9	0.0	4.6	0.0	71.8	3.3	49.8	7.7	2.9	0.0	3.2	-11.9	0.3	-0.3	27.6	0.0	-0.4	48.7	-0.4	0.1	-125.6	41.0	1.4	35.1	20.9
C9-1	TR	c3L2	71.6	37.6	0.7	0.3	5.6	0.0	99.8	56.1	93.9	44.7	79.8	49.7	0.0	3.5	0.0	72.8	3.3	50.8	7.9	3.0	0.1	2.9	-23.9	1.1	-3.6	28.4	-0.1	-0.6	48.9	-0.4	0.1	-129.8	39.4	2.6	34.6	20.7
C9-3	TR	i3L2	73.4	39.9	0.7	0.3	5.7	0.0	105.4	61.5	98.4	48.5	83.3	52.9	0.0	4.4	0.1	78.9	3.6	46.2	5.2	3.0	0.2	2.7	-204.1	0.1	-4.8	26.6	-0.1	-1.0	51.3	-0.4	0.1	-111.8	39.6	3.0	35.5	20.6
C9-3	TR	i3L2	70.9	43.2	0.7	0.4	6.1	0.0	104.4	60.3	97.4	46.7	82.0	52.7	0.0	4.6	0.0	76.5	3.6	53.3	5.4	3.0	0.0	2.7	-83.7	1.0	-5.4	29.1	-0.1	-0.8	50.1	-0.5	0.1	-126.7	39.7	2.4	35.3	20.6
C9-3	TR	i3L2	75.9	30.2	0.6	0.3	5.0	0.0	100.4	55.3	95.0	45.7	78.8	45.3	0.1	4.2	1.0	76.1	3.0	38.3	8.8	3.2	0.3	3.0	-210.3	0.7	-1.6	24.2	0.0	-0.8	52.1	-0.2	0.1	-102.6	41.5	3.0	34.7	20.8
C7-12	TR	c2H3	77.5	33.6	0.6	0.4	4.7	0.1	96.7	52.7	92.0	46.9	89.3	57.2	0.2	4.9	0.0	66.0	2.4	45.3	7.3	2.0	-0.1	3.5	155.6	1.4	3.6	22.5	0.0	0.0	49.2	-0.4	0.1	-81.1	39.6	1.3	33.5	21.0
C7-12	TR	c2H3	72.7	37.7	0.7	0.4	5.6	0.1	103.9	61.3	97.0	47.0	87.5	49.9	0.0	4.4	0.0	75.6	3.3	46.6	6.4	2.7	0.1	3.0	-81.9	0.4	-2.5	27.3	-0.1	-0.7	51.4	-0.4	0.1	-110.6	38.8	3.2	35.2	20.6
C9-6	TR	i2L3	73.8	39.5	0.8	0.4	5.8	0.1	102.5	58.0	96.1	47.9	90.0	57.1	0.0	5.0	0.0	72.3	3.1	48.1	6.6	2.4	-0.1	3.0	76.2	1.3	-1.0	26.3	-0.1	-0.4	51.2	-0.5	0.1	-103.0	39.2	1.8	34.6	20.8
C9-6	TR	i2L3	72.4	43.0	0.9	0.4	6.3	0.1	105.9	61.4	98.6	48.2	92.6	55.8	0.0	4.4	0.0	76.9	3.7	49.6	6.6	2.4	-0.2	2.8	10.9	1.2	-3.8	27.6	-0.1	-0.6	53.1	-0.5	0.1	-116.3	38.3	1.4	35.0	20.6
C9-6	TR	i2L3	72.3	38.3	0.7	0.4	5.5	0.1	101.5	60.5	94.5	48.2	82.9	59.3	0.0	4.4	0.0	69.2	3.5	52.7	8.8	2.7	-0.1	3.1	-8.9	0.2	-0.6	27.7	0.0	-0.3	47.1	-0.4	0.1	-119.3	39.0	1.6	34.9	21.0
C7-8	TR	c1H1	73.6	36.6	0.7	0.4	5.4	0.1	100.6	56.6	94.8	47.5	86.6	53.4	0.0	5.0	0.0	71.1	2.8	46.4	6.6	2.6	-0.1	3.2	33.0	1.4	-0.1	26.5	0.0	-0.4	50.9	-0.4	0.1	-102.2	41.0	2.7	34.3	20.8
C7-8	TR	c1H1	77.0	30.4	0.5	0.3	4.5	0.0	96.8	53.6	92.1	45.8	81.6	47.8	0.0	4.6	1.1	69.9	2.3	40.7	7.5	2.5	0.2	3.2	-60.9	0.5	1.7	23.0	0.0	-0.4	49.5	-0.3	0.1	-81.7	39.7	3.4	34.5	20.8
C7-8	TR	c1H1	75.1	32.6	0.6	0.4	5.4	0.0	105.0	60.5	98.3	45.9	87.3	42.8	0.0	4.6	0.4	79.9	3.0	36.2	7.2	3.1	0.3	2.8	-170.3	0.5	-3.8	25.0	-0.1	-1.0	55.0	-0.3	0.1	-99.2	40.0	4.0	34.9	20.6
B73	WT	L	74.6	32.3	0.9	0.4	5.1	0.1	99.5	57.2	93.6	43.9	85.5	44.8	0.9	3.0	0.2	71.4	3.3	42.3	12.3	2.7	0.1	3.2	-31.2	0.7	0.7	25.4	0.0	-0.4	50.0	-0.3	0.1	-113.0	37.6	1.8	34.5	20.9
B73	WT	L	64.3	50.2	0.8	0.3	6.6	0.0	102.3	63.8	94.4	43.3	72.0	52.8	0.0	2.1	0.0	72.4	5.1	71.9	4.8	3.2	-0.6	2.7	26.8	0.9	-6.7	35.7	-0.1	-0.6	45.0	-0.5	0.1	-205.2	38.2	2.8	35.7	20.7
B73	WT	H	72.6	37.2	0.8	0.4	5.6	0.0	100.4	55.9	94.3	44.2	79.3	46.4	0.0	3.7	0.9	75.3	3.4	45.7	8.9	3.1	0.3	2.8	-151.7	0.3	-4.0	27.4	-0.1	-0.8	49.1	-0.4	0.1	-117.2	38.0	2.3	35.0	20.6
B73	WT	L	70.7	39.4	0.8	0.3	5.3	0.0	96.7	54.0	91.2	40.3	72.0	43.6	0.1	2.0	0.1	70.9	3.9	56.4	9.4	3.0	-0.1	2.8	30.6	1.0	-3.2	29.3	-0.1	-0.5	45.3	-0.4	0.1	-156.2	38.0	0.6	34.6	20.8
B73	WT	H	69.1	39.2	0.6	0.3	5.4	0.0	98.2	56.3	92.3	43.7	71.2	47.6	0.0	3.0	0.4	72.9	3.8	55.2	7.0	3.2	-0.1	2.8	-109.0	0.6	-4.6	30.9	-0.1	-0.7	47.1	-0.4	0.1	-153.8	40.0	2.6	35.0	20.7
B73	WT	L	73.4	32.5	0.7	0.4	5.4	0.0	102.0	56.5	95.9	44.4	83.1	43.1	0.0	4.2	0.5	77.7	3.0	41.2	9.3	3.2	0.3	2.8	-74.3	0.8	-3.9	26.6	-0.1	-0.9	51.9	-0.4	0.1	-112.6	40.0	2.3	34.7	20.6
B73	WT	L	77.2	33.3	0.6	0.4	5.1	0.1	98.7	56.1	93.2	46.9	88.3	51.9	0.1	4.7	0.4	68.6	2.6	42.7	8.0	2.3	0.2	3.3	-17.0	0.6	2.4	22.8	0.0	-0.2	49.1	-0.4	0.1	-80.0	38.3	2.7	34.3	20.9
B73	WT	H	70.4	40.9	0.8	0.4	6.0	0.0	106.8	66.2	99.1	47.0	83.2	46.9	0.0	3.9	0.0	77.2	3.9	51.2	7.2	3.1	0.0	2.8	-161.3	0.4	-4.4	29.6	-0.1	-0.9	49.5	-0.4	0.1	-133.0	39.4	2.6	35.9	20.7
B73	WT	H	61.5	55.5	1.0	0.4	7.8	0.1	108.2	66.8	99.1	45.6	79.2	54.4	0.0	3.0	0.0	79.3	5.4	71.3	4.1	3.4	-0.7	2.4	15.6	0.8	-10.3	38.5	-0.1	-0.9	48.4	-0.6	0.1	-212.1	39.4	1.4	36.3	20.5
B73	WT	H	67.5	50.4	0.8	0.3	6.4	0.0	102.2	63.4	94.7	45.0	76.8	54.6	0.0	2.9	0.0	71.5	4.7	69.6	4.1	2.7	-0.5	2.9	55.0	0.6	-4.1	32.5	-0.1	-0.4	45.0	-0.5	0.1	-179.6	38.2	0.7	35.6	20.8
B73	WT	L	71.7	37.2	0.7	0.3	5.6	0.0	102.5	60.5	95.7	45.1	82.1	47.2	0.0	3.6	0.0	76.9	3.6	48.9	7.3	2.9	0.1	2.8	-94.8	0.2	-4.4	28.3	-0.1	-0.8	50.9	-0.5	0.1	-129.2	38.4	2.2	35.2	20.5
B73	WT	L	68.6	40.3	0.7	0.3	5.5	0.0	97.4	54.4	91.7	41.7	69.2	47.8	0.0	2.6	0.0	73.2	3.9	57.9	7.0	3.4	0.0	2.6	5.5	0.8	-6.5	31.4	-0.1	-0.8	44.9	-0.5	0.1	-163.3	39.0	2.0	34.8	20.6
B73	WT	L	69.9	38.1	0.9	0.4	6.3	0.0	108.3	64.1	100.3	44.8	88.8	43.0	0.0	3.8	0.0	82.7	3.9	42.8	9.7	3.4	0.3	2.5	-148.6	0.6	-6.8	30.1	-0.1	-1.1	54.6	-0.4	0.1	-135.2	38.9	2.8	35.6	20.5
B73	WT	H	71.7	37.2	0.9	0.4	5.8	0.1	103.5	60.5	96.6	45.4	87.1	46.8	0.0	3.7	0.0	75.1	3.4	46.2	10.7	2.9	0.2	2.8	-39.0	1.2	-3.2	28.3	-0.1	-0.7	50.6	-0.4	0.1	-116.8	37.2	2.8	35.2	20.7
B73	WT	L	70.8	39.2	0.8	0.4	6.1	0.0	107.1	66.6	99.3	45.0	84.0	41.1	0.0	2.8	0.0	80.5	4.2	47.8	8.0	3.2	0.1	2.7	-238.4	0.1	-5.1	29.2	-0.1	-1.1	51.9	-0.4	0.1	-138.9	38.0	2.4	36.0	20.6
NT_AVG	NT		75.5	33.9	0.6	0.3	4.7	0.1	96.8	54.1	91.6	44.2	83.3	51.4	0.4	3.9	0.2	67.6	2.7	48.7	8.3	2.4	0.1	3.2	120.7	1.1	1.3	24.5	0.0	-0.2	47.4	-0.4	0.1	-102.2	38.2	1.8	34.0	20.9
TR_AVG	TR		74.4	35.6	0.7	0.4	5.3	0.1	101.5	57.7	95.3	46.5	86.1	51.2	0.1	4.5	0.2	73.1	3.0	45.3	7.5	2.7	0.1	3.0	-9.4	0.9	-1.2	25.6	0.0	-0.5	51.0	-0.4	0.1	-104.7	39.6	2.4	34.5	20.8
WT_AVG	WT		70.3	40.2	0.8	0.3	5.9	0.0	102.3	60.1	95.4	44.4	80.1	47.4	0.1	3.3	0.2	75.0	3.9	52.7	7.9	3.0	0.0	2.8	-62.1	0.6	-4.3	29.7	-0.1	-0.7	48.9	-0.4	0.1	-143.1	38.6	2.1	35.2	20.7
NT_STDEV	NT		4.1	6.7	0.1	0.1	0.8	0.0	3.2	4.2	2.2	2.2	5.4	6.4	0.6	0.6	0.5	3.5	0.7	9.6	1.6	0.3	0.3	0.3	82.4	0.4	3.1	4.1	0.0	0.2	2.1	0.1	0.0	31.4	0.9	0.8	0.7	0.1
TR_STDEV	TR		2.7	4.6	0.1	0.0	0.7	0.0	3.5	3.3	2.7	1.5	5.4	4.5	0.4	0.4	0.3	4.6	0.5	5.6	1.3	0.4	0.2	0.3	122.3	0.5	3.3	2.7	0.0	0.3	2.5	0.1	0.0	20.8	1.0	0.8	0.7	0.2
WT_STDEV	WT		3.9	6.8	0.1	0.1	0.7	0.0	3.9	4.7	2.9	1.7	6.6	4.2	0.2	0.8	0.3	4.0	0.8	10.8	2.3	0.3	0.3	0.2	86.3	0.3	3.0	3.9	0.0	0.3	2.9	0.1	0.0	36.1	0.8	0.7	0.6	0.1
