# Supplementary material for: Utilizing MIKC-type MADS-box protein SOC1 for yield potential enhancement in maize
Source: Plant Cell Rep. 2021 Jun 6;40(9):1679–93. doi: 10.1007/s00299-021-02722-4 (PMC8376726; doi:10.1007/s00299-021-02722-4)
Supplement: Supplementary file 2 — Supplementary file2 (PPTX 14277 KB) [file 299_2021_2722_MOESM2_ESM.pptx]

## Slide 1
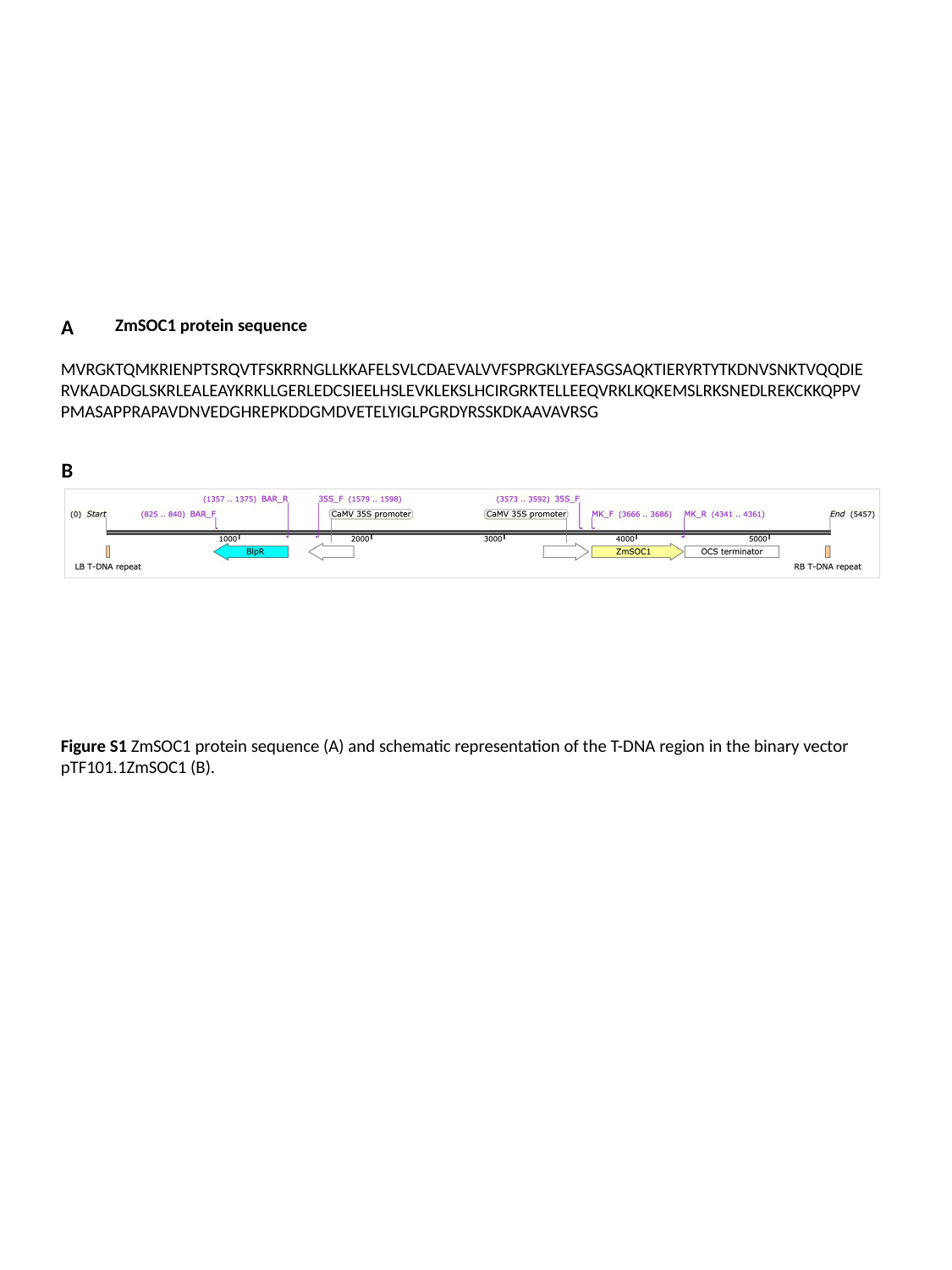

A
ZmSOC1 protein sequence
MVRGKTQMKRIENPTSRQVTFSKRRNGLLKKAFELSVLCDAEVALVVFSPRGKLYEFASGSAQKTIERYRTYTKDNVSNKTVQQDIERVKADADGLSKRLEALEAYKRKLLGERLEDCSIEELHSLEVKLEKSLHCIRGRKTELLEEQVRKLKQKEMSLRKSNEDLREKCKKQPPVPMASAPPRAPAVDNVEDGHREPKDDGMDVETELYIGLPGRDYRSSKDKAAVAVRSG
B
Figure S1 ZmSOC1 protein sequence (A) and schematic representation of the T-DNA region in the binary vector pTF101.1ZmSOC1 (B).

## Slide 2
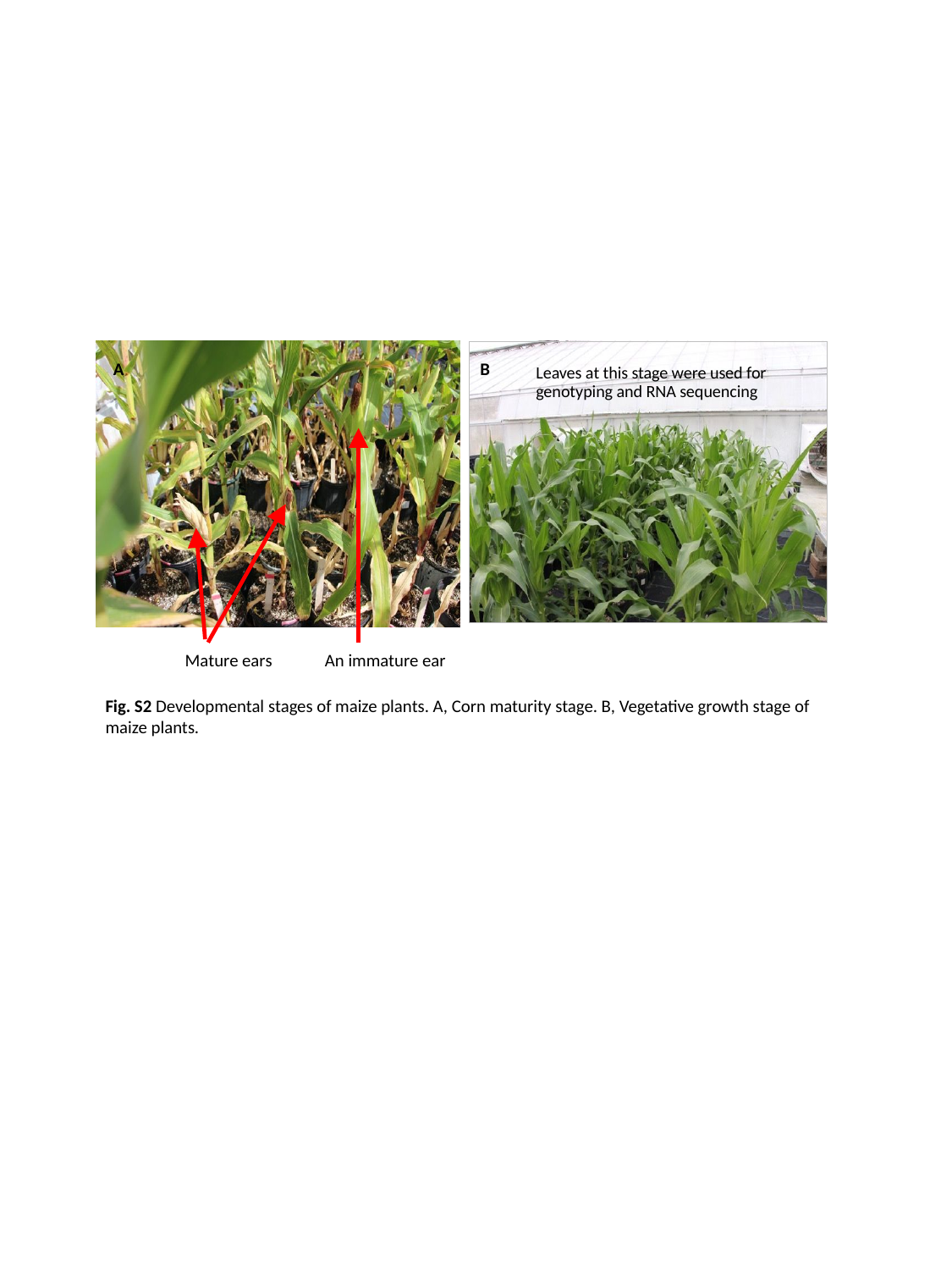

B
A
Leaves at this stage were used for genotyping and RNA sequencing
An immature ear
Mature ears
Fig. S2 Developmental stages of maize plants. A, Corn maturity stage. B, Vegetative growth stage of maize plants.

## Slide 3
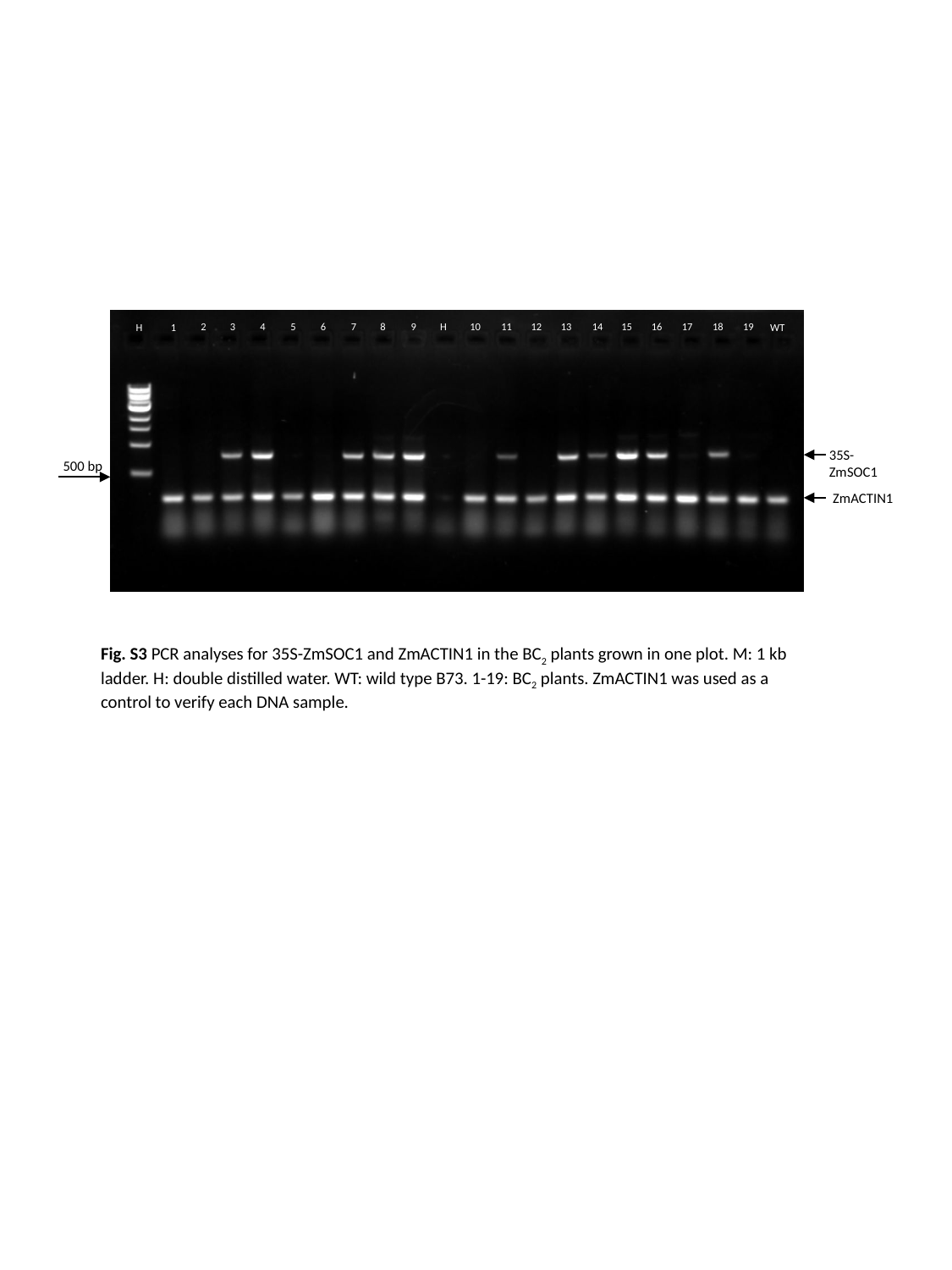

2
3
4
5
6
7
8
9
H
10
11
12
13
14
15
16
17
18
19
H
1
WT
35S-ZmSOC1
500 bp
ZmACTIN1
Fig. S3 PCR analyses for 35S-ZmSOC1 and ZmACTIN1 in the BC2 plants grown in one plot. M: 1 kb ladder. H: double distilled water. WT: wild type B73. 1-19: BC2 plants. ZmACTIN1 was used as a control to verify each DNA sample.

## Slide 4
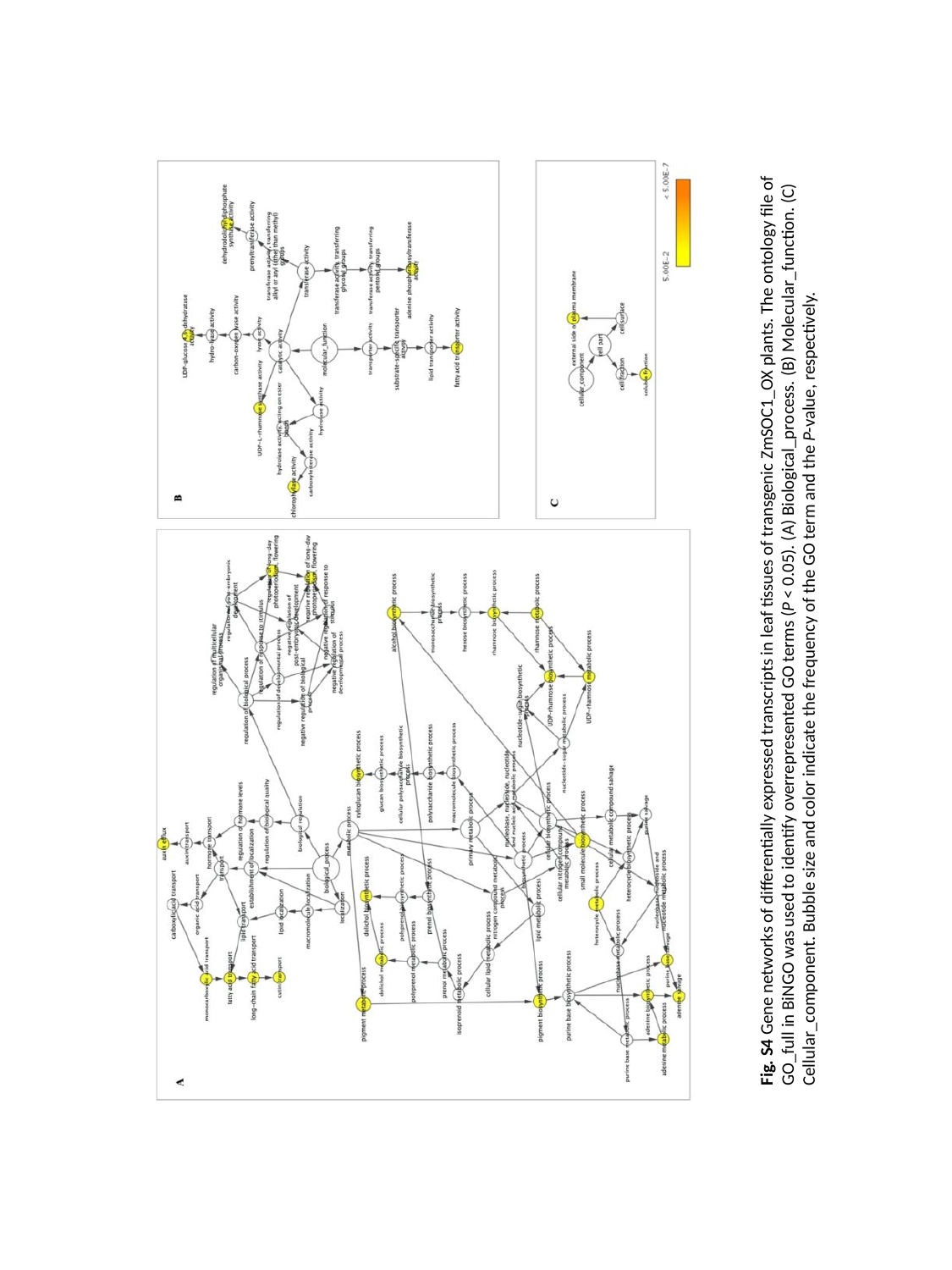

Fig. S4 Gene networks of differentially expressed transcripts in leaf tissues of transgenic ZmSOC1_OX plants. The ontology file of GO_full in BiNGO was used to identify overrepresented GO terms (P < 0.05). (A) Biological_process. (B) Molecular_function. (C) Cellular_component. Bubble size and color indicate the frequency of the GO term and the P-value, respectively.
